# Systematics of the parasitic wasp genus *Oxyscelio* Kieffer (Hymenoptera, Platygastridae s.l.), part II: the Australian and southwest Pacific fauna

**DOI:** 10.3897/zookeys.331.5152

**Published:** 2013-09-13

**Authors:** Roger A. Burks, Lubomír Masner, Norman F. Johnson, Andrew D. Austin

**Affiliations:** 1 Department of Evolution, Ecology, and Organismal Biology, The Ohio State University, 1315 Kinnear Road, Columbus, OH 43212, U.S.A.; 2 Agriculture and Agri-Food Canada, K.W. Neatby Building, Ottawa, ON K1A 0C6, Canada; 3 Australian Centre for Evolutionary Biology and Biodiversity, School of Earth and Environmental Sciences, The University of Adelaide, SA 5005, Australias

**Keywords:** Platygastroidea, Scelionidae, Scelioninae, key, revision, database, parasitoid

## Abstract

The Australasian and southwest Pacific species of *Oxyscelio* (Hymenoptera: Platygastridae s.l.) are revised. A total of 80 species are recognized as valid, 13 of which are redescribed: *O. atricoxa* (Dodd), *O. concoloripes* (Dodd), *O. flavipes* (Kieffer), *O. grandis* (Dodd), *O. hyalinipennis* (Dodd), *O. magniclava* (Dodd), *O. mirellus* (Dodd), *O. montanus* (Dodd), *O. nigriclava* (Dodd), *O. nigricoxa* (Dodd), *O. rugulosus* (Dodd), *O. shakespearei* (Girault), and *O. solitarius* (Dodd). *Oxyscelio glabriscutellum* (Dodd) **syn. n.** is placed as a subjective junior synonym of *O. rugulosus*. Sixty-seven new species are described, many representing new distributional records for the genus - *O. aciculae* Burks, **sp. n.**, *O. anfractus* Burks, **sp. n.**, *O. bellariorum* Burks, **sp. n.**, *O. bicoloripedis* Burks, **sp. n.**, *O. brevitas* Burks, **sp. n.**, *O. catenae* Burks, **sp. n.**, *O. caudarum* Burks, **sp. n.**, *O. circulorum* Burks, **sp. n.**, *O. clivi* Burks, **sp. n.**, *O. clupei* Burks, **sp. n.**, *O. conjuncti* Burks, **sp. n.**, *O. contusionis* Burks, **sp. n.**, *O. corrugationis* Burks, **sp. n.**, *O. croci* Burks, **sp. n.**, *O. cuspidis* Burks, **sp. n.**, *O. densitatis* Burks, **sp. n.**, *O. dissimulationis* Burks, **sp. n.**, *O. divisionis* Burks, **sp. n.**, *O. exiguitatis* Burks, **sp. n.**, *O. fluctuum* Burks, **sp. n.**, *O. foliorum* Burks, **sp. n.**, *O. funis* Burks, **sp. n.**, *O. gressus* Burks, **sp. n.**, *O. hamorum* Burks, **sp. n.**, *O. incisurae* Burks, **sp. n.**, *O. lenitatis* Burks, **sp. n.**, *O. leviventris* Burks, **sp. n.**, *O. limbi* Burks, **sp. n.**, *O. liminis* Burks, **sp. n.**, *O. linguae* Burks, **sp. n.**, *O. lintris* Burks, **sp. n.**, *O. livens* Burks, **sp. n.**, *O. mystacis* Burks, **sp. n.**, *O. nasi* Burks, **sp. n.**, *O. nitoris* Burks, **sp. n.**, *O. obliquiatis* Burks, **sp. n.**, *O. oblongiclypei* Burks, **sp. n.**, *O. obturationis* Burks, **sp. n.**, *O. oculi* Burks, **sp. n.**, *O. palati* Burks, **sp. n.**, *O. pectinis* Burks, **sp. n.**, *O. pollicis* Burks, **sp. n.**, *O. proceritatis* Burks, **sp. n.**, *O. productionis* Burks, **sp. n.**, *O. radii* Burks, **sp. n.**, *O. rami* Burks, **sp. n.**, *O. rupturae* Burks, **sp. n.**, *O. sarcinae* Burks, **sp. n.**, *O. scismatis* Burks, **sp. n.**, *O. sciuri* Burks, **sp. n.**, *O. scutorum* Burks, **sp. n.**, *O. sepisessor* Burks, **sp. n.**, *O. sinuationis* Burks, **sp. n.**, *O. sordes* Burks, **sp. n.**, *O. spatula* Burks, **sp. n.**, *O. stipulae* Burks, **sp. n.**, *O. stringerae* Burks, **sp. n.**, *O. tenuitatis* Burks, **sp. n.**, *O. truncationis* Burks, **sp. n.**, *O. tubi* Burks, **sp. n.**, *O. umbonis* Burks, **sp. n.**, *O. uncinorum* Burks, **sp. n.**, *O. valdecatenae* Burks, **sp. n.**, *O. velamenti* Burks, **sp. n.**, *O. verrucae* Burks, **sp. n.**, *O. viator* Burks, **sp. n.**, and *O. wa* Burks, **sp. n.** The fauna is divided into nine diagnostic species groups, with five species unplaced to group.

## Introduction

The genus *Oxyscelio* Kiefer is comprised of relatively robust platygastroid wasps that occur across equatorial and east Africa, Madagascar, the southeastern part of the Palaearctic, and the Indo-Malayan, Australasian and southwest Pacific regions. They are relatively easily identified by the fore wing submarginal vein being distant from wing margin, the marginal vein being very short, a postmarginal vein being virtually absent, and the metascutellum being plate-like. In addition, many species can be recognised by the presence of a frontal depression on the head.

This is the second of three studies aimed at revising the global fauna of *Oxyscelio*, the southeastern Palaearctic and Indo-Malayan species having been completed recently (Burks et al. 2013) with the African-Malagasy fauna currently in preparation. A more detailed taxonomic history, generic description and bibliography of the genus are presented in the first paper.

Previously, 14 species were described from the region, all from the Australian mainland and all prior to 1930. Dodd (1913, 1914, 1920) was responsible for 12 species, with a single species each described by Kieffer (1907) and Girault (1926). Since then, very large amounts of material have been collected, mostly in the last 30 years using modern collecting techniques, particularly yellow pan and Malaise traps. The current study treats the Australasian and southwest Pacific taxa, and we recognize 67 new species, redescribe 13 species, and recommend one new synonymy. This work has arisen from our Platygastroidea Planetary Biodiversity Inventory (see below), which aims to revise all species on a worldwide basis for a number of important platygastroid genera.

The contributions of the individual authors are as follows; R.A. Burks: character definition, species concept development; key development, imaging, capture of specimen data, manuscript preparation, phylogenetic analysis and illustration; L. Masner: specimen acquisition, and generic overview; N.F. Johnson: generic concept development and manuscript preparation; A.D. Austin: project planning, species concept discussions, manuscript preparation, and taxonomic overview.

## Materials and methods

Specimens examined were provided by the following collections: The American Entomological Institute, Gainesville, Florida, USA (AEIC)^1^; Australian Museum, Sydney, Australia (AMSA)^2^; Australian National Insect Collection, Canberra, Australia (ANIC)^3^; New South Wales Department of Primary Industries, Agricultural Scientific Collections Unit, Orange, Australia (ASCU)^4^; The Natural History Museum, London, United Kingdom (BMNH)^5^; Canadian National Collection of Insects, Arachnids and Nematodes, Ottawa, Canada (CNCI)^6^; Illinois Natural History Survey, Urbana, IL Illinois, USA (INHS)^7^; Institut Royal des Sciences Naturelles de Belgique, Bruxelles, Belgium (ISNB)^8^; Museum of Comparative Zoology, Harvard University, Cambridge, Massachusetts, USA (MCZC)^9^; Muséum National d’Histoire Naturelle, Paris, France (MNHN)^10^; Museum of Victoria, Entomology, Melbourne, Australia (MVMA)^11^; National Museum of Natural History, Washington, DC, USA (NMNH)^12^; C.A. Triplehorn Insect Collection, Ohio State University, Columbus, Ohio, USA (OSUC)^13^; Queensland Primary Industries and Fisheries Insect Collection, Indooroopilly, Australia (QDPC)^14^; Queensland Museum, Brisbane, Australia (QMBA)^15^; Queensland Primary Industries Insect Collection, Mareeba, Australia (QPIM)^16^; Royal Museum of Central Africa, Tervuren, Belgium (RMCA)^17^; South Australian Museum, Adelaide, Australia (SAMA)^18^; South African National Collection of Insects, Pretoria, South Africa (SANC)^19^; Tasmanian Department of Primary Industries and Water, Hobart, Australia (TDAH)^20^; University of California, Riverside, California, USA (UCRC)^21^; University of Queensland Insect Collection, Brisbane, Australia (UQIC)^22^; National Museum of Natural History, Washington, DC, USA (USNM)^23^; Western Australian Museum, Perth, Australia (WAMP)^24^; Waite Insect and Nematode Collection, Adelaide, Australia (WINC)^25^.

This revision is a product of the Platygastroidea Planetary Biodiversity Inventory, funded by the U.S. National Science Foundation (N.F. Johnson, Ohio State University; A.D. Austin, University of Adelaide; Principal Investigators). An objective of this project is to use biodiversity informatics resources to accelerate taxonomic work, making real-time collaboration possible. Data associated with specimens examined in this study can be accessed at hol.osu.edu by entering the unique specimen identifier (e.g. OSUC 359541) in the search form. Life science identifiers (LSIDs) can be resolved at http://zoobank.org (i.e. http://zoobank.org/99E3E72E-DA88-4740-9ECB-2D03BCD1DACE).

Morphological terminology follows Mikó et al. (2007) except as follows. Ovipositor terminology is used as described by Austin and Field (1997). “Middle genal carina” is the largest carina subparallel to the eye, but between the genal carina and the carina immediately encircling the eye; it has proven to be recognizable as homologous (when present) in *Oxyscelio*. “Mesoscutal midlobe” refers to the area of the dorsal surface of the mesoscutum that is between notauli; in rare cases where notauli are not indicated, this is determined ultimately through comparison with other species. “T1 midlobe” refers to the raised antero-medial area of T1 that is flanked by depressed lateral areas. This is usually flat and only weakly elevated in *Oxyscelio*, and therefore is not strictly the same as a T1 horn, but a T1 midlobe can be expressed as a T1 horn. “Metasomal flange” refers to the “fins” or “fin-like structures” discussed by Masner (1976), which he had named in reference to the fins found on some automobiles; defined here as a flange that is present on the lateral or posterolateral edge of a metasomal tergite.

Surface sculpture terminology follows Eady (1968) in most cases and Burks et al. (2013) in some novel interpretations of major sculpture versus microsculpture, which are repeated here with some minor clarifications. It should be stressed that our system of sculptural description here is intended to report only what we saw in *Oxyscelio*, and is not intended to be appropriate across all other taxa. This is in part because the causes and true extent of variation of surface-sculpture variation are as a general rule not well known – the causes of variation may differ in other taxa, and the ways of variation may differ in other taxa.

**Figure 1a–1d.**
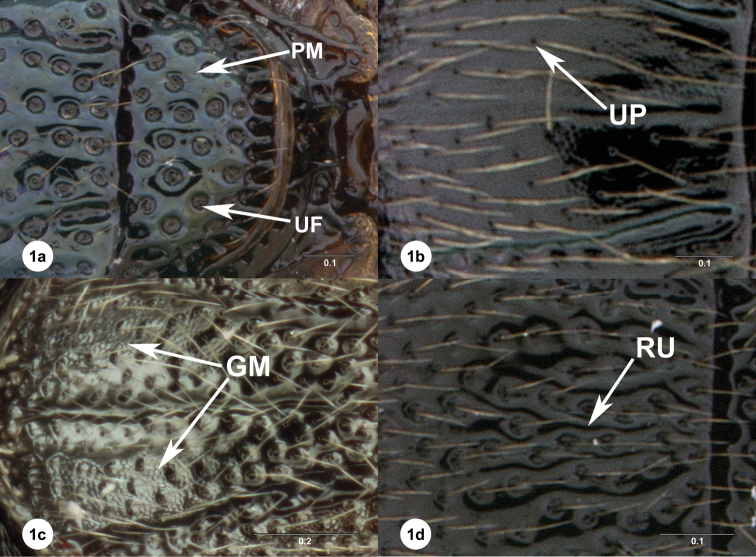
Examples of surface sculpture in *Oxyscelio*. *Oxyscelio livens* sp. n. holotype female (OSUC 148485) **1a** Mesoscutellum, PM = punctate microsculpture (arrow indicates an individual punctum), UF = umbilicate fovea. *Oxyscelio nasi* sp. n. holotype male (OSUC 368915) **1b** Mesoscutum, UP = umbilicate punctum. *Oxyscelio striarum* Burks paratype female (OSUC 257067) **1c** Mesoscutum, GM = granulate microsculpture. *Oxyscelio sepisessor* sp. n. holotype female (OSUC 438936) **1d** Mesoscutum, RU = ruga.

Diminutive variant terms (such as “foveolate” or “rugulose”) were avoided because of a lack of criteria for separating them from non-diminutive alternatives. “Major” surface sculpture refers to repeated sculptural patterns that interact with seta placement. It does not include non-repeated elements or those which are repeated only once due to bilateral symmetry. “Umbilicate-foveate” sculpture refers to rounded crater-like sculptural elements, each surrounding a setiferous punctum (and thus interacting with a seta), with each fovea being much larger than its setiferous punctum and spatially separated from it (Fig. 1a: UF). Umbilicate-punctate sculpture indicates that no sculptural element accompanies the setiferous punctum – and therefore the setiferous punctum is the “major” surface sculpture element here (Fig. 1b: UP). Rugose sculpture refers to branching or wrinkling elevations that flank setiferous puncta but do not fully surround them (Fig. 1d: RU). Where both umbilicate-foveate and umbilicate-punctate sculpture are reported for the same sclerite, this should be interpreted as variable sculpture where some setiferous puncta are surrounded by foveae while others are not – and such variation may occur in a single species or specimen. Under this scheme, “major” surface sculpture cannot occur in any part of the sclerite that lacks setae.

“Microsculpture” refers to repeated sculptural elements that do not interact with seta placement. Microsculpture can occur on “major” sculptural elements, such as on rugae and on all surfaces of foveae. Punctate microsculpture refers to tiny round pits that do not bear setae (Fig. 1a: PM). Granulate microsculpture refers to sculpture that is similar to that of leather or skin, with a network of grooves (Fig. 1c: GM). Microsculpture can occur in areas that lack setae. While there are other ways of classifying surface sculpture according to subjective differences in interests, we use this method because we maintain that major sculpture and microsculpture are mutually exclusive classes. One might maintain that “smoothness” plays a role in sculptural classification, but we find that word to be problematic because it describes an absence of sculptural variation, not the specific absence of one definable type of sculpture. We are much more satisfied with descriptors that refer to particular types of sculpture.

One of the difficulties in defining surface sculpture using hierarchies and genus-differentia formats is that there is more than one valid way of classifying surface sculpture into Aristotelian genera. We maintain that the art of creating genus-differentia definitions, although ancient, is not yet at a methodologically mature state. Another problem is that variation can render some commonly-used sculptural classifications problematic. Finally, surface sculpture has typically been named and defined using only vague shape or pattern-based comparisons to other, better known entities (such as reticulate = net-like), and not through more purely logical means. Umbilicate-foveate sculpture could be called “reticulate,” for instance, because of a net-like pattern of foveae. However, in some Australian *Oxyscelio* this is not clearly reticulate because only very sparsely distributed umbilicate foveae may be present, with very broad interspaces—and variation from a pattern that appears reticulate to a much more sparse pattern can occur within species. For that reason, we have avoided using the term “reticulate” for *Oxyscelio* even though it is valid in many other taxa. Others could dispute this assertion, but we maintain that this would reflect only differences in subjective perspectives.

We assert that the word “pit” is a word but not a specialized term, having terminological importance only as part of a two-word term that includes a specialized term as a qualifier, as in “tentorial pit.” For that reason, we have changed from using “pit” to using “punctum” (the Latin word for “puncture”) here. We make no distinction between types of puncti other than to describe them as setiferous or not, but other criteria for classifying puncti probably exist for *Oxyscelio*. For raised sculptural elements, some might claim that all raised sculpture is the same unless it can be related to an internal structure, but we maintain that actual word synonymy implies that the words are truly interchangeable. For instance, “carina” and “ruga” are synonymous only if every instance of “carina” could also be referred to as “ruga,” and vice versa. We maintain that this is not true for carina versus ruga, and therefore we consider those terms distinct. The term “stria” has historically been used for repeating linear elements that form a localized pattern in certain cases, and we uphold previous usage here. For certain “carinae” that are named as particular structures, their shape can be irregular but we have followed established usage for naming those structures. We maintain that terms such as “occipital carina” are two-word terms that refer consistently to a homologous structure regardless of that structure’s actual shape, for that reason. For the sculptural terms used here, we recommend the following definitions:

carina: The process that is linear and is regular.

fovea: The depression that is ovate and is shallow relative to its diameters.

granulate: The sculptural pattern that is a network of branched impressions.

interspace: The area that is between sculptural elements.

major sculpture: The sculptural pattern that is associated with seta placement.

microsculpture: The sculptural pattern that is not associated with seta placement.

punctate microsculpture: The sculptural pattern that is comprised of puncta.

punctum: The depression that is ovate and is deep relative to its diameters.

ruga: The process that is linear and is irregular.

sculptural element: The anatomical structure that is one unit in a sculptural pattern.

sculptural pattern: The pattern that is formed by repeated adjacent surface sculpture.

setiferous fovea: The fovea that surrounds a seta.

setiferous punctum: The punctum that surrounds a seta.

sculptural septum: The septum that separates sculptural elements.

surface sculpture: The anatomical structure that is texture.

umbilicate-foveate: The sculptural pattern that consists of setiferous foveae.

umbilicate-punctate: The sculptural pattern that consists of only setiferous puncta.

**Illustration and data citations.** Photographs were taken using one of the following systems: 1) Visionary Digital BK+ Imaging System, November 2010 model, with either a K2 Long Distance Microscope or a 65 mm varifocal lens; 2) Synoptics, Ltd. system using a Leica Z16 APO microscope and a JVC KY-F75U 3-CCD camera; 3) GT EntoVision Mobile Imaging System, or 4) for microscope slides, a Leica Integrated System using a Leica 205c microscope with a DFC500 camera and 5000HDI illuminator. Source photos were stacked using Zerene Stacker version 1.04, Auto-Montage Pro version 5.01.0005, or Leica Application Suite, and enhanced using Adobe Photoshop CS5 or CS6.

## Taxonomy

### Oxyscelio

Kieffer

http://zoobank.org/99E3E72E-DA88-4740-9ECB-2D03BCD1DACE

urn:lsid:biosci.ohio-state.edu:osuc_concepts:529

http://species-id.net/wiki/Oxyscelio

Oxyscelio Kieffer, 1907: 310. Original description. Type: *Oxyscelio foveatus* Kieffer, by monotypy.
Oxyscelio See Burks et al. (2013) for complete bibliography of the genus.

#### Description.

Body length: 2.6–7.1 mm.

Head shape in dorsal view: weakly transverse, width approximately 1.5x greatest length; subquadrate. Hyperoccipital carina: absent; present. Occipital carina: present, complete medially; present, broadly interrupted medially. Occipital carina sculpture: crenulate. Ocular ocellar line (OOL): OOL < 0.5 ocellar diameter (OD). Dorsal area of frons: convex, without frontal shelf. Antennal scrobe shape: present, unmargined; scrobe margined by carina. Frons sculpture: umbilicate-punctate, with transverse carinae within scrobe; scrobe largely smooth, otherwise with transverse carinae. Submedian carina: absent. Orbital carina: absent. Inner orbits: diverging ventrally. Interocular space(IOS)/Eye height (EH): IOS distinctly less than EH. Interantennal process: triangular in lateral view. Central keel: absent. Antennal foramen opening: oriented laterally on interantennal process. Facial striae: present. Malar sulcus: present. Compound eye size: not significantly reduced. Compound eye setation: absent. Gena: weakly convex, receding behind posterior orbit; convex, distinctly produced behind eye. Clypeus shape: narrow, slightly convex medially, lateral corner not produced. Apical margin of clypeus: with small median point. Labrum: not visible. Mandibular teeth: apex with 2, acute, subequal teeth. Arrangement of mandibular teeth: transverse. Number of maxillary palpomeres: 4. Shape of maxillary palpomeres: cylindrical. Number of labial palpomeres: 2. Number of antennomeres in female: 12.

Number of antennomeres in male: 12. Insertion of radicle into A1: parallel to longitudinal axis of A1. Shape of A1: more or less cylindrical, not flattened. Length of A3 of female: subequal to length of A2; distinctly longer than A2. Number of clavomeres in female antenna: 7; 0. Claval formula of female antenna: A12-A7/1-2-2-2-2-1; A12-A6/1-2-2-2-2-2-2. Arrangement of doubled multiporous plate sensilla on female clava: in longitudinal pairs. Tyloid distribution on male antenna: A5 only. Shape of male flagellum: filiform.

Mesosoma shape in dorsal view: longer than wide. Mesosoma shape in lateral view: longer than high. Medial portion of transverse pronotal carina: weakly indicated laterally; absent. Posterior apex of pronotum in dorsal view: straight, bifid apically to articulate with tegula. Vertical epomial carina: present. Dorsal epomial carina (lateral portion of transverse pronotal carina of Vilhelmsen et al. 2010): present. Anterior face of pronotum: oblique, visible dorsally, short. Lateral face of pronotum: deeply concave below dorsal pronotal superhumeral sulcus. Netrion: present. Netrion shape: open ventrally. Anterior portion of mesoscutum: vertical, flexed ventrally to meet pronotum. Mesoscutum shape: pentagonal in dorsal view, posterolateral corner rounded. Skaphion: absent. Notaulus: present, percurrent. Parapsidal lines: present; absent. Anteroadmedial lines: present. Scutoscutellar sulcus: well-developed, narrow. Shape of mesoscutellum: quadrate to trapezoidal. Armature of mesoscutellum: absent. Surface of mesoscutellum: convex throughout. Median longitudinal furrow on mesoscutellum: absent. Shape of axillula: small, dorsal margin sinuate. Metascutellum shape: clearly differentiated. Metascutellar armature: produced medially into short, shallowly bidentate process; produced into broad flattened plate; produced into narrow, flat, apically blunt process. Metascutellar setation: absent; present dorsally and ventrally. Extent of metasomal depression of propodeum: percurrent, extending anteriorly to anterior margin of propodeum. Lateral propodeal projection: well-developed, extending clearly beyond anterior margin of T1. Mesopleural carina: present across sclerite; absent or strongly abbreviated, present only near mid coxa. Mesal course of acetabular carina: projecting as small spur anteriorly, not long enough to intercede between fore coxae. Mesopleural pit: absent. Sternaulus: absent. Posterodorsal corner of mesopleuron: rounded anteriorly.

Number of mid tibial spurs: 1. Number of hind tibial spurs: 1. Dorsal surface of hind coxa: smooth. Hind tibia shape: cylindrical, ecarinate. Trochantellus: present.

Wing size of female: macropterous. Wing size of male: macropterous. Tubular veins in fore wing: present. Bulla of fore wing R: absent. Extent of marginal venation of fore wing: R1 reaching and ending at costal margin; distinct marginal or postmarginal veins present. Origin of r-rs in fore wing: arising before (basad of) R/R1 attains costal margin. Structure of basal vein (Rs+M) in fore wing: spectral. Structure of R in hind wing: elongate, extending to costal margin; abbreviated, not attaining costal margin.

Number of externally visible terga in female: 6. Number of externally visible sterna in female: 6. Number of externally visible terga in male: 8. Number of externally visible sterna in male: 7. Shape of metasoma: acuminate, widest submedially. Laterotergites: present, narrow. Laterosternites: present. T1 of female: raised medially into low, rectangular or subelliptical platform, laterally depressed. Relative size of metasomal segments: T2 distinctly largest; T2 and T3 distinctly larger, subequal in size. Terga with basal crenulae: T1, T2. Sublateral carinae on tergites: present on T1. Median longitudinal carina on metasomal terga: absent. Shape of female T6: flattened. Shape of posterior margin of male T7: straight; incised medially. Anterior margin of S1: protruding anteriorly as short sharp extension of median longitudinal carina. Felt fields: absent. Ovipositor type: *Scelio*-type (Austin and Field 1997).

#### Comments.

The genus is highly diverse and comprises in excess of 200 species worldwide. It has been recorded from Africa, the Indo-Malayan and eastern Palearctic regions (Burks et al. 2013). Following this study, 80 species are known from Australasia and the southwest Pacific. The genus is found broadly across mainland Australia and Tasmania but not New Zealand. In the southwest Pacific it has been recorded from Papua New Guinea, New Britain, Vanuatu, Fiji, New Caledonia and Lord Howe Island.

*Oxyscelio* has been collected from a large range of habitats from rainforest, open dry forest, grasslands to more open, dry environments including the malle, semi-arid and arid zones of Australia. Species have been collected using a variety of standard collecting techniques for small parasitic Hymenoptera, but they can be particularly numerous in yellow pan traps, even in closed habitats, indicating that many species may be living close to the ground.

#### Key to Australian and Oceanic species of *Oxyscelio*

**Table:** 

1	Male (female unknown): T7 with elongate and flat subparallel apical projections, but metasomal flanges absent from other terga; mesoscutal midlobe anteriorly granulate and without median carina but with very strong notauli (Fig. 126)	*Oxyscelio grandis* (Dodd)
–	Female, or if T7 with such apical projections (Figs 182, 224), then either mesoscutal midlobe with median carina, mesosoma very tall and steep anteriorly (Figs 177, 219), or notauli absent	2
2	Gena in lateral view longer than eye (Fig. 307)	*Oxyscelio sciuri* Burks, sp. n.
–	Gena in lateral view shorter than eye (Figs 177, 331)	3
3	T2 and T3 in females with sublateral depressions present, medially defined by sharp carina (Fig. 338). Males T1 midlobe carinae obscured, T7 apex concave but with weakly rounded lateral lobes that are not broad (Fig. 340)	*Oxyscelio solitarius* (Dodd)
–	T2 and T3 in females without sublateral depressions. T1 midlobe in males with distinct carinae or T7 apex either truncate (Fig. 7), with elongate spines (Fig. 182), or with broad lobes (Fig. 62)	4
4	Metasomal depression setose (Fig. 150)	*Oxyscelio lenitatis* Burks, sp. n.
–	Metasomal depression without setae (Fig. 334)	5
5	Metascutellum with a median carina that ends in a granulate area posteriorly (Fig. 348). New Guinea	*Oxyscelio spatulae* Burks, sp. n.
–	Metascutellum if with median carina (rare), then without posterior granulate area (Fig. 38)	6
6	Metascutellum convex, lateral rims directed ventrally and not visible in dorsal view (Figs 9, 68, 200), rarely very narrow and resting in groove formed by propodeum (Fig. 170)	7
–	Metascutellum concave, with raised lateral rims (Figs 58, 102)	10
7	Metascutellum much longer than broad, fitting in groove formed by lateral pronotal areas (Fig. 170)	*Oxyscelio linguae* Burks, sp. n.
–	Metascutellum as broad as or broader than long, not in contact with propodeum (Figs 9, 68, 200)	8
8	Face without oblique carina between eye and frontal depression (Fig. 69)	*Oxyscelio corrugationis* Burks, sp. n.
–	Face with oblique carina between eye and frontal depression (Figs 10, 201)	9
9	Mesoscutellum with granulate sculpture (Fig. 9). T1 midlobe with 6 separate longitudinal carinae despite presence of anterior T1 horn (Fig. 9). Submedian carina indicated by a single carina (Fig. 10). New Guinea	*Oxyscelio anfractus* Burks, sp. n.
–	Mesoscutellum without granulate sculpture (Fig. 200). T1 midlobe carinae obscured, indistinct anteriorly (Fig. 202). Submedian carina indicated by a set of weak rugae (Fig. 201)	*Oxyscelio mystacis* Burks, sp. n.
10	Interantennal process elongate (Fig. 203). New Guinea, New Britain	*Oxyscelio nasi* Burks, sp. n.
–	Interantennal process not elongate (Fig. 11)	11
11	Hyperoccipital carina indicated by a sharp carina (Figs 103, 284) and T1 lateral carina not expanded (Fig. 285) andoccipital carina absent medially (Figs 72, 103, 284). Postmarginal vein present and large (Fig. 246): *Oxyscelio flavipes* group (part)	12
–	Hyperoccipital carina incomplete (Fig. 12), indicated by rounded rugae (Fig. 178), or indicated by a set of 3 or more carinae (Figs 38, 370). If hyperoccipital carina sharp, then T1 with expanded lateral carina (Fig. 326). Postmarginal vein variable	19
12	Mesoscutellum with oblique elevated sculpture in female (Fig. 226), extensive granulate sculpture in male	*Oxyscelio obliquiatis* Burks, sp. n.
–	Mesoscutellum in female without oblique elevated sculpture (Fig. 102), without granulate sculpture in male	13
13	Mesoscutal midlobe with median carina (Fig. 284). Female: fore wing long enough to reach beyond metasomal apex. Male: T1 midlobe with 4 longitudinal carinae anteriorly (Fig. 288)	*Oxyscelio rugulosus* (Dodd)
–	Mesoscutal midlobe without a median carina (Fig. 103). If with a weak, indistinct one present (Fig. 320), then in female fore wing not reaching beyond metasomal apex. Male: T1 midlobe with 3 or 4 longitudinal carinae anteriorly (Fig. 106)	14
14	Lower face crossed by many curved rugae extending from eye across frontal depression (Fig. 111), weaker in female (Fig. 109). Submedian carina indicated by set of weak rugae (Figs 109, 111)	*Oxyscelio fluctuum* Burks, sp. n.
–	Lower face not entirely crossed by curved rugae, any transverse rugae present are restricted to frontal depression. Submedian carina variable, but often indicated by a single elevation (Fig. 73)	15
15	Metascutellum with only a small median concave area, laterally with broad longitudinally striate areas (Figs 72, 242)	16
–	Metascutellum with broad median concave area, laterally with narrow or no longitudinally striate areas (Figs 103, 320)	17
16	Female: occiput completely smooth (Fig. 241); mesoscutellum with setiferous puncta (Fig. 242). Male: mesoscutellum predominantly longitudinally rugose, but without any strong median carina and without foveate sculpture (Fig. 246)	*Oxyscelio oculi* Burks, sp. n.
–	Female: occiput with elevated sculpture (Fig. 72); mesoscutellum densely foveate (Fig. 72). Male: mesoscutellum with a median carina or ruga, otherwise densely foveolate to punctate, without other longitudinal rugae (Fig. 76)	*Oxyscelio croci* Burks, sp. n.
17.	Occiput strongly sculptured, with transverse rugae (Fig. 320). Female: T1 midlobe with raised smooth area obscuring longitudinal carinae anteriorly (Fig. 321)	*Oxyscelio sepisessor* Burks, sp. n.
–	Occiput weakly sculptured, almost entirely smooth (Fig. 103). Female: T1 midlobe without raised smooth area, all longitudinal carinae complete anteriorly (Fig. 104)	8
18	Metascutellum with median carina (Fig. 20). New Caledonia	*Oxyscelio bicoloripedis* Burks, sp. n.
–	Metascutellum without median carina (Fig. 103). Australia	*Oxyscelio flavipes* (Kieffer)
19	T1 lateral carina expanded and laterally lobe-like (Fig. 13), or absent with the area obscured by dense sculpture (Fig. 168). Metasomal flanges present on T5 and/or T6 in male and female of species with an obscured lateral T1 carina (Fig. 168). T7 deeply concave apically in some males (Fig. 62)	20
–	T1 lateral carina simple, not expanded, carina-like (Figs 3, 5). Metasomal flanges absent (Fig. 5). T7 apically truncate or very weakly emarginate in male (Fig. 7)	72
20	T6 in female [for male, see couplet 50] with subapical spine-like (cornicle-shaped) dorsally projecting spines (Fig. 180)	*Oxyscelio livens* Burks, sp. n.
–	T6 in female either lacking spines or with flat, apical spines (Fig. 222)	21
21	Hyperoccipital carina indicated by a strong carina; occiput almost entirely smooth (Figs 226, 326)	22
–	If hyperoccipital carina strong, then occiput densely sculptured (Fig. 86)	23
22	Female: mesoscutellum with strong longitudinal carinae and/or rugae (Fig. 326). Male: mesoscutellum with strong rugose sculpture (Fig. 330)	*Oxyscelio shakespearei* (Girault)
–	Female: mesoscutellum with oblique raised sculpture (Fig. 226). Male: mesoscutellum granulate, weakly sculptured (Fig. 230)	*Oxyscelio obliquiatis* Burks, sp. n.
23	Female: Main body of T6 forming a complete ledge above apical rim, hiding it from dorsal view, instead usually concave and revealing apex of S6 to dorsal view (Figs 190, 368). Male: T5-T7 with metasomal flanges (Fig. 192): *Oxyscelio mirellus* species group (part)	24
–	Female: Main body of T6 either not forming ledge, or with ledge interrupted medially and descending to a clearly visible apical rim, not revealing S6 to dorsal view (Fig. 46). Male: T5, T6 of most species without metasomal flanges (Fig. 132), but with sharp corners in some species in which T7 also has elongate, narrow metasomal flanges (Fig. 282)	35
24	Female: T6 with complete peripheral ledge formed by thin cuticle (Fig. 210). Male: All flagellomeres between A3 and A12 broader than long (Fig. 211) andcoxae same color as rest of leg andT6 with only tiny metasomal flanges (Fig. 212)	*Oxyscelio nigriclava* (Dodd)
–	Female: T6 with lateral metasomal flanges or a posterior metasomal flange that is interrupted medially (Fig. 190). Male: if flagellomeres transverse then coxae dark or T6 metasomal flanges elongate (Fig. 192)	25
25	Mesoscutal midlobe with transverse carinae (Fig. 114). Metasomal flanges flat and elongate, acuminate, with irregular edges (Figs 116, 118)	*Oxyscelio foliorum* Burks, sp. n.
–	Mesoscutal midlobe without transverse carinae (Fig. 188). Metasomal flanges not as elongate (Figs 164, 192)	26
26	Frontal depression with transverse carinae present in dorsal portion (Fig. 121), although these carinae may be medially interrupted (Fig. 395); submedian carina weakly developed or absent (Figs 121, 395). Coxa always darker than rest of leg (Fig. 119)	27
–	Frontal depression without transverse carinae in dorsal portion; submedian carina strong, sharp (Fig. 161). Coxa color variable	32
27	Female: A4 longer than broad, A5 as long as or longer than broad (Fig. 121). Male: T6 metasomal flanges with strong corners and hardly projecting (Fig. 124)	*Oxyscelio funis* Burks, sp. n.
–	Female: A4 not longer than broad, A5 broader than long. Male: T6 metasomal flanges (when male known) with rounded projecting lobes (Fig. 148)	28
28	Mesoscutellar rim without median notch (Fig. 366)	29
–	Mesoscutellar rim with median notch (Fig. 144)	30
29	Metascutellum tiny (Fig. 394). Apex of S6 exposed to dorsal view (Fig. 396)	*Oxyscelio velamenti* Burks, sp. n.
–	Metascutellum broad (Fig. 365). S6 hidden from dorsal view by large apical rim of T6 (Fig. 368)	*Oxyscelio truncationis* Burks, sp. n.
30	Female: T5 without metasomal flanges, T6 weakly or not at all emarginate apically (Fig. 354)	*Oxyscelio stipulae* Burks, sp. n.
–	Female: T5 with metasomal flanges, T6 deeply emarginate apically (Figs 18, 146)	31
31	Mesoscutal midlobe with strong, coarse foveate sculpture, without smooth areas (Fig. 144)	*Oxyscelio incisurae* Burks, sp. n.
–	Mesoscutal midlobe with weaker sculpture and with smooth area near midlength (Fig. 16)	*Oxyscelio bellariorum* Burks, sp. n.
32	Female: T6 deeply emarginate but medially truncate (Fig. 190). Male: A11 broader than long (Fig. 191). Coxa darker than rest of leg (Fig. 187)	*Oxyscelio mirellus* (Dodd)
–	Female: T6 V-shaped or rounded medially if deeply emarginate (Figs 162, 168, 304). Male: A11 longer than broad (Figs 163, 305). Coxa usually (2 out of 3 species) same color as rest of leg (Fig. 159)	33
33	Mesoscutellar rim expanded (Fig. 160). Female: T6 deeply emarginate, angular medially (Fig. 162). Male: T5 with metasomal flanges (Fig. 164)	*Oxyscelio limbi* Burks, sp. n.
–	Mesoscutellar rim not expanded (Fig. 166). If rim weakly sculptured (Fig. 302) then T6 weakly or not at all emarginate in female (Figs 168, 304). Male T5 phenotype unknown	34
34	Coxa darker than rest of leg (Fig. 301). Female: T5 with rounded metasomal flanges (Fig. 304)	*Oxyscelio scismatis* Burks, sp. n.
–	Coxa same color as rest of leg (Fig. 165). Female: T5 with sharp metasomal flanges (Fig. 168)	*Oxyscelio liminis* Burks, sp. n.
35	T6 abruptly narrower than T5 (sometimes T5 also abruptly narrower than T4), these with strong, straight posterolateral corners (Fig. 280). T6 in female with narrow and elongate posterior metasomal flanges or sharp posterolateral corners (Fig. 280) or T5 with subapical protrusion (Fig. 378). Males with strong narrow T7 metasomal flanges (Fig. 282): *Oxyscelio rami* species group	36
–	If apical terga abruptly narrower than preceding terga, then some of them other than apical tergum with metasomal flanges (Fig. 380). T7 emarginate but without metasomal flanges (Fig. 294)	39
36	T5 with subapical rounded protrusions (Figs 378, 380)	*Oxyscelio umbonis* Burks, sp. n.
–	T5 without subapical protrusions (Figs 36, 66, 280)	37
37	Apical tergum medially blunt or roundly emarginate, with elongate narrow metasomal flanges (Figs 36, 280)	38
–	Apical tergum medially angular, with sharp posterior corners that are not elongate (Fig. 66)	*Oxyscelio contusionis* Burks, sp. n.
38	Submedian carina distinct (Fig. 279)	*Oxyscelio rami* Burks, sp. n.
–	Submedian carina absent medially (Fig. 35)	*Oxyscelio caudarum* Burks, sp. n.
39	Female: T6 with lateral ledges that medially slope down to the apical rim (Fig. 46), or T6 with subapical metasomal flanges (Figs 80) or a broad shelf (Fig. 368). Male: Fore wing not long enough to reach beyond metasomal apex andT7 deeply emarginate apically but without posterior spines (Fig. 132)	40
–	Female: T6 without lateral ledges, without metasomal flanges (Fig. 140). Male: Either fore wing exceeding metasomal apex orT7 truncate or hardly emarginate apically (Fig. 142) or T7 with narrow, spine-like posterior projections (Fig. 182)	48
40	Female: T6 with tiny sharp subapical metasomal flanges, tergum abruptly narrowed posterior to them (Fig. 80). Male: A11 broader than long (Fig. 131) and T7 deeply emarginate (Fig. 132)	41
–	Female: T6 with lateral margins forming shelf-like areas (Fig. 215, 368). Male: A11 longer than broad (Fig. 293)	42
41	Coxa darker than rest of leg (Fig. 77). Female: A3 longer than pedicel, T1 midlobe with 6 longitudinal carinae (Fig. 80). Males unknown	*Oxyscelio cuspidis* Burks, sp. n.
–	Coxa same color as most of femur (Fig. 127). Female: A3 not longer than pedicel, T1 midlobe with 5 longitudinal carinae. Males with A4 and A11 broader than long (Fig. 131)	*Oxyscelio gressus* Burks, sp. n.
42	Female: T6 apex with strongly protruding posterior rim, main body of tergum anterior to it forming a narrowed shelf laterally, median carina extending from main body of tergum to apical rim; all this together forming an almost trident-shaped apex (Figs 46, 50). Submedian carina sharp (Figs 45, 49)	43
–	Female: T6 with hardly protruding posterior rim, main body of tergum forming broad shelf laterally that is partially raised above apical rim and does not have a median carina; all this together forming an essentially truncate apex (Figs 215, 368). Submedian carina weak or absent (Figs 216, 367)	44
43	Female: T4 with broad, flat metasomal flanges (Fig. 50)	*Oxyscelio clupei* Burks, sp. n.
–	Female: T4 without metasomal flanges, or with very tiny and sharply pointed metasomal flanges (Fig. 46)	*Oxyscelio clivi* Burks, sp. n.
44	Frontal depression without carinae in dorsal portion (Figs 216, 367)	45
–	Frontal depression with some carinae in dorsal portion, which may be interrupted medially (Fig. 99, 353)	46
45	Female (males unknown): Fore wings not long enough to extend beyond T5 (Fig. 368)	*Oxyscelio truncationis* Burks, sp. n.
–	Female: Fore wings extending beyond T5 (Fig. 215). Male: A11 broader than long (Fig. 217); T7 tiny, not deeply emarginate apically (Fig. 218)	*Oxyscelio nigricoxa* (Dodd)
46	Female: A4 broader than long. [Males unknown]	47
–	Female: A4 longer than broad. [Male: (Figs 293, 294)]	*Oxyscelio rupturae* Burks, sp. n.
47	Female: T6 only shallowly emarginate medially, not sloping to apical rim (Fig. 354)	*Oxyscelio stipulae* Burks, sp. n.
–	Female: T6 deeply emarginate medially, with a median slope leading down to apical rim (Fig. 100)	*Oxyscelio exiguitatis* Burks, sp. n.
48	Males only: T7 with long posterior spines (Fig. 182)	49
–	Male or female: apical tergum without spines (Fig. 142)	51
49	A4 longer than broad (Fig. 223). Body without metallic color (Fig. 220), legs yellowish	*Oxyscelio nitoris* Burks, sp. n.
–	A4 broader than long (Figs 181). Either body with some metallic color (Fig. 178), or entirely dark brown including legs (Fig. 381)	50
50	Head and mesosoma weakly metallic blue or green (Fig. 178). Legs yellowish (Fig. 177)	*Oxyscelio livens* Burks, sp. n.
–	Entire body non-metallic dark brown, including legs (Fig. 381)	*Oxyscelio uncinorum* Burks, sp. n.
51	Frontal depression deep and nearly parallel-sided, with many transverse carinae above and below dorsal separator, but submedian carina weak or absent medially (Fig. 59). Female: Fore wing long enough to reach middle of T4 or T5; T6 apically steep and not emarginate (Fig. 60). Male: Fore wing long enough to reach middle of T5, T7 broadly emarginate (Fig. 62), A11 longer than broad (Fig. 61)	*Oxyscelio conjuncti* Burks, sp. n.
–	Frontal depression, if with many carinae, then strongly broadening ventrally and not deep (Fig. 135). Other features variable, but rarely as above	52
52	Tibiae with spines (Figs 341)	53
–	Tibiae without spines	54
53	Frontal depression very broad, with strong submedian carina (Fig. 343)	*Oxyscelio sordes* Burks, sp. n.
–	Frontal depression not very broad, submedian carina weak or absent (Figs 135)	*Oxyscelio hamorum* Burks, sp. n.
54	Mesoscutal midlobe posteriorly with many strong longitudinal rugae	*Oxyscelio obturationis* Burks, sp. n.
–	Mesoscutal midlobe posteriorly with few or no longitudinal rugae	55
55	Hyperoccipital carina indicated by sharp carina (Fig. 314). Female: submedian carina indicated by a set of weak rugae or carinae (Fig. 315). Mesoscutellum with elevated sculpture, including weakly indicated oblique elevations (Fig. 314)	*Oxyscelio scutorum* Burks, sp. n.
–	If hyperoccipital carina sharp and submedian carina accompanied by additional carinae, then mesoscutellum without elevated sculpture (Fig. 86)	56
56	Frontal depression with carinae or transverse elevations, which may be medially interrupted, above dorsal separator (Figs 14, 83)	57
–	Frontal depression smooth dorsally (Fig. 25)	61
57	Coxa darker than rest of leg (Fig. 11), or entire body dark brown including legs	58
–	Coxa not darker than rest of leg, apical part of legs yellowish (Fig. 193)	*Oxyscelio montanus* (Dodd)
58	Female only (male cannot be reliably keyed past this point): A4 longer than broad	59
–	Female: A4 broader than long	60
59	Submedian carina complete medially as a distinct carina (Fig. 14)	*Oxyscelio atricoxa* (Dodd)
–	Submedian carina absent medially (Fig. 83)	*Oxyscelio densitatis* Burks, sp. n.
60	Postmarginal vein present, venation reaching or nearly reaching anterior wing margin (Fig. 410)	*Oxyscelio wa* Burks, sp. n.
–	Postmarginal vein absent, venation not closely approaching anterior wing margin (Fig. 298)	*Oxyscelio sarcinae* Burks, sp. n.
61	Entire body dark brown, including antennae and legs	62
–	Some part(s) of legs and antennae yellowish	64
62	Occipital carina with flat transverse sections connected median arch to lateral areas (Fig. 382). Male: T7 with apical spines (Fig. 386)	*Oxyscelio uncinorum* Burks, sp. n.
–	Occipital carina nearly uniformly arched, without flat transverse sublateral sections. (Figs 258, 360). Male: T7 without spines (Figs 262, 364)	63
63	Female: T1 midlobe longitudinal carinae obscured by smooth area anteriorly. Male: most flagellomeres between A4 and A11 broader than long, T7 blunt apically and without lobes	*Oxyscelio pollicis* Burks, sp. n.
–	Female: T1 midlobe with 4 distinct longitudinal carinae. Male: all flagellomeres longer than broad, T7 emarginate apically and with rounded posterior lobes	*Oxyscelio tenuitatis* Burks, sp. n.
64	Coxa darker than rest of leg (Figs 81, 183, 213)	65
–	Coxa not darker than rest of leg (Fig. 137)	67
65	Female: fore wing not long enough to exceed metasomal apex; A4 longer than broad; T6 not concave apically (Fig. 84)	*Oxyscelio densitatis* Burks, sp. n.
–	Female: fore wing long enough to reach or exceed metasomal apex, A4 as broad or broader than long; T6 at least mildly concave apically (Figs 186, 215)	66
66	Female: mesoscutellum very sparsely foveate, with broad smooth areas between foveae (Fig. 184); T6 only slightly concave apically (Fig. 186); A3 not longer than pedicel	*Oxyscelio magniclava* (Dodd)
–	Female: mesoscutellum densely foveate (Fig. 214); T6 distinctly concave apically (Fig. 215); A3 longer than pedicel	*Oxyscelio nigricoxa* (Dodd)
67	Occipital carina omicron-shaped, with narrow and strongly convex median section connected to lateral sections by a transverse carina (Fig. 220)	68
–	Occipital carina broadly rounded, with no differentiation medially (Fig. 138)	69
68	Metanotum and posterior rim of mesoscutellum pale, in contrast with surrounding areas (Fig. 248). Female: A4 longer than broad. Male: T7 without posterior spines (Fig. 252)	*Oxyscelio palati* Burks, sp. n.
–	Metanotum and posterior rim of mesoscutellum not pale (Fig. 220). Female: A4 broader than long. Male: T7 with posterior spines (Fig. 224)	*Oxyscelio nitoris* Burks, sp. n.
69	Occipital carina absent medially	*Oxyscelio dissimulationis* Burks, sp. n.
–	Occipital carina present medially	70
70	Submedian carina weak and indicated by rounded ruga, frontal depression shallow (Fig. 139). Postmarginal vein present but very short	*Oxyscelio hyalinipennis* (Dodd)
–	Submedian carina sharp and strong, and frontal depression deep (Figs 25, 195). Postmarginal vein absent (as in Fig. 298)	71
71	Dorsal portion of frontal depression without transverse carinae (Fig. 25). Female: A3 shorter than pedicel (Fig. 25)	*Oxyscelio brevitas* Burks, sp. n.
–	Female: Dorsal portion of frontal depression with interrupted transverse carinae (Fig. 195). A3 longer than pedicel	*Oxyscelio montanus* (Dodd)
72	Hyperoccipital carina and submedian carina represented by several (more than 3) sharp and distinct rugae (Figs 38, 39, 370, 371). Legs, including coxae, bicolored (Figs 37, 369). New Caledonia	73
–	Hyperoccipital carina and submedian carina not both represented by so many rugae (Figs 3, 4). Leg color variable, usually not bicolored	74
73	Female: Fore wing long enough to reach middle of T5; T1 with very strong anterior horn; T6 longer than broad (Fig. 372). Male: fore wing long enough to reach apex of T5	*Oxyscelio tubi* Burks, sp. n.
–	Female: Fore wing long enough to reach beyond metasomal apex; T1 with very weak anterior swelling; T6 broader than long (Fig. 40). Male: fore wing long enough to reach beyond metasomal apex	*Oxyscelio circulorum* Burks, sp. n.
74	Metasomal sterna entirely smooth aside from setiferous puncta (Fig. 157): *Oxyscelio leviventris* species group	75
–	Metasomal sterna with some longitudinal rugae or carinae (Fig. 136)	76
75	Clypeus elongate (Fig. 233). Metascutellum setose dorsally (Fig. 232)	*Oxyscelio oblongiclypei* Burks, sp. n.
–	Clypeus not elongate (Fig. 155). Metascutellum not setose (Fig. 154)	*Oxyscelio leviventris* Burks, sp. n.
76	Epomial corner projecting laterally (Figs 28, 388). Genal carina expanded in ventral half, with long foveae separating it from gena (Figs 27, 387): *Oxyscelio catenae* species group	77
–	Epomial corner not projecting laterally (Fig. 3). Genal carina not expanded (Fig. 2)	78
77	Submedian carina present, indicated by rounded ruga (Fig. 29). Female: mesoscutellum with smooth area centrally (Fig. 28); fore wing long enough to reach middle of T5. Male: Fore wing long enough to reach middle of T6	*Oxyscelio catenae* Burks, sp. n.
–	Submedian carina absent, the area with some convergent heart-shaped sculpture (Fig. 389). Female: mesoscutellum densely sculptured centrally (Fig. 388); fore wing long enough to reach apex of T5 or middle of T6. Male: Fore wing long enough to reach beyond metasomal apex	*Oxyscelio valdecatenae* Burks, sp. n.
78	Mesoscutum and mesoscutellum with sunken median longitudinal depression where median carinae would otherwise be; areas lateral to these depressions strongly raised (Fig. 398)	*Oxyscelio verrucae* Burks, sp. n.
–	Mesoscutum and mesoscutellum without longitudinal depression, without raised submedian areas (Fig. 3)	79
79	Submedian carina and hyperoccipital carina absent (Fig. 406). Mesosoma yellow, with only tiny setiferous puncta dorsally (Fig. 405). Vanuatu, Fiji	*Oxyscelio viator* Burks, sp. n.
–	Submedian carina and/or hyperoccipital carina indicated by carinae or rugae (Fig. 4). Mesosomal color variable, but with more sculpture than just setiferous puncta if yellow (Fig. 3)	80
80	Mesoscutellum, posterior portion of mesoscutal midlobe with many strong longitudinal rugae (Fig. 254)	*Oxyscelio pectinis* Burks, sp. n.
–	Mesoscutellum especially, and usually mesoscutal midlobe, without longitudinal rugae (Fig. 3)	81
81	Metascutellum broad and convex, rugose centrally (Fig. 264)	*Oxyscelio proceritatis* Burks, sp. n.
–	Metascutellum about as long as broad, concave dorsally, without rugae centrally. (Fig. 278)	82
82	Postmarginal vein absent or extremely short. Entire body, including antennae and legs, dark brown (Fig. 51)	*Oxyscelio concoloripes* (Dodd)
–	Postmarginal vein long, thick. At least part of legs yellowish or reddish (Fig. 2)	83
83	Hyperoccipital carina sharp and strongly developed (Fig. 320). Frontal depression without transverse carinae (Fig. 322). Occipital carina incomplete medially (Fig. 320; can be difficult to assess due to rough sculpture)	*Oxyscelio sepisessor* Burks, sp. n.
–	Either: hyperoccipital carina irregular or weakly developed, or: frontal depression with transverse carinae that may be interrupted medially (Fig. 4) and occipital carina complete medially (Fig. 3)	84
84	Metasomal depression roughly sculptured, with anterior carinae or submedian foveae (Figs 334, 357). Frontal depression deep, without transverse carinae or with very short and broadly interrupted carinae, without median carina (Fig. 333)	85
–	Metasomal depression smooth anteriorly (Fig. 94), sometimes hidden by T1 horn. Frontal depression variable	86
85	T1 midlobe with 4 longitudinal carinae	*Oxyscelio stringerae* Burks, sp. n.
–	T1 midlobe with 5 longitudinal carinae	*Oxyscelio sinuationis* Burks, sp. n.
86	Occiput concave dorsally, outlined by carinae because of weak connection between hyperoccipital carina and occipital carina (Figs 3, 92, 272)	87
–	Occiput convex dorsally, with no connection between occipital carina and hyperoccipital carina (Figs 174, 268)	89
87	Median carina of frontal depression reaching submedian carina (Fig. 93). Mesoscutellum smooth, with only some tiny setiferous puncta; metascutellum tiny, very narrow (Fig. 92). Female: T1 midlobe with 5 distinct longitudinal carinae (Fig. 92). Male: T1 midlobe with 4 longitudinal carinae (Fig. 96)	*Oxyscelio divisionis* Burks, sp. n.
–	Median carina of frontal depression not reaching submedian carina (Figs 4, 273). Mesoscutellum densely foveate; metascutellum moderately broad, superficially rugose (Figs 3, 272). Female: T1 midlobe with anterior swelling obscuring longitudinal carinae (Figs 3, 272). Male: T1 midlobe with 3 longitudinal carinae (Figs 7, 276)	88
88	Female: T1 horn weakly developed, not nearly reaching metascutellum (Figs 3, 5); fore wing long enough to reach middle of T6; T6 broader than long (Fig. 5). Male: fore wing long enough to exceed metasomal apex; metasoma moderately broad (Fig. 7)	*Oxyscelio aciculae* Burks, sp. n.
–	Female: T1 horn strongly developed, nearly reaching metascutellum (Fig. 272); fore wing long enough to reach middle of T5 or base of T6; T6 longer than broad (Fig. 274. Male: fore wing not long enough to exceed metasomal apex; metasoma very narrow (Fig. 276)	*Oxyscelio radii* Burks, sp. n.
89	Metascutellum medially with a branched ruga (Fig. 174). Lord Howe Island	*Oxyscelio lintris* Burks, sp. n.
–	Metascutellum medially with 3 longitudinal carinae between the lateral margins (Fig. 268). Queensland	*Oxyscelio productionis* Burks, sp. n.

##### Species groups of Australasian and Oceanic *Oxyscelio*

These groups are provided here to indicate intuitively perceived structure within the genus, and to provide an aid for identification. They are characterized in the above key and are diagnosed here in a succinct way. Some characters are omitted from certain species group diagnoses because those characters are variable within the group or are otherwise unhelpful for that particular group’s identification. Individual species descriptions can be consulted regarding characters omitted from these diagnoses.

##### *Oxyscelio aciculae* species group

*Characteristics*: Hyperoccipital carina absent or represented by weak rugae. Occipital carina complete or incomplete. Metascutellum nearly flat, subrectangular. Postmarginal vein present, strong. T1 lateral carina not expanded. Metasomal flanges absent. Main body of T6 in females not forming ledge above apical rim. T7 in males small, not or only weakly emarginate.

*Comments*: The *aciculae*-group contains long-bodied species with a narrower and flatter metascutellum than similarly-shaped species from Asia. An anterior T1 horn may be present or not. Metasomal depression sculpture, frontal depression depth and sculpture, and the presence of an outlined dorsal area of the occiput can be used to further subdivide these species. Some species in Asia and Africa also have an outlined, dorsally concave area of the occiput, but do not strongly resemble any species in this group in features of the face and metascutellum.

This group resembles the and *catenae*-group, *concoloripes*-group, and *proceritatis*-group in general habitus, but lack distinctive features present in those groups. The *aciculae*-group may be closely related to the *flavipes*-group, especially to species such as *Oxyscelio tubi*, but these groups are kept separate based on differences in the metascutellum.

*Included species*: *Oxyscelio aciculae*, *Oxyscelio divisionis*, *Oxyscelio lintris*, *Oxyscelio pectinis*, *Oxyscelio productionis*, *Oxyscelio radii*, *Oxyscelio sciuri*, *Oxyscelio sinuationis*, *Oxyscelio stringerae*.

##### *Oxyscelio atricoxa* species group

*Characteristics*: Hyperoccipital carina variable. Occipital carina complete medially. Metascutellum with a broad apical fovea and dorsally projecting posterior corners. Postmarginal vein absent or extremely short. T1 lateral carina expanded, visible from ventral view. Metasomal flanges present, or main body of T6 abruptly elevated above apical rim, in some species.

*Comments*: The *atricoxa*-group contains species that have either an expanded lateral T1 carina or a strongly sculptured area in its place (in which case distinct Metasomal flanges are present). While it is not convenient to lump distinctive species such as *Oxyscelio mirellus* with very generalized species such as *Oxyscelio atricoxa*, the fine gradient of variation between presence and absence of Metasomal flanges prevents any logical dividing line between these sets of species. No intuitive group containing *Oxyscelio atricoxa* is monophyletic in our analysis (Fig. 1), but we hypothesize that the presence of Metasomal flanges represents an apomorphic character. The *limae*-group from Sri Lanka and India resembles this group in body shape, and in that some members have apparent tiny Metasomal flanges, but they do not resemble the *atricoxa*-group in features of the metascutellum or T1.

*Included species*: *Oxyscelio atricoxa*, *Oxyscelio bellariorum*, *Oxyscelio brevitas*, *Oxyscelio caudarum*, *Oxyscelio clivi*, *Oxyscelio clupei*, *Oxyscelio conjuncti*, *Oxyscelio contusionis*, *Oxyscelio cuspidis*, *Oxyscelio densitatis*, *Oxyscelio exiguitatis*, *Oxyscelio foliorum*, *Oxyscelio funis*, *Oxyscelio gressus*, *Oxyscelio hamorum*, *Oxyscelio hyalinipennis*, *Oxyscelio incisurae*, *Oxyscelio limbi*, *Oxyscelio liminis*, *Oxyscelio livens*, *Oxyscelio magniclava*, *Oxyscelio mirellus*, *Oxyscelio montanus*, *Oxyscelio nigriclava*, *Oxyscelio nigricoxa*, *Oxyscelio nitoris*, *Oxyscelio obturationis*, *Oxyscelio palati*, *Oxyscelio pollicis*, *Oxyscelio rami*, *Oxyscelio rupturae*, *Oxyscelio sarcinae*, *Oxyscelio sarcinae*, *Oxyscelio scismatis*, *Oxyscelio scutorum*, *Oxyscelio sordes*, *Oxyscelio stipulae*, *Oxyscelio tenuitatis*, *Oxyscelio truncationis*, *Oxyscelio umbonis*, *Oxyscelio uncinorum*, *Oxyscelio velamenti*, *Oxyscelio wa*.

##### *Oxyscelio catenae* species group

*Characteristics*: Hyperoccipital carina indicated by rugae. Occipital carina complete, convex. Epomial corner sharp, protruding. Mesoscutum with raised longitudinal smooth area postero-medially. Postmarginal vein present. T1 lateral carina not expanded. Metasomal flanges absent. Main body of T6 in females not forming shelf above apical rim. T7 in males truncate or slightly emarginate apically, without protrusions.

*Comments*: The *catenae*-group encompasses two very similar Western Australian species that strongly differ from other Australian *Oxyscelio*. The distinctive mesoscutum and laterally protruding epomial corners aid in identification of this group.

*Included species*: *Oxyscelio catenae*, *Oxyscelio valdecatenae*.

##### *Oxyscelio concoloripes* species group

*Characteristics*: Hyperoccipital carina indicated by sharp rugae or carinae. Occipital carina variable. Metascutellum deeply concave, with broad posterior fovea. T1 lateral carina not expanded. Postmarginal vein absent or nearly so. Metasomal flanges absent. Main body of T6 not forming ledge above apical rim. T7 in males truncate or nearly so.

*Comments*: The *concoloripes*-group contains a pair of species that are somewhat similar to the *aciculae*-group and *flavipes*-group, but which do not have a simple hyperoccipital carina and do not have a strongly developed postmarginal vein. Both included species can have mostly or entirely dark brown bodies, but this feature is variable in *Oxyscelio verrucae* and is present in some other species that are not otherwise similar to these species.

*Included species*: *Oxyscelio concoloripes*, *Oxyscelio verrucae*.

##### *Oxyscelio dasymesos* species group

*Characteristics*: Hyperoccipital carina indicated by rugae. Occipital carina absent medially. Metascutellum concave dorsally. Postmarginal vein present. Metasomal depression setose. T1 lateral carina not expanded. Metasomal flanges absent. Main body of T6 in females not separated from apical rim. T7 in males weakly emarginate.

*Comments*: The otherwise Asian *dasymesos*-group has one known Australian species. This group is distinct in having a setose metasomal depression.

*Included species*: *Oxyscelio lenitatis*.

##### *Oxyscelio flavipes* species group

*Characteristics*: Hyperoccipital carina sharply indicated (exceptions: *Oxyscelio circulorum*, *Oxyscelio tubi*, *Oxyscelio viator*). Occipital carina absent medially, but without strong lateral corners. Metascutellum concave dorsally, with broad apical fovea. Postmarginal vein present, well-developed. T1 lateral carina not expanded (exception: some *Oxyscelio obliquiatis*). Metasomal flanges absent. Main body of T6 in females not separated from apical rim. T7 in males truncate or weakly emarginate, without projections.

*Comments*: The *flavipes*-group is a major group that is limited to Australia and the Pacific Islands. In having a strong hyperoccipital carina, it resembles the Asian *cuculli*-group, but differs from it in having an incomplete occipital carina in which the lateral branches closely approach the hyperoccipital carina dorsally. These two groups otherwise resemble each other in having a relatively fusiform metasoma. Some species in this group, especially *Oxyscelio tubi*, resemble some in the *aciculae*-group, but are placed here because of metascutellar features.

*Included species*: *Oxyscelio bicoloripedis*, *Oxyscelio circulorum*, *Oxyscelio croci*, *Oxyscelio flavipes*, *Oxyscelio fluctuum*, *Oxyscelio obliquiatis*, *Oxyscelio oblongiclypei*, *Oxyscelio oculi*, *Oxyscelio rugulosus*, *Oxyscelio sepisessor*, *Oxyscelio tubi*, *Oxyscelio viator*.

##### *Oxyscelio fossarum* species group

Characteristics:Hyperoccipital carina absent or represented by weak rugae. Occipital carina incomplete medially, with strong lateral corners. Metascutellum nearly flat. Postmarginal vein present. T1 lateral carina not expanded. Metasomal flanges absent. T2 with sublateral depressions, set off by carinae medially. Main body of T6 in females not separated from apical rim. T7 in males weakly emarginate apically.

*Comments*: The mostly Asian *fossarum*-group has one known Australian species. This group is very different from most other Australian *Oxyscelio*, only resembling some members of the *aciculae*-group and *proceritatis*-group.

*Included species*: *Oxyscelio solitarius*.

##### *Oxyscelio foveatus* species group

*Characteristics*: Hyperoccipital carina absent or represented by weak rugae. Occipital carina incomplete medially, with strong lateral corners. Lower face, between antennal insertion and eye, with oblique flange-like expansion. Metascutellum nearly flat. Postmarginal vein present. T1 lateral carina not expanded. Metasomal flanges absent. Main body of T6 in females not separated from apical rim.

*Comments*: Most known species of the *foveatus*-group occur in Asia. This group may not be monophyletic, being united mainly by the presence of the oblique facial flange and a long body, but splitting it would result in many small species groups that would ultimately be no better supported. The Philippine species *Oxyscelio cupularis* (Kieffer) is very similar to the three species discussed here.

*Included species*: *Oxyscelio anfractus*, *Oxyscelio linguae*, *Oxyscelio mystacis*.

##### *Oxyscelio proceritatis* species group

*Characteristics*: Hyperoccipital carina indicated by weak rugae. Occipital carina absent medially. Gena posteroventrally smooth and glossy. Lower face without oblique flange between antennal insertion and eye. Metascutellum very broad, rugose. Postmarginal vein present. T1 lateral carina not expanded. T2 without sublateral depressions. Metasomal flanges absent. Main body of T6 in females not separated from apical rim. T7 in males weakly emarginate apically.

*Comments*: These two species resemble the Asian *latitudinis*-group in having a broad, rugose metascutellum and a long body. They differ in having a weakly sculptured head, including the posteroventrally smooth gena. They are kept separate from the *latitudinis*-group because of these differences.

*Included species*: *Oxyscelio corrugationis*, *Oxyscelio proceritatis*.

##### Species not placed to group

*Included species*: *Oxyscelio dissimulationis*, *Oxyscelio grandis*, *Oxyscelio nasi*, *Oxyscelio shakespearei*, *Oxyscelio spatulae*.

*Comments*: *Oxyscelio dissimulationis* and *Oxyscelio shakespearei* possess features of both the *atricoxa*-group and *flavipes*-group, and are therefore problematic. *Oxyscelio nasi* is somewhat similar to the *flavipes*-group, but lacks all distinctive features of that group. *Oxyscelio spatulae* is similar to some species of the *foveatus*-group, but lacks oblique facial flanges.The holotype of *Oxyscelio grandis* is incomplete, and does not provide enough information to place it into a species group.

### Species descriptions

#### Oxyscelio aciculae

Burks sp. n.

http://zoobank.org/95680207-9162-40EB-8054-39AEF9294D81

urn:lsid:biosci.ohio-state.edu:osuc_concepts:307063

http://species-id.net/wiki/Oxyscelio_aciculae

**Figures 2–7.**
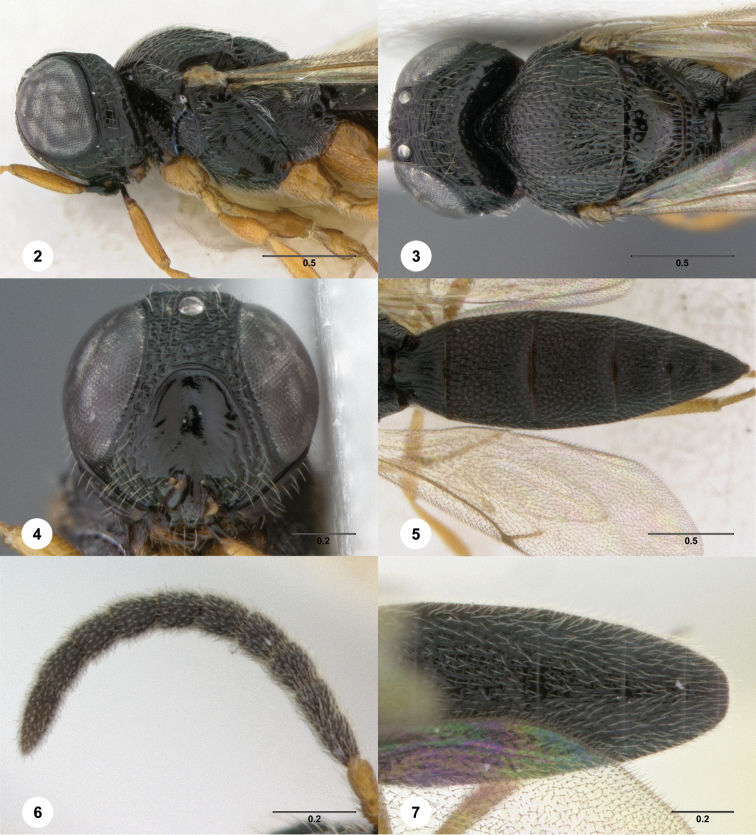
*Oxyscelio aciculae* sp. n., paratype female (OSUC 368139) **2** Head and mesosoma, lateral view. Paratype female (OSUC 368110) **3** Head and mesosoma, dorsal view **4** Head, anterior view **5** Metasoma, dorsal view. Paratype male (OSUC 368143) **6** Antenna **7** Metasoma, dorsal view. Morphbank^26^

Figures 2–7 ; Morphbank^26^

##### Description.

Female. Body length 3.1–4.15 mm (n=20).

Radicle color and shade: darker than scape. Pedicel color: same as scape. A3: shorter than pedicel. A4: broader than long. A5: broader than long.

Ventral clypeal margin: with slightly convex median lobe. Interantennal process: not elongate. Lower frons at dorsal margin of interantennal process: without transverse carina. Transverse curved rugae extending from frontal depression to eye: absent. Median longitudinal carina in frontal depression: absent. Ventral portion of frontal depression: with medially interrupted transverse carinae. Dorsal portion of frontal depression: without transverse carinae. Submedian carina: present. Frontal depression dorsally: not hood-like, open dorsally. Upper frons major sculpture: umbilicate foveate; transversely rugose. Upper frons microsculpture: absent. Hyperoccipital carina: indicated by a set of irregular elevations. Carina connecting occipital carina to hyperoccipital carina: present. Occipital carina: present laterally, absent medially. Occiput sculpture: irregularly sculptured. Extra carina ventral to occipital carina: absent. Gena length: shorter than eye. Major sculpture of gena anteroventrally: umbilicate foveate; rugose; umbilicate punctate. Major sculpture of gena posteroventrally: rugose; umbilicate punctate. Microsculpture of gena anteroventrally: absent. Microsculpture of gena posteroventrally: absent.

Lateral pronotal area sculpture: irregularly sculptured. Posterior border of central pronotal area: directed posteriorly, epomial carina absent or meeting transverse pronotal carina at arch on lateral surface of pronotum. Mesoscutum anteriorly: not steep, forming less than a right angle. Major sculpture of mesoscutal midlobe anteriorly: umbilicate foveate. Mesoscutal midlobe sculpture at midlength: not different from nearby sculpture. Major sculpture of mesoscutal midlobe posteriorly: umbilicate foveate; longitudinally rugose. Microsculpture of mesoscutal midlobe anteriorly: granulate. Microsculpture of mesoscutal midlobe posteriorly: absent. Median mesoscutal carina: present as a ruga. Major sculpture of mesoscutellum centrally: umbilicate foveate; umbilicate punctate. Major sculpture of mesoscutellum peripherally: umbilicate foveate; umbilicate punctate. Microsculpture of mesoscutellum centrally: absent. Microsculpture of mesoscutellum peripherally: absent. Mesoscutellar rim: not expanded. Mesoscutellar rim medially: without notch. Mesofemoral depression: longitudinally striate dorsally, smooth ventrally. Metascutellum shape: not emarginate, concave but elevated posteriorly. Metascutellar setae: absent. Metascutellum sculpture: with large smooth posterior fovea. Postmarginal vein: present. Fore wing apex at rest: reaching middle of T5; reaching near apex of T5; reaching middle of T6. Coxae color brightness: same color as femora. Spines along tibiae: absent. Lateral propodeal carinae: broadly separated, not parallel anteriorly. Setae in metasomal depression: absent. Anterior sculpture of metasomal depression: absent. Median propodeal carina: absent.

T1 horn: present. Number of longitudinal carinae of T1 midlobe: obscured by other raised sculpture. T1 lateral carina: straight. T2 sculpture: densely foveolate, longitudinal sculpture irregular. T2 sublateral longitudinal foveae: absent. T3 metasomal flanges: absent. T4 sculpture: densely foveate, longitudinal sculpture irregular. T4 metasomal flanges: absent. T5 sculpture: densely foveate, longitudinal sculpture irregular. T5 metasomal flanges: absent. T6: longer than broad; broader than long. Major sculpture of T6: umbilicate punctate. Microsculpture of T6: absent. T6 medially: flat and tapering to a rounded apex, not separated from apical rim. T6 metasomal flanges: absent. T6 raised peripheral rim: absent. S4 sculpture: longitudinally striate or rugose, setal pits spanning interspaces. S5 sculpture: longitudinally striate to rugose, setal pits spanning interspaces. S5 median carina: absent. S6 peripheral carina: absent. S6 apex in relation to T6: not exposed to dorsal view. S6 apex: rounded or acuminate.

*Male*. Body length 2.95–3.6 mm (n=20). A3: longer than pedicel. A5 tyloid shape: narrow, linear. A6: broader than long. A11: longer than broad. Major sculpture of mesoscutal midlobe anteriorly: umbilicate foveate. Major sculpture of mesoscutal midlobe posteriorly: umbilicate foveate; longitudinally rugose. Microsculpture of mesoscutal midlobe anteriorly: granulate. Microsculpture of mesoscutal midlobe posteriorly: absent. Major sculpture of mesoscutellum centrally: umbilicate foveate. Major sculpture of mesoscutellum peripherally: umbilicate foveate. Microsculpture of mesoscutellum centrally: absent. Microsculpture of mesoscutellum peripherally: absent. Fore wing apex at rest: reaching middle of T5. T1 midlobe longitudinal carinae: 5. T3 metasomal flanges: absent. T4 metasomal flanges: absent. T5 metasomal flanges: absent. T6 metasomal flanges: absent. T7: truncate.

##### Diagnosis.

Both sexes: Frontal depression flat, with oblique interrupted carinae and sometimes with an incomplete longitudinal carina; submedian carina weak. Hyperoccipital carina indicated by sharp rugae. Occipital carina connected to hyperoccipital carina by a weak longitudinal carina or irregular ruga, laterally with strong corners and medially sinuate; area between occipital and hyperoccipital carinae densely sculptured. Mesoscutellum densely foveolate. Metascutellum flat, rugose. Postmarginal vein present. Coxa not darker than rest of leg. T1 lateral carina not expanded laterally. Metasomal flanges absent. Female: A3 not longer than pedicel. A4 slightly longer than broad, A5 broader than long. T1 with slight horn obscuring longitudinal carinae. Fore wing long enough to reach middle of T6. T6 broader than long. Male: A4 slightly longer than broad, A11 longer than broad. T1 midlobe with 3 longitudinal carinae. Fore wing long enough to exceed metasomal apex. T7 truncate or slightly emarginate apically. *Oxyscelio aciculae* is very similar to *Oxyscelio radii*, but has a shorter metasoma in males and females, with T6 broader than long in females. The metasoma in males of *Oxyscelio radii* is also very narrow compared with that of *Oxyscelio aciculae*.

##### Etymology.

Latin noun, genitive case, meaning “needle.”

##### Link to distribution map.

[http://hol.osu.edu/map-full.html?id=307063]

##### Material examined.

Holotype, female: AUSTRALIA: QLD, Mount Glorious, 1977, malaise trap, OSUC 368117 (deposited in QMBA). Paratypes: AUSTRALIA: 37 females, 37 males, Australian Museum K245256, K245257, K245263, K245273, K245275 (AMSA); ANIC DB 32-020074, 32-020886, OSUC 368113, OSUC 368114, OSUC 368135, OSUC 368137, OSUC 368138, OSUC 368142, OSUC 368159 (ANIC); OSUC 227551, 227611, 462583-462584 (CNCI); OSUC 368141, 368143, 368156, 368158, QDPC 0-165753 (QDPC); OSUC 368111 (QMBA); OSUC 448965 (UQIC); OSUC 368107-368110, 368112, 368115-368116, 368118-368123, 368131-368134, 368136, 368139-368140, 368145-368150, 368152-368155, 368157, 368160-368165, 368172, 368174, 448946-448948, 448951-448953, 448961-448964 (WINC).

#### Oxyscelio anfractus

Burks sp. n.

http://zoobank.org/D98EE916-6807-446F-AC13-A6E4A93F6AFA

urn:lsid:biosci.ohio-state.edu:osuc_concepts:307127

http://species-id.net/wiki/Oxyscelio_anfractus

**Figures 8–10.**
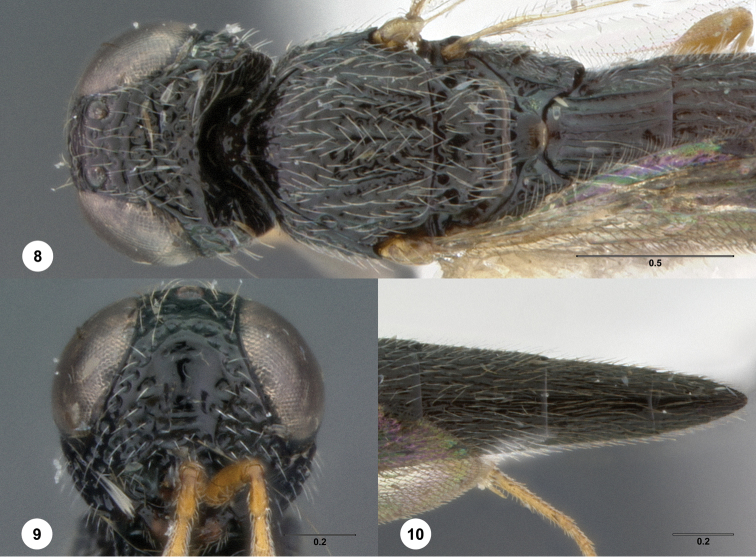
*Oxyscelio anfractus* sp. n., holotype female (OSUC 448564) **8** Head and mesosoma, lateral view **9** Head and mesosoma, dorsal view **10** Head, anterior view. Morphbank^27^

Figures 8–10 ; Morphbank^27^

##### Description.

Female. Body length 4.25 mm (n=1).

Radicle color and shade: darker than scape. Pedicel color: same as scape. A3: longer than pedicel. A4: broader than long. A5: broader than long.

Ventral clypeal margin: concave. Interantennal process: not elongate. Lower frons at dorsal margin of interantennal process: with oblique carina extending towards mouth corner. Transverse curved rugae extending from frontal depression to eye: absent. Median longitudinal carina in frontal depression: absent. Ventral portion of frontal depression: with transverse carinae. Dorsal portion of frontal depression: without transverse carinae. Submedian carina: present only as a weak shift in elevation. Frontal depression dorsally: not hood-like, open dorsally. Upper frons major sculpture: umbilicate foveate; irregularly rugose. Upper frons microsculpture: absent. Hyperoccipital carina: indicated by a set of irregular elevations. Carina connecting occipital carina to hyperoccipital carina: present. Occipital carina: present laterally, absent medially. Occiput sculpture: umbilicate foveate. Extra carina ventral to occipital carina: absent. Gena length: shorter than eye. Major sculpture of gena anteroventrally: umbilicate foveate. Major sculpture of gena posteroventrally: umbilicate foveate; rugose. Microsculpture of gena anteroventrally: absent. Microsculpture of gena posteroventrally: absent.

Lateral pronotal area sculpture: anteriorly smooth, posterodorsal corner with dense microsculpture, ventral corner with irregular carinae. Posterior border of central pronotal area: directed posteriorly, epomial carina absent or meeting transverse pronotal carina at arch on lateral surface of pronotum. Mesoscutum anteriorly: not steep, forming less than a right angle. Major sculpture of mesoscutal midlobe anteriorly: umbilicate foveate; transversely rugose. Mesoscutal midlobe sculpture at midlength: not different from nearby sculpture. Major sculpture of mesoscutal midlobe posteriorly: umbilicate foveate. Microsculpture of mesoscutal midlobe anteriorly: granulate. Microsculpture of mesoscutal midlobe posteriorly: absent. Median mesoscutal carina: present as a flattened or rounded elevation. Major sculpture of mesoscutellum centrally: umbilicate foveate. Major sculpture of mesoscutellum peripherally: umbilicate foveate. Microsculpture of mesoscutellum centrally: granulate. Microsculpture of mesoscutellum peripherally: granulate. Mesoscutellar rim: not expanded. Mesoscutellar rim medially: without notch. Mesofemoral depression: longitudinally striate dorsally, smooth ventrally. Metascutellum shape: not emarginate, convex dorsally. Metascutellar setae: absent. Metascutellum sculpture: with many longitudinal rugae. Postmarginal vein: present. Fore wing apex at rest: not reaching base of T5. Coxae color brightness: same color as femora. Spines along tibiae: absent. Lateral propodeal carinae: broadly separated, not parallel anteriorly. Setae in metasomal depression: unknown. Anterior sculpture of metasomal depression: unknown. Median propodeal carina: unknown.

T1 horn: present. Number of longitudinal carinae of T1 midlobe: 6. T1 lateral carina: straight. T2 sculpture: with longitudinal striae or rugae, setiferous puncta present between them. T2 sublateral longitudinal foveae: absent. T3 metasomal flanges: absent. T4 sculpture: longitudinally striate to rugose, setal pits spanning interspaces. T4 metasomal flanges: absent. T5 sculpture: longitudinally striate to rugose, setal pits spanning interspaces. T5 metasomal flanges: absent. T6: longer than broad. Major sculpture of T6: umbilicate punctate. Microsculpture of T6: absent. T6 medially: flat and tapering to a rounded apex, not separated from apical rim. T6 metasomal flanges: absent. T6 raised peripheral rim: absent. S4 sculpture: longitudinally striate or rugose, setal pits spanning interspaces. S5 sculpture: longitudinally striate to rugose, setal pits spanning interspaces. S5 median carina: present. S6 peripheral carina: absent. S6 apex in relation to T6: not exposed to dorsal view. S6 apex: rounded or acuminate.

*Male*. unknown.

##### Diagnosis.

Both sexes: Frontal depression concave, transverse carinae present ventrally; dorsal separator present as a complete carina; submedian carina weakly indicated by a single carina. Oblique carina extending from bottom of frontal depression towards lower margin of eye. Head directed downward. Hyperoccipital carina indicated by rugae. Occipital carina absent medially, with protruding lateral corners. Metascutellum convex. Postmarginal vein present. Coxa not darker than rest of leg. T1 lateral carina not expanded laterally. Metasomal flanges absent. Female: A3 longer than pedicel. A4, A5 broader than long. Mesoscutellum with granulate sculpture. T1 midlobe with strong anterior horn, 6 strong longitudinal carinae nevertheless distinct. Fore wing long enough to reach middle of T4. T6 longer than broad. *Oxyscelio anfractus* is very similar to *Oxyscelio mystacis*, especially in having a carina between the bottom of the frontal depression and the eye; it differs in several features mentioned in the diagnosis. From some similar Asian species in the *foveatus*-group, it differs in having strong longitudinal carinae on the T1 midlobe that are not obscured by the long T1 horn.

##### Etymology.

Latin noun, genitive case, meaning “a curve.”

##### Link to distribution map.

[http://hol.osu.edu/map-full.html?id=307127]

##### Material examined.

Holotype, female: PAPUA NEW GUINEA: East Sepik Prov., New Guinea Isl., Sepik River, Korogo, 8.III.1964, D. H. Colless, OSUC 448564 (deposited in ANIC).

#### Oxyscelio atricoxa

(Dodd)

http://zoobank.org/F96ABF90-FFB8-4580-9D55-43F1387D40C9

urn:lsid:biosci.ohio-state.edu:osuc_concepts:5006

http://species-id.net/wiki/Oxyscelio_atricoxa

**Figures 11–14.**
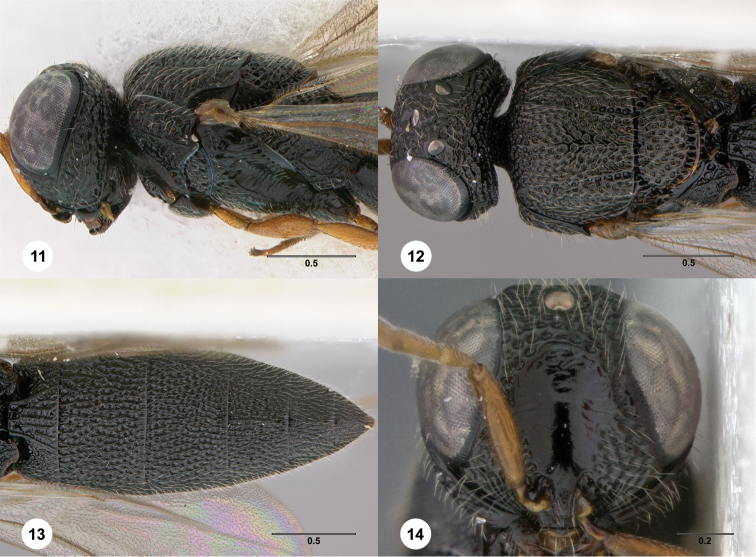
*Oxyscelio atricoxa* (Dodd), female (OSUC 429876) **11** Head and mesosoma, lateral view **12** Head and mesosoma, dorsal view **13** Metasoma, dorsal view. Female (OSUC 429885) **14** Head, anterior view. Morphbank^28^

Figures 11–14 ; Morphbank^28^

Sceliomorpha atricoxa Dodd, 1914: 104 (original description); Kieffer 1926: 303, 308 (description, keyed); Naumann et al. 1994: 71 (holotype transferred to ANIC).
Oxyscelio atricoxa (Dodd): Dodd 1931: 74 (generic transfer); Galloway 1976: 99 (type information).

##### Description.

Female. Body length 3.55–8.7 mm (n=20).

Radicle color and shade: darker than scape. Pedicel color: same as scape. A3: longer than pedicel. A4: longer than broad. A5: longer than broad; as long as broad.

Ventral clypeal margin: with slightly convex median lobe. Interantennal process: not elongate. Lower frons at dorsal margin of interantennal process: without transverse carina. Transverse curved rugae extending from frontal depression to eye: absent. Median longitudinal carina in frontal depression: absent. Ventral portion of frontal depression: smooth. Dorsal portion of frontal depression: with medially interrupted transverse carinae. Submedian carina: present only as a weak shift in elevation. Frontal depression dorsally: not hood-like, open dorsally. Upper frons major sculpture: umbilicate foveate. Upper frons microsculpture: granulate. Hyperoccipital carina: absent. Carina connecting occipital carina to hyperoccipital carina: absent. Occipital carina: weakly arched dorsally, with rounded lateral corners. Occiput sculpture: umbilicate foveate. Extra carina ventral to occipital carina: absent. Gena length: shorter than eye. Major sculpture of gena anteroventrally: umbilicate foveate. Major sculpture of gena posteroventrally: umbilicate foveate; rugose. Microsculpture of gena anteroventrally: absent. Microsculpture of gena posteroventrally: granulate.

Lateral pronotal area sculpture: anteriorly smooth, posterodorsal corner with dense microsculpture, ventral corner with irregular carinae. Posterior border of central pronotal area: directed anteriorly, protruding at corner of epomial carina and transverse pronotal carina. Mesoscutum anteriorly: very steep and tall, descending at a right angle or protruding anteriorly. Major sculpture of mesoscutal midlobe anteriorly: umbilicate foveate. Mesoscutal midlobe sculpture at midlength: not different from nearby sculpture. Major sculpture of mesoscutal midlobe posteriorly: umbilicate foveate; longitudinally rugose. Microsculpture of mesoscutal midlobe anteriorly: granulate. Microsculpture of mesoscutal midlobe posteriorly: absent. Median mesoscutal carina: present as a vague, occasionally interrupted elevation. Major sculpture of mesoscutellum centrally: umbilicate foveate. Major sculpture of mesoscutellum peripherally: umbilicate foveate. Microsculpture of mesoscutellum centrally: absent. Microsculpture of mesoscutellum peripherally: absent. Mesoscutellar rim: not expanded. Mesoscutellar rim medially: without notch. Mesofemoral depression: longitudinally striate dorsally, smooth ventrally. Metascutellum shape: slightly emarginate posteriorly, concave but elevated posteriorly. Metascutellar setae: absent. Metascutellum sculpture: with large smooth posterior fovea. Postmarginal vein: absent. Fore wing apex at rest: reaching near apex of T5; reaching middle of T6. Coxae color brightness: darker than femora. Spines along tibiae: absent. Lateral propodeal carinae: broadly separated, not parallel anteriorly. Setae in metasomal depression: absent. Anterior sculpture of metasomal depression: absent. Median propodeal carina: absent.

T1 horn: absent. Number of longitudinal carinae of T1 midlobe: 5. T1 lateral carina: protruding laterally, visible from ventral view. T2 sculpture: densely foveolate, longitudinal sculpture irregular. T2 sublateral longitudinal foveae: absent. T3 metasomal flanges: absent. T4 sculpture: longitudinally striate to rugose, setal pits spanning interspaces. T4 metasomal flanges: absent. T5 sculpture: longitudinally striate to rugose, setal pits spanning interspaces. T5 metasomal flanges: absent. T6: broader than long. Major sculpture of T6: umbilicate foveate. Microsculpture of T6: absent. T6 medially: flat and tapering to a rounded apex, not separated from apical rim. T6 metasomal flanges: absent. T6 raised peripheral rim: absent. S4 sculpture: longitudinally striate or rugose, setal pits spanning interspaces. S5 sculpture: longitudinally striate to rugose, setal pits spanning interspaces. S5 median carina: absent. S6 peripheral carina: absent. S6 apex in relation to T6: not exposed to dorsal view. S6 apex: rounded or acuminate.

*Male*. unknown.

##### Diagnosis.

Both sexes: Frontal depression shallow, not parallel-sided, many interrupted transverse carinae present, including some above dorsal separator; submedian carina weakly indicated but complete. Hyperoccipital carina indicated by weak rugae. Occipital carina complete, weakly convex medially. Metascutellum deeply concave, emarginate apically, projecting dorsally. Postmarginal vein absent. Coxa darker than rest of leg. T1 lateral carina expanded laterally. Metasomal flanges absent. Female only: A3 much longer than pedicel. A4 longer than broad, A5 nearly as long as broad. Mesoscutellum without granulate sculpture. T1 midlobe with 4 longitudinal carinae or with raised sculpture obscuring them. T6 without metasomal flanges, main body of tergum not separated from apical rim. Fore wing long enough to reach middle of T5 or middle of T6. *Oxyscelio atricoxa* can be distinguished from *Oxyscelio conjuncti* mainly in having a shallower frontal depression, finer and denser mesoscutal and mesoscutellar foveae, and by the shape of T6. Males are unknown, but likely differ from those of *Oxyscelio conjuncti* mostly in depth of the frontal depression.

##### Link to distribution map.

[http://hol.osu.edu/map-full.html?id=5006]

##### Material examined.

Holotype, female, S. atricoxa: AUSTRALIA: NSW, Sydney, Elizabeth Bay, no date, ANIC DB 32-012551 (deposited in ANIC). Other material: AUSTRALIA: 67 females, OSUC 429912-429917, 429919 (ANIC); OSUC 462560 (CNCI); OSUC 281687-281688, 309399 (OSUC); QDPC 0-165655, 0-165726, 0-165754 (QDPC); OSUC 429918 (QMBA); OSUC 437012 (UQIC); OSUC 429876-429911, 429920-429921, 448968-448980 (WINC).

#### Oxyscelio bellariorum

Burks sp. n.

http://zoobank.org/7FC6E9B1-A5F3-4877-B4B0-A1E428550121

urn:lsid:biosci.ohio-state.edu:osuc_concepts:307064

http://species-id.net/wiki/Oxyscelio_bellariorum

**Figures 15–18.**
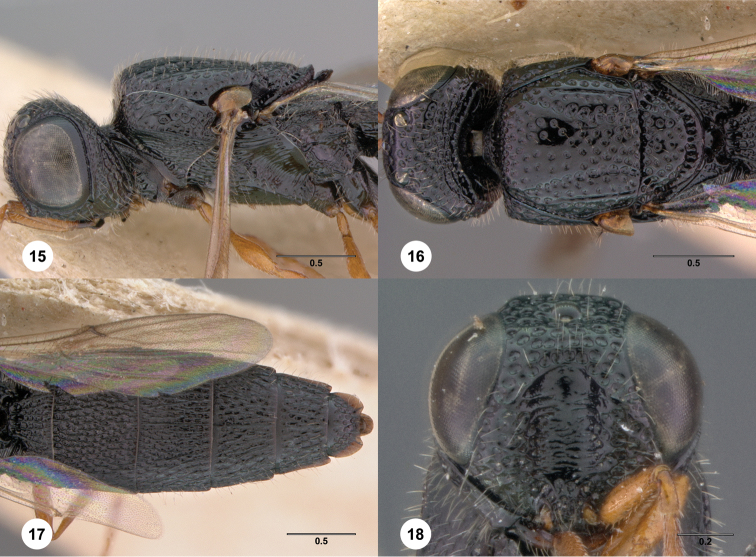
*Oxyscelio bellariorum* sp. n., paratype female (OSUC 435954) **15** Head and mesosoma, lateral view **16** Head and mesosoma, dorsal view **17** Head, anterior view **18** Metasoma, dorsal view. Morphbank^29^

Figures 15–18 ; Morphbank^29^

##### Description.

Female. Body length 4.5–4.9 mm (n=12).

Radicle color and shade: darker than scape. Pedicel color: same as scape. A3: longer than pedicel. A4: broader than long. A5: broader than long.

Ventral clypeal margin: with slightly convex median lobe. Interantennal process: not elongate. Lower frons at dorsal margin of interantennal process: without transverse carina. Transverse curved rugae extending from frontal depression to eye: absent. Median longitudinal carina in frontal depression: absent. Ventral portion of frontal depression: with transverse carinae. Dorsal portion of frontal depression: with some transverse carinae. Submedian carina: present. Frontal depression dorsally: not hood-like, open dorsally. Upper frons major sculpture: umbilicate foveate. Upper frons microsculpture: absent. Hyperoccipital carina: absent. Carina connecting occipital carina to hyperoccipital carina: absent. Occipital carina: weakly arched dorsally, with rounded lateral corners. Occiput sculpture: umbilicate foveate. Extra carina ventral to occipital carina: present, medially incomplete. Gena length: shorter than eye. Major sculpture of gena anteroventrally: umbilicate foveate; rugose. Major sculpture of gena posteroventrally: umbilicate foveate; rugose. Microsculpture of gena anteroventrally: absent. Microsculpture of gena posteroventrally: absent.

Lateral pronotal area sculpture: with shallow irregular carinae, posterodorsal corner with dense microsculpture. Posterior border of central pronotal area: directed anteriorly, protruding at corner of epomial carina and transverse pronotal carina. Mesoscutum anteriorly: very steep and tall, descending at a right angle or protruding anteriorly. Major sculpture of mesoscutal midlobe anteriorly: umbilicate foveate. Mesoscutal midlobe sculpture at midlength: with large smooth areas. Major sculpture of mesoscutal midlobe posteriorly: umbilicate foveate; longitudinally rugose. Microsculpture of mesoscutal midlobe anteriorly: granulate. Microsculpture of mesoscutal midlobe posteriorly: absent. Median mesoscutal carina: absent. Major sculpture of mesoscutellum centrally: umbilicate foveate. Major sculpture of mesoscutellum peripherally: umbilicate foveate. Microsculpture of mesoscutellum centrally: absent. Microsculpture of mesoscutellum peripherally: absent; punctate. Mesoscutellar rim: expanded. Mesoscutellar rim medially: with notch. Mesofemoral depression: longitudinally striate dorsally, smooth ventrally. Metascutellum shape: not emarginate, concave but elevated posteriorly. Metascutellar setae: absent. Metascutellum sculpture: with large smooth posterior fovea. Postmarginal vein: absent. Fore wing apex at rest: reaching base of T5. Coxae color brightness: darker than femora. Spines along tibiae: absent. Lateral propodeal carinae: broadly separated, not parallel anteriorly. Setae in metasomal depression: absent. Anterior sculpture of metasomal depression: absent. Median propodeal carina: absent.

T1 horn: absent. Number of longitudinal carinae of T1 midlobe: 4. T1 lateral carina: protruding laterally, visible from ventral view. T2 sculpture: with longitudinal striae or rugae, setiferous puncta present between them. T2 sublateral longitudinal foveae: absent. T3 metasomal flanges: present. T4 sculpture: longitudinally striate to rugose, setal pits spanning interspaces. T4 metasomal flanges: present as slightly protruding sharp corners. T5 sculpture: longitudinally striate to rugose, setal pits spanning interspaces. T5 metasomal flanges: present, blade-like with rounded margins. T6: broader than long. Major sculpture of T6: longitudinally striate; umbilicate foveate. Microsculpture of T6: absent. T6 medially: with deep emargination that is V-shaped medially, separated from apical rim. T6 metasomal flanges: present, very broadly rounded, with rounded apices. T6 raised peripheral rim: absent. S4 sculpture: longitudinally striate or rugose, setal pits spanning interspaces. S5 sculpture: longitudinally striate to rugose, setal pits spanning interspaces. S5 median carina: absent. S6 peripheral carina: present, posteriorly complete. S6 apex in relation to T6: exposed to dorsal view by T6 emargination. S6 apex: rounded or acuminate.

*Male*. unknown.

##### Diagnosis.

Both sexes: Frontal depression deep, all carinae except dorsal separator interrupted medially, carinae present above dorsal separator; submedian carina weakly defined or absent medially. Hyperoccipital carina absent. Occipital carina complete, medially sinuate. Mesoscutal midlobe weakly sculptured with some smooth areas near midlength. Mesoscutellar rim not expanded, with median notch. Metascutellum scoop-like, projecting dorsally. Coxa darker than rest of leg. Postmarginal vein absent. Female: A3 longer than pedicel. A4 not longer than broad, A5 broader than long. T4 with weak metasomal flanges. T5 with lobe-like metasomal flanges. T6 with broad, wavy lateral metasomal flanges. T6 broadly and deeply emarginate, roundly concave medially. S6 exposed to dorsal view, rounded apically. This species is similar to *Oxyscelio incisurae* and *Oxyscelio funis*, but has a shorter antenna than in *Oxyscelio funis* and weaker sculpture than in *Oxyscelio incisurae*.

##### Etymology.

Latin noun, genitive case, meaning “confectioneries.”

##### Link to distribution map.

[http://hol.osu.edu/map-full.html?id=307064]

##### Material examined.

Holotype, female: AUSTRALIA: NSW, Pilliga, 1.II.1931, A. P. Dodd, OSUC 435957 (deposited in ANIC). Paratypes: AUSTRALIA: 11 females, OSUC 435950-435956, 435958-435960 (ANIC); OSUC 449096 (WINC).

#### Oxyscelio bicoloripedis

Burks sp. n.

http://zoobank.org/96B81714-73D9-4C0B-B51D-F9D0C745F35E

urn:lsid:biosci.ohio-state.edu:osuc_concepts:307123

http://species-id.net/wiki/Oxyscelio_bicoloripedis

**Figures 19–22.**
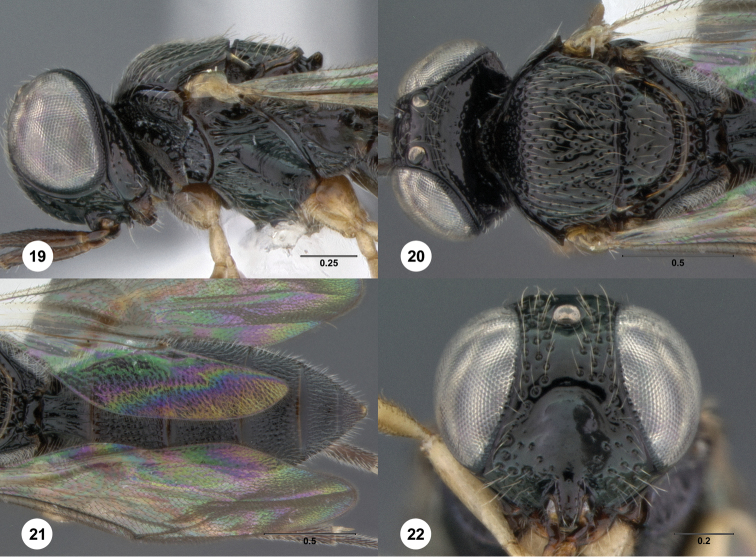
*Oxyscelio bicoloripedis* sp. n., holotype female (OSUC 225506) **19** Head and mesosoma, lateral view **20** Head and mesosoma, dorsal view **21** Metasoma, dorsal view. Paratype female (OSUC 221895) **22** Head, anterior view. Morphbank^30^

Figures 19–22 ; Morphbank^30^

##### Description.

Female. Body length 3.25–3.4 mm (n=2).

Radicle color and shade: same as scape, both dark brown. Pedicel color: same as scape. A3: longer than pedicel. A4: broader than long. A5: broader than long.

Ventral clypeal margin: with slightly convex median lobe. Interantennal process: not elongate. Lower frons at dorsal margin of interantennal process: without transverse carina. Transverse curved rugae extending from frontal depression to eye: absent. Median longitudinal carina in frontal depression: absent. Ventral portion of frontal depression: with medially interrupted transverse carinae. Dorsal portion of frontal depression: without transverse carinae. Submedian carina: absent; present only as a weak shift in elevation. Frontal depression dorsally: not hood-like, open dorsally. Upper frons major sculpture: umbilicate foveate. Upper frons microsculpture: punctate. Hyperoccipital carina: present as a single carina. Carina connecting occipital carina to hyperoccipital carina: absent. Occipital carina: present laterally, absent medially. Occiput sculpture: smooth; umbilicate punctate. Extra carina ventral to occipital carina: absent. Gena length: shorter than eye. Major sculpture of gena anteroventrally: umbilicate punctate. Major sculpture of gena posteroventrally: umbilicate punctate; absent. Microsculpture of gena anteroventrally: absent. Microsculpture of gena posteroventrally: absent.

Lateral pronotal area sculpture: anteriorly smooth, posterodorsal corner with dense microsculpture, ventral corner with irregular carinae. Posterior border of central pronotal area: directed posteriorly, epomial carina absent or meeting transverse pronotal carina at arch on lateral surface of pronotum. Mesoscutum anteriorly: not steep, forming less than a right angle. Major sculpture of mesoscutal midlobe anteriorly: umbilicate foveate. Mesoscutal midlobe sculpture at midlength: not different from nearby sculpture. Major sculpture of mesoscutal midlobe posteriorly: umbilicate foveate; longitudinally rugose. Microsculpture of mesoscutal midlobe anteriorly: granulate. Microsculpture of mesoscutal midlobe posteriorly: absent. Median mesoscutal carina: absent. Major sculpture of mesoscutellum centrally: umbilicate foveate. Major sculpture of mesoscutellum peripherally: umbilicate foveate. Microsculpture of mesoscutellum centrally: absent. Microsculpture of mesoscutellum peripherally: absent. Mesoscutellar rim: not expanded. Mesoscutellar rim medially: without notch. Mesofemoral depression: longitudinally striate dorsally, smooth ventrally. Metascutellum shape: slightly emarginate posteriorly, concave but elevated posteriorly. Metascutellar setae: absent. Metascutellum sculpture: with a median carina, otherwise weakly sculptured. Postmarginal vein: present. Fore wing apex at rest: exceeding metasomal apex. Coxae color brightness: same color as femora. Spines along tibiae: absent. Lateral propodeal carinae: broadly separated, not parallel anteriorly. Setae in metasomal depression: present. Anterior sculpture of metasomal depression: with median areole or pair of pits. Median propodeal carina: absent.

T1 horn: absent. Number of longitudinal carinae of T1 midlobe: 4. T1 lateral carina: straight. T2 sculpture: densely foveolate, longitudinal sculpture irregular. T2 sublateral longitudinal foveae: absent. T3 metasomal flanges: absent. T4 sculpture: densely foveate, longitudinal sculpture irregular. T4 metasomal flanges: absent. T5 sculpture: longitudinally striate to rugose, setal pits spanning interspaces. T5 metasomal flanges: absent. T6: broader than long. Major sculpture of T6: umbilicate punctate. Microsculpture of T6: absent. T6 medially: flat and tapering to a rounded apex, not separated from apical rim. T6 metasomal flanges: absent. T6 raised peripheral rim: absent. S4 sculpture: longitudinally striate or rugose, setal pits spanning interspaces. S5 sculpture: longitudinally striate to rugose, setal pits spanning interspaces. S5 median carina: absent. S6 peripheral carina: absent. S6 apex in relation to T6: not exposed to dorsal view. S6 apex: rounded or acuminate.

*Male*. unknown.

##### Diagnosis.

Both sexes: Frontal depression shallow; submedian carina weakly indicated by a rounded carina. Hyperoccipital carina indicated by one complete carina that may be accompanied by an additional ruga. Occipital carina incomplete, lateral portions approaching hyperoccipital carina. Occiput umbilicate punctate. Metascutellum broad and short, concave, with a distinct median carina. Postmarginal vein present. Coxa and rest of leg bicolored. T1 lateral carina not expanded laterally. Metasomal flanges absent. Female: A3 longer than pedicel. A4, A5 broader than long. Mesoscutellum with small umbilicate foveae. T1 midlobe without anterior horn, with 4 longitudinal carinae. Fore wing long enough to reach beyond metasomal apex. T6 broader than long. *Oxyscelio bicoloripedis* is very different from the other New Caledonian species of *Oxyscelio*. It belongs to the *flavipes*-group like the others, but lacks the extra carinae near the submedian carina, especially. Like other New Caledonian species, it has bicolored legs.

##### Etymology.

Latin noun, genitive case, meaning “bicolored foot.”

##### Link to distribution map.

[http://hol.osu.edu/map-full.html?id=307123]

##### Material examined.

Holotype, female: NEW CALEDONIA: Sud Prov., Riviere Bleue Territorial Park, 22°12.967'S, 166°39.267'E, 201m, 17.XI-22.XI.1998, malaise trap, M. E. Irwin & D. W. Webb, OSUC 225506 (deposited in MNHN). Paratype: NEW CALEDONIA: 1 female, OSUC 221895 (OSUC).

#### Oxyscelio brevitas

Burks sp. n.

http://zoobank.org/46B04FD3-FBC3-474A-93EF-127CE86C0117

urn:lsid:biosci.ohio-state.edu:osuc_concepts:307120

http://species-id.net/wiki/Oxyscelio_brevitas

**Figures 23–26.**
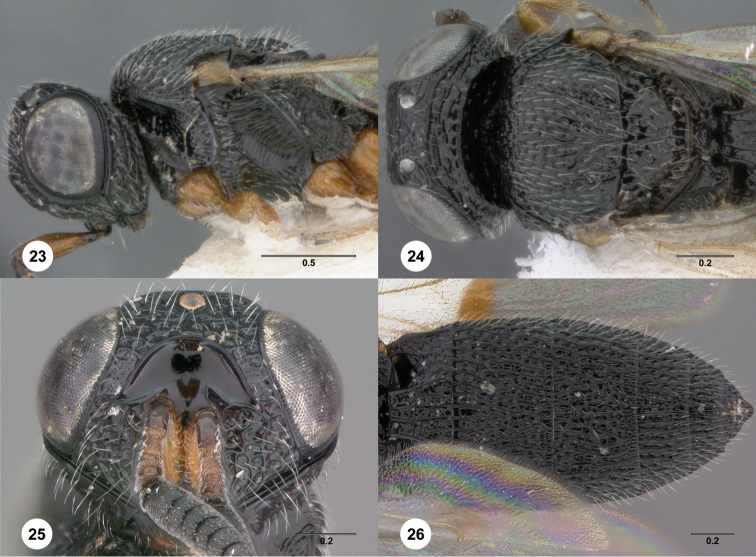
*Oxyscelio brevitas* sp. n., holotype female (OSUC 441375) **23** Head and mesosoma, lateral view **24** Head and mesosoma, dorsal view **25** Head, anterior view **26** Metasoma, dorsal view. Morphbank^31^

Figures 23–26 ; Morphbank^31^

##### Description.

Female. Body length 2.4–3.75 mm (n=6).

Radicle color and shade: darker than scape. Pedicel color: at least partially darker than scape. A3: shorter than pedicel. A4: broader than long. A5: broader than long.

Ventral clypeal margin: with slightly convex median lobe. Interantennal process: not elongate. Lower frons at dorsal margin of interantennal process: without transverse carina. Transverse curved rugae extending from frontal depression to eye: absent. Median longitudinal carina in frontal depression: absent. Ventral portion of frontal depression: smooth. Dorsal portion of frontal depression: without transverse carinae. Submedian carina: present. Frontal depression dorsally: not hood-like, open dorsally. Upper frons major sculpture: umbilicate foveate; irregularly rugose. Upper frons microsculpture: absent. Hyperoccipital carina: present as a single carina; indicated by a set of irregular elevations. Carina connecting occipital carina to hyperoccipital carina: absent. Occipital carina: omicron-shaped, with sharp corners where median portion meets lateral portions. Occiput sculpture: irregularly sculptured. Extra carina ventral to occipital carina: present, complete. Gena length: shorter than eye. Major sculpture of gena anteroventrally: umbilicate foveate; rugose. Major sculpture of gena posteroventrally: umbilicate foveate; rugose. Microsculpture of gena anteroventrally: absent; granulate. Microsculpture of gena posteroventrally: absent; granulate.

Lateral pronotal area sculpture: anteriorly smooth, posterodorsal corner with dense microsculpture, ventral corner with irregular carinae. Posterior border of central pronotal area: directed anteriorly, protruding at corner of epomial carina and transverse pronotal carina. Mesoscutum anteriorly: very steep and tall, descending at a right angle or protruding anteriorly. Major sculpture of mesoscutal midlobe anteriorly: umbilicate foveate. Mesoscutal midlobe sculpture at midlength: not different from nearby sculpture. Major sculpture of mesoscutal midlobe posteriorly: umbilicate foveate; longitudinally rugose. Microsculpture of mesoscutal midlobe anteriorly: granulate. Microsculpture of mesoscutal midlobe posteriorly: absent. Median mesoscutal carina: present as a vague, occasionally interrupted elevation. Major sculpture of mesoscutellum centrally: umbilicate foveate. Major sculpture of mesoscutellum peripherally: umbilicate foveate. Microsculpture of mesoscutellum centrally: punctate. Microsculpture of mesoscutellum peripherally: punctate. Mesoscutellar rim: not expanded. Mesoscutellar rim medially: without notch. Mesofemoral depression: longitudinally striate dorsally, smooth ventrally. Metascutellum shape: slightly emarginate posteriorly, concave but elevated posteriorly. Metascutellar setae: absent. Metascutellum sculpture: with large smooth posterior fovea. Postmarginal vein: absent. Fore wing apex at rest: reaching near apex of T5; reaching middle of T6. Coxae color brightness: same color as femora. Spines along tibiae: absent. Lateral propodeal carinae: broadly separated, but parallel for a short distance anteriorly. Setae in metasomal depression: absent. Anterior sculpture of metasomal depression: absent. Median propodeal carina: absent.

T1 horn: absent. Number of longitudinal carinae of T1 midlobe: 4. T1 lateral carina: protruding laterally, visible from ventral view. T2 sculpture: with longitudinal striae or rugae, setiferous puncta present between them. T2 sublateral longitudinal foveae: absent. T3 metasomal flanges: absent. T4 sculpture: longitudinally striate to rugose, setal pits spanning interspaces. T4 metasomal flanges: absent. T5 sculpture: longitudinally striate to rugose, setal pits spanning interspaces. T5 metasomal flanges: present as strong posterior corners. T6: broader than long. Major sculpture of T6: umbilicate punctate. Microsculpture of T6: absent. T6 medially: strongly convex, tapering and sloping down to a rounded apex, not separated from apical rim. T6 metasomal flanges: absent. T6 raised peripheral rim: absent. S4 sculpture: longitudinally striate or rugose, setal pits spanning interspaces. S5 sculpture: longitudinally striate to rugose, setal pits spanning interspaces. S5 median carina: present. S6 peripheral carina: absent. S6 apex in relation to T6: not exposed to dorsal view. S6 apex: rounded or acuminate.

*Male*. unknown.

##### Diagnosis.

Both sexes: Mesoscutum and mesoscutellum black. Frontal depression deep, transverse carinae absent; submedian carina strong. Hyperoccipital carina indicated by strong rugae. Occipital carina complete, convex medially. Metascutellum concave, broad and short, projecting dorsally. Coxa not darker than rest of leg. T1 lateral carina expanded laterally. Female only: A3 shorter than pedicel. A4, A5 much broader than long. T1 midlobe with 4 longitudinal carinae. T6 without metasomal flanges. Fore wing long enough to reach or exceed metasomal apex.

##### Etymology.

Latin noun in apposition, meaning “the short.”

##### Link to distribution map.

[http://hol.osu.edu/map-full.html?id=307120]

##### Material examined.

Holotype, female: AUSTRALIA: ACT, Canberra, 4.II.1964, I. D. Galloway, OSUC 441375 (deposited in ANIC). Paratypes: AUSTRALIA: 6 females, OSUC 437878, 441376-441378 (ANIC); OSUC 441374, 451319 (WINC).

##### Comments.

This species can be recognized by its extremely short A3, A4, and A5. Additionally, the strongly defined frontal depression and submedian carina are unusual for a short-bodied species.

#### Oxyscelio catenae

Burks sp. n.

http://zoobank.org/DEA01D2A-6CE1-4ADA-B019-6834CBC43B1B

urn:lsid:biosci.ohio-state.edu:osuc_concepts:307065

http://species-id.net/wiki/Oxyscelio_catenae

**Figures 27–32.**
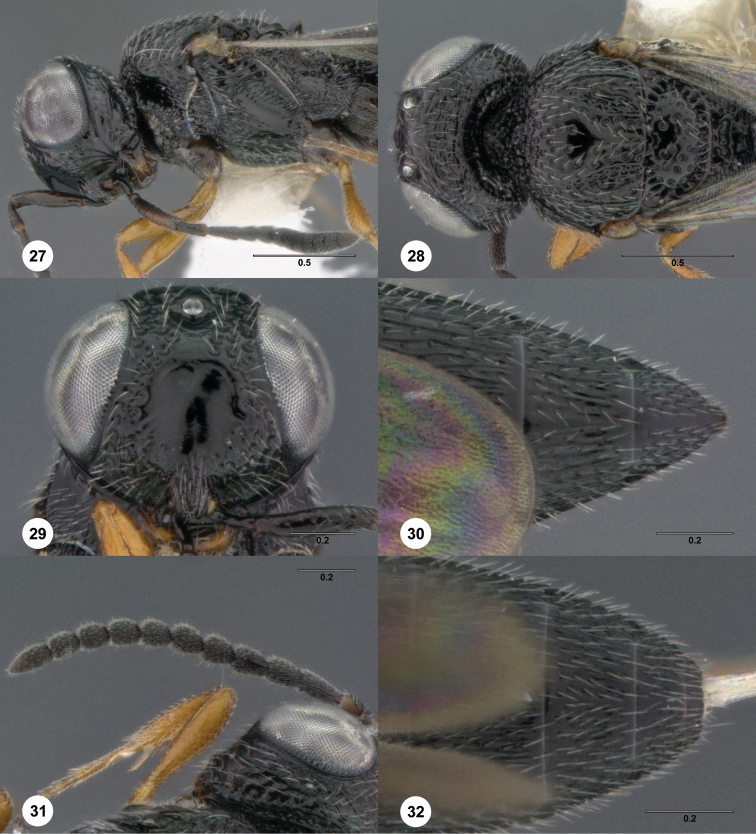
*Oxyscelio catenae* sp. n., paratype female (OSUC 448980) **27** Head and mesosoma, lateral view. Holotype female (OSUC 448987) **28** Head and mesosoma, dorsal view **29** Head, anterior view **30** Metasoma, dorsal view. Paratype male (OSUC 439569) **31** Antenna **32** Metasoma, dorsal view. Morphbank^32^

Figures 27–32 ; Morphbank^32^

##### Description.

Female. Body length 3.5–4.05 mm (n=11).

Radicle color and shade: darker than scape; same as scape, both dark brown. Pedicel color: same as scape. A3: longer than pedicel. A4: longer than broad. A5: broader than long.

Ventral clypeal margin: with slightly convex median lobe. Interantennal process: not elongate. Lower frons at dorsal margin of interantennal process: without transverse carina. Transverse curved rugae extending from frontal depression to eye: absent. Median longitudinal carina in frontal depression: absent. Ventral portion of frontal depression: smooth. Dorsal portion of frontal depression: without transverse carinae. Submedian carina: present only as a weak shift in elevation. Frontal depression dorsally: not hood-like, open dorsally. Upper frons major sculpture: umbilicate foveate; transversely rugose. Upper frons microsculpture: granulate. Hyperoccipital carina: indicated by a set of irregular elevations. Carina connecting occipital carina to hyperoccipital carina: absent. Occipital carina: uniformly rounded dorsally. Occiput sculpture: irregularly sculptured. Extra carina ventral to occipital carina: present, medially incomplete. Gena length: shorter than eye. Major sculpture of gena anteroventrally: umbilicate foveate; rugose. Major sculpture of gena posteroventrally: umbilicate foveate. Microsculpture of gena anteroventrally: absent. Microsculpture of gena posteroventrally: granulate.

Lateral pronotal area sculpture: densely covered with setiferous puncta. Posterior border of central pronotal area: directed posteriorly, epomial carina absent or meeting transverse pronotal carina at arch on lateral surface of pronotum. Mesoscutum anteriorly: very steep and tall, descending at a right angle or protruding anteriorly. Major sculpture of mesoscutal midlobe anteriorly: umbilicate foveate. Mesoscutal midlobe sculpture at midlength: with large smooth areas. Major sculpture of mesoscutal midlobe posteriorly: umbilicate foveate; longitudinally rugose. Microsculpture of mesoscutal midlobe anteriorly: granulate. Microsculpture of mesoscutal midlobe posteriorly: absent. Median mesoscutal carina: present as a vague, occasionally interrupted elevation. Major sculpture of mesoscutellum centrally: absent; umbilicate foveate. Major sculpture of mesoscutellum peripherally: umbilicate foveate. Microsculpture of mesoscutellum centrally: absent. Microsculpture of mesoscutellum peripherally: punctate. Mesoscutellar rim: not expanded. Mesoscutellar rim medially: without notch. Mesofemoral depression: longitudinally striate dorsally, smooth ventrally. Metascutellum shape: slightly emarginate posteriorly, concave but elevated posteriorly. Metascutellar setae: absent. Metascutellum sculpture: with large smooth posterior fovea. Postmarginal vein: present. Fore wing apex at rest: reaching base of T5. Coxae color brightness: darker than femora. Spines along tibiae: absent. Lateral propodeal carinae: broadly separated, but parallel for a short distance anteriorly. Setae in metasomal depression: absent. Anterior sculpture of metasomal depression: with median areole or pair of pits. Median propodeal carina: absent.

T1 horn: absent. Number of longitudinal carinae of T1 midlobe: obscured by other raised sculpture. T1 lateral carina: straight. T2 sculpture: with longitudinal striae or rugae, setiferous puncta present between them. T2 sublateral longitudinal foveae: absent. T3 metasomal flanges: absent. T4 sculpture: longitudinally striate to rugose, setal pits spanning interspaces. T4 metasomal flanges: absent. T5 sculpture: longitudinally striate to rugose, setal pits spanning interspaces. T5 metasomal flanges: absent. T6: broader than long. Major sculpture of T6: umbilicate punctate; longitudinally striate. Microsculpture of T6: absent. T6 medially: flat and tapering to a rounded apex, not separated from apical rim. T6 metasomal flanges: absent. T6 raised peripheral rim: absent. S4 sculpture: longitudinally striate or rugose, setal pits spanning interspaces. S5 sculpture: longitudinally striate to rugose, setal pits spanning interspaces. S5 median carina: absent. S6 peripheral carina: absent. S6 apex in relation to T6: not exposed to dorsal view. S6 apex: truncate.

*Male*. Body length 3.4–3.55 mm (n=4). A3: longer than pedicel. A5 tyloid shape: narrow, linear. A6: longer than broad. A11: longer than broad. Major sculpture of mesoscutal midlobe anteriorly: umbilicate foveate. Major sculpture of mesoscutal midlobe posteriorly: umbilicate foveate; longitudinally rugose. Microsculpture of mesoscutal midlobe anteriorly: granulate. Microsculpture of mesoscutal midlobe posteriorly: absent. Major sculpture of mesoscutellum centrally: umbilicate foveate; absent. Major sculpture of mesoscutellum peripherally: umbilicate foveate. Microsculpture of mesoscutellum centrally: absent. Microsculpture of mesoscutellum peripherally: absent. Fore wing apex at rest: reaching middle of T5. T1 midlobe longitudinal carinae: 4. T3 metasomal flanges: absent. T4 metasomal flanges: absent. T5 metasomal flanges: absent. T6 metasomal flanges: absent. T7: weakly emarginate.

**Diagnosis**. Both sexes: Frontal depression shallow, transverse carinae absent; submedian carina weak and rounded. Genal carina expanded, with large foveae between it and gena laterally. Hyperoccipital carina indicated by rugae. Occipital carina complete, convex. Epomial corner sharp, protruding. Mesoscutum with raised longitudinal smooth area postero-medially. Metascutellum broad, deeply concave, emarginate apically. Postmarginal vein present. Coxa darker than rest of leg. Metasomal depression with irregular sculpture. T1 lateral carina not expanded laterally. Metasomal flanges absent. Female: A3 longer than pedicel. A4 longer than broad, A5 nearly as long as broad. Mesoscutellum without granulate sculpture. T1 midlobe carinae obscured by raised area. Fore wing long enough to reach middle of T5. Male: A4, A11 longer than broad. T1 midlobe with 4 longitudinal carinae. Fore wing long enough to reach middle of T6. T7 truncate or slightly emarginate apically, without apical protrusions. *Oxyscelio catenae* is unusual in having sharp epomial corners and a raised smooth strip on the mesoscutal midlobe postero-medially. These features are shared with *Oxyscelio valdecatenae*, which has a more strongly sculptured mesoscutellum and shorter body and metasoma.

##### Etymology.

Latin noun, genitive case, meaning “chain.”

##### Link to distribution map.

[http://hol.osu.edu/map-full.html?id=307065]

##### Material examined.

Holotype, female: AUSTRALIA: WA, Mount Cooke, 28.I-17.II.1991, malaise trap, M. S. Harvey & J. M. Waldock, OSUC 448987 (deposited in WAMP). Paratypes: AUSTRALIA: 10 females, 4 males, OSUC 439567-439569, 448986, 448988-448997 (WINC).

#### Oxyscelio caudarum

Burks sp. n.

http://zoobank.org/1786EBF8-AB3F-40A3-B7D8-CA65E40B352B

urn:lsid:biosci.ohio-state.edu:osuc_concepts:307066

http://species-id.net/wiki/Oxyscelio_caudarum

**Figures 33–36.**
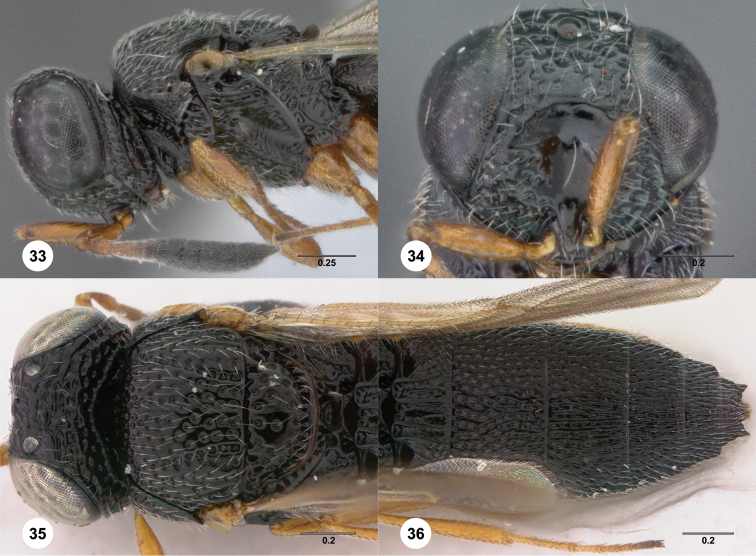
*Oxyscelio caudarum* sp. n., holotype female (OSUC 437874) **33** Head and mesosoma, lateral view **34** Head and mesosoma, dorsal view **35** Head, anterior view **36** Metasoma, dorsal view. Morphbank^33^

Figures 33–36 ; Morphbank^33^

##### Description.

Female. Body length 2.8–3.1 mm (n=4).

Radicle color and shade: same as scape, both yellowish or reddish. Pedicel color: same as scape. A3: shorter than pedicel. A4: broader than long. A5: broader than long.

Ventral clypeal margin: with slightly convex median lobe. Interantennal process: not elongate. Lower frons at dorsal margin of interantennal process: without transverse carina. Transverse curved rugae extending from frontal depression to eye: absent. Median longitudinal carina in frontal depression: absent. Ventral portion of frontal depression: smooth. Dorsal portion of frontal depression: without transverse carinae. Submedian carina: present only as a weak shift in elevation. Frontal depression dorsally: not hood-like, open dorsally. Upper frons major sculpture: umbilicate foveate. Upper frons microsculpture: granulate. Hyperoccipital carina: absent. Carina connecting occipital carina to hyperoccipital carina: absent. Occipital carina: broadly angular, with rounded median peak. Occiput sculpture: umbilicate foveate. Extra carina ventral to occipital carina: present, medially incomplete. Gena length: shorter than eye. Major sculpture of gena anteroventrally: umbilicate foveate. Major sculpture of gena posteroventrally: umbilicate foveate. Microsculpture of gena anteroventrally: absent. Microsculpture of gena posteroventrally: absent.

Lateral pronotal area sculpture: with shallow irregular carinae, posterodorsal corner with dense microsculpture. Posterior border of central pronotal area: directed anteriorly, protruding at corner of epomial carina and transverse pronotal carina. Mesoscutum anteriorly: very steep and tall, descending at a right angle or protruding anteriorly. Major sculpture of mesoscutal midlobe anteriorly: umbilicate foveate. Mesoscutal midlobe sculpture at midlength: not different from nearby sculpture. Major sculpture of mesoscutal midlobe posteriorly: umbilicate foveate. Microsculpture of mesoscutal midlobe anteriorly: granulate. Microsculpture of mesoscutal midlobe posteriorly: absent. Median mesoscutal carina: present as a vague, occasionally interrupted elevation. Major sculpture of mesoscutellum centrally: umbilicate foveate. Major sculpture of mesoscutellum peripherally: longitudinally rugose. Microsculpture of mesoscutellum centrally: absent. Microsculpture of mesoscutellum peripherally: absent. Mesoscutellar rim: not expanded. Mesoscutellar rim medially: without notch. Mesofemoral depression: longitudinally striate dorsally, smooth ventrally. Metascutellum shape: slightly emarginate posteriorly, concave but elevated posteriorly. Metascutellar setae: absent. Metascutellum sculpture: with large smooth posterior fovea. Postmarginal vein: absent. Fore wing apex at rest: reaching near apex of T5. Coxae color brightness: same color as femora. Spines along tibiae: absent. Lateral propodeal carinae: broadly separated, not parallel anteriorly. Setae in metasomal depression: absent. Anterior sculpture of metasomal depression: absent. Median propodeal carina: absent.

T1 horn: absent. Number of longitudinal carinae of T1 midlobe: 5. T1 lateral carina: protruding laterally, visible from ventral view. T2 sculpture: with longitudinal striae or rugae, setiferous puncta present between them. T2 sublateral longitudinal foveae: absent. T3 metasomal flanges: absent. T4 sculpture: longitudinally striate to rugose, setal pits spanning interspaces. T4 metasomal flanges: absent. T5 sculpture: longitudinally striate to rugose, setal pits spanning interspaces. T5 metasomal flanges: present as slightly protruding sharp corners. T6: broader than long. Major sculpture of T6: umbilicate punctate. Microsculpture of T6: absent. T6 medially: with medially truncate emargination, sloping down to apical rim. T6 metasomal flanges: present as spine-like structures posterolaterally. T6 raised peripheral rim: absent. S4 sculpture: densely setose, setal pits between very weak longitudinal rugae. S5 sculpture: densely setose, setal pits between very weak longitudinal rugae. S5 median carina: present. S6 peripheral carina: absent. S6 apex in relation to T6: exposed to dorsal view by T6 emargination. S6 apex: truncate.

*Male*. unknown.

##### Diagnosis.

Both sexes: Frontal depression shallow, without carinae ventrally, dorsal separator interrupted medially, weakly indicated carinae present above dorsal separator; submedian carina absent. Hyperoccipital carina indicated by vague rugae. Occipital carina complete, medially sinuate. Mesoscutellar rim not expanded, without median notch. Metascutellum small, weakly emarginate apically, projecting dorsally. Coxa same color as rest of leg. Postmarginal vein absent. Female: A4, A5 broader than long. T4, T5 without distinct metasomal flanges, but T5 with sharp posterior corners. T6 abruptly narrower than T5, without expanded lateral margins but with narrow and sharp elongate posterior metasomal flanges. Main surface of T6 strongly emarginate medially, sloping down to apical rim. S6 exposed to dorsal view, rounded apically. It differs from *Oxyscelio livens* and similar species in that T6 is abruptly narrower than T5, which itself has sharp corners that may represent tiny metasomal flanges.

##### Etymology.

Latin noun, genitive case, meaning “tails.”

##### Link to distribution map.

[http://hol.osu.edu/map-full.html?id=307066]

##### Material examined.

Holotype, female: AUSTRALIA: QLD, open forest, 3.4km S Port Douglas, 17.XI.1979, E. C. Dahms, J. B. Woolley & J. LaSalle, OSUC 437874 (deposited in QMBA). Paratypes: AUSTRALIA: 3 females, OSUC 437873, 437876 (ANIC); OSUC 437875 (WINC).

#### Oxyscelio circulorum

Burks sp. n.

http://zoobank.org/5BB83EC9-B496-4FCF-8A8F-B79A42A5C3C6

urn:lsid:biosci.ohio-state.edu:osuc_concepts:307124

http://species-id.net/wiki/Oxyscelio_circulorum

**Figures 37–42.**
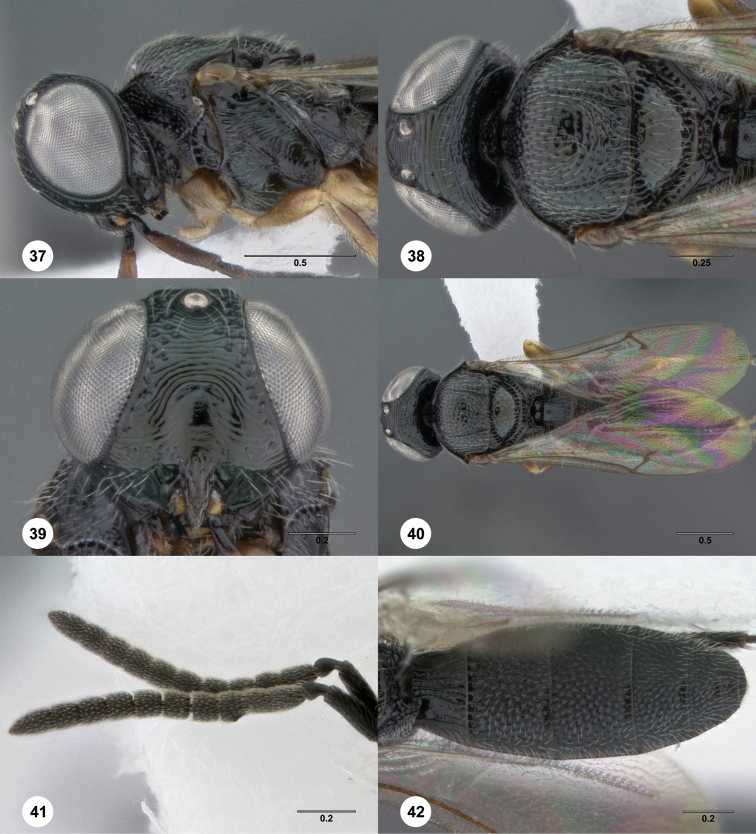
*Oxyscelio circulorum* sp. n., holotype female (OSUC 225606) **37** Head and mesosoma, lateral view **38** Head and mesosoma, dorsal view **39** Head, anterior view **40** Metasoma, dorsal view. Paratype male (OSUC 148409) **41** Antenna **42** Metasoma, dorsal view. Morphbank^34^

Figures 37–42 ; Morphbank^34^

##### Description.

Female. Body length 3.25 mm (n=1).

Radicle color and shade: darker than scape. Pedicel color: same as scape. A3: longer than pedicel. A4: longer than broad. A5: longer than broad.

Ventral clypeal margin: concave. Interantennal process: not elongate. Lower frons at dorsal margin of interantennal process: with transverse ledge, face sharply receding below it. Transverse curved rugae extending from frontal depression to eye: present. Median longitudinal carina in frontal depression: absent. Ventral portion of frontal depression: with medially interrupted transverse carinae. Dorsal portion of frontal depression: without transverse carinae. Submedian carina: present. Frontal depression dorsally: not hood-like, open dorsally. Upper frons major sculpture: umbilicate foveate; transversely rugose. Upper frons microsculpture: absent. Hyperoccipital carina: present as multiple regular elevations. Carina connecting occipital carina to hyperoccipital carina: absent. Occipital carina: present laterally, absent medially. Occiput sculpture: irregularly sculptured. Extra carina ventral to occipital carina: absent. Gena length: shorter than eye. Major sculpture of gena anteroventrally: umbilicate punctate; absent. Major sculpture of gena posteroventrally: umbilicate punctate; absent. Microsculpture of gena anteroventrally: absent; granulate. Microsculpture of gena posteroventrally: absent.

Lateral pronotal area sculpture: anteriorly smooth, posterodorsal corner with dense microsculpture, ventral corner with irregular carinae. Posterior border of central pronotal area: directed posteriorly, epomial carina absent or meeting transverse pronotal carina at arch on lateral surface of pronotum. Mesoscutum anteriorly: very steep and tall, descending at a right angle or protruding anteriorly. Major sculpture of mesoscutal midlobe anteriorly: umbilicate foveate. Mesoscutal midlobe sculpture at midlength: with narrow curved smooth elevations. Major sculpture of mesoscutal midlobe posteriorly: umbilicate foveate; transversely rugose. Microsculpture of mesoscutal midlobe anteriorly: granulate. Microsculpture of mesoscutal midlobe posteriorly: absent. Median mesoscutal carina: absent. Major sculpture of mesoscutellum centrally: umbilicate punctate. Major sculpture of mesoscutellum peripherally: umbilicate punctate. Microsculpture of mesoscutellum centrally: absent. Microsculpture of mesoscutellum peripherally: absent. Mesoscutellar rim: not expanded. Mesoscutellar rim medially: without notch. Mesofemoral depression: longitudinally striate dorsally, smooth ventrally. Metascutellum shape: slightly emarginate posteriorly, concave but elevated posteriorly. Metascutellar setae: absent. Metascutellum sculpture: with a median carina, otherwise weakly sculptured. Postmarginal vein: present. Fore wing apex at rest: exceeding metasomal apex. Coxae color brightness: same color as femora. Spines along tibiae: absent. Lateral propodeal carinae: broadly separated, not parallel anteriorly. Setae in metasomal depression: absent. Anterior sculpture of metasomal depression: absent. Median propodeal carina: absent.

T1 horn: present. Number of longitudinal carinae of T1 midlobe: obscured by other raised sculpture. T1 lateral carina: straight. T2 sculpture: densely foveolate, longitudinal sculpture irregular. T2 sublateral longitudinal foveae: absent. T3 metasomal flanges: absent. T4 sculpture: longitudinally striate to rugose, setal pits spanning interspaces. T4 metasomal flanges: absent. T5 sculpture: longitudinally striate to rugose, setal pits spanning interspaces. T5 metasomal flanges: absent. T6: broader than long. Major sculpture of T6: umbilicate punctate. Microsculpture of T6: absent. T6 medially: flat and tapering to a rounded apex, not separated from apical rim. T6 metasomal flanges: absent. T6 raised peripheral rim: absent. S4 sculpture: longitudinally striate or rugose, setal pits spanning interspaces. S5 sculpture: longitudinally striate to rugose, setal pits spanning interspaces. S5 median carina: absent. S6 peripheral carina: absent. S6 apex in relation to T6: not exposed to dorsal view. S6 apex: rounded or acuminate.

*Male*. Body length 2.85–3 mm (n=3). A3: longer than pedicel. A5 tyloid shape: narrow, linear. A6: broader than long. A11: longer than broad. Major sculpture of mesoscutal midlobe anteriorly: umbilicate foveate; transversely rugose. Major sculpture of mesoscutal midlobe posteriorly: umbilicate foveate; irregularly rugose. Microsculpture of mesoscutal midlobe anteriorly: granulate. Microsculpture of mesoscutal midlobe posteriorly: absent. Major sculpture of mesoscutellum centrally: umbilicate punctate. Major sculpture of mesoscutellum peripherally: umbilicate punctate. Microsculpture of mesoscutellum centrally: granulate. Microsculpture of mesoscutellum peripherally: granulate. Fore wing apex at rest: exceeding metasomal apex. T1 midlobe longitudinal carinae: 6 or more. T3 metasomal flanges: absent. T4 metasomal flanges: absent. T5 metasomal flanges: absent. T6 metasomal flanges: absent. T7: truncate.

##### Diagnosis.

Both sexes: Frontal depression nearly flat, surrounded by many transverse curved rugae; submedian carina indicated by a set of rugae. Hyperoccipital carina indicated by many transverse carinae. Occipital carina incomplete, lateral portions approaching hyperoccipital carina. Occiput irregularly sculptured. Metascutellum broad and very short, concave, with a distinct median carina. Postmarginal vein present. Coxa and rest of leg bicolored. T1 lateral carina not expanded laterally. Metasomal flanges absent. Female: A3 longer than pedicel. A4, A5 longer than broad. Mesoscutellum with setiferous puncta. T1 midlobe with very weak anterior horn, no complete longitudinal carinae. Fore wing long enough to reach beyond metasomal apex. T6 broader than long. Male: Fore wing long enough to exceed metasomal apex. T7 tiny, truncate. *Oxyscelio circulorum* is similar to *Oxyscelio tubi*, but has a much shorter T1 horn and metasoma.

##### Etymology.

Latin noun, genitive case, meaning “rings.”

##### Link to distribution map.

[http://hol.osu.edu/map-full.html?id=307124]

##### Material examined.

Holotype, female: NEW CALEDONIA: Nord Prov., 5km WSW Pouebo, Mount Mandjelia, 20.397°S, 164.528°E, 780m, 9.XII-15.XII.2000, malaise trap, M. E. Irwin, OSUC 225606 (deposited in MNHN). Paratypes: NEW CALEDONIA: 2 females, 1 male, OSUC 77540-77541 (OSUC); OSUC 148409 (QMBA).

#### Oxyscelio clivi

Burks sp. n.

http://zoobank.org/65B2B16D-4156-42EF-9E1F-A81D628C9B11

urn:lsid:biosci.ohio-state.edu:osuc_concepts:307067

http://species-id.net/wiki/Oxyscelio_clivi

**Figures 43–46.**
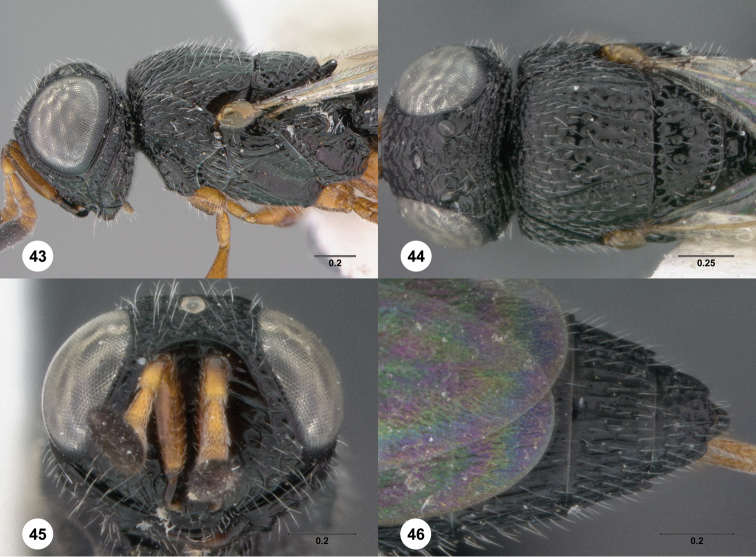
*Oxyscelio clivi* sp. n., holotype female (OSUC 359627) **43** Head and mesosoma, lateral view **44** Head and mesosoma, dorsal view. Paratype female (OSUC 462732) **45** Head, anterior view **46** Metasomal apex, dorsal view. Morphbank^35^

Figures 43–46 ; Morphbank^35^

##### Description.

Female. Body length 3.3–4.75 mm (n=20).

Radicle color and shade: darker than scape. Pedicel color: at least partially darker than scape. A3: longer than pedicel. A4: broader than long. A5: broader than long.

Ventral clypeal margin: uniformly convex. Interantennal process: not elongate. Lower frons at dorsal margin of interantennal process: without transverse carina. Transverse curved rugae extending from frontal depression to eye: absent. Median longitudinal carina in frontal depression: absent. Ventral portion of frontal depression: with medially interrupted transverse carinae. Dorsal portion of frontal depression: without transverse carinae. Submedian carina: present. Frontal depression dorsally: not hood-like, open dorsally. Upper frons major sculpture: umbilicate foveate; irregularly rugose. Upper frons microsculpture: absent. Hyperoccipital carina: indicated by a set of irregular elevations. Carina connecting occipital carina to hyperoccipital carina: absent. Occipital carina: broadly angular, with rounded median peak. Occiput sculpture: irregularly sculptured. Extra carina ventral to occipital carina: present, complete. Gena length: shorter than eye. Major sculpture of gena anteroventrally: umbilicate foveate. Major sculpture of gena posteroventrally: umbilicate foveate; rugose. Microsculpture of gena anteroventrally: absent. Microsculpture of gena posteroventrally: absent; granulate.

Lateral pronotal area sculpture: anteriorly smooth, posterodorsal corner with dense microsculpture, ventral corner with irregular carinae. Posterior border of central pronotal area: directed anteriorly, protruding at corner of epomial carina and transverse pronotal carina. Mesoscutum anteriorly: very steep and tall, descending at a right angle or protruding anteriorly. Major sculpture of mesoscutal midlobe anteriorly: umbilicate foveate. Mesoscutal midlobe sculpture at midlength: not different from nearby sculpture. Major sculpture of mesoscutal midlobe posteriorly: umbilicate foveate; longitudinally rugose. Microsculpture of mesoscutal midlobe anteriorly: granulate. Microsculpture of mesoscutal midlobe posteriorly: absent. Median mesoscutal carina: present as a ruga. Major sculpture of mesoscutellum centrally: umbilicate foveate. Major sculpture of mesoscutellum peripherally: umbilicate foveate. Microsculpture of mesoscutellum centrally: punctate. Microsculpture of mesoscutellum peripherally: punctate. Mesoscutellar rim: not expanded. Mesoscutellar rim medially: without notch. Mesofemoral depression: longitudinally striate dorsally, smooth ventrally. Metascutellum shape: slightly emarginate posteriorly, concave but elevated posteriorly. Metascutellar setae: absent. Metascutellum sculpture: with large smooth posterior fovea. Postmarginal vein: absent. Fore wing apex at rest: reaching base of T5. Coxae color brightness: same color as femora. Spines along tibiae: absent. Lateral propodeal carinae: broadly separated, not parallel anteriorly. Setae in metasomal depression: absent. Anterior sculpture of metasomal depression: absent. Median propodeal carina: absent.

T1 horn: absent. Number of longitudinal carinae of T1 midlobe: 4. T1 lateral carina: protruding laterally, visible from ventral view. T2 sculpture: with longitudinal striae or rugae, setiferous puncta present between them. T2 sublateral longitudinal foveae: absent. T3 metasomal flanges: absent. T4 sculpture: longitudinally striate to rugose, setal pits spanning interspaces. T4 metasomal flanges: present as slightly protruding sharp corners. T5 sculpture: longitudinally striate to rugose, setal pits spanning interspaces. T5 metasomal flanges: present as strongly protruding acuminate flanges. T6: broader than long. Major sculpture of T6: longitudinally striate; umbilicate foveate. Microsculpture of T6: absent. T6 medially: with a median projection set off by an abrupt narrowing posterior to tiny metasomal flanges, not separated from apical rim. T6 metasomal flanges: present subapically. T6 raised peripheral rim: absent. S4 sculpture: longitudinally striate or rugose, setal pits spanning interspaces. S5 sculpture: longitudinally striate to rugose, setal pits spanning interspaces. S5 median carina: present. S6 peripheral carina: absent. S6 apex in relation to T6: not exposed to dorsal view. S6 apex: rounded or acuminate.

*Male*. unknown.

##### Diagnosis.

Both sexes: Frontal depression deep and broad, carinae not present above dorsal separator; submedian carina distinct, sharp. Hyperoccipital carina indicated by vague rugae. Occipital carina complete, medially weakly convex. Metascutellum concave dorsally, weakly emarginate apically, projecting dorsally. Coxa color variable. Postmarginal vein absent. Female: A3 longer than pedicel. A4, A5 broader than long. T1 midlobe with 5 longitudinal carinae or obscured by raised sculpture. T4 with or without small metasomal flanges, T5 with metasomal flanges, not otherwise abruptly narrower than preceding tergum. Main body of T6 raised above apical rim, but sloping down to it medially, not abruptly separated from it. S6 not exposed to dorsal view.

##### Etymology.

Latin noun, genitive case, meaning “slope.”

##### Link to distribution map.

[http://hol.osu.edu/map-full.html?id=307067]

##### Material examined.

Holotype, female: AUSTRALIA: SA, 79km NNW Renmark, Calperum Station, Bookmark Biosphere Reserve, 33°31’S, 140°24’E, 12.XII-25.I.1996, flight intercept trap/gutter trap, K. R. Pullen, OSUC 359627 (deposited in ANIC). Paratypes: AUSTRALIA: 61 females, OSUC 359639, 359642, 359648-359657, 359659, 359661-359666, 359668-359671, 437857, 448612-448615 (ANIC); OSUC 462732-462736 (CNCI); OSUC 218838-218839 (INHS); OSUC 359667 (MVMA); OSUC 359640-359641, 359658 (QDPC); OSUC 148357, 148379, 359660, QM Reg. No. T35147 (QMBA); OSUC 448611 (QPIM); OSUC 359638 (UQIC); OSUC 268194 (USNM); OSUC 359633-359637, 359644-359647, 436997, 449092-449093, 449101, 453940-453941 (WINC).

##### Comments.

*Oxyscelio clivi* may represent a complex of sibling species, but these would be separated only by problematic, overlapping features. Based on current information, it seems best to consider this to be one species with some variable features. There is some variation in the T6 metasomal flanges, which may be broad and semi-translucent or narrower and opaque. The narrow T4 metasomal flanges may be present or absent. Specimens with dark coxae and antennae were considered to be a melanistic form.

#### Oxyscelio clupei

Burks sp. n.

http://zoobank.org/5877D8C2-5A16-4204-9798-F7A2860CCCFF

urn:lsid:biosci.ohio-state.edu:osuc_concepts:307068

http://species-id.net/wiki/Oxyscelio_clupei

**Figures 47–50.**
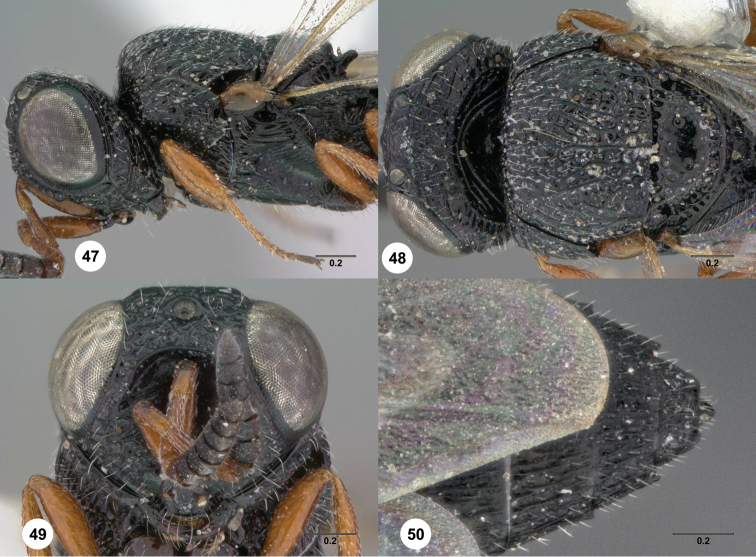
*Oxyscelio clupei* sp. n., paratype female (OSUC 359628) **47** Head and mesosoma, lateral view **48** Head and mesosoma, dorsal view **49** Head, anterior view **50** Metasoma, dorsal view. Morphbank^36^

Figures 47–50 ; Morphbank^36^

##### Description.

Female. Body length 3.7–4.15 mm (n=7).

Radicle color and shade: darker than scape. Pedicel color: same as scape. A3: longer than pedicel. A4: broader than long. A5: broader than long.

Ventral clypeal margin: with slightly convex median lobe. Interantennal process: not elongate. Lower frons at dorsal margin of interantennal process: without transverse carina. Transverse curved rugae extending from frontal depression to eye: absent. Median longitudinal carina in frontal depression: absent. Ventral portion of frontal depression: with medially interrupted transverse carinae. Dorsal portion of frontal depression: without transverse carinae. Submedian carina: present. Frontal depression dorsally: not hood-like, open dorsally. Upper frons major sculpture: umbilicate foveate; irregularly rugose. Upper frons microsculpture: absent. Hyperoccipital carina: indicated by a set of irregular elevations. Carina connecting occipital carina to hyperoccipital carina: absent. Occipital carina: weakly arched dorsally, with rounded lateral corners. Occiput sculpture: irregularly sculptured. Extra carina ventral to occipital carina: present, complete. Gena length: shorter than eye. Major sculpture of gena anteroventrally: umbilicate foveate. Major sculpture of gena posteroventrally: umbilicate foveate; rugose. Microsculpture of gena anteroventrally: absent. Microsculpture of gena posteroventrally: granulate.

Lateral pronotal area sculpture: anteriorly smooth, posterodorsal corner with dense microsculpture, ventral corner with irregular carinae. Posterior border of central pronotal area: directed anteriorly, protruding at corner of epomial carina and transverse pronotal carina. Mesoscutum anteriorly: very steep and tall, descending at a right angle or protruding anteriorly. Major sculpture of mesoscutal midlobe anteriorly: umbilicate foveate; longitudinally rugose. Mesoscutal midlobe sculpture at midlength: not different from nearby sculpture. Major sculpture of mesoscutal midlobe posteriorly: umbilicate foveate; longitudinally rugose. Microsculpture of mesoscutal midlobe anteriorly: granulate. Microsculpture of mesoscutal midlobe posteriorly: absent. Median mesoscutal carina: present as a ruga. Major sculpture of mesoscutellum centrally: absent; umbilicate foveate. Major sculpture of mesoscutellum peripherally: umbilicate foveate. Microsculpture of mesoscutellum centrally: absent; punctate. Microsculpture of mesoscutellum peripherally: punctate. Mesoscutellar rim: not expanded. Mesoscutellar rim medially: without notch. Mesofemoral depression: longitudinally striate dorsally, smooth ventrally. Metascutellum shape: slightly emarginate posteriorly, concave but elevated posteriorly. Metascutellar setae: absent. Metascutellum sculpture: with large smooth posterior fovea. Postmarginal vein: absent. Fore wing apex at rest: reaching base of T5. Coxae color brightness: darker than femora. Spines along tibiae: absent. Lateral propodeal carinae: broadly separated, not parallel anteriorly. Setae in metasomal depression: absent. Anterior sculpture of metasomal depression: absent. Median propodeal carina: absent.

T1 horn: absent. Number of longitudinal carinae of T1 midlobe: 4. T1 lateral carina: protruding laterally, visible from ventral view. T2 sculpture: with longitudinal striae or rugae, setiferous puncta present between them. T2 sublateral longitudinal foveae: absent. T3 metasomal flanges: absent. T4 sculpture: longitudinally striate to rugose, setal pits spanning interspaces. T4 metasomal flanges: present as slightly protruding sharp corners. T5 sculpture: longitudinally striate to rugose, setal pits spanning interspaces. T5 metasomal flanges: present as strongly protruding acuminate flanges. T6: broader than long. Major sculpture of T6: umbilicate punctate; longitudinally striate. Microsculpture of T6: absent. T6 medially: with a median projection set off by an abrupt narrowing posterior to tiny metasomal flanges, not separated from apical rim. T6 metasomal flanges: present subapically. T6 raised peripheral rim: absent. S4 sculpture: longitudinally striate or rugose, setal pits spanning interspaces. S5 sculpture: longitudinally striate to rugose, setal pits spanning interspaces. S5 median carina: present. S6 peripheral carina: absent. S6 apex in relation to T6: not exposed to dorsal view. S6 apex: rounded or acuminate.

*Male*. unknown.

##### Diagnosis.

Both sexes: Frontal depression deep and broad, carinae not present above dorsal separator; submedian carina distinct, sharp. Hyperoccipital carina indicated by strong rugae. Occipital carina complete, medially weakly convex. Metascutellum concave dorsally, weakly emarginate apically, projecting dorsally. Coxa darker than rest of leg. Postmarginal vein absent. Female: A3 longer than pedicel. A4, A5 broader than long. T4, T5 with sharp metasomal flanges, not otherwise abruptly narrower than preceding tergum. Main body of T6 raised above apical rim, but sloping down to it medially, not abruptly separated from it; T6 apex forming a trident-like shape. S6 not exposed to dorsal view.

##### Etymology.

Latin noun, genitive case, meaning “bronze shield.”

##### Link to distribution map.

[http://hol.osu.edu/map-full.html?id=307068]

##### Material examined.

Holotype, female: AUSTRALIA: NSW, Fowlers Gap Research Station, 31°05'S, 141°42'E, 29.XI–2.XII.1981, J. C. Cardale, OSUC 359631 (deposited in ANIC). Paratypes: AUSTRALIA: 6 females, OSUC 359630, 359632 (ANIC); OSUC 376681 (MCZC); OSUC 359628-359629, 449091 (WINC).

#### Oxyscelio concoloripes

(Dodd)

http://zoobank.org/CE4C149E-1454-49F8-B79E-600A6FEA5958

urn:lsid:biosci.ohio-state.edu:osuc_concepts:5011

http://species-id.net/wiki/Oxyscelio_concoloripes

**Figures 51–56.**
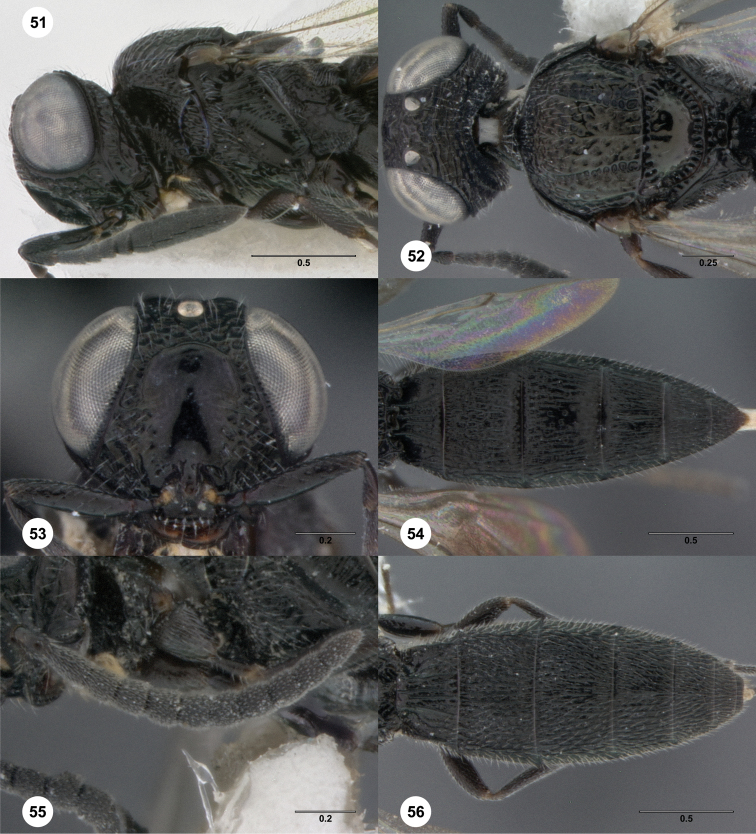
*Oxyscelio concoloripes* (Dodd), female (OSUC 439387) **51** Head and mesosoma, lateral view. Female (OSUC 439399) **52** Head and mesosoma, dorsal view **53** Head, anterior view **54** Metasoma, dorsal view. Male (OSUC 439527) **55** Antenna **56** Metasoma, dorsal view. Morphbank^37^

Figures 51–56 ; Morphbank^37^

Sceliomorpha concoloripes Dodd, 1914: 104 (original description); Kieffer 1926: 303, 308 (description, keyed); Naumann et al. 1994: 71 (holotype transferred to ANIC).
Oxyscelio concoloripes (Dodd): Dodd 1931: 75 (generic transfer); Galloway 1976: 99 (type information).

##### Description.

Female. Body length 3.55–4.15 mm (n=20).

Radicle color and shade: same as scape, both dark brown. Pedicel color: same as scape. A3: shorter than pedicel; as long as pedicel. A4: broader than long. A5: broader than long.

Ventral clypeal margin: with slightly convex median lobe. Interantennal process: not elongate. Lower frons at dorsal margin of interantennal process: without transverse carina. Transverse curved rugae extending from frontal depression to eye: absent. Median longitudinal carina in frontal depression: absent. Ventral portion of frontal depression: smooth. Dorsal portion of frontal depression: without transverse carinae. Submedian carina: present. Frontal depression dorsally: not hood-like, open dorsally. Upper frons major sculpture: umbilicate foveate; irregularly rugose. Upper frons microsculpture: absent. Hyperoccipital carina: indicated by a set of irregular elevations. Carina connecting occipital carina to hyperoccipital carina: absent. Occipital carina: uniformly rounded dorsally; weakly arched dorsally, with rounded lateral corners. Occiput sculpture: transversely rugose. Extra carina ventral to occipital carina: absent. Gena length: shorter than eye. Major sculpture of gena anteroventrally: umbilicate foveate. Major sculpture of gena posteroventrally: umbilicate punctate; absent. Microsculpture of gena anteroventrally: absent. Microsculpture of gena posteroventrally: granulate.

Lateral pronotal area sculpture: anteriorly smooth, posterodorsal corner with dense microsculpture, ventral corner with irregular carinae. Posterior border of central pronotal area: directed posteriorly, epomial carina absent or meeting transverse pronotal carina at arch on lateral surface of pronotum. Mesoscutum anteriorly: very steep and tall, descending at a right angle or protruding anteriorly. Major sculpture of mesoscutal midlobe anteriorly: umbilicate foveate. Mesoscutal midlobe sculpture at midlength: not different from nearby sculpture. Major sculpture of mesoscutal midlobe posteriorly: umbilicate foveate; irregularly rugose. Microsculpture of mesoscutal midlobe anteriorly: absent. Microsculpture of mesoscutal midlobe posteriorly: absent. Median mesoscutal carina: present as a ruga. Major sculpture of mesoscutellum centrally: absent; umbilicate punctate. Major sculpture of mesoscutellum peripherally: umbilicate foveate; umbilicate punctate. Microsculpture of mesoscutellum centrally: absent. Microsculpture of mesoscutellum peripherally: absent. Mesoscutellar rim: not expanded. Mesoscutellar rim medially: without notch. Mesofemoral depression: longitudinally striate dorsally, smooth ventrally. Metascutellum shape: slightly emarginate posteriorly, concave but elevated posteriorly. Metascutellar setae: absent. Metascutellum sculpture: with large smooth posterior fovea. Spines along tibiae: absent. Lateral propodeal carinae: broadly separated, but parallel for a short distance anteriorly. Setae in metasomal depression: absent. Anterior sculpture of metasomal depression: absent. Median propodeal carina: absent. Postmarginal vein: present. Fore wing apex at rest: reaching middle of T6. Coxae color brightness: same color as femora.

T1 horn: absent. Number of longitudinal carinae of T1 midlobe: 5; obscured by other raised sculpture. T1 lateral carina: straight. T2 sublateral longitudinal foveae: absent. T3 metasomal flanges: absent. T4 sculpture: longitudinally striate to rugose, setal pits spanning interspaces. T4 metasomal flanges: absent. T5 sculpture: longitudinally striate to rugose, setal pits spanning interspaces. T5 metasomal flanges: absent. T6: broader than long. Major sculpture of T6: umbilicate punctate; longitudinally striate. Microsculpture of T6: absent. T6 medially: flat and tapering to a rounded apex, not separated from apical rim. T6 metasomal flanges: absent. T6 raised peripheral rim: absent. S4 sculpture: longitudinally striate or rugose, setal pits spanning interspaces. S5 sculpture: longitudinally striate to rugose, setal pits spanning interspaces. S5 median carina: present. S6 peripheral carina: absent. S6 apex in relation to T6: not exposed to dorsal view. S6 apex: rounded or acuminate.

*Male*. Body length 3.2–3.65 mm (n=20). A3: longer than pedicel. A5 tyloid shape: narrow, linear. A6: broader than long. A11: broader than long; as long as broad. Major sculpture of mesoscutal midlobe anteriorly: umbilicate foveate. Major sculpture of mesoscutal midlobe posteriorly: umbilicate foveate; irregularly rugose. Microsculpture of mesoscutal midlobe anteriorly: granulate. Microsculpture of mesoscutal midlobe posteriorly: absent. Major sculpture of mesoscutellum centrally: absent. Major sculpture of mesoscutellum peripherally: umbilicate foveate. Microsculpture of mesoscutellum centrally: absent. Microsculpture of mesoscutellum peripherally: absent. Fore wing apex at rest: exceeding metasomal apex. T1 midlobe longitudinal carinae: 3; 4. T3 metasomal flanges: absent. T4 metasomal flanges: absent. T5 metasomal flanges: absent. T6 metasomal flanges: absent. T7: weakly emarginate.

##### Diagnosis.

Both sexes: Body, including antennae and legs, entirely dark brown. Frontal depression small and nearly flat, transverse carinae absent; submedian carina indicated by weak rugae. Hyperoccipital carina indicated by one or more sharp rugae. Occipital carina incomplete, weakly convex medially; occiput with many transverse rugae. Metascutellum deeply concave, emarginate apically, projecting dorsally. Postmarginal vein absent or extremely short. T1 lateral carina not expanded laterally. Female: A3 longer than pedicel. A4, A5 broader than long. T1 midlobe with 5 longitudinal carinae that may be obscured by a smooth raised area. Fore wing long enough to reach middle of T6 or beyond metasomal apex. T6 broader than long. Male: A4, A11 about as long as broad. T1 midlobe with 4 longitudinal carinae. Fore wing long enough to reach beyond T7. T7 truncate or weakly concave.

##### Link to distribution map.

[http://hol.osu.edu/map-full.html?id=5011]

##### Material examined.

Holotype, female, S. concoloripes: AUSTRALIA: NSW, no date, ANIC DB 32-012463 (deposited in ANIC). Other material: AUSTRALIA: 76 females, 99 males, OSUC 438799-438808, 438811-438814, 439384-439387, 439389-439395, 439409-439442, 439445-439481, 439483-439485, 445326 (ANIC); OSUC 462587-462589 (CNCI); OSUC 438810, 439388, 439396-439408, 439443-439444, 439482, 439486-439539 (WINC).

##### Comments.

Our concept of this species suggests that it is variable in female metasomal length. The holotype has a shorter metasoma, in which T6 is broader than long, but some specimens have a longer metasoma, where T6 is longer than broad. Specimens of both forms have been found from the same collection events, and some specimens appear to be intermediate between the two states. Several other Australian species are entirely dark brown, but these species differ in many other respects, and do not appear to form a monophyletic group.

#### Oxyscelio conjuncti

Burks sp. n.

http://zoobank.org/F30ABCD6-76FB-49CF-A574-E84DBD3ED93F

urn:lsid:biosci.ohio-state.edu:osuc_concepts:307069

http://species-id.net/wiki/Oxyscelio_conjuncti

**Figures 57–62.**
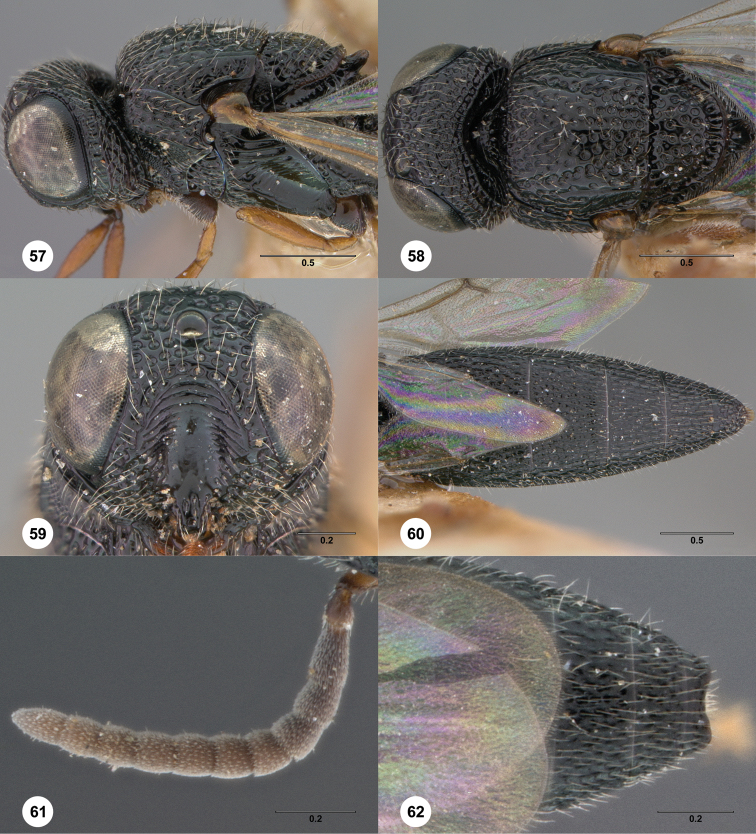
*Oxyscelio conjuncti* sp. n., paratype female (OSUC 429810) **57** Head and mesosoma, lateral view **58** Head and mesosoma, dorsal view **59** Head, anterior view **60** Metasoma, dorsal view. Paratype male (OSUC 429872) **61** Antenna **62** Metasoma, dorsal view. Morphbank^38^

Figures 57–62 ; Morphbank^38^

##### Description.

Female. Body length 3.35–4.85 mm (n=20).

Radicle color and shade: darker than scape. Pedicel color: same as scape. A3: longer than pedicel. A4: longer than broad; as long as broad. A5: broader than long.

Ventral clypeal margin: with slightly convex median lobe. Interantennal process: not elongate. Lower frons at dorsal margin of interantennal process: without transverse carina. Transverse curved rugae extending from frontal depression to eye: absent. Median longitudinal carina in frontal depression: absent. Ventral portion of frontal depression: with medially interrupted transverse carinae. Dorsal portion of frontal depression: with medially interrupted transverse carinae. Submedian carina: present only as a weak shift in elevation. Frontal depression dorsally: not hood-like, open dorsally. Upper frons major sculpture: umbilicate foveate; transversely rugose. Upper frons microsculpture: granulate. Hyperoccipital carina: absent. Carina connecting occipital carina to hyperoccipital carina: absent. Occipital carina: weakly arched dorsally, with rounded lateral corners. Occiput sculpture: irregularly sculptured. Extra carina ventral to occipital carina: absent. Gena length: shorter than eye. Major sculpture of gena anteroventrally: umbilicate foveate; rugose. Major sculpture of gena posteroventrally: umbilicate foveate; rugose. Microsculpture of gena anteroventrally: absent. Microsculpture of gena posteroventrally: absent.

Lateral pronotal area sculpture: anteriorly smooth, posterodorsal corner with dense microsculpture, ventral corner with irregular carinae. Posterior border of central pronotal area: directed anteriorly, protruding at corner of epomial carina and transverse pronotal carina. Mesoscutum anteriorly: very steep and tall, descending at a right angle or protruding anteriorly. Major sculpture of mesoscutal midlobe anteriorly: umbilicate foveate. Mesoscutal midlobe sculpture at midlength: not different from nearby sculpture. Major sculpture of mesoscutal midlobe posteriorly: umbilicate foveate; longitudinally rugose. Microsculpture of mesoscutal midlobe anteriorly: absent; granulate. Microsculpture of mesoscutal midlobe posteriorly: absent. Median mesoscutal carina: present as a vague, occasionally interrupted elevation. Major sculpture of mesoscutellum centrally: umbilicate foveate. Major sculpture of mesoscutellum peripherally: umbilicate foveate. Microsculpture of mesoscutellum centrally: absent. Microsculpture of mesoscutellum peripherally: absent. Mesoscutellar rim: not expanded. Mesoscutellar rim medially: without notch. Mesofemoral depression: longitudinally striate dorsally, smooth ventrally. Metascutellum shape: slightly emarginate posteriorly, concave but elevated posteriorly. Metascutellar setae: absent. Metascutellum sculpture: with large smooth posterior fovea. Postmarginal vein: absent. Fore wing apex at rest: reaching middle of T5. Coxae color brightness: darker than femora. Spines along tibiae: absent. Lateral propodeal carinae: broadly separated, not parallel anteriorly. Setae in metasomal depression: absent. Anterior sculpture of metasomal depression: absent. Median propodeal carina: absent.

T1 horn: absent. Number of longitudinal carinae of T1 midlobe: 4. T1 lateral carina: protruding laterally, visible from ventral view. T2 sculpture: with longitudinal striae or rugae, setiferous puncta present between them. T2 sublateral longitudinal foveae: absent. T3 metasomal flanges: absent. T4 sculpture: longitudinally striate to rugose, setal pits spanning interspaces. T4 metasomal flanges: absent. T5 sculpture: longitudinally striate to rugose, setal pits spanning interspaces. T5 metasomal flanges: absent. T6: broader than long. Major sculpture of T6: umbilicate foveate. Microsculpture of T6: absent. T6 medially: flat and tapering to a rounded apex, not separated from apical rim. T6 metasomal flanges: absent. T6 raised peripheral rim: absent. S4 sculpture: longitudinally striate or rugose, setal pits spanning interspaces. S5 sculpture: longitudinally striate to rugose, setal pits spanning interspaces. S5 median carina: present. S6 peripheral carina: absent. S6 apex in relation to T6: not exposed to dorsal view. S6 apex: rounded or acuminate.

*Male*. Body length 3.55–4.05 mm (n=6).

A3: longer than pedicel. A5 tyloid shape: narrow, linear. A6: broader than long. A11: longer than broad. Major sculpture of mesoscutal midlobe anteriorly: umbilicate foveate. Major sculpture of mesoscutal midlobe posteriorly: umbilicate foveate. Microsculpture of mesoscutal midlobe anteriorly: granulate. Microsculpture of mesoscutal midlobe posteriorly: absent. Major sculpture of mesoscutellum centrally: umbilicate foveate. Major sculpture of mesoscutellum peripherally: umbilicate foveate. Microsculpture of mesoscutellum centrally: absent. Microsculpture of mesoscutellum peripherally: absent. Fore wing apex at rest: reaching middle of T5. T1 midlobe longitudinal carinae: 4. T3 metasomal flanges: absent. T4 metasomal flanges: absent. T5 metasomal flanges: absent. T6 metasomal flanges: absent. T7: broadly and deeply emarginate, with rounded posterolateral margins.

##### Diagnosis.

Both sexes: Frontal depression deep and broad but nearly parallel-sided, many interrupted transverse carinae present, including some above dorsal separator; submedian carina weakly indicated or absent medially. Hyperoccipital carina indicated by weak rugae. Occipital carina complete, sinuate medially. Metascutellum deeply concave, weakly emarginate apically, projecting dorsally. Postmarginal vein absent. Coxa darker than rest of leg. T1 lateral carina expanded laterally. Metasomal flanges absent. Female: A3 much longer than pedicel. A4 about as long as broad. Mesoscutellum without granulate sculpture. T1 midlobe with 4 longitudinal carinae. T6 without metasomal flanges, main body of tergum raised but not always clearly separated from apical rim. Fore wing long enough to reach middle of T4 or middle of T5. Male: A4 as broad or broader than long, A11 longer than broad. T1 midlobe with 4 longitudinal carinae. Fore wing long enough to reach middle of T5. T7 medially emarginate, with rounded lobes laterally.

**Etymology**. Latin noun, genitive case, meaning “connection.”

##### Link to distribution map.

[http://hol.osu.edu/map-full.html?id=307069]

##### Material examined.

Holotype, female: AUSTRALIA: QLD, 5729 ‘amphitheatre’ yards, open forest, Expedition National Park, 25°13’S, 149°01’E, 440m, 19.XII-4.III.1998, interception trap, Cook & Monteith, OSUC 429869 (deposited in QMBA). Paratypes: AUSTRALIA: 71 females, 6 males, ANIC DB 32-020081, 32-020083, 32-020084, 32-020085, OSUC 429809, OSUC 429810, OSUC 429812, OSUC 429813, OSUC 429814, OSUC 429815, OSUC 429816, OSUC 429818, OSUC 429819, OSUC 429820, OSUC 429821, OSUC 429822, OSUC 429823, OSUC 429824, OSUC 429825, OSUC 429826, OSUC 429827, OSUC 429828, OSUC 429829, OSUC 429830, OSUC 429831, OSUC 429832, OSUC 429833, OSUC 429834, OSUC 429835, OSUC 429836, OSUC 429837, OSUC 429838, OSUC 429839, OSUC 429840, OSUC 429841, OSUC 429842, OSUC 429843, OSUC 429844, OSUC 429845, OSUC 429846, OSUC 429847, OSUC 429848, OSUC 429849, OSUC 429850, OSUC 429855, OSUC 429856, OSUC 429857, OSUC 429858, OSUC 429859, OSUC 429860, OSUC 429861, OSUC 429862, OSUC 429863, OSUC 429864, OSUC 429865, OSUC 429866, OSUC 429867, OSUC 429870, OSUC 429871, OSUC 429872, OSUC 429873, OSUC 429874, OSUC 429875, OSUC 448633 (ANIC); OSUC 451354 (BMNH); OSUC 462753 (CNCI); OSUC 429851-429854, 429868, 449023-449025, 456183-456185 (WINC).

#### Oxyscelio contusionis

Burks sp. n.

http://zoobank.org/82C78BC3-EDDB-46B6-917C-FBF1FC564E4D

urn:lsid:biosci.ohio-state.edu:osuc_concepts:307070

http://species-id.net/wiki/Oxyscelio_contusionis

**Figures 63–66.**
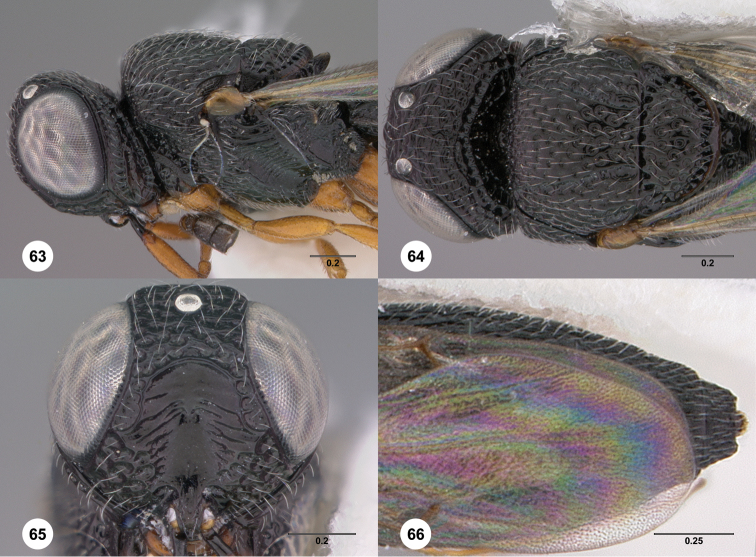
*Oxyscelio contusionis* sp. n., holotype female (ANIC DB 32-020142) **63** Head and mesosoma, lateral view **64** Head and mesosoma, dorsal view **65** Head, anterior view **66** Metasoma, dorsal view. Morphbank^39^

Figures 63–66 ; Morphbank^39^

##### Description.

Female. Body length 2.95–3.95 mm (n=20).

Radicle color and shade: same as scape, both yellowish or reddish. Pedicel color: same as scape. A3: as long as pedicel. A4: broader than long. A5: broader than long.

Ventral clypeal margin: uniformly convex. Interantennal process: not elongate. Lower frons at dorsal margin of interantennal process: without transverse carina. Transverse curved rugae extending from frontal depression to eye: absent. Median longitudinal carina in frontal depression: absent. Ventral portion of frontal depression: with medially interrupted transverse carinae. Dorsal portion of frontal depression: without transverse carinae. Submedian carina: present. Frontal depression dorsally: not hood-like, open dorsally. Upper frons major sculpture: umbilicate foveate; irregularly rugose. Upper frons microsculpture: absent. Hyperoccipital carina: indicated by a set of irregular elevations. Carina connecting occipital carina to hyperoccipital carina: absent. Occipital carina: weakly arched dorsally, with rounded lateral corners. Occiput sculpture: irregularly sculptured. Extra carina ventral to occipital carina: present, complete. Gena length: shorter than eye. Major sculpture of gena anteroventrally: umbilicate foveate. Major sculpture of gena posteroventrally: umbilicate foveate. Microsculpture of gena anteroventrally: absent. Microsculpture of gena posteroventrally: absent.

Lateral pronotal area sculpture: with shallow irregular carinae, posterodorsal corner with dense microsculpture. Posterior border of central pronotal area: directed anteriorly, protruding at corner of epomial carina and transverse pronotal carina. Mesoscutum anteriorly: very steep and tall, descending at a right angle or protruding anteriorly. Major sculpture of mesoscutal midlobe anteriorly: umbilicate foveate. Mesoscutal midlobe sculpture at midlength: not different from nearby sculpture. Major sculpture of mesoscutal midlobe posteriorly: umbilicate foveate. Microsculpture of mesoscutal midlobe anteriorly: granulate. Microsculpture of mesoscutal midlobe posteriorly: absent. Median mesoscutal carina: present as a vague, occasionally interrupted elevation. Major sculpture of mesoscutellum centrally: umbilicate foveate. Major sculpture of mesoscutellum peripherally: umbilicate foveate. Microsculpture of mesoscutellum centrally: absent. Microsculpture of mesoscutellum peripherally: absent. Mesoscutellar rim: not expanded. Mesoscutellar rim medially: without notch. Mesofemoral depression: longitudinally striate dorsally, smooth ventrally. Metascutellum shape: slightly emarginate posteriorly, concave but elevated posteriorly. Metascutellar setae: absent. Metascutellum sculpture: with large smooth posterior fovea. Postmarginal vein: absent. Fore wing apex at rest: reaching middle of T5. Coxae color brightness: same color as femora. Spines along tibiae: absent. Lateral propodeal carinae: broadly separated, not parallel anteriorly. Setae in metasomal depression: absent. Anterior sculpture of metasomal depression: absent. Median propodeal carina: absent.

T1 horn: absent. Number of longitudinal carinae of T1 midlobe: 4. T1 lateral carina: protruding laterally, visible from ventral view. T2 sculpture: with longitudinal striae or rugae, setiferous puncta present between them. T2 sublateral longitudinal foveae: absent. T3 metasomal flanges: absent. T4 sculpture: longitudinally striate to rugose, setal pits spanning interspaces. T4 metasomal flanges: present as slightly protruding sharp corners. T5 sculpture: longitudinally striate to rugose, setal pits spanning interspaces. T5 metasomal flanges: present as strong posterior corners. T6: broader than long. Major sculpture of T6: umbilicate punctate. Microsculpture of T6: absent. T6 medially: with deep emargination that is V-shaped medially, separated from apical rim. T6 metasomal flanges: present as slightly expanded lateral rims, rounded posteriorly. T6 raised peripheral rim: absent. S4 sculpture: longitudinally striate or rugose, setal pits spanning interspaces. S5 sculpture: longitudinally striate to rugose, setal pits spanning interspaces. S5 median carina: present. S6 peripheral carina: absent. S6 apex in relation to T6: not exposed to dorsal view. S6 apex: rounded or acuminate.

*Male*. unknown.

##### Diagnosis.

Both sexes: Frontal depression deep, with interrupted carinae ventrally, dorsal separator complete medially, weakly indicated carinae present above dorsal separator; submedian carina distinct but not strongly projecting. Hyperoccipital carina indicated by vague rugae. Occipital carina complete, medially weakly convex. Mesoscutellar rim not expanded, without median notch. Metascutellum small, weakly emarginate apically, projecting dorsally. Coxa same color as rest of leg. Postmarginal vein absent. Female: A3 longer than pedicel. A4, A5 broader than long. T4, T5 without distinct metasomal flanges. T6 abruptly narrower than T5, without expanded lateral margins. Main surface of T6 strongly emarginate medially, with sharp posterior corners, sharply separated from apical rim. S6 exposed to dorsal view, rounded apically.

##### Etymology.

Latin noun, genitive case, meaning “contusion.”

##### Link to distribution map.

[http://hol.osu.edu/map-full.html?id=307070]

##### Associations.

Collected on *Eucalyptus* L’Hér.: [Myrtales: Myrtaceae]; collected on ironbark: [Myrtales: Myrtaceae]

##### Material examined.

Holotype, female: AUSTRALIA: QLD, Batavia Downs, 12°40'S, 142°39'E, 22.VI–23.VIII.1992, flight intercept trap, P. Zborowski & J. C. Cardale, ANIC DB 32-020142 (deposited in ANIC). Paratypes: AUSTRALIA: 27 females, OSUC 359672-359679, 436940-436942, 436945, 436995, 454000 (ANIC); OSUC 449094-449095 (BMNH); OSUC 462729-462731 (CNCI); OSUC 359681, QM Reg. No. T35149 (QMBA); OSUC 436944, 436996 (QPIM); OSUC 359680 (UQIC); OSUC 436943, 453944-453945 (WINC).

#### Oxyscelio corrugationis

Burks sp. n.

http://zoobank.org/5625556F-F8EB-4AF3-B09D-7FF7C20CC298

urn:lsid:biosci.ohio-state.edu:osuc_concepts:307071

http://species-id.net/wiki/Oxyscelio_corrugationis

**Figures 67–70.**
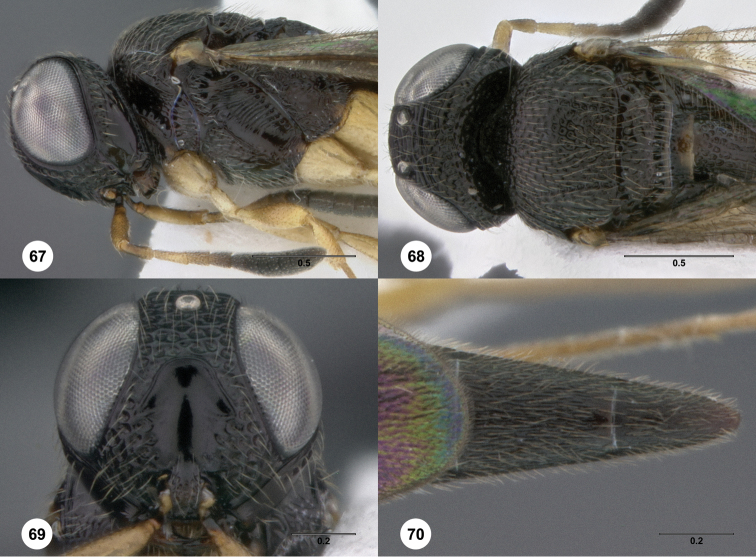
*Oxyscelio corrugationis* sp. n., female (OSUC 368229) **67** Head and mesosoma, lateral view **68** Head and mesosoma, dorsal view **69** Head, anterior view **70** Metasoma, dorsal view. Morphbank^40^

Figures 67–70 ; Morphbank^40^

##### Description.

Female. Body length 4.3–4.85 mm (n=7).

Radicle color and shade: darker than scape. Pedicel color: at least partially darker than scape. A3: longer than pedicel. A4: longer than broad. A5: longer than broad.

Ventral clypeal margin: concave. Interantennal process: not elongate. Lower frons at dorsal margin of interantennal process: without transverse carina. Transverse curved rugae extending from frontal depression to eye: absent. Median longitudinal carina in frontal depression: absent. Ventral portion of frontal depression: smooth. Dorsal portion of frontal depression: without transverse carinae. Submedian carina: present. Frontal depression dorsally: not hood-like, open dorsally. Upper frons major sculpture: umbilicate foveate; transversely rugose. Upper frons microsculpture: absent. Hyperoccipital carina: indicated by a set of irregular elevations. Carina connecting occipital carina to hyperoccipital carina: absent. Occipital carina: present laterally, absent medially. Occiput sculpture: irregularly sculptured. Extra carina ventral to occipital carina: absent. Gena length: shorter than eye. Major sculpture of gena anteroventrally: umbilicate foveate. Major sculpture of gena posteroventrally: umbilicate punctate. Microsculpture of gena anteroventrally: absent. Microsculpture of gena posteroventrally: granulate.

Lateral pronotal area sculpture: mostly granulate, ventral corner with irregular carinae. Posterior border of central pronotal area: directed posteriorly, epomial carina absent or meeting transverse pronotal carina at arch on lateral surface of pronotum. Mesoscutum anteriorly: not steep, forming less than a right angle. Major sculpture of mesoscutal midlobe anteriorly: umbilicate foveate. Mesoscutal midlobe sculpture at midlength: not different from nearby sculpture. Major sculpture of mesoscutal midlobe posteriorly: umbilicate foveate. Microsculpture of mesoscutal midlobe anteriorly: absent. Microsculpture of mesoscutal midlobe posteriorly: absent. Median mesoscutal carina: absent. Major sculpture of mesoscutellum centrally: umbilicate foveate. Major sculpture of mesoscutellum peripherally: umbilicate foveate. Microsculpture of mesoscutellum centrally: absent. Microsculpture of mesoscutellum peripherally: absent. Mesoscutellar rim: not expanded. Mesoscutellar rim medially: without notch. Mesofemoral depression: longitudinally striate dorsally and ventrally. Metascutellum shape: not emarginate, convex dorsally. Metascutellar setae: absent. Metascutellum sculpture: with many longitudinal rugae. Postmarginal vein: present. Fore wing apex at rest: reaching base of T5. Coxae color brightness: same color as femora. Spines along tibiae: absent. Lateral propodeal carinae: broadly separated, not parallel anteriorly. Setae in metasomal depression: absent. Anterior sculpture of metasomal depression: absent. Median propodeal carina: absent.

T1 horn: present. Number of longitudinal carinae of T1 midlobe: obscured by other raised sculpture. T1 lateral carina: straight. T2 sculpture: with longitudinal striae or rugae, setiferous puncta present between them. T2 sublateral longitudinal foveae: absent. T3 metasomal flanges: absent. T4 sculpture: longitudinally striate to rugose, setal pits spanning interspaces. T4 metasomal flanges: absent. T5 sculpture: longitudinally striate to rugose, setal pits spanning interspaces. T5 metasomal flanges: absent. T6: longer than broad. Major sculpture of T6: umbilicate punctate; longitudinally striate. Microsculpture of T6: absent. T6 medially: flat and tapering to a rounded apex, not separated from apical rim. T6 metasomal flanges: absent. T6 raised peripheral rim: absent. S4 sculpture: longitudinally striate or rugose, setal pits spanning interspaces. S5 sculpture: longitudinally striate to rugose, setal pits spanning interspaces. S5 median carina: present. S6 peripheral carina: absent. S6 apex in relation to T6: not exposed to dorsal view. S6 apex: rounded or acuminate.

*Male*. unknown.

##### Diagnosis.

Both sexes: Frontal depression shallow, transverse carinae absent; submedian carina sharp. Hyperoccipital carina indicated by rugae. Occipital carina absent medially, lateral corners not protruding. Postmarginal vein present. Coxa not darker than rest of leg. T1 lateral carina not expanded laterally. Metasomal flanges absent. Female: A4 longer than broad, A5 about as long as broad. Mesoscutellum without granulate sculpture. Metascutellum broad and short, slightly convex, irregularly rugose. Fore wing long enough to reach base of T5. T6 longer than broad.

##### Etymology.

Latin noun, genitive case, meaning “corrugation.”

##### Link to distribution map.

[http://hol.osu.edu/map-full.html?id=307071]

##### Material examined.

Holotype, female: AUSTRALIA: QLD, GS2, Mount Edith, 17°06'S, 145°38'E, 1050m, 3.I–4.II.1995, malaise trap, P. Zborowski, OSUC 368229 (deposited in ANIC). Paratypes: AUSTRALIA: 6 females, OSUC 368228 (ANIC); OSUC 227543, 227555-227556, 462578 (CNCI); QDPC 0-165741 (QDPC).

#### Oxyscelio croci

Burks sp. n.

http://zoobank.org/B74702BA-2E4A-4640-ABC4-321CA7ABF807

urn:lsid:biosci.ohio-state.edu:osuc_concepts:307072

http://species-id.net/wiki/Oxyscelio_croci

**Figures 71–76.**
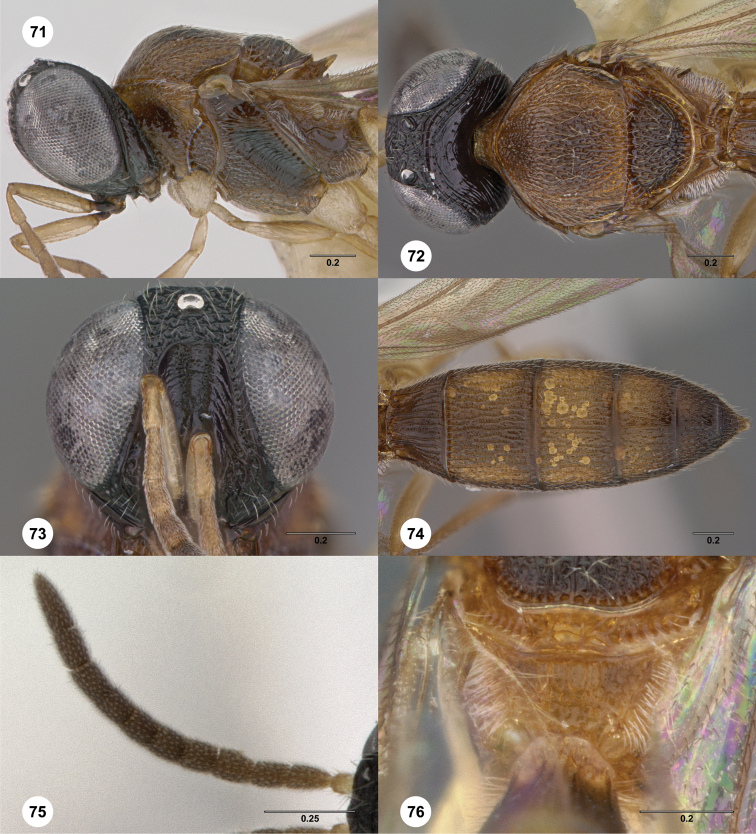
*Oxyscelio croci* sp. n., paratype female (OSUC 442324) **71** Head and mesosoma, lateral view **72** Head and mesosoma, dorsal view **73** Head, anterior view **74** Metasoma, dorsal view. Paratype male (OSUC 442329) **75** Antenna **76** Mesosoma, dorsal view. Morphbank^41^

Figures 71–76 ; Morphbank^41^

##### Description.

Female. Body length 2.9–3.55 mm (n=19).

Radicle color and shade: darker than scape. Pedicel color: same as scape. A3: longer than pedicel. A4: longer than broad. A5: longer than broad.

Ventral clypeal margin: concave. Interantennal process: not elongate. Lower frons at dorsal margin of interantennal process: without transverse carina. Transverse curved rugae extending from frontal depression to eye: absent. Median longitudinal carina in frontal depression: absent. Ventral portion of frontal depression: smooth. Dorsal portion of frontal depression: without transverse carinae. Submedian carina: present. Frontal depression dorsally: not hood-like, open dorsally. Upper frons major sculpture: umbilicate foveate; transversely rugose. Upper frons microsculpture: granulate. Hyperoccipital carina: present as a single carina. Carina connecting occipital carina to hyperoccipital carina: absent. Occipital carina: present laterally, absent medially. Occiput sculpture: transversely rugose. Extra carina ventral to occipital carina: present, complete. Gena length: shorter than eye. Major sculpture of gena anteroventrally: umbilicate punctate. Major sculpture of gena posteroventrally: umbilicate punctate. Microsculpture of gena anteroventrally: granulate. Microsculpture of gena posteroventrally: granulate.

Lateral pronotal area sculpture: irregularly sculptured. Posterior border of central pronotal area: directed posteriorly, epomial carina absent or meeting transverse pronotal carina at arch on lateral surface of pronotum. Mesoscutum anteriorly: very steep and tall, descending at a right angle or protruding anteriorly. Major sculpture of mesoscutal midlobe anteriorly: umbilicate foveate; umbilicate punctate. Mesoscutal midlobe sculpture at midlength: not different from nearby sculpture. Major sculpture of mesoscutal midlobe posteriorly: umbilicate foveate; longitudinally rugose. Microsculpture of mesoscutal midlobe anteriorly: granulate. Microsculpture of mesoscutal midlobe posteriorly: absent. Median mesoscutal carina: present as a vague, occasionally interrupted elevation. Major sculpture of mesoscutellum centrally: umbilicate foveate; irregularly rugose. Major sculpture of mesoscutellum peripherally: umbilicate foveate; irregularly rugose. Microsculpture of mesoscutellum centrally: absent. Microsculpture of mesoscutellum peripherally: absent. Mesoscutellar rim: not expanded. Mesoscutellar rim medially: without notch. Mesofemoral depression: longitudinally striate dorsally and ventrally. Metascutellum shape: slightly emarginate posteriorly, concave but elevated posteriorly. Metascutellar setae: absent. Metascutellum sculpture: with large smooth posterior fovea. Postmarginal vein: present. Fore wing apex at rest: reaching middle of T6. Coxae color brightness: same color as femora. Spines along tibiae: absent. Lateral propodeal carinae: meeting near propodeal midlength. Setae in metasomal depression: absent. Anterior sculpture of metasomal depression: with series of longitudinal carinae extending to lateral propodeal carinae. Median propodeal carina: absent.

T1 horn: absent. Number of longitudinal carinae of T1 midlobe: 5. T1 lateral carina: straight. T2 sculpture: with longitudinal striae or rugae, setiferous puncta present between them. T2 sublateral longitudinal foveae: absent. T3 metasomal flanges: absent. T4 sculpture: longitudinally striate to rugose, setal pits spanning interspaces. T4 metasomal flanges: absent. T5 sculpture: longitudinally striate to rugose, setal pits spanning interspaces. T5 metasomal flanges: absent. T6: broader than long. Major sculpture of T6: umbilicate punctate. Microsculpture of T6: granulate. T6 medially: flat and tapering to a rounded apex, not separated from apical rim. T6 metasomal flanges: absent. T6 raised peripheral rim: absent. S4 sculpture: longitudinally striate or rugose, setal pits spanning interspaces. S5 sculpture: longitudinally striate to rugose, setal pits spanning interspaces. S5 median carina: present. S6 peripheral carina: absent. S6 apex in relation to T6: not exposed to dorsal view. S6 apex: rounded or acuminate.

*Male*. Body length 2.85–3.25 mm (n=20). A3: longer than pedicel. A5 tyloid shape: narrow, linear. A6: longer than broad. A11: longer than broad. Major sculpture of mesoscutal midlobe anteriorly: umbilicate foveate; umbilicate punctate. Major sculpture of mesoscutal midlobe posteriorly: umbilicate foveate. Microsculpture of mesoscutal midlobe anteriorly: granulate. Microsculpture of mesoscutal midlobe posteriorly: absent. Major sculpture of mesoscutellum centrally: umbilicate foveate; irregularly rugose. Major sculpture of mesoscutellum peripherally: umbilicate foveate; irregularly rugose. Microsculpture of mesoscutellum centrally: granulate. Microsculpture of mesoscutellum peripherally: granulate. Fore wing apex at rest: exceeding metasomal apex. T1 midlobe longitudinal carinae: 3; 4. T3 metasomal flanges: absent. T4 metasomal flanges: absent. T5 metasomal flanges: absent. T6 metasomal flanges: absent. T7: truncate.

##### Diagnosis.

Both sexes: Frontal depression nearly parallel-sided, transverse carinae complete but weakly indicated; submedian carina sharp or weak and irregular. Hyperoccipital carina sharp and strong. Occipital carina incomplete, lateral portions almost reaching hyperoccipital carina; occiput with many weak rugae and a row of strong setiferous puncta. Metascutellum with a concave postero-medial area, laterally with broad longitudinally striate area, weakly emarginate, projecting dorsally. Postmarginal vein present. Coxa not darker than rest of leg. T1 lateral carina not expanded laterally. Female: A3 longer than pedicel. A4 longer than broad, A5 as long or longer than broad. Mesoscutellum densely and coarsely sculptured. T1 midlobe with 5 longitudinal carinae. Fore wing long enough to reach or exceed T6. Male: All flagellomeres longer than broad. T1 midlobe with 3 or 4 longitudinal carinae, hardly raised above sidelobes. Mesoscutellum with strong irregular rugae and some granulate sculpture. Fore wing long enough to reach beyond T7. T7 truncate, steeply sloping. *Oxyscelio croci* is very similar to *Oxyscelio oculi*, differing in having stronger sculpture in females and in having more irregular mesoscutellar sculpture in males. These species have overlapping distributions, and males can be difficult to distinguish.

##### Etymology.

Latin noun, genitive case, meaning “saffron.”

##### Link to distribution map.

[http://hol.osu.edu/map-full.html?id=307072]

##### Material examined.

Holotype, female: AUSTRALIA: QLD, via Canungra, forest edge, O’Reilley’s Guest House, 3.II-2.III.1980, malaise trap, OSUC 442318 (deposited in QMBA). Paratypes: AUSTRALIA: 18 females, 23 males, OSUC 442321, 442340 (ANIC); OSUC 451285 (BMNH); OSUC 227541, 227610, 227628, 462567-462570 (CNCI); OSUC 442324, 442329, QDPC 0-165788 (QDPC); OSUC 442319 (QPIM); OSUC 442311-442317, 442320, 442323, 442325-442328, 442330-442339, 451284, 451286-451288 (WINC).

#### Oxyscelio cuspidis

Burks sp. n.

http://zoobank.org/A2AF97D1-9746-4013-9EE3-F4A8EC23D356

urn:lsid:biosci.ohio-state.edu:osuc_concepts:307073

http://species-id.net/wiki/Oxyscelio_cuspidis

**Figures 77–80.**
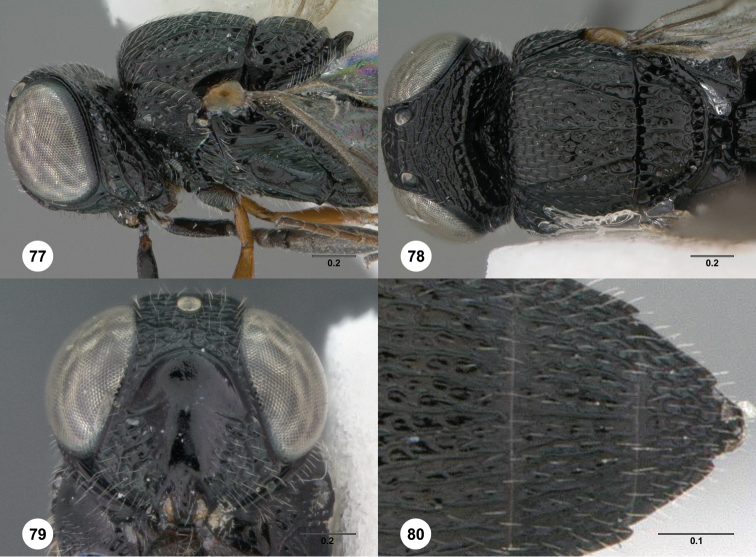
*Oxyscelio cuspidis* sp. n., holotype female (OSUC 439574) **77** Head and mesosoma, lateral view **78** Head and mesosoma, dorsal view **79** Head, anterior view **80** Metasoma, dorsal view. Morphbank^42^

Figures 77–80 ; Morphbank^42^

##### Description.

Female. Body length 3.2–3.95 mm (n=14).

Radicle color and shade: same as scape, both dark brown. Pedicel color: same as scape. A3: longer than pedicel. A4: broader than long. A5: broader than long.

Ventral clypeal margin: with slightly convex median lobe. Interantennal process: not elongate. Lower frons at dorsal margin of interantennal process: without transverse carina. Transverse curved rugae extending from frontal depression to eye: absent. Median longitudinal carina in frontal depression: absent. Ventral portion of frontal depression: smooth. Dorsal portion of frontal depression: without transverse carinae. Submedian carina: present. Frontal depression dorsally: not hood-like, open dorsally. Upper frons major sculpture: umbilicate foveate; transversely rugose. Upper frons microsculpture: granulate. Hyperoccipital carina: indicated by a set of irregular elevations. Carina connecting occipital carina to hyperoccipital carina: absent. Occipital carina: weakly arched dorsally, with rounded lateral corners. Occiput sculpture: irregularly sculptured. Extra carina ventral to occipital carina: present, medially incomplete. Gena length: shorter than eye. Major sculpture of gena anteroventrally: umbilicate foveate. Major sculpture of gena posteroventrally: umbilicate foveate; rugose. Microsculpture of gena anteroventrally: absent. Microsculpture of gena posteroventrally: granulate.

Lateral pronotal area sculpture: anteriorly smooth, posterodorsal corner with dense microsculpture, ventral corner with irregular carinae. Posterior border of central pronotal area: directed anteriorly, protruding at corner of epomial carina and transverse pronotal carina. Mesoscutum anteriorly: very steep and tall, descending at a right angle or protruding anteriorly. Major sculpture of mesoscutal midlobe anteriorly: umbilicate foveate. Mesoscutal midlobe sculpture at midlength: with large smooth areas. Major sculpture of mesoscutal midlobe posteriorly: umbilicate foveate; longitudinally rugose. Microsculpture of mesoscutal midlobe anteriorly: granulate. Microsculpture of mesoscutal midlobe posteriorly: absent. Median mesoscutal carina: present as a narrow carina; present as a flattened or rounded elevation. Major sculpture of mesoscutellum centrally: umbilicate foveate. Major sculpture of mesoscutellum peripherally: umbilicate foveate; irregularly rugose. Microsculpture of mesoscutellum centrally: absent. Microsculpture of mesoscutellum peripherally: absent. Mesoscutellar rim: not expanded. Mesoscutellar rim medially: without notch. Mesofemoral depression: longitudinally striate dorsally, smooth ventrally. Metascutellum shape: slightly emarginate posteriorly, concave but elevated posteriorly. Metascutellar setae: absent. Metascutellum sculpture: with large smooth posterior fovea. Postmarginal vein: absent. Fore wing apex at rest: reaching base of T5. Coxae color brightness: darker than femora. Spines along tibiae: absent. Lateral propodeal carinae: broadly separated, not parallel anteriorly. Setae in metasomal depression: absent. Anterior sculpture of metasomal depression: absent. Median propodeal carina: absent.

T1 horn: absent. Number of longitudinal carinae of T1 midlobe: 6. T1 lateral carina: protruding laterally, visible from ventral view. T2 sculpture: with longitudinal striae or rugae, setiferous puncta present between them. T2 sublateral longitudinal foveae: absent. T3 metasomal flanges: present. T4 sculpture: longitudinally striate to rugose, setal pits spanning interspaces. T4 metasomal flanges: present as slightly protruding sharp corners. T5 sculpture: longitudinally striate to rugose, setal pits spanning interspaces. T5 metasomal flanges: present as slightly protruding sharp corners. T6: broader than long. Major sculpture of T6: longitudinally striate; umbilicate foveate. Microsculpture of T6: absent. T6 medially: with a median projection set off by an abrupt narrowing posterior to tiny metasomal flanges, not separated from apical rim. T6 metasomal flanges: present subapically. T6 raised peripheral rim: absent. S4 sculpture: longitudinally striate or rugose, setal pits spanning interspaces. S5 sculpture: longitudinally striate to rugose, setal pits spanning interspaces. S5 median carina: present. S6 peripheral carina: absent. S6 apex in relation to T6: not exposed to dorsal view. S6 apex: truncate.

*Male*. unknown.

##### Diagnosis.

Both sexes: Frontal depression deep, carinae not present above dorsal separator; submedian carina distinct, sharp. Hyperoccipital carina indicated by rugae. Occipital carina complete, medially weakly convex. Metascutellum concave dorsally, weakly emarginate apically, projecting dorsally. Coxa darker than rest of leg. Postmarginal vein absent. Female: A3 longer than pedicel. A4, A5 broader than long. T4 with sharp corners, T5 with small sharp metasomal flanges, not otherwise abruptly narrower than preceding tergum. Main body of T6 not raised above apical rim, not abruptly separated from it; T6 with small and sharp subapical metasomal flanges. S6 not exposed to dorsal view.

##### Etymology.

Latin noun, genitive case, meaning “point.”

##### Link to distribution map.

[http://hol.osu.edu/map-full.html?id=307073]

##### Material examined.

Holotype, female: AUSTRALIA: QLD, Mount Moffatt Section, 3km SE park headquarters, Carnarvon National Park, 25°04'39"S, 148°00'30"E, 740m, 21.XI.1995, malaise trap, Irwin, Gaimari et al., OSUC 439574 (deposited in QMBA). Paratypes: AUSTRALIA: 13 females, ANIC DB 32-020111, 32-020112, 32-020113, OSUC 439570, OSUC 439571, OSUC 439572, OSUC 439573, OSUC 439577, OSUC 439578, OSUC 439579, OSUC 439580, OSUC 439581 (ANIC); OSUC 439575 (WINC).

##### Comments.

Females of *Oxyscelio cuspidis* are easily recognized by the convex and smoothly sloping T6 with subapical spine-like metasomal flanges. Males are currently unknown, but may be difficult to distinguish from those of other *atricoxa*-group species.

#### Oxyscelio densitatis

Burks sp. n.

http://zoobank.org/F8B54218-5049-4CF5-A40F-686E4741F351

urn:lsid:biosci.ohio-state.edu:osuc_concepts:307074

http://species-id.net/wiki/Oxyscelio_densitatis

**Figures 81–84.**
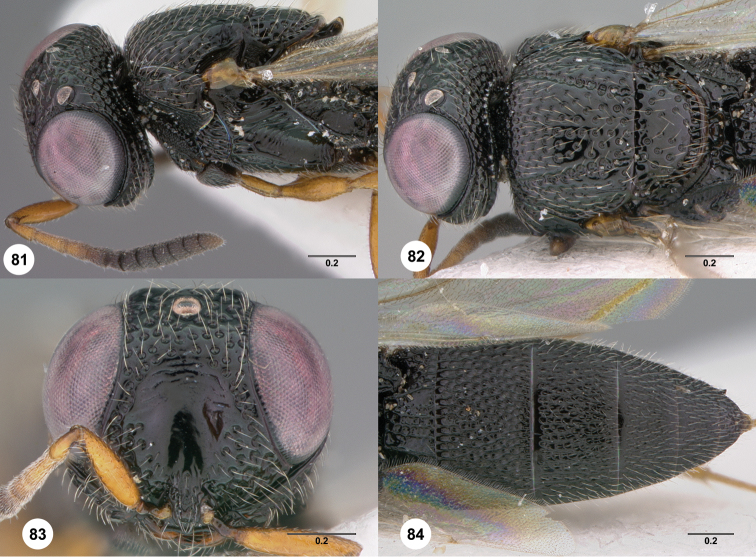
*Oxyscelio densitatis* sp. n., holotype female (QM Reg. No. T35158) **81** Head and mesosoma, lateral view **82** Head and mesosoma, dorsal view **83** Head, anterior view **84** Metasoma, dorsal view. Morphbank^43^

Figures 81–84 ; Morphbank^43^

##### Description.

Female. Body length 2.75–4 mm (n=20).

Radicle color and shade: darker than scape. Pedicel color: same as scape. A3: longer than pedicel. A4: longer than broad. A5: broader than long.

Ventral clypeal margin: with slightly convex median lobe. Interantennal process: not elongate. Lower frons at dorsal margin of interantennal process: without transverse carina. Transverse curved rugae extending from frontal depression to eye: absent. Median longitudinal carina in frontal depression: absent. Ventral portion of frontal depression: smooth. Dorsal portion of frontal depression: with medially interrupted transverse carinae. Submedian carina: present only as a weak shift in elevation. Frontal depression dorsally: not hood-like, open dorsally. Upper frons major sculpture: umbilicate foveate. Upper frons microsculpture: absent. Hyperoccipital carina: absent. Carina connecting occipital carina to hyperoccipital carina: absent. Occipital carina: weakly arched dorsally, with rounded lateral corners. Occiput sculpture: umbilicate foveate. Extra carina ventral to occipital carina: present, complete. Gena length: shorter than eye. Major sculpture of gena anteroventrally: umbilicate foveate; umbilicate punctate. Major sculpture of gena posteroventrally: umbilicate foveate; rugose. Microsculpture of gena anteroventrally: absent. Microsculpture of gena posteroventrally: absent.

Lateral pronotal area sculpture: anteriorly smooth, posterodorsal corner with dense microsculpture, ventral corner with irregular carinae. Posterior border of central pronotal area: directed anteriorly, protruding at corner of epomial carina and transverse pronotal carina. Mesoscutum anteriorly: very steep and tall, descending at a right angle or protruding anteriorly. Major sculpture of mesoscutal midlobe anteriorly: umbilicate foveate. Mesoscutal midlobe sculpture at midlength: not different from nearby sculpture. Major sculpture of mesoscutal midlobe posteriorly: umbilicate foveate; longitudinally rugose. Microsculpture of mesoscutal midlobe anteriorly: granulate. Microsculpture of mesoscutal midlobe posteriorly: absent. Median mesoscutal carina: present as a vague, occasionally interrupted elevation. Major sculpture of mesoscutellum centrally: umbilicate foveate. Major sculpture of mesoscutellum peripherally: umbilicate foveate. Microsculpture of mesoscutellum centrally: absent; punctate. Microsculpture of mesoscutellum peripherally: absent. Mesoscutellar rim: not expanded. Mesoscutellar rim medially: without notch. Mesofemoral depression: longitudinally striate dorsally, smooth ventrally. Metascutellum shape: deeply emarginate, with the resulting pair of posterior processes subtriangular and directed dorsally. Metascutellar setae: absent. Metascutellum sculpture: with large smooth posterior fovea. Postmarginal vein: absent. Fore wing apex at rest: reaching middle of T6. Coxae color brightness: darker than femora. Spines along tibiae: absent. Lateral propodeal carinae: broadly separated, not parallel anteriorly. Setae in metasomal depression: absent. Anterior sculpture of metasomal depression: absent. Median propodeal carina: absent.

T1 horn: absent. Number of longitudinal carinae of T1 midlobe: obscured by other raised sculpture. T1 lateral carina: protruding laterally, visible from ventral view. T2 sculpture: densely foveolate, longitudinal sculpture irregular. T2 sublateral longitudinal foveae: absent. T3 metasomal flanges: absent. T4 sculpture: longitudinally striate to rugose, setal pits spanning interspaces. T4 metasomal flanges: absent. T5 sculpture: longitudinally striate to rugose, setal pits spanning interspaces. T5 metasomal flanges: absent. T6: broader than long. Major sculpture of T6: umbilicate foveate. Microsculpture of T6: absent. T6 medially: flat and tapering to a rounded apex, not separated from apical rim. T6 metasomal flanges: absent. T6 raised peripheral rim: absent. S4 sculpture: longitudinally striate or rugose, setal pits spanning interspaces. S5 sculpture: longitudinally striate to rugose, setal pits spanning interspaces. S5 median carina: absent. S6 peripheral carina: absent. S6 apex in relation to T6: not exposed to dorsal view. S6 apex: rounded or acuminate.

*Male*. Unknown.

##### Diagnosis.

Both sexes: Frontal depression shallow, transverse carinae absent or broadly interrupted ventrally, with sometimes a few faint carinae dorsally; submedian carina absent medially. Hyperoccipital carina indicated by weak rugae. Occipital carina complete, weakly convex or omicron-shaped medially. Metascutellum deeply concave, truncate or weakly emarginate apically, projecting dorsally. Postmarginal vein absent. Coxa darker than rest of leg. T1 lateral carina expanded laterally. Metasomal flanges absent. Female: A3 longer than pedicel. A4 longer than broad, A5 nearly as long as broad. Mesoscutellum without granulate sculpture. T1 midlobe with 4 longitudinal carinae or with raised sculpture obscuring them. T6 without metasomal flanges, main body of tergum sloping down to apical rim. Fore wing long enough to reach middle of T5 or middle of T6. *Oxyscelio densitatis* is very similar to *Oxyscelio nigricoxa* and a few other species with dark coxae. It is larger-bodied than those species, and does not have a concave T6. Among larger-bodied species, it is similar to *Oxyscelio atricoxa*, but has an incomplete submedian carina and differently-shaped T6.

##### Etymology.

Latin noun, genitive case, meaning “density, abundance.” Refers to the abundance of mesoscutal midlobe foveae.

##### Link to distribution map.

[http://hol.osu.edu/map-full.html?id=307074]

##### Associations.

Inhabits brigalow: [Fabales: Fabaceae]

##### Material examined.

Holotype, female: AUSTRALIA: QLD, via Taroom, FIT 057, Boggomoss No. 30, 25°29'S, 150°08'E, 14-XI-1996 - I-1997, flight intercept trap, Cook & Monteith, QM Reg. No. T35158 (deposited in QMBA). Paratypes: AUSTRALIA: 36 females, ANIC DB 32-020090, 32-020094, 32-020095, OSUC 429972, OSUC 429976, OSUC 429980, OSUC 436978, OSUC 436980 (ANIC); OSUC 448944-448945 (BMNH); OSUC 227554 (CNCI); QDPC 0-165627, 0-165723, 0-165725 (QDPC); OSUC 148355, 148359, 148363, 429970, 429981, QM Reg. No. T35154, QM Reg. No. T35155, QM Reg. No. T35157 (QMBA); OSUC 429969, 429975 (UQIC); OSUC 429962-429968, 429971, 429973-429974, 429977-429979, 429982, 436979, 436981, 448940-448943 (WINC).

#### Oxyscelio dissimulationis

Burks sp. n.

http://zoobank.org/9829B8AA-2DC5-40ED-8F26-FBDF79F712A2

urn:lsid:biosci.ohio-state.edu:osuc_concepts:307075

http://species-id.net/wiki/Oxyscelio_dissimulationis

**Figures 85–90.**
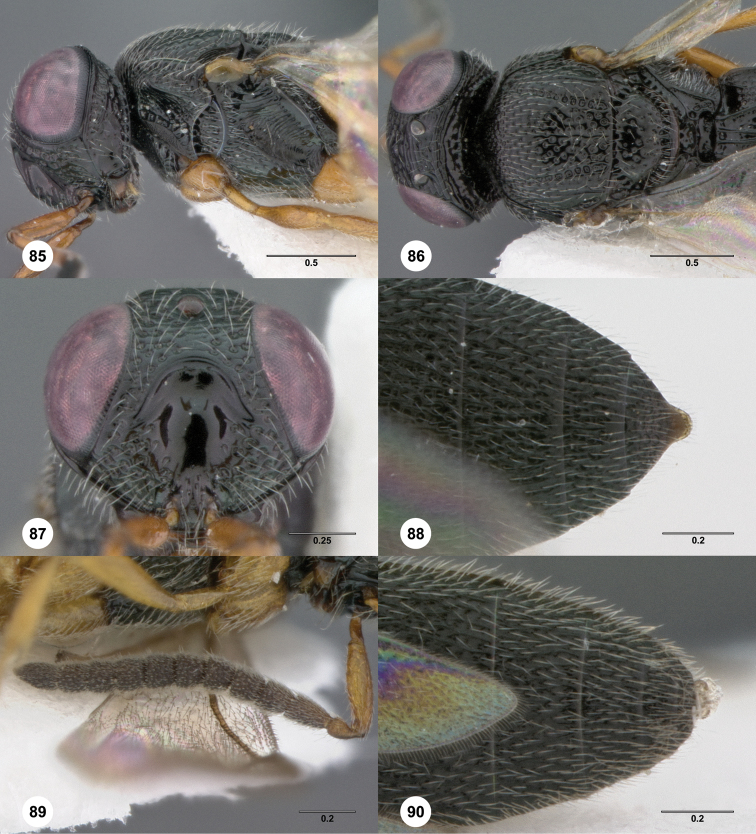
*Oxyscelio dissimulationis* sp. n., holotype female (QM Reg. No. T35159) **85** Head and mesosoma, lateral view **86** Head and mesosoma, dorsal view **87** Head, anterior view **88** Metasoma, dorsal view. Paratype male (OSUC 359737) **89** Antenna **90** Metasoma, dorsal view. Morphbank^44^

Figures 85–90 ; Morphbank^44^

##### Description.

Female. Body length 4–4.15 mm (n=2).

Radicle color and shade: same as scape, both yellowish or reddish. Pedicel color: same as scape. A3: longer than pedicel. A4: broader than long. A5: broader than long.

Ventral clypeal margin: concave. Interantennal process: not elongate. Lower frons at dorsal margin of interantennal process: without transverse carina. Transverse curved rugae extending from frontal depression to eye: absent. Median longitudinal carina in frontal depression: absent. Ventral portion of frontal depression: smooth. Dorsal portion of frontal depression: without transverse carinae. Submedian carina: present. Frontal depression dorsally: not hood-like, open dorsally. Upper frons major sculpture: umbilicate foveate; transversely rugose. Upper frons microsculpture: granulate. Hyperoccipital carina: present as a single carina. Carina connecting occipital carina to hyperoccipital carina: absent. Occipital carina: present laterally, absent medially. Occiput sculpture: transversely rugose; umbilicate punctate. Extra carina ventral to occipital carina: absent. Gena length: shorter than eye. Major sculpture of gena anteroventrally: umbilicate foveate. Major sculpture of gena posteroventrally: umbilicate foveate. Microsculpture of gena anteroventrally: absent. Microsculpture of gena posteroventrally: absent; punctate.

Lateral pronotal area sculpture: irregularly foveate, with smooth area dorsally. Posterior border of central pronotal area: directed posteriorly, epomial carina absent or meeting transverse pronotal carina at arch on lateral surface of pronotum. Mesoscutum anteriorly: very steep and tall, descending at a right angle or protruding anteriorly. Major sculpture of mesoscutal midlobe anteriorly: umbilicate foveate. Mesoscutal midlobe sculpture at midlength: not different from nearby sculpture. Major sculpture of mesoscutal midlobe posteriorly: umbilicate foveate; irregularly rugose. Microsculpture of mesoscutal midlobe anteriorly: granulate. Microsculpture of mesoscutal midlobe posteriorly: absent. Median mesoscutal carina: present as a vague, occasionally interrupted elevation. Major sculpture of mesoscutellum centrally: umbilicate foveate; umbilicate punctate. Major sculpture of mesoscutellum peripherally: umbilicate foveate. Microsculpture of mesoscutellum centrally: absent. Microsculpture of mesoscutellum peripherally: absent. Mesoscutellar rim: not expanded. Mesoscutellar rim medially: without notch. Mesofemoral depression: longitudinally striate dorsally and ventrally. Metascutellum shape: not emarginate, concave but elevated posteriorly. Metascutellar setae: absent. Metascutellum sculpture: with large smooth posterior fovea. Postmarginal vein: present. Fore wing apex at rest: reaching middle of T5; reaching near apex of T5. Coxae color brightness: same color as femora. Spines along tibiae: absent. Lateral propodeal carinae: narrowly separated, angled anteriorly to become parallel. Setae in metasomal depression: absent. Anterior sculpture of metasomal depression: with median areole or pair of pits. Median propodeal carina: absent.

T1 horn: absent. Number of longitudinal carinae of T1 midlobe: 4. T1 lateral carina: protruding laterally, visible from ventral view. T2 sculpture: with longitudinal striae or rugae, setiferous puncta present between them. T2 sublateral longitudinal foveae: absent. T3 metasomal flanges: absent. T4 sculpture: longitudinally striate to rugose, setal pits spanning interspaces. T4 metasomal flanges: absent. T5 sculpture: longitudinally striate to rugose, setal pits spanning interspaces. T5 metasomal flanges: absent. T6: broader than long. Major sculpture of T6: umbilicate punctate. Microsculpture of T6: absent. T6 medially: strongly convex, tapering and sloping down to a rounded apex, not separated from apical rim. T6 metasomal flanges: absent. T6 raised peripheral rim: absent. S4 sculpture: longitudinally striate or rugose, setal pits spanning interspaces. S5 sculpture: longitudinally striate to rugose, setal pits spanning interspaces. S5 median carina: present. S6 peripheral carina: absent. S6 apex in relation to T6: not exposed to dorsal view. S6 apex: rounded or acuminate.

*Male*. Body length 3.5–3.85 mm (n=2). A3: longer than pedicel. A5 tyloid shape: narrow, linear. A6: broader than long. A11: broader than long. Major sculpture of mesoscutal midlobe anteriorly: umbilicate foveate. Major sculpture of mesoscutal midlobe posteriorly: umbilicate foveate. Microsculpture of mesoscutal midlobe anteriorly: granulate. Microsculpture of mesoscutal midlobe posteriorly: absent. Major sculpture of mesoscutellum centrally: umbilicate foveate. Major sculpture of mesoscutellum peripherally: umbilicate foveate. Microsculpture of mesoscutellum centrally: absent. Microsculpture of mesoscutellum peripherally: absent. Fore wing apex at rest: exceeding metasomal apex. T1 midlobe longitudinal carinae: 4. T3 metasomal flanges: absent. T4 metasomal flanges: absent. T5 metasomal flanges: absent. T6 metasomal flanges: absent. T7: weakly emarginate.

##### Diagnosis.

Both sexes: Frontal depression deep and broad, transverse carinae interrupted or absent; submedian carina present. Hyperoccipital carina indicated by strong and sharp ruga. Occipital carina incomplete medially. Metascutellum deeply concave, not emarginate apically, projecting dorsally. Postmarginal vein present, short. Coxa not darker than rest of leg. T1 lateral carina expanded laterally. Metasomal flanges absent. Female: A3 longer than pedicel. A4, A5 much broader than long. Mesoscutellum without granulate sculpture. T1 midlobe with 4 longitudinal carinae or with these obscured by raised sculpture. T6 without metasomal flanges, not concave apically. Fore wing long enough to reach middle or apex of T5. Male: Flagellomeres between A3 and A11 broader than long. T1 midlobe with 4 longitudinal carinae. Fore wing long enough to reach to or beyond T7. T7 weakly emarginate, without projections.

##### Etymology.

Latin noun, genitive case, meaning “concealment.”

##### Link to distribution map.

[http://hol.osu.edu/map-full.html?id=307075]

##### Material examined.

Holotype, female: AUSTRALIA: QLD, via Taroom, FIT 057, Boggomoss No. 30, 25°29'S, 150°08'E, 14-XI-1996 - I-1997, flight intercept trap, Cook & Monteith, QM Reg. No. T35159 (deposited in QMBA). Paratypes: AUSTRALIA: 1 female, 2 males, OSUC 359737 (ANIC); OSUC 445333-445334 (WINC).

##### Comments.

*Oxyscelio dissimulationis* has a sharp hyperoccipital carina but medially interrupted occipital carina, as in the *flavipes*-group. The expanded lateral carina of T1 is like that found in the *atricoxa*-group. *Oxyscelio shakespearei* is similar in having the above combination of features, but lacks a postmarginal vein.

#### Oxyscelio divisionis

Burks sp. n.

http://zoobank.org/4C5D9FCC-E55D-488E-8B01-62D193CD24CA

urn:lsid:biosci.ohio-state.edu:osuc_concepts:307076

http://species-id.net/wiki/Oxyscelio_divisionis

**Figures 91–96.**
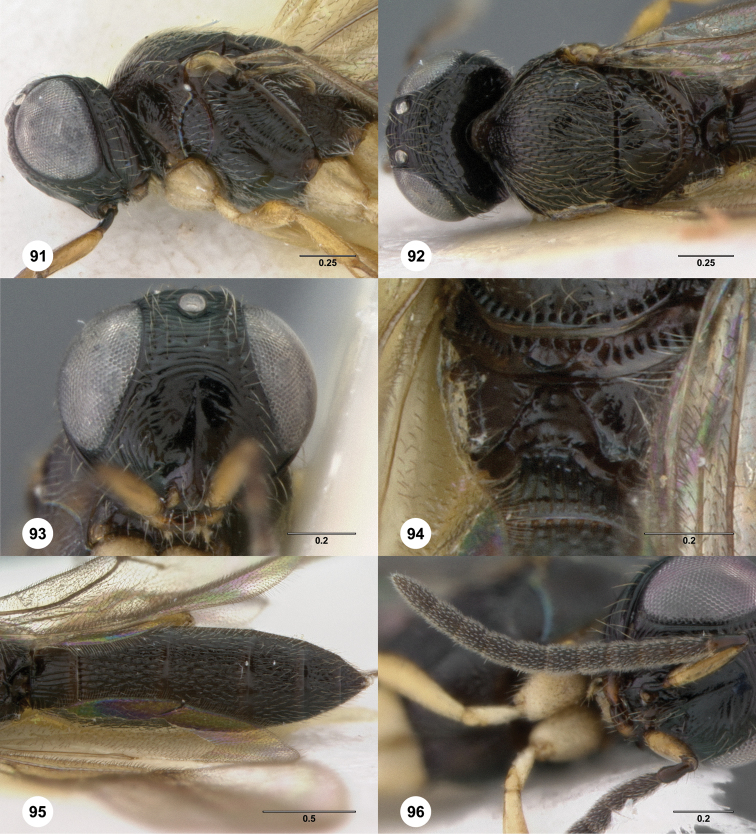
*Oxyscelio divisionis* sp. n., holotype female (OSUC 368204) **91** Head and mesosoma, lateral view **92** Head and mesosoma, dorsal view **93** Head, anterior view **94** Propodeum, posterodorsal view. Paratype male (OSUC 368205) **95** Antenna **96** Metasoma, dorsal view. Morphbank^45^

Figures 91–96 ; Morphbank^45^

##### Description.

Female. Body length 3.2–3.5 mm (n=11).

Radicle color and shade: darker than scape. Pedicel color: at least partially darker than scape. A3: shorter than pedicel. A4: longer than broad. A5: longer than broad; broader than long.

Ventral clypeal margin: concave. Interantennal process: not elongate. Lower frons at dorsal margin of interantennal process: without transverse carina. Transverse curved rugae extending from frontal depression to eye: absent. Median longitudinal carina in frontal depression: present. Ventral portion of frontal depression: with medially interrupted transverse carinae. Dorsal portion of frontal depression: with some transverse carinae. Submedian carina: absent. Frontal depression dorsally: not hood-like, open dorsally. Upper frons major sculpture: umbilicate foveate; transversely rugose. Upper frons microsculpture: absent. Hyperoccipital carina: present as a single carina. Carina connecting occipital carina to hyperoccipital carina: present. Occipital carina: present laterally, absent medially. Occiput sculpture: irregularly sculptured. Extra carina ventral to occipital carina: absent. Gena length: shorter than eye. Major sculpture of gena anteroventrally: rugose; umbilicate punctate. Major sculpture of gena posteroventrally: rugose; umbilicate punctate. Microsculpture of gena anteroventrally: absent. Microsculpture of gena posteroventrally: absent.

Lateral pronotal area sculpture: anteriorly smooth, posterodorsal corner with dense microsculpture, ventral corner with irregular carinae. Posterior border of central pronotal area: directed posteriorly, epomial carina absent or meeting transverse pronotal carina at arch on lateral surface of pronotum. Mesoscutum anteriorly: not steep, forming less than a right angle. Major sculpture of mesoscutal midlobe anteriorly: umbilicate foveate. Mesoscutal midlobe sculpture at midlength: not different from nearby sculpture. Major sculpture of mesoscutal midlobe posteriorly: umbilicate foveate; longitudinally rugose. Microsculpture of mesoscutal midlobe anteriorly: granulate. Microsculpture of mesoscutal midlobe posteriorly: absent. Median mesoscutal carina: present as a ruga. Major sculpture of mesoscutellum centrally: umbilicate punctate. Major sculpture of mesoscutellum peripherally: umbilicate punctate. Microsculpture of mesoscutellum centrally: absent. Microsculpture of mesoscutellum peripherally: absent. Mesoscutellar rim: not expanded. Mesoscutellar rim medially: without notch. Mesofemoral depression: longitudinally striate dorsally and ventrally. Metascutellum shape: slightly emarginate posteriorly, concave but elevated posteriorly. Metascutellar setae: absent. Metascutellum sculpture: with large smooth posterior fovea. Postmarginal vein: present. Fore wing apex at rest: exceeding metasomal apex. Coxae color brightness: same color as femora. Spines along tibiae: absent. Lateral propodeal carinae: broadly separated, not parallel anteriorly. Setae in metasomal depression: absent. Anterior sculpture of metasomal depression: absent. Median propodeal carina: absent.

T1 horn: absent. Number of longitudinal carinae of T1 midlobe: 5; 6. T1 lateral carina: straight. T2 sculpture: with longitudinal striae or rugae, setiferous puncta present between them. T2 sublateral longitudinal foveae: absent. T3 metasomal flanges: absent. T4 sculpture: longitudinally striate to rugose, setal pits spanning interspaces. T4 metasomal flanges: absent. T5 sculpture: longitudinally striate to rugose, setal pits spanning interspaces. T5 metasomal flanges: absent. T6: broader than long. Major sculpture of T6: umbilicate punctate. Microsculpture of T6: absent. T6 medially: flat and tapering to a rounded apex, not separated from apical rim. T6 metasomal flanges: absent. T6 raised peripheral rim: absent. S4 sculpture: longitudinally striate or rugose, setal pits spanning interspaces. S5 sculpture: longitudinally striate to rugose, setal pits spanning interspaces. S5 median carina: present. S6 peripheral carina: absent. S6 apex in relation to T6: not exposed to dorsal view. S6 apex: rounded or acuminate.

*Male*. Body length 3.35–3.45 mm (n=2). A3: shorter than pedicel; as long as pedicel. A5 tyloid shape: narrow, linear. A6: broader than long. A11: longer than broad. Major sculpture of mesoscutal midlobe anteriorly: umbilicate foveate. Major sculpture of mesoscutal midlobe posteriorly: umbilicate foveate; longitudinally rugose. Microsculpture of mesoscutal midlobe anteriorly: granulate. Microsculpture of mesoscutal midlobe posteriorly: absent; granulate. Major sculpture of mesoscutellum centrally: longitudinally rugose. Major sculpture of mesoscutellum peripherally: umbilicate foveate. Microsculpture of mesoscutellum centrally: absent. Microsculpture of mesoscutellum peripherally: granulate. Fore wing apex at rest: exceeding metasomal apex. T1 midlobe longitudinal carinae: 4. T3 metasomal flanges: absent. T4 metasomal flanges: absent. T5 metasomal flanges: absent. T6 metasomal flanges: absent. T7: truncate.

##### Diagnosis.

Both sexes: Frontal depression flat, with oblique interrupted carinae and a strong longitudinal carina that extends throughout its length; submedian carina indicated by weak rugae. Hyperoccipital carina indicated by rugae. Occipital carina connected to hyperoccipital carina by a weak longitudinal carina, laterally with strong corners and arched; area between occipital and hyperoccipital carinae densely sculptured and having many short setae. Mesoscutellum smooth, with scattered setiferous puncta. Metascutellum tiny, concave. Postmarginal vein present. Coxa not darker than rest of leg. Metasomal depression smooth. T1 lateral carina not expanded laterally. Metasomal flanges absent. Female: A3 not longer than pedicel. A4 longer than broad, A5 broader than long. T1 midlobe with 5 longitudinal carinae. Fore wing long enough to reach middle of T6 or beyond metasomal apex. T6 broader than long. Male: A4 about as broad as long, A11 longer than broad. T1 midlobe with 4 longitudinal carinae. Fore wing long enough to exceed metasomal apex. T7 tiny, truncate apically.

##### Etymology.

Latin noun, genitive case, meaning “a division.”

##### Link to distribution map.

[http://hol.osu.edu/map-full.html?id=307076]

##### Material examined.

Holotype, female: AUSTRALIA: QLD, summit TV station, rainforest, Bellenden Ker Range, 1560m, 17.X–5.XI.1981, yellow pan trap, OSUC 368204 (deposited in QMBA). Paratypes: AUSTRALIA: 10 females, 2 males, OSUC 368195, 368197-368199, 368205-368206 (ANIC); OSUC 462581 (CNCI); OSUC 368196, 368200-368203 (WINC).

#### Oxyscelio exiguitatis

Burks sp. n.

http://zoobank.org/70513FD4-F630-4E90-B766-1FD74195425D

urn:lsid:biosci.ohio-state.edu:osuc_concepts:307077

http://species-id.net/wiki/Oxyscelio_exiguitatis

**Figures 97–100.**
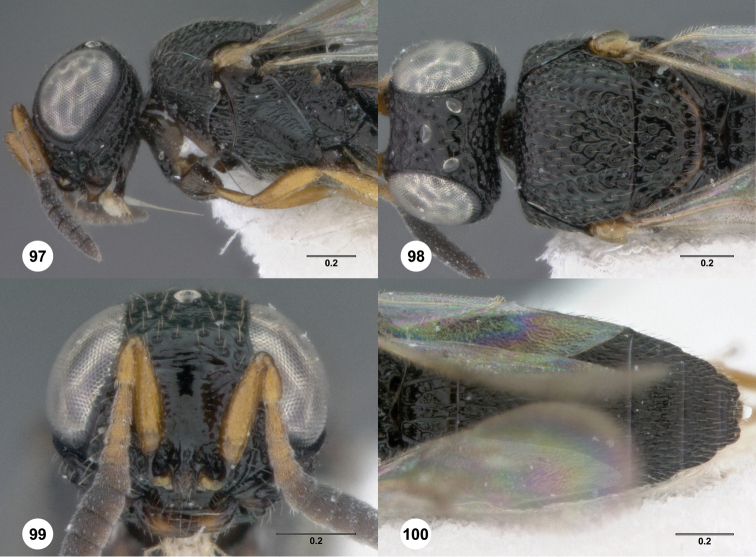
*Oxyscelio exiguitatis* sp. n., holotype female (OSUC 359682) **97** Head and mesosoma, lateral view **98** Head and mesosoma, dorsal view **99** Head, anterior view **100** Metasoma, dorsal view. Morphbank^46^

Figures 97–100 ; Morphbank^46^

##### Description.

Female. Body length 2.3–3.05 mm (n=15).

Radicle color and shade: darker than scape. Pedicel color: same as scape. A3: shorter than pedicel. A4: longer than broad. A5: broader than long.

Ventral clypeal margin: with slightly convex median lobe. Interantennal process: not elongate. Lower frons at dorsal margin of interantennal process: without transverse carina. Transverse curved rugae extending from frontal depression to eye: absent. Median longitudinal carina in frontal depression: absent. Ventral portion of frontal depression: with medially interrupted transverse carinae. Dorsal portion of frontal depression: with some transverse carinae. Submedian carina: present only as a weak shift in elevation. Frontal depression dorsally: not hood-like, open dorsally. Upper frons major sculpture: umbilicate foveate. Upper frons microsculpture: granulate. Hyperoccipital carina: indicated by a set of irregular elevations. Carina connecting occipital carina to hyperoccipital carina: absent. Occipital carina: omicron-shaped, with sharp corners where median portion meets lateral portions. Occiput sculpture: umbilicate foveate. Extra carina ventral to occipital carina: absent. Gena length: shorter than eye. Major sculpture of gena anteroventrally: umbilicate foveate. Major sculpture of gena posteroventrally: umbilicate foveate; absent. Microsculpture of gena anteroventrally: granulate. Microsculpture of gena posteroventrally: absent.

Lateral pronotal area sculpture: with shallow irregular carinae, posterodorsal corner with dense microsculpture. Posterior border of central pronotal area: directed anteriorly, protruding at corner of epomial carina and transverse pronotal carina. Mesoscutum anteriorly: very steep and tall, descending at a right angle or protruding anteriorly. Major sculpture of mesoscutal midlobe anteriorly: umbilicate foveate. Mesoscutal midlobe sculpture at midlength: not different from nearby sculpture. Major sculpture of mesoscutal midlobe posteriorly: umbilicate foveate; irregularly rugose. Microsculpture of mesoscutal midlobe anteriorly: granulate. Microsculpture of mesoscutal midlobe posteriorly: absent. Median mesoscutal carina: present as a vague, occasionally interrupted elevation. Major sculpture of mesoscutellum centrally: umbilicate foveate. Major sculpture of mesoscutellum peripherally: umbilicate foveate. Microsculpture of mesoscutellum centrally: absent; punctate. Microsculpture of mesoscutellum peripherally: absent; punctate. Mesoscutellar rim: not expanded. Mesoscutellar rim medially: without notch. Mesofemoral depression: longitudinally striate dorsally, smooth ventrally. Metascutellum shape: slightly emarginate posteriorly, concave but elevated posteriorly. Metascutellar setae: absent. Metascutellum sculpture: with large smooth posterior fovea. Postmarginal vein: absent. Fore wing apex at rest: reaching middle of T5. Coxae color brightness: darker than femora. Spines along tibiae: absent. Lateral propodeal carinae: broadly separated, not parallel anteriorly. Setae in metasomal depression: absent. Anterior sculpture of metasomal depression: absent. Median propodeal carina: absent.

T1 horn: absent. Number of longitudinal carinae of T1 midlobe: 4. T1 lateral carina: protruding laterally, visible from ventral view. T2 sculpture: with longitudinal striae or rugae, setiferous puncta present between them. T2 sublateral longitudinal foveae: absent. T3 metasomal flanges: absent. T4 sculpture: longitudinally striate to rugose, setal pits spanning interspaces. T4 metasomal flanges: absent. T5 sculpture: longitudinally striate to rugose, setal pits spanning interspaces. T5 metasomal flanges: absent. T6: broader than long. Major sculpture of T6: umbilicate punctate. Microsculpture of T6: absent. T6 medially: with medially truncate emargination, sloping down to apical rim. T6 metasomal flanges: present as tiny apical sharp projections. T6 raised peripheral rim: absent. S4 sculpture: longitudinally striate or rugose, setal pits spanning interspaces. S5 sculpture: longitudinally striate to rugose, setal pits spanning interspaces. S5 median carina: present. S6 peripheral carina: absent. S6 apex in relation to T6: not exposed to dorsal view. S6 apex: rounded or acuminate.

*Male*. unknown.

##### Diagnosis.

Both sexes: Frontal depression deep, carinae present above dorsal separator; submedian carina weak or absent medially. Hyperoccipital carina indicated by rugae. Occipital carina complete, medially sinuate. Metascutellum concave dorsally, weakly emarginate apically, projecting dorsally. Coxa darker than rest of leg. Postmarginal vein absent. Female: A3 longer than pedicel. A4, A5 broader than long. Fore wing long enough to reach middle of T5 or to metasomal apex. T4, T5 without metasomal flanges. T6 abruptly narrower than T5. Main body of T6 raised above apical rim, medially sloping down to it. T6 with broadly rounded apical lobes. S6 not exposed to dorsal view. *Oxyscelio exiguitatis* is very similar to *Oxyscelio nigricoxa*, but with much shorter fore wings and a deeper frontal depression.

##### Etymology.

Latin noun, genitive case, meaning “smallness.”

##### Link to distribution map.

[http://hol.osu.edu/map-full.html?id=307077]

##### Material examined.

Holotype, female: AUSTRALIA: QLD, 7km S Batavia Downs, 12°43'S, 142°42'E, 23.XI–11.XII.1992, malaise trap, P. Zborowski & W. Dressler, OSUC 359682 (deposited in ANIC). Paratypes: AUSTRALIA: 14 females, ANIC DB 32-020148, OSUC 359684, OSUC 359685, OSUC 359686, OSUC 359687, OSUC 359688, OSUC 359689, OSUC 359690, OSUC 359691, OSUC 359692, OSUC 359693, OSUC 359694, OSUC 359695 (ANIC); OSUC 359683 (WINC).

#### Oxyscelio flavipes

(Kieffer)

http://zoobank.org/315E663B-10FF-440D-BE9F-156BED579B44

urn:lsid:biosci.ohio-state.edu:osuc_concepts:5018

http://species-id.net/wiki/Oxyscelio_flavipes

**Figures 101–106.**
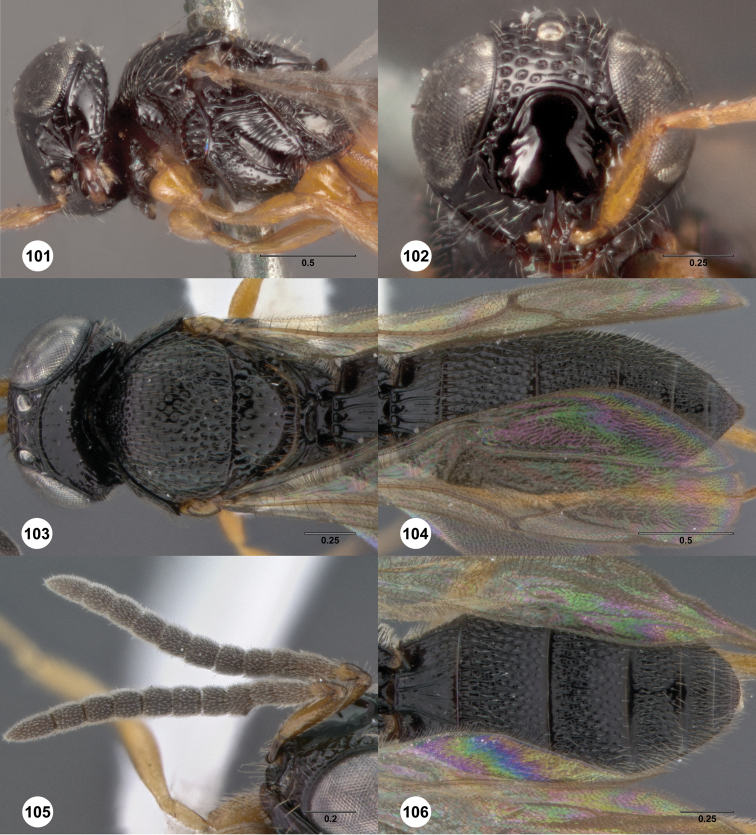
*Oxyscelio flavipes* (Kieffer), Holotype female (B.M. TYPE HYM. 9.510) **101** Head and mesosoma, lateral view **102** Head, anterior view. Female (OSUC 438914) **103** Head and mesosoma, dorsal view. **104** Metasoma, dorsal view. Male (OSUC 227622) **105** Antenna **106** Metasoma, dorsal view. Morphbank^47^

Figures 101–106 ; Morphbank^47^

Sceliomorpha flavipes Kieffer, 1907: 296 (original description); Masner 1965: 96 (type information).
Psilanteris flavipes (Kieffer): Kieffer 1916: 177 (generic transfer); Kieffer 1926: 433, 435 (description, keyed).
Oxyscelio flavipes (Kieffer): Dodd 1931: 75 (generic transfer); Galloway 1976: 99 (type information); Masner 1976: 24.

##### Description.

Female. Body length 2.85–4 mm (n=20).

Radicle color and shade: same as scape, both yellowish or reddish. Pedicel color: same as scape. A3: longer than pedicel. A4: broader than long. A5: broader than long.

Ventral clypeal margin: with slightly convex median lobe. Interantennal process: not elongate. Lower frons at dorsal margin of interantennal process: with transverse ledge, face sharply receding below it. Transverse curved rugae extending from frontal depression to eye: absent. Median longitudinal carina in frontal depression: present. Ventral portion of frontal depression: smooth. Dorsal portion of frontal depression: without transverse carinae. Submedian carina: present; present only as a weak shift in elevation. Frontal depression dorsally: not hood-like, open dorsally. Upper frons major sculpture: umbilicate foveate; irregularly rugose. Upper frons microsculpture: absent. Hyperoccipital carina: present as a single carina. Carina connecting occipital carina to hyperoccipital carina: absent. Occipital carina: present laterally, absent medially. Occiput sculpture: umbilicate punctate. Extra carina ventral to occipital carina: present, complete. Gena length: shorter than eye. Major sculpture of gena anteroventrally: umbilicate foveate; umbilicate punctate; absent. Major sculpture of gena posteroventrally: umbilicate punctate; absent. Microsculpture of gena anteroventrally: absent. Microsculpture of gena posteroventrally: absent.

Lateral pronotal area sculpture: smooth anteriorly, densely setose posteriorly. Posterior border of central pronotal area: directed posteriorly, epomial carina absent or meeting transverse pronotal carina at arch on lateral surface of pronotum. Mesoscutum anteriorly: not steep, forming less than a right angle. Major sculpture of mesoscutal midlobe anteriorly: umbilicate foveate; umbilicate punctate. Mesoscutal midlobe sculpture at midlength: not different from nearby sculpture. Major sculpture of mesoscutal midlobe posteriorly: umbilicate foveate; longitudinally rugose. Microsculpture of mesoscutal midlobe anteriorly: granulate. Microsculpture of mesoscutal midlobe posteriorly: absent. Median mesoscutal carina: absent. Major sculpture of mesoscutellum centrally: umbilicate foveate. Major sculpture of mesoscutellum peripherally: umbilicate foveate; umbilicate punctate. Microsculpture of mesoscutellum centrally: absent. Microsculpture of mesoscutellum peripherally: absent. Mesoscutellar rim: not expanded. Mesoscutellar rim medially: without notch. Mesofemoral depression: longitudinally striate dorsally, smooth ventrally. Metascutellum shape: not emarginate, concave but elevated posteriorly. Metascutellar setae: absent. Metascutellum sculpture: with large smooth posterior fovea. Spines along tibiae: absent. Lateral propodeal carinae: broadly separated, but parallel for a short distance anteriorly. Setae in metasomal depression: absent. Anterior sculpture of metasomal depression: absent. Median propodeal carina: absent. Postmarginal vein: present. Fore wing apex at rest: reaching middle of T6. Coxae color brightness: same color as femora.

T1 horn: absent. Number of longitudinal carinae of T1 midlobe: obscured by other raised sculpture. T1 lateral carina: straight. T2 sculpture: densely foveolate, longitudinal sculpture irregular. T2 sublateral longitudinal foveae: absent. T3 metasomal flanges: absent. T4 sculpture: longitudinally striate to rugose, setal pits spanning interspaces. T4 metasomal flanges: absent. T5 sculpture: longitudinally striate to rugose, setal pits spanning interspaces. T5 metasomal flanges: absent. T6: broader than long. Major sculpture of T6: umbilicate punctate. Microsculpture of T6: absent. T6 medially: flat and tapering to a rounded apex, not separated from apical rim. T6 metasomal flanges: absent. T6 raised peripheral rim: present. S4 sculpture: longitudinally striate or rugose, setal pits spanning interspaces. S5 sculpture: longitudinally striate to rugose, setal pits spanning interspaces. S5 median carina: absent. S6 peripheral carina: absent. S6 apex in relation to T6: not exposed to dorsal view. S6 apex: rounded or acuminate.

*Male*. Body length 2.45–4 mm (n=20). A3: longer than pedicel. A5 tyloid shape: narrow, linear. A6: broader than long. A11: longer than broad. Major sculpture of mesoscutal midlobe anteriorly: umbilicate foveate. Major sculpture of mesoscutal midlobe posteriorly: umbilicate foveate; longitudinally rugose. Microsculpture of mesoscutal midlobe anteriorly: granulate. Microsculpture of mesoscutal midlobe posteriorly: absent. Major sculpture of mesoscutellum centrally: umbilicate punctate; absent. Major sculpture of mesoscutellum peripherally: umbilicate foveate. Microsculpture of mesoscutellum centrally: absent. Microsculpture of mesoscutellum peripherally: absent. Fore wing apex at rest: exceeding metasomal apex. T1 midlobe longitudinal carinae: 4. T3 metasomal flanges: absent. T4 metasomal flanges: absent. T5 metasomal flanges: absent. T6 metasomal flanges: absent. T7: truncate.

##### Diagnosis.

Both sexes: Frontal depression shallow, transverse carinae absent or interrupted; submedian carina indicated by a weak ruga, flat or only weakly rounded dorsally. Hyperoccipital carina sharp and strong. Occipital carina incomplete, lateral portions nearly reaching hyperoccipital carina. Occiput almost entirely smooth, with a row of setiferous puncta. Mesoscutum without median carina. Metascutellum broad and concave, truncate or slightly convex apically, projecting dorsally. Postmarginal vein present. Coxa not darker than rest of leg. T1 lateral carina not expanded laterally. Metasomal flanges absent. Female: A3 much longer than pedicel. A4 longer than broad, A5 broader than long. Mesoscutellum weakly sculptured, with only setiferous puncta or small foveae. T1 midlobe with 4 longitudinal carinae. Fore wing long enough to reach T6. T6 broader than long. Male: A4 broader than long, A11 longer than broad; A5 tyloid narrow. T1 midlobe with 4 longitudinal carinae. Fore wing long enough to reach far beyond metasomal apex. T7 tiny, truncate.

##### Link to distribution map.

[http://hol.osu.edu/map-full.html?id=5018]

##### Material examined.

Holotype, female, S. flavipes: AUSTRALIA: QLD, Mackay, II-1900, R. E. Turner, B.M. TYPE HYM. 9.510 (deposited in BMNH). Other material: AUSTRALIA: 106 females, 64 males, Australian Museum K245259, K245269 (AMSA); OSUC 368242-368244, 368254, 438857-438858, 438874-438878, 438886, 438901-438906, 438914, 438916-438917, 438920, 438923, 445348, 451292, 451294, 451296-451297 (ANIC); OSUC 227566, 227606, 227609, 227615, 227622, 227629, 462592-462597 (CNCI); OSUC 376737 (MCZC); OSUC 165732, 221760-221762, 221764, 451273, 62719, 62728, 62739-62741, 63076, 63086, 63095, 63097, 63102, 63104-63105, 63190, 63228, 72257-72258 (OSUC); OSUC 368253, 438891, 438910, 451269, QDPC 0-165632, QDPC 0-165635, QDPC 0-165638, QDPC 0-165639, QDPC 0-165642, QDPC 0-165644, QDPC 0-165657, QDPC 0-165665, QDPC 0-165679, QDPC 0-165693, QDPC 0-165706, QDPC 0-165720, QDPC 0-165721, QDPC 0-165730, QDPC 0-165734, QDPC 0-165736, QDPC 0-165747, QDPC 0-165758, QDPC 0-165770, QDPC 0-165772, QDPC 0-165778, QDPC 0-165787 (QDPC); OSUC 148371, 368240-368241, 368256, 438879-438880, 438907-438909, 438915 (QMBA); OSUC 448596 (QPIM); UCRC ENT 100244 (UCRC); OSUC 368238-368239, 368245-368252, 368255, 438855-438856, 438859-438873, 438881-438885, 438887-438890, 438892-438900, 438911-438913, 438918-438919, 438921-438922, 438924-438929, 445347, 451268, 451270-451272, 451293, 451295, 451298 (WINC).

##### Comments.

Our concept of this species allows for considerable variation in sculpture and setation. The sculptural variation includes differences in the height and distinction of the submedian carina, strength of a longitudinal ridge along the median axis of the frontal depression, sculpture of the metasomal depression, and strength of the longitudinal elevations on the posteromedian part of the mesoscutal midlobe. Density of setation also may vary on the occiput and mesoscutellum. This variation does correlate with collection locality. It is possible that our concept of *Oxyscelio flavipes* applies to several sibling species, but no consistent patterns of variation were found to support that possibility.

#### Oxyscelio fluctuum

Burks sp. n.

http://zoobank.org/57EF33BC-8A25-47F4-89BF-5ED6C8E37D33

urn:lsid:biosci.ohio-state.edu:osuc_concepts:307078

http://species-id.net/wiki/Oxyscelio_fluctuum

**Figures 107–112.**
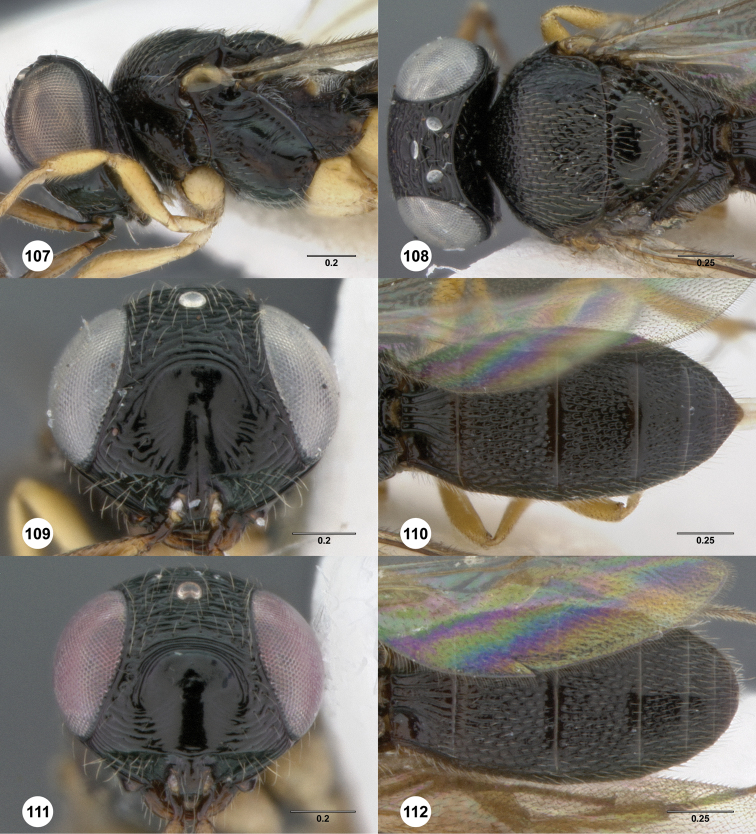
*Oxyscelio fluctuum* sp. n., paratype female (OSUC 148368) **107** Head and mesosoma, lateral view. Paratype female (OSUC 148365) **108** Head and mesosoma, dorsal view **109** Head, anterior view **110** Metasoma, dorsal view. Paratype male (OSUC 438977) **111** Head, anterior view **112** Metasoma, dorsal view. Morphbank^48^

Figures 107–112 ; Morphbank^48^

##### Description.

Female. Body length 2.55–3.25 mm (n=17).

Radicle color and shade: darker than scape. Pedicel color: same as scape. A3: longer than pedicel. A4: broader than long. A5: broader than long.

Ventral clypeal margin: with slightly convex median lobe. Interantennal process: not elongate. Lower frons at dorsal margin of interantennal process: with transverse ledge, face sharply receding below it. Transverse curved rugae extending from frontal depression to eye: present. Median longitudinal carina in frontal depression: absent. Ventral portion of frontal depression: with medially interrupted transverse carinae. Dorsal portion of frontal depression: without transverse carinae. Submedian carina: present. Frontal depression dorsally: not hood-like, open dorsally. Upper frons major sculpture: umbilicate foveate; transversely rugose. Upper frons microsculpture: absent. Hyperoccipital carina: present as a single carina. Carina connecting occipital carina to hyperoccipital carina: absent. Occipital carina: present laterally, absent medially. Occiput sculpture: umbilicate punctate. Extra carina ventral to occipital carina: present, complete. Gena length: shorter than eye. Major sculpture of gena anteroventrally: umbilicate punctate; absent. Major sculpture of gena posteroventrally: umbilicate punctate; absent. Microsculpture of gena anteroventrally: absent. Microsculpture of gena posteroventrally: absent.

Lateral pronotal area sculpture: smooth anteriorly, densely setose posteriorly. Posterior border of central pronotal area: directed posteriorly, epomial carina absent or meeting transverse pronotal carina at arch on lateral surface of pronotum. Mesoscutum anteriorly: not steep, forming less than a right angle. Major sculpture of mesoscutal midlobe anteriorly: umbilicate punctate. Mesoscutal midlobe sculpture at midlength: not different from nearby sculpture. Major sculpture of mesoscutal midlobe posteriorly: obliquely rugose; umbilicate punctate. Microsculpture of mesoscutal midlobe anteriorly: absent. Microsculpture of mesoscutal midlobe posteriorly: absent. Median mesoscutal carina: absent. Major sculpture of mesoscutellum centrally: umbilicate punctate. Major sculpture of mesoscutellum peripherally: umbilicate punctate. Microsculpture of mesoscutellum centrally: absent. Microsculpture of mesoscutellum peripherally: absent. Mesoscutellar rim: not expanded. Mesoscutellar rim medially: without notch. Mesofemoral depression: longitudinally striate dorsally, smooth ventrally. Metascutellum shape: slightly emarginate posteriorly, concave but elevated posteriorly. Metascutellar setae: absent. Metascutellum sculpture: with large smooth posterior fovea. Postmarginal vein: present. Fore wing apex at rest: exceeding metasomal apex. Coxae color brightness: same color as femora. Spines along tibiae: absent. Lateral propodeal carinae: narrowly separated, angled anteriorly to become parallel. Setae in metasomal depression: absent. Anterior sculpture of metasomal depression: with median areole or pair of pits. Median propodeal carina: present.

T1 horn: absent. Number of longitudinal carinae of T1 midlobe: 4. T1 lateral carina: straight. T2 sculpture: densely foveolate, longitudinal sculpture irregular. T2 sublateral longitudinal foveae: absent. T3 metasomal flanges: absent. T4 sculpture: densely foveate, longitudinal sculpture irregular. T4 metasomal flanges: absent. T5 sculpture: densely foveate, longitudinal sculpture irregular. T5 metasomal flanges: absent. T6: broader than long. Major sculpture of T6: umbilicate punctate. Microsculpture of T6: absent. T6 medially: flat and tapering to a rounded apex, not separated from apical rim. T6 metasomal flanges: absent. T6 raised peripheral rim: absent. S4 sculpture: longitudinally striate or rugose, setal pits spanning interspaces. S5 sculpture: longitudinally striate to rugose, setal pits spanning interspaces. S5 median carina: absent. S6 peripheral carina: absent. S6 apex in relation to T6: not exposed to dorsal view. S6 apex: rounded or acuminate.

*Male*. Body length 2.45–3.1 mm (n=). A3: longer than pedicel. A5 tyloid shape: narrow, linear. A6: longer than broad. A11: longer than broad. Major sculpture of mesoscutal midlobe anteriorly: umbilicate foveate. Major sculpture of mesoscutal midlobe posteriorly: umbilicate foveate; irregularly rugose. Microsculpture of mesoscutal midlobe anteriorly: granulate. Microsculpture of mesoscutal midlobe posteriorly: absent. Major sculpture of mesoscutellum centrally: umbilicate foveate; umbilicate punctate. Major sculpture of mesoscutellum peripherally: umbilicate foveate; umbilicate punctate. Microsculpture of mesoscutellum centrally: absent. Microsculpture of mesoscutellum peripherally: absent. Fore wing apex at rest: exceeding metasomal apex. T1 midlobe longitudinal carinae: 4. T3 metasomal flanges: absent. T4 metasomal flanges: absent. T5 metasomal flanges: absent. T6 metasomal flanges: absent. T7: truncate.

##### Diagnosis.

Both sexes: Frontal depression shallow; submedian carina indicated by a set of weak rugae, flat or only weakly rounded dorsally. Hyperoccipital carina sharp and strong. Occipital carina incomplete, lateral portions nearly reaching hyperoccipital carina. Occiput almost entirely smooth, with a row of setiferous puncta. Mesoscutum without median carina. Metascutellum broad and concave, truncate or slightly convex apically, projecting dorsally. Postmarginal vein present. Coxa not darker than rest of leg. T1 lateral carina not expanded laterally. Metasomal flanges absent. Female: A4, A5 broader than long. Frontal depression with very weak oblique, interrupted carinae in lower portion. Mesoscutellum weakly sculptured, with only setiferous puncta or small foveae. T1 midlobe with 4 longitudinal carinae. Fore wing long enough to reach far beyond metasomal apex. T6 broader than long. Male: A4 broader than long, A11 longer than broad. Frontal depression with long, strongly oblique carinae that are continuous with rugae that extend outside frontal depression to approach eye margin. T1 midlobe with 4 longitudinal carinae. Fore wing long enough to reach far beyond metasomal apex. T7 tiny, truncate. The head of *Oxyscelio fluctuum* is similar to that in *Oxyscelio rugulosus*, except in having much stronger and more elongate oblique carinae. The body otherwise resembles that of *Oxyscelio flavipes*.

##### Etymology.

Latin noun, genitive case, meaning “waves.”

##### Link to distribution map.

[http://hol.osu.edu/map-full.html?id=307078]

##### Material examined.

Holotype, female: AUSTRALIA: QLD, rainforest, Charmillin Creek, 17°42'S, 145°31'E, 940m, 1.XII.1997, C. J. Burwell, OSUC 148365 (deposited in QMBA). Paratypes: AUSTRALIA: 16 females, 54 males, Australian Museum K245260 (AMSA); OSUC 438952-438955, 438958-438988, 438992, 439003 (ANIC); QDPC 0-165636, 0-165669, 0-165751, 0-165752, 0-165771, 0-165775, 0-165780, 0-165781, 0-165782, 0-165789 (QDPC); OSUC 148368, 368235, 438957, 438993 (QMBA); OSUC 368233-368234, 368236-368237, 438956, 438989-438991, 438994-439002, 451306 (WINC).

#### Oxyscelio foliorum

Burks sp. n.

http://zoobank.org/C0B96CD8-566E-4683-AE35-E1E9F1366348

urn:lsid:biosci.ohio-state.edu:osuc_concepts:307079

http://species-id.net/wiki/Oxyscelio_foliorum

**Figures 113–118.**
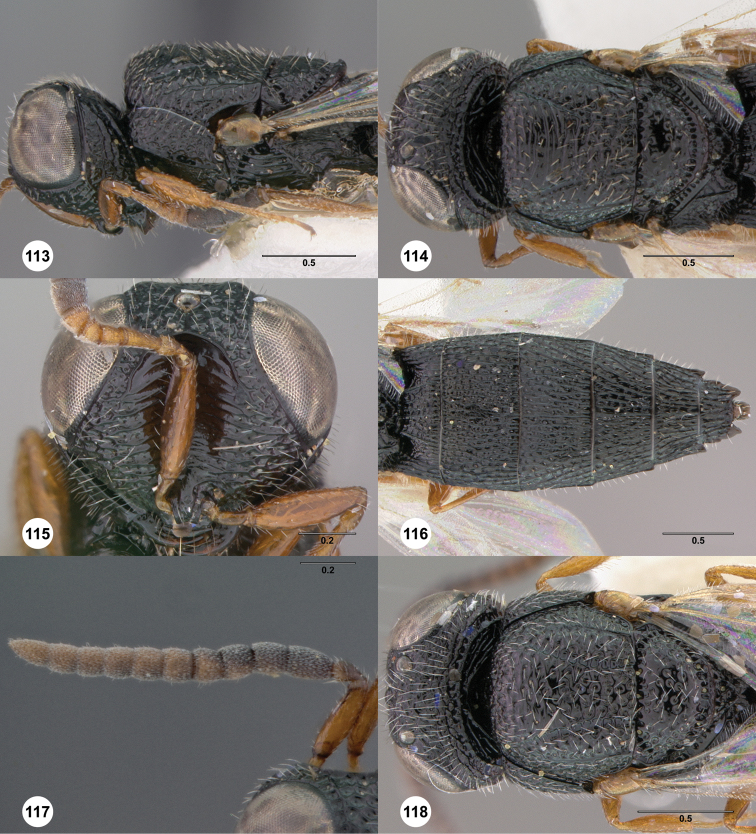
*Oxyscelio foliorum* sp. n., holotype female (OSUC 376702) **113** Head and mesosoma, lateral view **114** Head and mesosoma, dorsal view **115** Head, anterior view **116** Metasoma, dorsal view. Paratype male (OSUC 376701) **117** Antenna **118** Metasoma, dorsal view. Morphbank^49^

Figures 113–118 ; Morphbank^49^

##### Description.

Female. Body length 4.6 mm (n=1).

Radicle color and shade: same as scape, both yellowish or reddish. Pedicel color: same as scape. A3: longer than pedicel. A4: as long as broad. A5: broader than long.

Ventral clypeal margin: with slightly convex median lobe. Interantennal process: not elongate. Lower frons at dorsal margin of interantennal process: without transverse carina. Transverse curved rugae extending from frontal depression to eye: absent. Median longitudinal carina in frontal depression: absent. Ventral portion of frontal depression: with transverse carinae. Dorsal portion of frontal depression: without transverse carinae. Submedian carina: present. Frontal depression dorsally: not hood-like, open dorsally. Upper frons major sculpture: umbilicate foveate. Upper frons microsculpture: absent. Hyperoccipital carina: absent. Carina connecting occipital carina to hyperoccipital carina: absent. Occipital carina: weakly arched dorsally, with rounded lateral corners. Occiput sculpture: irregularly sculptured. Extra carina ventral to occipital carina: present, complete. Gena length: shorter than eye. Major sculpture of gena anteroventrally: umbilicate foveate; rugose. Major sculpture of gena posteroventrally: umbilicate foveate. Microsculpture of gena anteroventrally: absent. Microsculpture of gena posteroventrally: absent.

Lateral pronotal area sculpture: with a series of arched carinae, posterodorsal corner with weak longitudinal rugae. Posterior border of central pronotal area: directed anteriorly, protruding at corner of epomial carina and transverse pronotal carina. Mesoscutum anteriorly: very steep and tall, descending at a right angle or protruding anteriorly. Major sculpture of mesoscutal midlobe anteriorly: transversely rugose; umbilicate punctate. Mesoscutal midlobe sculpture at midlength: not different from nearby sculpture. Major sculpture of mesoscutal midlobe posteriorly: umbilicate foveate. Microsculpture of mesoscutal midlobe anteriorly: absent. Microsculpture of mesoscutal midlobe posteriorly: absent. Median mesoscutal carina: absent. Major sculpture of mesoscutellum centrally: absent. Major sculpture of mesoscutellum peripherally: umbilicate foveate. Microsculpture of mesoscutellum centrally: absent; punctate. Microsculpture of mesoscutellum peripherally: punctate. Mesoscutellar rim: expanded. Mesoscutellar rim medially: without notch. Mesofemoral depression: longitudinally striate dorsally, smooth ventrally. Metascutellum shape: not emarginate, concave but elevated posteriorly. Metascutellar setae: absent. Metascutellum sculpture: with large smooth posterior fovea. Postmarginal vein: absent. Fore wing apex at rest: reaching base of T5. Coxae color brightness: darker than femora. Spines along tibiae: absent. Lateral propodeal carinae: broadly separated, not parallel anteriorly. Setae in metasomal depression: absent. Anterior sculpture of metasomal depression: absent. Median propodeal carina: absent.

T1 horn: absent. Number of longitudinal carinae of T1 midlobe: 4. T1 lateral carina: protruding laterally, visible from ventral view. T2 sculpture: with longitudinal striae or rugae, setiferous puncta present between them. T2 sublateral longitudinal foveae: absent. T3 metasomal flanges: present. T4 sculpture: longitudinally striate to rugose, setal pits spanning interspaces. T4 metasomal flanges: present as slightly protruding sharp corners. T5 sculpture: longitudinally striate to rugose, setal pits spanning interspaces. T5 metasomal flanges: present as strongly protruding acuminate flanges. T6: broader than long. Major sculpture of T6: umbilicate punctate; longitudinally striate. Microsculpture of T6: absent. T6 medially: with medially truncate emargination, sloping down to apical rim. T6 metasomal flanges: present, broad and elongate, with slight lateral incisions near midlength. T6 raised peripheral rim: absent. S4 sculpture: longitudinally striate or rugose, setal pits spanning interspaces. S5 sculpture: longitudinally striate to rugose, setal pits spanning interspaces. S5 median carina: present. S6 peripheral carina: present, posteriorly complete. S6 apex in relation to T6: exposed to dorsal view by T6 emargination. S6 apex: truncate.

*Male*. Body length 4.1 mm (n=1). A3: longer than pedicel. A5 tyloid shape: narrow, linear. A6: broader than long. A11: longer than broad. Major sculpture of mesoscutal midlobe anteriorly: umbilicate foveate; transversely rugose. Major sculpture of mesoscutal midlobe posteriorly: umbilicate foveate. Microsculpture of mesoscutal midlobe anteriorly: absent. Microsculpture of mesoscutal midlobe posteriorly: absent. Major sculpture of mesoscutellum centrally: umbilicate foveate. Major sculpture of mesoscutellum peripherally: umbilicate foveate. Microsculpture of mesoscutellum centrally: punctate. Microsculpture of mesoscutellum peripherally: punctate. Fore wing apex at rest: reaching apex of T5. T1 midlobe longitudinal carinae: obscured by other raised sculpture. T3 metasomal flanges: absent. T4 metasomal flanges: absent. T5 metasomal flanges: present as strongly protruding acuminate flanges. T6 metasomal flanges: present as strongly protruding acuminate flanges. T7: M-shaped, with a triangular median emargination.

##### Diagnosis.

Both sexes: Frontal depression deep, all carinae complete medially, carinae present above dorsal separator; submedian carina very strong. Hyperoccipital carina absent. Occipital carina complete, medially weakly convex. Mesoscutal midlobe with strong transverse carinae. Mesoscutellar rim expanded and strongly sculptured, without median notch. Metascutellum pentagonal and shovel-like, projecting dorsally. Coxa darker than rest of leg. Postmarginal vein absent. Female: A3 longer than pedicel. A4, A5 broader than long. T4 with small sharp metasomal flanges. T5, T6 with elongate, flat and sharp metasomal flanges. T6 broadly and deeply emarginate, truncate or roundly concave medially. S6 exposed to dorsal view, truncate apically. Male: A3, A11 longer than broad; A4 slightly broader than long. Mesoscutellum and mesoscutal midlobe posteriorly with small foveae, mesoscutellum without smooth area medially. T5, T6 with long sharp metasomal flanges. T7 with flat sharp metasomal flanges, deeply emarginate medially, posterior margin deeply M-shaped. *Oxyscelio foliorum* is similar to *Oxyscelio limbi* in several ways, but differs in the short and flat metasoma with elongate, leaf-like metasomal flanges.

##### Etymology.

Latin noun, genitive case, meaning “leaves.”

##### Link to distribution map.

[http://hol.osu.edu/map-full.html?id=307079]

##### Associations.

On flower of *Heterodendron oleifolium* Desf.: [Sapindales: Sapindaceae]

##### Material examined.

Holotype, female: AUSTRALIA: SA, nr. Ikara (Wilpena Pound) Valley, Edeowie Homestead, 29.X.1972, H. E. Evans, OSUC 376702 (deposited in MCZC). Paratype: AUSTRALIA: 1 male, OSUC 376701 (MCZC).

#### Oxyscelio funis

Burks sp. n.

http://zoobank.org/AEA4DB0C-145D-474A-B58D-230D2554A827

urn:lsid:biosci.ohio-state.edu:osuc_concepts:307080

http://species-id.net/wiki/Oxyscelio_funis

**Figures 119–124.**
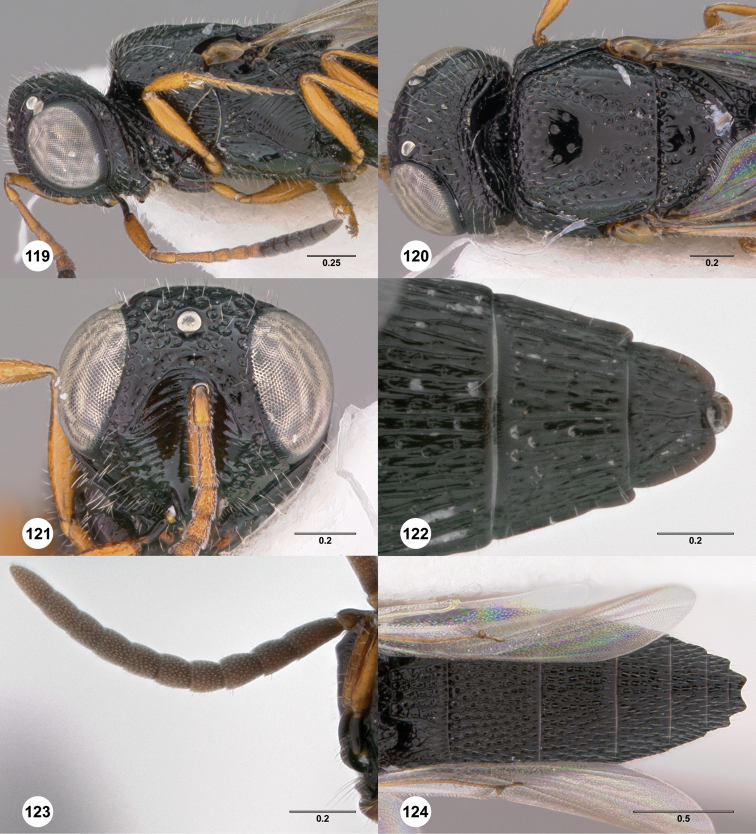
*Oxyscelio funis* sp. n., holotype female (OSUC 435969) **119** Head and mesosoma, lateral view **120** Head and mesosoma, dorsal view **121** Head, anterior view **122** Metasoma, dorsal view. Paratype male (OSUC 435980) **123** Antenna **124** Metasoma, dorsal view. Morphbank^50^

Figures 119–124 ; Morphbank^50^

##### Description.

Female. Body length 3.75–5.2 mm (n=17).

Radicle color and shade: darker than scape. Pedicel color: same as scape. A3: longer than pedicel. A4: longer than broad. A5: as long as broad.

Ventral clypeal margin: with slightly convex median lobe. Interantennal process: not elongate. Lower frons at dorsal margin of interantennal process: without transverse carina. Transverse curved rugae extending from frontal depression to eye: absent. Median longitudinal carina in frontal depression: absent. Ventral portion of frontal depression: with medially interrupted transverse carinae. Dorsal portion of frontal depression: with some transverse carinae. Submedian carina: present. Frontal depression dorsally: not hood-like, open dorsally. Upper frons major sculpture: umbilicate foveate; irregularly rugose. Upper frons microsculpture: absent. Hyperoccipital carina: absent. Carina connecting occipital carina to hyperoccipital carina: absent. Occipital carina: weakly arched dorsally, with rounded lateral corners. Occiput sculpture: irregularly sculptured. Extra carina ventral to occipital carina: present, medially incomplete. Gena length: shorter than eye. Major sculpture of gena anteroventrally: umbilicate foveate; rugose. Major sculpture of gena posteroventrally: umbilicate foveate; rugose. Microsculpture of gena anteroventrally: absent. Microsculpture of gena posteroventrally: absent.

Lateral pronotal area sculpture: with shallow irregular carinae, posterodorsal corner with dense microsculpture. Posterior border of central pronotal area: directed anteriorly, protruding at corner of epomial carina and transverse pronotal carina. Mesoscutum anteriorly: very steep and tall, descending at a right angle or protruding anteriorly. Major sculpture of mesoscutal midlobe anteriorly: umbilicate foveate. Mesoscutal midlobe sculpture at midlength: with large smooth areas. Major sculpture of mesoscutal midlobe posteriorly: umbilicate foveate; longitudinally rugose. Microsculpture of mesoscutal midlobe anteriorly: granulate. Microsculpture of mesoscutal midlobe posteriorly: absent. Median mesoscutal carina: absent; present as a vague, occasionally interrupted elevation. Major sculpture of mesoscutellum centrally: absent; umbilicate foveate. Major sculpture of mesoscutellum peripherally: umbilicate foveate. Microsculpture of mesoscutellum centrally: absent. Microsculpture of mesoscutellum peripherally: absent. Mesoscutellar rim: not expanded. Mesoscutellar rim medially: without notch; with notch. Mesofemoral depression: longitudinally striate dorsally, smooth ventrally. Metascutellum shape: not emarginate, concave but elevated posteriorly. Metascutellar setae: absent. Metascutellum sculpture: with large smooth posterior fovea. Postmarginal vein: absent. Fore wing apex at rest: not reaching base of T5. Coxae color brightness: darker than femora. Spines along tibiae: absent. Lateral propodeal carinae: broadly separated, not parallel anteriorly. Setae in metasomal depression: absent. Anterior sculpture of metasomal depression: absent. Median propodeal carina: absent.

T1 horn: absent. Number of longitudinal carinae of T1 midlobe: 4. T1 lateral carina: protruding laterally, visible from ventral view. T2 sculpture: with longitudinal striae or rugae, setiferous puncta present between them. T2 sublateral longitudinal foveae: absent. T3 metasomal flanges: absent. T4 sculpture: longitudinally striate to rugose, setal pits spanning interspaces. T4 metasomal flanges: present as slightly protruding sharp corners. T5 sculpture: longitudinally striate to rugose, setal pits spanning interspaces. T5 metasomal flanges: present, rounded and lobe-like. T6: broader than long. Major sculpture of T6: umbilicate punctate; longitudinally striate. Microsculpture of T6: absent. T6 medially: with deep emargination that is V-shaped medially, separated from apical rim. T6 metasomal flanges: present as slightly expanded lateral rims, rounded posteriorly. T6 raised peripheral rim: absent. S4 sculpture: longitudinally striate or rugose, setal pits spanning interspaces. S5 sculpture: longitudinally striate to rugose, setal pits spanning interspaces. S5 median carina: present. S6 peripheral carina: present, posteriorly complete. S6 apex in relation to T6: exposed to dorsal view by T6 emargination. S6 apex: rounded or acuminate.

*Male*. Body length 3.4–4.45 mm (n=19). A3: longer than pedicel. A5 tyloid shape: narrow, linear. A6: broader than long. A11: longer than broad. Major sculpture of mesoscutal midlobe anteriorly: umbilicate foveate. Major sculpture of mesoscutal midlobe posteriorly: umbilicate foveate. Microsculpture of mesoscutal midlobe anteriorly: granulate. Microsculpture of mesoscutal midlobe posteriorly: absent. Major sculpture of mesoscutellum centrally: umbilicate foveate. Major sculpture of mesoscutellum peripherally: umbilicate foveate. Microsculpture of mesoscutellum centrally: absent; punctate. Microsculpture of mesoscutellum peripherally: absent. Fore wing apex at rest: reaching middle of T5. T1 midlobe longitudinal carinae: 5; obscured by other raised sculpture. T3 metasomal flanges: absent. T4 metasomal flanges: absent. T5 metasomal flanges: present as slightly protruding sharp corners. T6 metasomal flanges: present as sharp corners that do not protrude. T7: M-shaped, with a triangular median emargination.

##### Diagnosis.

Both sexes: Frontal depression deep, all carinae complete medially, carinae present above dorsal separator; submedian carina weakly defined or absent medially. Hyperoccipital carina absent. Occipital carina complete, medially weakly convex or sinuate. Mesoscutellar rim not expanded, with or without median notch. Metascutellum scoop-like, projecting dorsally. Coxa darker than rest of leg. Postmarginal vein absent. Female: A3 longer than pedicel. A4 longer than broad. A5 as long or longer than broad. T4 with very weak metasomal flanges. T5 with lobe-like metasomal flanges. T6 with expanded lateral margins. T6 deeply emarginate, angularly emarginate medially. S6 exposed to dorsal view, rounded apically. Male: A3, A4, A11 longer than broad. Mesoscutellum and mesoscutal midlobe posteriorly with small and densely set foveae, mesoscutellum without smooth area medially. T5 with weak and indistinct metasomal flanges, T6 with sharp metasomal flanges. T7 with long postero-lateral lobes, deeply emarginate medially, posterior margin M-shaped. *Oxyscelio funis* can be recognized with care using the long antenna of both sexes. The transversely carinate dorsal portion of the frontal depression is distinctive, and the Metasomal flanges are unusually weak.

##### Etymology.

Latin noun, genitive case, meaning “rope.”

##### Link to distribution map.

[http://hol.osu.edu/map-full.html?id=307080]

##### Associations.

On blossom of *Eucalyptus* L’Hér.: [Myrtales: Myrtaceae]; on flower of *Heterodendron oleifolium* Desf.: [Sapindales: Sapindaceae]; on or near flowers of *Myoporum* Sol. ex G. Forst.: [Lamiales: Myoporaceae]

##### Material examined.

Holotype, female: AUSTRALIA: SA, 12km NE Morgan, 34°01'S, 139°49'E, 12.XI.1987, I. Naumann & J. Cardale, OSUC 435969 (deposited in ANIC). Paratypes: AUSTRALIA: 16 females, 19 males, OSUC 435961-435965, 435968, 435970-435985, 435987-435994 (ANIC); OSUC 376703, 376706 (MCZC); OSUC 435966-435967, 453991 (WINC).

#### Oxyscelio grandis

(Dodd)

http://zoobank.org/9B933FA1-39A4-4FB7-894C-11C823B16548

urn:lsid:biosci.ohio-state.edu:osuc_concepts:5022

http://species-id.net/wiki/Oxyscelio_grandis

**Figures 125–126.**
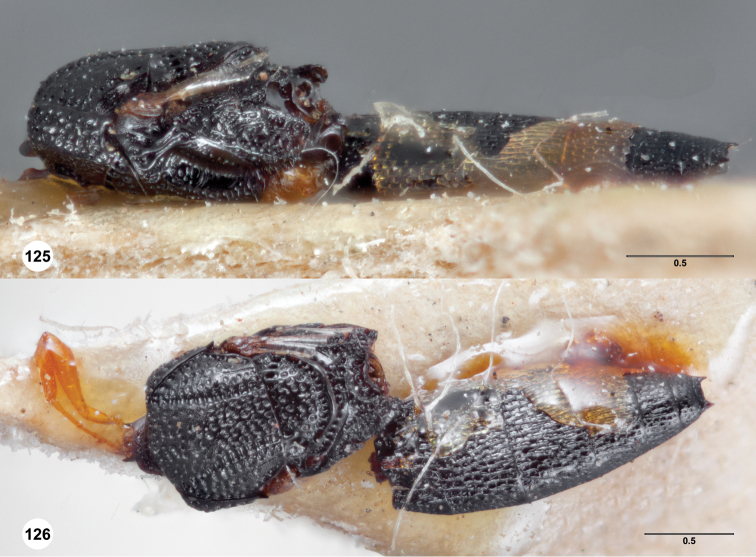
*Oxyscelio grandis* (Dodd), holotype male (SAMA DB 32-001588) **125** Mesosoma and metasoma, lateral view **126** Mesosoma and metasoma, dorsal view. Morphbank^51^

Figures 125–126 ; Morphbank^51^

Hoploteleia grandis Dodd, 1913: 176 (original description); Kieffer 1926: 367, 378 (description, keyed).
Oxyscelio grandis (Dodd): Dodd 1931: 75 (generic transfer); Galloway 1976: 99 (type information).

##### Description.

Female. Unknown.

*Male*. Mesosoma + metasoma length 3.38 mm (n=1).

Lateral pronotal area sculpture: anteriorly smooth, posterodorsal corner with dense microsculpture, ventral corner with irregular carinae. Posterior border of central pronotal area: directed anteriorly, protruding at corner of epomial carina and transverse pronotal carina. Mesoscutum anteriorly: not steep, forming less than a right angle. Median mesoscutal carina: absent. Mesoscutellar rim: not expanded. Mesoscutellar rim medially: without notch. Mesofemoral depression: longitudinally striate dorsally, smooth ventrally. Metascutellum shape: slightly emarginate posteriorly, concave but elevated posteriorly. Metascutellar setae: absent. Metascutellum sculpture: with large smooth posterior fovea. Coxae color brightness: same color as femora. Lateral propodeal carinae: broadly separated, not parallel anteriorly. Setae in metasomal depression: absent. Anterior sculpture of metasomal depression: absent. Median propodeal carina: absent. Major sculpture of mesoscutal midlobe anteriorly: umbilicate foveate. Major sculpture of mesoscutal midlobe posteriorly: umbilicate foveate. Microsculpture of mesoscutal midlobe anteriorly: granulate. Microsculpture of mesoscutal midlobe posteriorly: absent. Major sculpture of mesoscutellum centrally: umbilicate foveate; absent. Major sculpture of mesoscutellum peripherally: umbilicate foveate. Microsculpture of mesoscutellum centrally: absent. Microsculpture of mesoscutellum peripherally: absent. Fore wing apex at rest: unknown.

T1 midlobe longitudinal carinae: unknown. T3 metasomal flanges: absent. T4 metasomal flanges: absent. T5 metasomal flanges: absent. T6 metasomal flanges: absent. T7: with a pair of sharply defined spine-like posterolateral projections.

##### Diagnosis.

Male: Median carina of mesoscutum absent. Mesoscutellum without granulate sculpture. T1 lateral carina not expanded. T7 with narrow and elongate posterior spines.

##### Link to distribution map.

[http://hol.osu.edu/map-full.html?id=5022]

##### Material examined.

Holotype, male: H. grandis: AUSTRALIA: QLD, nr. Cairns, foliage / jungle, Gordonvale (Nelson), 8.V.1913, sweeping, A. P. Dodd, SAMA DB 32-001588 (deposited in SAMA).

##### Comments.

*Oxyscelio grandis* is known only from a broken male specimen consisting only of a mesosoma and metasoma, which was its state when described by Dodd (1913). These parts are sufficient to establish that no other known Australian specimens belong to this species. The elongate T7 spines are unlike those of any member of the *atricoxa*-group, suggesting that this species likely belongs to another species group, or that it is not closely related to any other known Australian *Oxyscelio*. This species is excluded from the phylogenetic analysis due to the very large amount of missing data.

#### Oxyscelio gressus

Burks sp. n.

http://zoobank.org/C86D2B48-55D3-43CA-9C04-033482990D2E

urn:lsid:biosci.ohio-state.edu:osuc_concepts:307081

http://species-id.net/wiki/Oxyscelio_gressus

**Figures 127–132.**
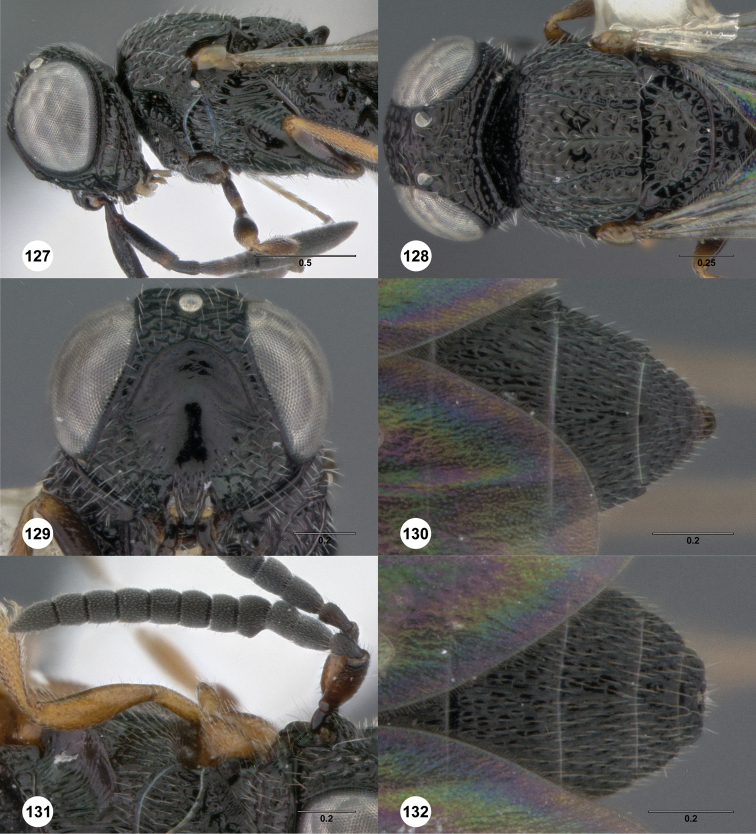
*Oxyscelio gressus* sp. n., holotype female (OSUC 456176) **127** Head and mesosoma, lateral view **128** Head and mesosoma, dorsal view **129** Head, anterior view **130** Metasoma, dorsal view. Paratype male (OSUC 456178) **131** Antenna **132** Metasoma, dorsal view. Morphbank^52^

Figures 127–132 ; Morphbank^52^

##### Description.

Female. Body length 3.55–3.7 mm (n=2).

Radicle color and shade: darker than scape. Pedicel color: same as scape. A3: shorter than pedicel; as long as pedicel. A4: broader than long. A5: broader than long.

Ventral clypeal margin: with slightly convex median lobe. Interantennal process: not elongate. Lower frons at dorsal margin of interantennal process: without transverse carina. Transverse curved rugae extending from frontal depression to eye: absent. Median longitudinal carina in frontal depression: absent. Ventral portion of frontal depression: with medially interrupted transverse carinae; smooth. Dorsal portion of frontal depression: with medially interrupted transverse carinae. Submedian carina: present. Frontal depression dorsally: not hood-like, open dorsally. Upper frons major sculpture: umbilicate foveate; irregularly rugose. Upper frons microsculpture: absent. Hyperoccipital carina: indicated by a set of irregular elevations. Carina connecting occipital carina to hyperoccipital carina: absent. Occipital carina: weakly arched dorsally, with rounded lateral corners. Occiput sculpture: transversely rugose. Extra carina ventral to occipital carina: present, complete. Gena length: shorter than eye. Major sculpture of gena anteroventrally: umbilicate foveate. Major sculpture of gena posteroventrally: rugose. Microsculpture of gena anteroventrally: absent. Microsculpture of gena posteroventrally: granulate.

Lateral pronotal area sculpture: anteriorly smooth, posterodorsal corner with dense microsculpture, ventral corner with irregular carinae. Posterior border of central pronotal area: directed anteriorly, protruding at corner of epomial carina and transverse pronotal carina. Mesoscutum anteriorly: very steep and tall, descending at a right angle or protruding anteriorly. Major sculpture of mesoscutal midlobe anteriorly: umbilicate foveate. Mesoscutal midlobe sculpture at midlength: with large smooth areas. Major sculpture of mesoscutal midlobe posteriorly: umbilicate foveate; irregularly rugose. Microsculpture of mesoscutal midlobe anteriorly: granulate. Microsculpture of mesoscutal midlobe posteriorly: absent. Median mesoscutal carina: present as a narrow carina. Major sculpture of mesoscutellum centrally: umbilicate foveate. Major sculpture of mesoscutellum peripherally: umbilicate foveate. Microsculpture of mesoscutellum centrally: absent. Microsculpture of mesoscutellum peripherally: absent. Mesoscutellar rim: not expanded. Mesoscutellar rim medially: without notch. Mesofemoral depression: longitudinally striate dorsally, smooth ventrally. Metascutellum shape: deeply emarginate, with the resulting pair of posterior processes subtriangular and directed dorsally. Metascutellar setae: absent. Metascutellum sculpture: with large smooth posterior fovea. Postmarginal vein: absent. Fore wing apex at rest: reaching middle of T5. Coxae color brightness: same color as femora. Spines along tibiae: absent. Lateral propodeal carinae: broadly separated, not parallel anteriorly. Setae in metasomal depression: present. Anterior sculpture of metasomal depression: absent. Median propodeal carina: absent.

T1 horn: absent. Number of longitudinal carinae of T1 midlobe: 5. T1 lateral carina: protruding laterally, visible from ventral view. T2 sculpture: with longitudinal striae or rugae, setiferous puncta present between them. T2 sublateral longitudinal foveae: absent. T3 metasomal flanges: absent. T4 sculpture: longitudinally striate to rugose, setal pits spanning interspaces. T4 metasomal flanges: present as slightly protruding sharp corners. T5 sculpture: longitudinally striate to rugose, setal pits spanning interspaces. T5 metasomal flanges: present as slightly protruding sharp corners. T6: broader than long. Major sculpture of T6: umbilicate punctate. Microsculpture of T6: absent. T6 medially: with a median projection set off by an abrupt narrowing posterior to tiny metasomal flanges, not separated from apical rim. T6 metasomal flanges: present subapically. T6 raised peripheral rim: absent. S4 sculpture: longitudinally striate or rugose, setal pits spanning interspaces. S5 sculpture: longitudinally striate to rugose, setal pits spanning interspaces. S5 median carina: present. S6 peripheral carina: absent. S6 apex in relation to T6: not exposed to dorsal view. S6 apex: rounded or acuminate.

*Male*. Body length 3.65–3.75 mm (n=2). A3: longer than pedicel. A5 tyloid shape: narrow, linear. A6: broader than long. A11: broader than long. Major sculpture of mesoscutal midlobe anteriorly: umbilicate foveate. Major sculpture of mesoscutal midlobe posteriorly: umbilicate foveate. Microsculpture of mesoscutal midlobe anteriorly: granulate. Microsculpture of mesoscutal midlobe posteriorly: absent. Major sculpture of mesoscutellum centrally: umbilicate foveate. Major sculpture of mesoscutellum peripherally: umbilicate foveate. Microsculpture of mesoscutellum centrally: absent. Microsculpture of mesoscutellum peripherally: absent. Fore wing apex at rest: reaching middle of T6. T1 midlobe longitudinal carinae: 4. T3 metasomal flanges: absent. T4 metasomal flanges: absent. T5 metasomal flanges: absent. T6 metasomal flanges: absent. T7: broadly and deeply emarginate, with rounded posterolateral margins.

##### Diagnosis.

Both sexes: Frontal depression deep; submedian carina distinct, sharp. Hyperoccipital carina indicated by rugae. Occipital carina complete, medially convex. Metascutellum concave dorsally, weakly emarginate apically, projecting dorsally. Coxa same color as most of femur, leg otherwise lighter in color. Postmarginal vein absent. Female: A3 not longer than pedicel. A4, A5 broader than long. Fore wing long enough to reach middle of T5. T1 midlobe with 5 longitudinal carinae. T4 with sharp corners, T5 with small sharp metasomal flanges, not otherwise abruptly narrower than preceding tergum. Main body of T6 not raised above apical rim, not abruptly separated from it; T6 with small and sharp subapical metasomal flanges. S6 not exposed to dorsal view. Male: A4, A11 broader than long. Fore wing long enough to reach middle of T6. T7 deeply emarginate, with broad posterolateral lobes. *Oxyscelio gressus* is very similar to *Oxyscelio cuspidis*, but females differ in having a shorter A3 and 4 T1 midlobe longitudinal carinae instead of 5. In *Oxyscelio cuspidis*, A3 is much longer than the pedicel, while in *Oxyscelio gressus* it is about the same length.

##### Etymology.

Latin 4th declension noun, genitive case, meaning “a step.”

##### Link to distribution map.

[http://hol.osu.edu/map-full.html?id=307081]

##### Material examined.

Holotype, female: AUSTRALIA: WA, 40km SE Armadale, Banksia-jarrah forest, Mount Cooke, 7.XII-22.XII.1990, malaise trap, A. D. Austin, OSUC 456176 (deposited in WAMP). Paratypes: AUSTRALIA: 1 female, 2 males, OSUC 456177-456179 (WINC).

#### Oxyscelio hamorum

Burks sp. n.

http://zoobank.org/BBC0038B-987B-417F-8119-8E46F39E7AF4

urn:lsid:biosci.ohio-state.edu:osuc_concepts:307082

http://species-id.net/wiki/Oxyscelio_hamorum

**Figures 133–136.**
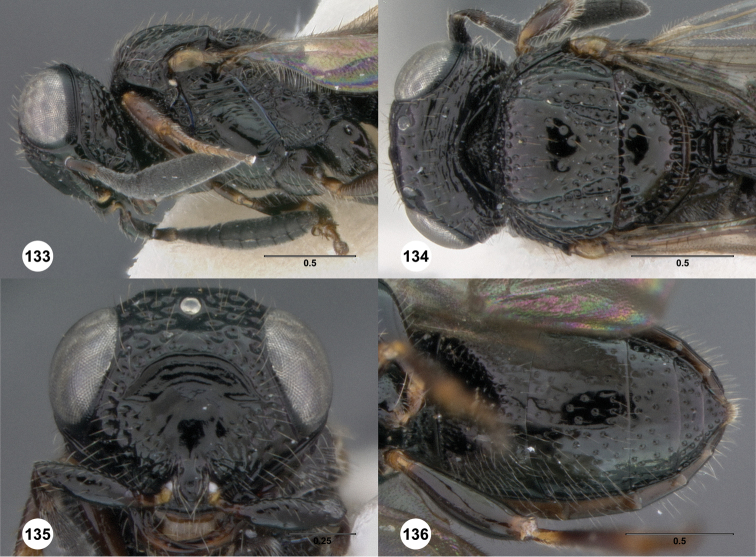
*Oxyscelio hamorum* sp. n., holotype female (OSUC 359626) **133** Head and mesosoma, lateral view **134** Head and mesosoma, dorsal view **135** Head, anterior view. Paratype female (462591) **136** Metasoma, ventral view. Morphbank^53^

Figures 133–136 ; Morphbank^53^

##### Description.

Female. Body length 3.2–3.45 mm (n=5).

Radicle color and shade: same as scape, both dark brown. Pedicel color: same as scape. A3: shorter than pedicel. A4: broader than long. A5: broader than long.

Ventral clypeal margin: with slightly convex median lobe. Interantennal process: not elongate. Lower frons at dorsal margin of interantennal process: without transverse carina. Transverse curved rugae extending from frontal depression to eye: absent. Median longitudinal carina in frontal depression: absent. Ventral portion of frontal depression: smooth. Dorsal portion of frontal depression: without transverse carinae. Submedian carina: present only as a weak shift in elevation. Frontal depression dorsally: not hood-like, open dorsally. Upper frons major sculpture: umbilicate foveate; irregularly rugose. Upper frons microsculpture: absent. Hyperoccipital carina: absent. Carina connecting occipital carina to hyperoccipital carina: absent. Occipital carina: present laterally, absent medially. Occiput sculpture: transversely rugose. Extra carina ventral to occipital carina: absent. Gena length: shorter than eye. Major sculpture of gena anteroventrally: umbilicate foveate. Major sculpture of gena posteroventrally: umbilicate foveate; umbilicate punctate; absent. Microsculpture of gena anteroventrally: absent. Microsculpture of gena posteroventrally: absent.

Lateral pronotal area sculpture: anteriorly smooth, posterodorsal corner with dense microsculpture, ventral corner with irregular carinae. Posterior border of central pronotal area: directed anteriorly, protruding at corner of epomial carina and transverse pronotal carina. Mesoscutum anteriorly: very steep and tall, descending at a right angle or protruding anteriorly. Major sculpture of mesoscutal midlobe anteriorly: umbilicate foveate. Mesoscutal midlobe sculpture at midlength: with large smooth areas. Major sculpture of mesoscutal midlobe posteriorly: umbilicate foveate. Microsculpture of mesoscutal midlobe anteriorly: absent. Microsculpture of mesoscutal midlobe posteriorly: absent. Median mesoscutal carina: present as a vague, occasionally interrupted elevation. Major sculpture of mesoscutellum centrally: absent. Major sculpture of mesoscutellum peripherally: umbilicate foveate. Microsculpture of mesoscutellum centrally: absent. Microsculpture of mesoscutellum peripherally: absent. Mesoscutellar rim: not expanded. Mesoscutellar rim medially: without notch. Mesofemoral depression: with slight, indistinct sculpture dorsally, smooth ventrally. Metascutellum shape: deeply emarginate, with the resulting pair of posterior processes subtriangular and directed dorsally. Metascutellar setae: absent. Metascutellum sculpture: with large smooth posterior fovea. Postmarginal vein: absent. Fore wing apex at rest: exceeding metasomal apex. Coxae color brightness: same color as femora. Spines along tibiae: present. Lateral propodeal carinae: broadly separated, but parallel for a short distance anteriorly. Setae in metasomal depression: absent. Anterior sculpture of metasomal depression: with median areole or pair of pits. Median propodeal carina: present.

T1 horn: absent. Number of longitudinal carinae of T1 midlobe: 3. T1 lateral carina: protruding laterally, visible from ventral view. T2 sculpture: densely foveolate, longitudinal sculpture irregular. T2 sublateral longitudinal foveae: absent. T3 metasomal flanges: absent. T4 sculpture: densely foveate, longitudinal sculpture irregular. T4 metasomal flanges: absent. T5 sculpture: densely foveate, interspaces not raised. T5 metasomal flanges: absent. T6: broader than long. Major sculpture of T6: umbilicate punctate. Microsculpture of T6: absent. T6 medially: with medially truncate emargination, sloping down to apical rim. T6 metasomal flanges: absent. T6 raised peripheral rim: absent. S4 sculpture: sparsely foveolate, with tiny pits in interspaces. S5 sculpture: sparsely foveate, with tiny pits in interspaces. S5 median carina: absent. S6 peripheral carina: absent. S6 apex in relation to T6: not exposed to dorsal view. S6 apex: rounded or acuminate.

*Male*. unknown.

##### Diagnosis.

Both sexes: Body entirely dark brown, including antennae and legs. Frontal depression shallow and very broad, transverse carinae weakly indicated above dorsal separator; submedian carina indicated by ruga. Hyperoccipital carina indicated by rugae. Occipital carina complete, sinuate or omicron-shaped medially. Metascutellum deeply concave, emarginate or truncate apically, projecting dorsally. Tibiae with flattened spines. T1 lateral carina expanded laterally. Metasomal sterna with few or no longitudinal rugae. Female: A3 much shorter than pedicel. A4, A5 broader than long. T1 midlobe with 4 longitudinal carinae. T6 without metasomal flanges. Fore wing long enough to reach beyond metasomal apex. Main body of T6 concave apically. *Oxyscelio hamorum* is very similar to *Oxyscelio uncinorum*, but has a broader frontal depression with some dorsal carinae, and has a much shorter A3. Both of these species have entirely dark brown bodies and tibial spines, but *Oxyscelio hamorum* resembles *Oxyscelio sordes* in other ways, which *Oxyscelio uncinorum* bears more resemblance to *Oxyscelio nitoris*.

##### Etymology.

Latin noun, genitive case, meaning “the barb of an arrow.”

##### Link to distribution map.

[http://hol.osu.edu/map-full.html?id=307082]

##### Associations.

Collected on *Eucalyptus stellulata* Sieber: [Myrtales: Myrtaceae]

##### Material examined.

Holotype, female: AUSTRALIA: TAS, 1km NE Herrick, 41°06'S, 147°53'E, 29.I–30.I.1983, I. D. Naumann & J. C. Cardale, OSUC 359626 (deposited in ANIC). Paratypes: AUSTRALIA: 4 females, OSUC 359623-359625 (ANIC); OSUC 462591 (CNCI).

#### Oxyscelio hyalinipennis

(Dodd)

http://zoobank.org/3546AB5F-12B5-4757-A529-95B8492A1455

urn:lsid:biosci.ohio-state.edu:osuc_concepts:5023

http://species-id.net/wiki/Oxyscelio_hyalinipennis

**Figures 137–142.**
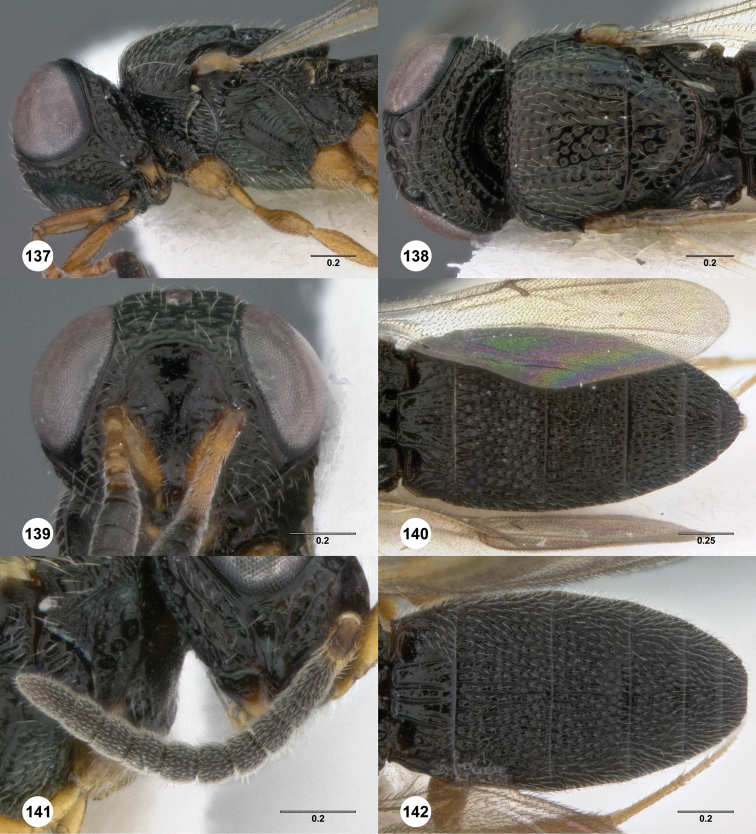
*Oxyscelio hyalinipennis* (Dodd), female (OSUC 437885) **137** Head and mesosoma, lateral view **138** Head and mesosoma, dorsal view **139** Head, anterior view **140** Metasoma, dorsal view. Male (OSUC 437979) **141** Antenna **142** Metasoma, dorsal view. Morphbank^54^

Figures 137–142 ; Morphbank^54^

Sceliomorpha hyalinipennis Dodd, 1913: 165 (original description); Kieffer 1926: 302, 307 (description, keyed).
Dicroteleia hyalinipennis (Dodd): Dodd 1914: 107 (generic transfer).
Oxyscelio hyalinipennis (Dodd): Dodd 1931: 75 (generic transfer); Galloway 1976: 99 (type information).

##### Description.

Female. Body length 2.6–3.85 mm (n=20).

Radicle color and shade: same as scape, both yellowish or reddish. Pedicel color: same as scape. A3: shorter than pedicel. A4: broader than long. A5: broader than long.

Ventral clypeal margin: concave. Interantennal process: not elongate. Lower frons at dorsal margin of interantennal process: with transverse ledge, face sharply receding below it. Transverse curved rugae extending from frontal depression to eye: absent. Median longitudinal carina in frontal depression: absent. Ventral portion of frontal depression: smooth. Dorsal portion of frontal depression: without transverse carinae. Submedian carina: absent. Frontal depression dorsally: not hood-like, open dorsally. Upper frons major sculpture: umbilicate foveate. Upper frons microsculpture: absent. Hyperoccipital carina: indicated by a set of irregular elevations. Carina connecting occipital carina to hyperoccipital carina: absent. Occipital carina: uniformly rounded dorsally. Occiput sculpture: umbilicate foveate medially, becoming smooth laterally. Extra carina ventral to occipital carina: absent. Gena length: shorter than eye. Major sculpture of gena anteroventrally: umbilicate foveate. Major sculpture of gena posteroventrally: umbilicate punctate; absent. Microsculpture of gena anteroventrally: absent. Microsculpture of gena posteroventrally: absent.

Lateral pronotal area sculpture: with shallow irregular carinae, posterodorsal corner with dense microsculpture. Posterior border of central pronotal area: directed posteriorly, epomial carina absent or meeting transverse pronotal carina at arch on lateral surface of pronotum. Mesoscutum anteriorly: very steep and tall, descending at a right angle or protruding anteriorly. Major sculpture of mesoscutal midlobe anteriorly: umbilicate foveate. Mesoscutal midlobe sculpture at midlength: not different from nearby sculpture. Major sculpture of mesoscutal midlobe posteriorly: umbilicate foveate; longitudinally rugose. Microsculpture of mesoscutal midlobe anteriorly: absent. Microsculpture of mesoscutal midlobe posteriorly: absent. Median mesoscutal carina: absent. Major sculpture of mesoscutellum centrally: umbilicate foveate. Major sculpture of mesoscutellum peripherally: umbilicate foveate. Microsculpture of mesoscutellum centrally: absent. Microsculpture of mesoscutellum peripherally: absent. Mesoscutellar rim: not expanded. Mesoscutellar rim medially: without notch. Mesofemoral depression: longitudinally striate dorsally, smooth ventrally. Metascutellum shape: not emarginate, concave but elevated posteriorly. Metascutellar setae: absent. Metascutellum sculpture: with large smooth posterior fovea. Spines along tibiae: absent. Lateral propodeal carinae: broadly separated, not parallel anteriorly. Setae in metasomal depression: absent. Anterior sculpture of metasomal depression: absent. Median propodeal carina: absent. Postmarginal vein: present. Fore wing apex at rest: exceeding metasomal apex; reaching middle of T6. Coxae color brightness: same color as femora.

T1 horn: absent. Number of longitudinal carinae of T1 midlobe: 5; obscured by other raised sculpture. T1 lateral carina: protruding laterally, visible from ventral view. T2 sculpture: with longitudinal striae or rugae, setiferous puncta present between them. T2 sublateral longitudinal foveae: absent. T3 metasomal flanges: absent. T4 sculpture: longitudinally striate to rugose, setal pits spanning interspaces. T4 metasomal flanges: absent. T5 sculpture: longitudinally striate to rugose, setal pits spanning interspaces. T5 metasomal flanges: absent. T6: broader than long. Major sculpture of T6: umbilicate punctate. Microsculpture of T6: absent. T6 medially: strongly convex, tapering and sloping down to a rounded apex, not separated from apical rim. T6 metasomal flanges: absent. T6 raised peripheral rim: absent. S4 sculpture: densely setose, setal pits between very weak longitudinal rugae. S5 sculpture: densely setose, setal pits between very weak longitudinal rugae. S5 median carina: absent. S6 peripheral carina: absent. S6 apex in relation to T6: not exposed to dorsal view. S6 apex: rounded or acuminate.

*Male*. Body length 2.45–3.85 mm (n=20). A3: longer than pedicel. A5 tyloid shape: narrow, linear. A6: broader than long. A11: broader than long. Major sculpture of mesoscutal midlobe anteriorly: umbilicate foveate. Major sculpture of mesoscutal midlobe posteriorly: umbilicate foveate. Microsculpture of mesoscutal midlobe anteriorly: granulate. Microsculpture of mesoscutal midlobe posteriorly: absent. Major sculpture of mesoscutellum centrally: umbilicate foveate. Major sculpture of mesoscutellum peripherally: umbilicate foveate. Microsculpture of mesoscutellum centrally: absent. Microsculpture of mesoscutellum peripherally: absent. Fore wing apex at rest: exceeding metasomal apex. T1 midlobe longitudinal carinae: 4. T3 metasomal flanges: absent. T4 metasomal flanges: absent. T5 metasomal flanges: absent. T6 metasomal flanges: absent. T7: weakly emarginate.

##### Diagnosis.

Both sexes: Frontal depression shallow; transverse carinae present ventrally, interrupted; submedian carina indicated by rounded ruga. Hyperoccipital carina indicated by rugae. Occipital carina complete, convex medially. Metascutellum deeply concave, not or hardly emarginate apically, projecting dorsally. Postmarginal vein present but sometimes very short. Coxa not darker than rest of leg. T1 lateral carina expanded laterally. Metasomal flanges absent. Female: A4, A5 broader than long. Mesoscutellum without granulate sculpture. T1 midlobe with 4 longitudinal carinae. Main body of T6 not abruptly separated from apical rim, T6 not concave apically. Fore wing long enough to reach middle of T6 or beyond metasomal apex. Male: Flagellomeres beyond A3 not or hardly longer than broad. T1 midlobe with 4 longitudinal carinae. Fore wing long enough to reach to or beyond metasomal apex. T7 tiny, truncate or very weakly emarginate.

##### Link to distribution map.

[http://hol.osu.edu/map-full.html?id=5023]

##### Associations.

Inhabits brigalow: [Fabales: Fabaceae]

##### Material examined.

Holotype, male, S. hyalinipennis: AUSTRALIA: QLD, tree foliage / forest, Gordonvale (Nelson), 30.IV.1913, A. P. Dodd, SAMA DB 32-001584 (deposited in SAMA). Other material: AUSTRALIA: 152 females, 73 males, ANIC DB 32-020077, 32-020078, 32-020119, 32-020120, OSUC 437877, OSUC 437879, OSUC 437882, OSUC 437886, OSUC 437891, OSUC 437971, OSUC 437972, OSUC 437973, OSUC 437974, OSUC 437979, OSUC 437980, OSUC 437981, OSUC 437982, OSUC 437983, OSUC 437986, OSUC 437990, OSUC 437991, OSUC 437994, OSUC 437995, OSUC 437996, OSUC 437997, OSUC 437998, OSUC 437999, OSUC 438002, OSUC 438003, OSUC 438019, OSUC 441298, OSUC 441300, OSUC 441301, OSUC 441308, OSUC 441311 (ANIC); OSUC 462602 (CNCI); OSUC 435938, 437887-437889, 437959, 437975, 437985, 437989, 451341-451345, QDPC 0-165625, QDPC 0-165626, QDPC 0-165629, QDPC 0-165633, QDPC 0-165637, QDPC 0-165640, QDPC 0-165643, QDPC 0-165661, QDPC 0-165664, QDPC 0-165672, QDPC 0-165673, QDPC 0-165759, QDPC 0-165761, QDPC 0-165762 (QDPC); OSUC 148477, 437885, 437978, 437993, 438024, 441309 (QMBA); OSUC 437005, 448605-448606, 448608-448609 (QPIM); UCRC ENT 151374 (UCRC); OSUC 437961-437966, 451361-451366 (UQIC); OSUC 437006-437009, 437880-437881, 437883-437884, 437890, 437892-437958, 437960, 437967-437970, 437976-437977, 437984, 437987-437988, 437992, 438000-438001, 438004-438018, 438020-438023, 438025-438038, 441293-441297, 441299, 441302-441307, 441310, 441312, 451346, 451367 (WINC).

##### Comments.

*Oxyscelio hyalinipennis* is a small-bodied member of the *atricoxa*-group with few distinctive features. Our concept of this species includes an unpublished Dodd species that he considered separate, differing from *Oxyscelio hyalinipennis* only in having a slightly larger body with a slightly longer metasoma. This variation is hypothesized by us to be the result of an increased body-size when parasitizing a slightly larger host.

#### Oxyscelio incisurae

Burks sp. n.

http://zoobank.org/4270F536-05E9-4F20-873D-CE8F455BD6D2

urn:lsid:biosci.ohio-state.edu:osuc_concepts:307083

http://species-id.net/wiki/Oxyscelio_incisurae

**Figures 143–148.**
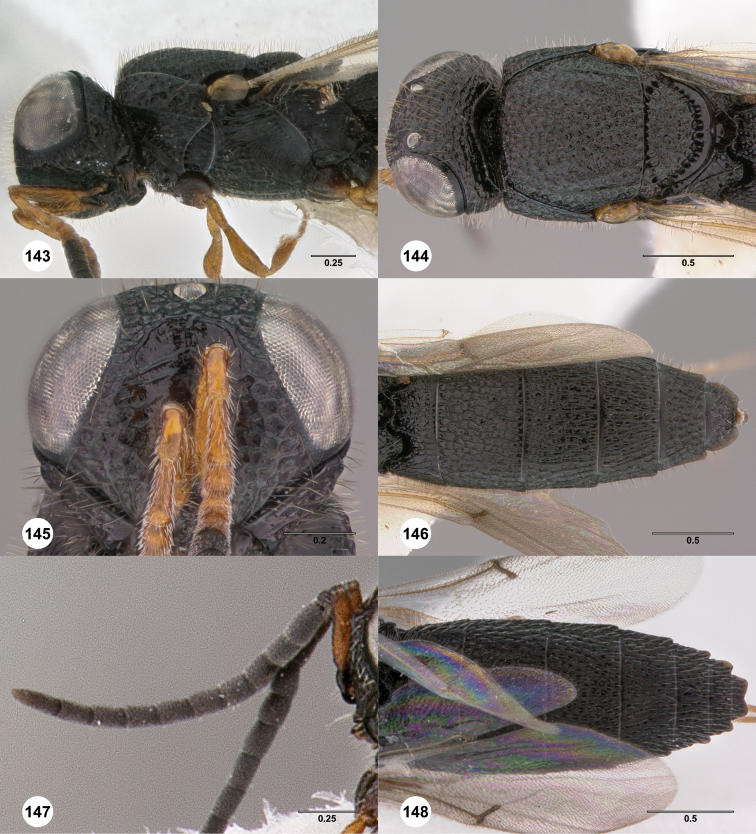
*Oxyscelio incisurae* sp. n., holotype female (OSUC 435942) **143** Head and mesosoma, lateral view **144** Head and mesosoma, dorsal view **145** Head, anterior view **146** Metasoma, dorsal view. Paratype male (OSUC 435949) **147** Antenna **148** Metasoma, dorsal view. Morphbank^55^

Figures 143–148 ; Morphbank^55^

##### Description.

Female. Body length 3.65–4.4 mm (n=11).

Radicle color and shade: darker than scape. Pedicel color: same as scape. A3: longer than pedicel. A4: broader than long. A5: broader than long.

Ventral clypeal margin: with slightly convex median lobe. Interantennal process: not elongate. Lower frons at dorsal margin of interantennal process: without transverse carina. Transverse curved rugae extending from frontal depression to eye: absent. Median longitudinal carina in frontal depression: absent. Ventral portion of frontal depression: with transverse carinae. Dorsal portion of frontal depression: with some transverse carinae. Submedian carina: present. Frontal depression dorsally: not hood-like, open dorsally. Upper frons major sculpture: umbilicate foveate. Upper frons microsculpture: absent. Hyperoccipital carina: absent. Carina connecting occipital carina to hyperoccipital carina: absent. Occipital carina: weakly arched dorsally, with rounded lateral corners. Occiput sculpture: irregularly sculptured. Extra carina ventral to occipital carina: absent. Gena length: shorter than eye. Major sculpture of gena anteroventrally: umbilicate foveate; rugose. Major sculpture of gena posteroventrally: umbilicate foveate; rugose. Microsculpture of gena anteroventrally: absent. Microsculpture of gena posteroventrally: absent.

Lateral pronotal area sculpture: irregularly foveate, with smooth area dorsally. Posterior border of central pronotal area: directed anteriorly, protruding at corner of epomial carina and transverse pronotal carina. Mesoscutum anteriorly: very steep and tall, descending at a right angle or protruding anteriorly. Major sculpture of mesoscutal midlobe anteriorly: umbilicate foveate. Mesoscutal midlobe sculpture at midlength: not different from nearby sculpture. Major sculpture of mesoscutal midlobe posteriorly: umbilicate foveate. Microsculpture of mesoscutal midlobe anteriorly: absent. Microsculpture of mesoscutal midlobe posteriorly: absent. Median mesoscutal carina: present as a flattened or rounded elevation. Major sculpture of mesoscutellum centrally: umbilicate foveate. Major sculpture of mesoscutellum peripherally: umbilicate foveate. Microsculpture of mesoscutellum centrally: absent. Microsculpture of mesoscutellum peripherally: absent. Mesoscutellar rim: expanded. Mesoscutellar rim medially: with notch. Mesofemoral depression: longitudinally striate dorsally, smooth ventrally. Metascutellum shape: not emarginate, concave but elevated posteriorly. Metascutellar setae: absent. Metascutellum sculpture: with large smooth posterior fovea. Postmarginal vein: absent. Fore wing apex at rest: reaching base of T5. Coxae color brightness: darker than femora. Spines along tibiae: absent. Lateral propodeal carinae: narrowly separated, angled anteriorly to become parallel. Setae in metasomal depression: absent. Anterior sculpture of metasomal depression: absent. Median propodeal carina: absent.

T1 horn: absent. Number of longitudinal carinae of T1 midlobe: 4. T1 lateral carina: protruding laterally, visible from ventral view. T2 sculpture: with longitudinal striae or rugae, setiferous puncta present between them. T2 sublateral longitudinal foveae: absent. T3 metasomal flanges: present. T4 sculpture: longitudinally striate to rugose, setal pits spanning interspaces. T4 metasomal flanges: present as slightly protruding sharp corners. T5 sculpture: longitudinally striate to rugose, setal pits spanning interspaces. T5 metasomal flanges: present, blade-like with rounded margins. T6: broader than long. Major sculpture of T6: longitudinally striate; umbilicate foveate. Microsculpture of T6: absent. T6 medially: with medially truncate emargination, separated from apical rim. T6 metasomal flanges: present, very broadly rounded but with sharp apices. T6 raised peripheral rim: absent. S4 sculpture: longitudinally striate or rugose, setal pits spanning interspaces. S5 sculpture: longitudinally striate to rugose, setal pits spanning interspaces. S5 median carina: present. S6 peripheral carina: present, posteriorly complete. S6 apex in relation to T6: exposed to dorsal view by T6 emargination. S6 apex: rounded or acuminate.

*Male*. Body length 3.85–3.9 mm (n=2). A3: longer than pedicel. A5 tyloid shape: narrow, linear. A6: longer than broad. A11: longer than broad. Major sculpture of mesoscutal midlobe anteriorly: umbilicate foveate. Major sculpture of mesoscutal midlobe posteriorly: umbilicate foveate. Microsculpture of mesoscutal midlobe anteriorly: absent. Microsculpture of mesoscutal midlobe posteriorly: absent. Major sculpture of mesoscutellum centrally: umbilicate foveate. Major sculpture of mesoscutellum peripherally: umbilicate foveate. Microsculpture of mesoscutellum centrally: absent. Microsculpture of mesoscutellum peripherally: absent. Fore wing apex at rest: reaching middle of T5. T1 midlobe longitudinal carinae: obscured by other raised sculpture. T3 metasomal flanges: present. T4 metasomal flanges: present. T5 metasomal flanges: present, rounded and lobe-like. T6 metasomal flanges: present, rounded and lobe-like. T7: M-shaped, with a triangular median emargination.

##### Diagnosis.

Both sexes: Frontal depression deep, all carinae except dorsal separator interrupted medially, carinae present above dorsal separator; submedian carina distinct but irregular medially. Hyperoccipital carina absent. Occipital carina complete, medially sinuate. Mesoscutal midlobe densely foveate. Mesoscutellar rim not expanded, with median notch. Metascutellum scoop-like, projecting dorsally. Coxa darker than rest of leg. Postmarginal vein absent. Female: A3 longer than pedicel. A4, A5 broader than long. T4 with weak metasomal flanges. T5 with lobe-like metasomal flanges. T6 with broad, wavy lateral metasomal flanges. T6 broadly and deeply emarginate, truncate medially. S6 exposed to dorsal view, rounded apically. Male: A3, A4, A11 longer than broad. Mesoscutellum and mesoscutal midlobe posteriorly with small and densely set foveae, mesoscutellum without smooth area medially. T5 with weak metasomal flanges, T6 with lobe-like metasomal flanges. T7 with broad postero-lateral lobes, deeply emarginate medially, posterior margin broadly M-shaped. *Oxyscelio incisurae* is very similar to *Oxyscelio funis* and *Oxyscelio bellariorum*, but differs in the short antenna and small and close-set mesoscutal midlobe foveae of females. Males may be more difficult to distinguish from similar species, but the long, dark flagellum in the only examined male was distinctive.

##### Etymology.

Latin noun, genitive case, meaning “incision.”

##### Link to distribution map.

[http://hol.osu.edu/map-full.html?id=307083]

##### Material examined.

Holotype, female: AUSTRALIA: SA, 14km WNW Renmark, Calperum Station, Bookmark Biosphere Reserve, 34°07'S, 140°37'E, 7.XI–13.XII.1995, flight intercept trap/gutter trap, K. R. Pullen, OSUC 435942 (deposited in ANIC). Paratypes: AUSTRALIA: 10 females, 2 males, OSUC 435939-435941, 435943-435949, 453998 (ANIC); OSUC 462737 (CNCI).

#### Oxyscelio lenitatis

Burks sp. n.

http://zoobank.org/7C5EFA9C-F6E5-4FC3-8162-1E815F93A45E

urn:lsid:biosci.ohio-state.edu:osuc_concepts:307084

http://species-id.net/wiki/Oxyscelio_lenitatis

**Figures 149–152.**
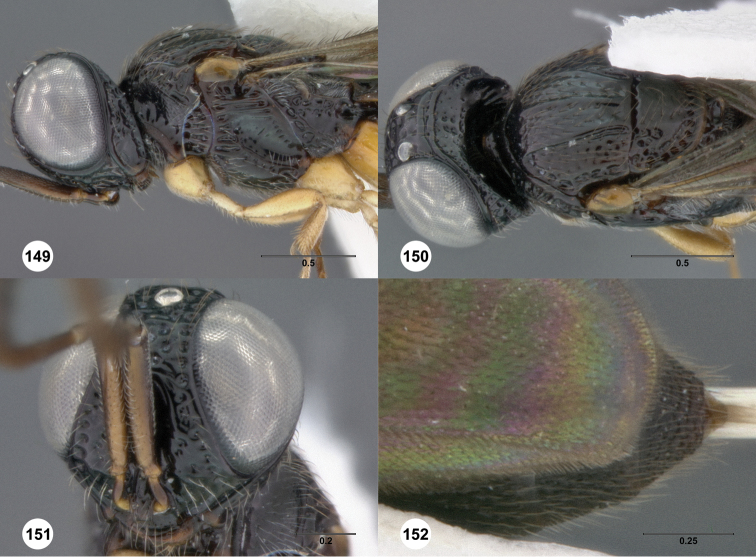
*Oxyscelio lenitatis* sp. n., holotype female (OSUC 368232) **149** Head and mesosoma, lateral view **150** Head and mesosoma, dorsal oblique view **151** Head, anterior view **152** Metasoma, dorsal view. Morphbank^56^

Figures 149–152 ; Morphbank^56^

##### Description.

Female. Body length 4 mm (n=1).

Radicle color and shade: same as scape, both dark brown. Pedicel color: same as scape. A3: longer than pedicel. A4: longer than broad. A5: broader than long.

Ventral clypeal margin: uniformly convex. Interantennal process: not elongate. Lower frons at dorsal margin of interantennal process: without transverse carina. Transverse curved rugae extending from frontal depression to eye: absent. Median longitudinal carina in frontal depression: absent. Ventral portion of frontal depression: with medially interrupted transverse carinae. Dorsal portion of frontal depression: without transverse carinae. Submedian carina: present only as a weak shift in elevation. Frontal depression dorsally: not hood-like, open dorsally. Upper frons major sculpture: umbilicate foveate; transversely rugose. Upper frons microsculpture: absent. Hyperoccipital carina: present as a single carina. Carina connecting occipital carina to hyperoccipital carina: absent. Occipital carina: present laterally, absent medially. Occiput sculpture: umbilicate punctate. Extra carina ventral to occipital carina: present, medially incomplete. Gena length: shorter than eye. Major sculpture of gena anteroventrally: umbilicate foveate; absent. Major sculpture of gena posteroventrally: umbilicate foveate; absent. Microsculpture of gena anteroventrally: absent. Microsculpture of gena posteroventrally: absent.

Lateral pronotal area sculpture: anteriorly smooth, posterodorsal corner with dense microsculpture, ventral corner with irregular carinae. Posterior border of central pronotal area: directed posteriorly, epomial carina absent or meeting transverse pronotal carina at arch on lateral surface of pronotum. Mesoscutum anteriorly: not steep, forming less than a right angle. Major sculpture of mesoscutal midlobe anteriorly: umbilicate punctate. Mesoscutal midlobe sculpture at midlength: not different from nearby sculpture. Major sculpture of mesoscutal midlobe posteriorly: umbilicate punctate. Microsculpture of mesoscutal midlobe anteriorly: granulate. Microsculpture of mesoscutal midlobe posteriorly: absent. Median mesoscutal carina: present as a vague, occasionally interrupted elevation. Major sculpture of mesoscutellum centrally: absent. Major sculpture of mesoscutellum peripherally: umbilicate punctate. Microsculpture of mesoscutellum centrally: absent. Microsculpture of mesoscutellum peripherally: absent. Mesoscutellar rim: not expanded. Mesoscutellar rim medially: without notch. Mesofemoral depression: with slight, indistinct sculpture dorsally, smooth ventrally. Metascutellum shape: slightly emarginate posteriorly, concave but elevated posteriorly. Metascutellar setae: absent. Metascutellum sculpture: with large smooth posterior fovea. Postmarginal vein: present. Fore wing apex at rest: reaching middle of T6. Coxae color brightness: same color as femora. Spines along tibiae: absent. Lateral propodeal carinae: narrowly separated, angled anteriorly to become parallel. Setae in metasomal depression: present. Anterior sculpture of metasomal depression: absent. Median propodeal carina: absent.

T1 horn: absent. Number of longitudinal carinae of T1 midlobe: unknown. T1 lateral carina: straight. T2 sculpture: with longitudinal striae or rugae, setiferous puncta present between them. T2 sublateral longitudinal foveae: absent. T3 metasomal flanges: absent. T4 sculpture: longitudinally striate to rugose, setal pits spanning interspaces. T4 metasomal flanges: absent. T5 sculpture: longitudinally striate to rugose, setal pits spanning interspaces. T5 metasomal flanges: absent. T6: broader than long. Major sculpture of T6: umbilicate punctate. Microsculpture of T6: absent. T6 medially: truncate, separated from apical rim. T6 metasomal flanges: absent. T6 raised peripheral rim: absent. S4 sculpture: longitudinally striate or rugose, setal pits spanning interspaces. S5 sculpture: longitudinally striate to rugose, setal pits spanning interspaces. S5 median carina: present. S6 peripheral carina: absent. S6 apex in relation to T6: not exposed to dorsal view. S6 apex: rounded or acuminate.

*Male*. unknown.

##### Diagnosis.

Both sexes: Frontal depression nearly flat, transverse carinae absent; submedian carina indicated by a weak ruga. Hyperoccipital carina indicated by rugae. Occipital carina absent medially, lateral corners not protruding. Metascutellum broad and short, concave. Postmarginal vein present. Coxa not darker than rest of leg. Metasomal depression setose. T1 lateral carina not expanded laterally. Metasomal flanges absent. Female: A3 much longer than pedicel. A4 about as long as broad, A5 broader than long. Mesoscutellum without granulate sculpture. Fore wing long enough to reach middle of T6. T6 broader than long, blunt apically with main body of tergum raised above apical rim.

##### Etymology.

Latin noun, genitive case, meaning “smoothness.”

##### Link to distribution map.

[http://hol.osu.edu/map-full.html?id=307084]

##### Material examined.

Holotype, female: AUSTRALIA: QLD, 3km ENE Mount Tozer, 12°44'S, 143°14'E, 28.VI–4.VII.1986, malaise trap, J. C. Cardale, OSUC 368232 (deposited in ANIC).

##### Comments.

*Oxyscelio lenitatis* is the only known Australian member of the otherwise Indonesian and Philippine *dasymesos*-group, characterized by a setose metasomal depression. It differs from other members of this group in having an apically rounded T6 in females in combination with a bare metascutellum.

#### Oxyscelio leviventris

Burks sp. n.

http://zoobank.org/3D711BFF-EFA8-49DE-B27E-5923F2D05BDC

urn:lsid:biosci.ohio-state.edu:osuc_concepts:307085

http://species-id.net/wiki/Oxyscelio_leviventris

**Figures 153–158.**
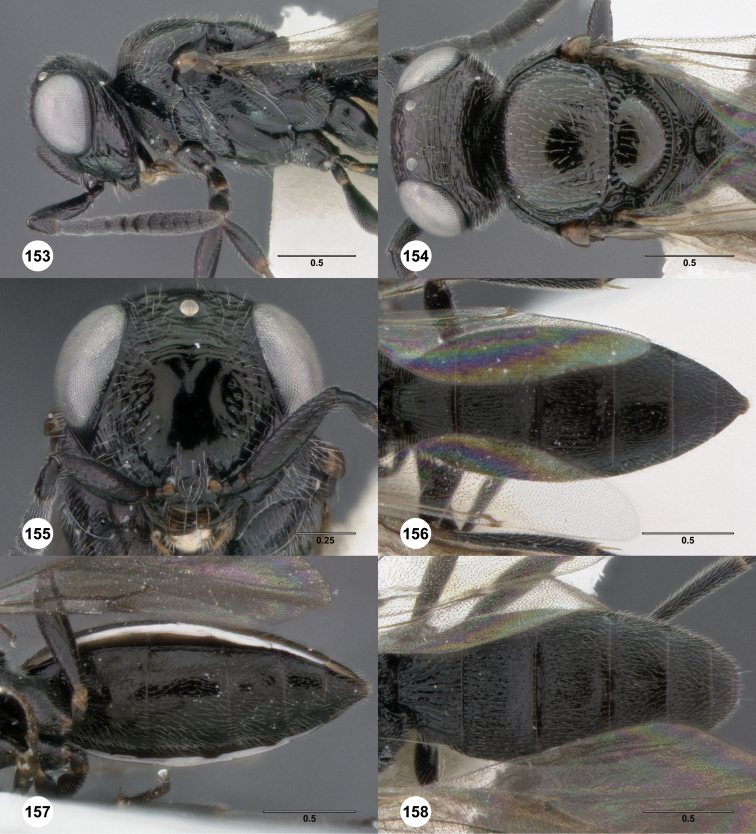
*Oxyscelio leviventris* sp. n., paratype female (OSUC 438829) **153** Head and mesosoma, lateral view **154** Head and mesosoma, dorsal view **155** Head, anterior view. Holotype female (OSUC 438828) **156** Metasoma, dorsal view **157** Metasoma, ventral view. Paratype male (OSUC 438832) **158** Metasoma, dorsal view. Morphbank^57^

Figures 153–158 ; Morphbank^57^

##### Description.

Female. Body length 3.5–4.25 mm (n=5).

Radicle color and shade: same as scape, both dark brown. Pedicel color: same as scape. A3: longer than pedicel. A4: longer than broad. A5: broader than long.

Ventral clypeal margin: with slightly convex median lobe. Interantennal process: not elongate. Lower frons at dorsal margin of interantennal process: without transverse carina. Transverse curved rugae extending from frontal depression to eye: absent. Median longitudinal carina in frontal depression: absent. Ventral portion of frontal depression: with medially interrupted transverse carinae. Dorsal portion of frontal depression: with medially interrupted transverse carinae. Submedian carina: present. Frontal depression dorsally: not hood-like, open dorsally. Upper frons major sculpture: umbilicate foveate; transversely rugose. Upper frons microsculpture: granulate. Hyperoccipital carina: indicated by a set of irregular elevations. Carina connecting occipital carina to hyperoccipital carina: absent. Occipital carina: weakly arched dorsally, with rounded lateral corners. Occiput sculpture: transversely rugose. Extra carina ventral to occipital carina: present, complete. Gena length: shorter than eye. Major sculpture of gena anteroventrally: rugose; umbilicate punctate. Major sculpture of gena posteroventrally: rugose; umbilicate punctate. Microsculpture of gena anteroventrally: absent. Microsculpture of gena posteroventrally: absent.

Lateral pronotal area sculpture: anteriorly smooth, posterodorsal corner with dense microsculpture, ventral corner with irregular carinae. Posterior border of central pronotal area: directed posteriorly, epomial carina absent or meeting transverse pronotal carina at arch on lateral surface of pronotum. Mesoscutum anteriorly: very steep and tall, descending at a right angle or protruding anteriorly. Major sculpture of mesoscutal midlobe anteriorly: umbilicate punctate. Mesoscutal midlobe sculpture at midlength: not different from nearby sculpture. Major sculpture of mesoscutal midlobe posteriorly: obliquely rugose. Microsculpture of mesoscutal midlobe anteriorly: granulate. Microsculpture of mesoscutal midlobe posteriorly: granulate. Median mesoscutal carina: absent. Major sculpture of mesoscutellum centrally: absent; umbilicate punctate. Major sculpture of mesoscutellum peripherally: umbilicate punctate. Microsculpture of mesoscutellum centrally: absent; granulate. Microsculpture of mesoscutellum peripherally: absent; granulate. Mesoscutellar rim: not expanded. Mesoscutellar rim medially: without notch. Mesofemoral depression: smooth. Metascutellum shape: not emarginate, concave but elevated posteriorly. Metascutellar setae: absent. Metascutellum sculpture: with large smooth posterior fovea. Postmarginal vein: present. Fore wing apex at rest: exceeding metasomal apex; reaching middle of T6. Coxae color brightness: same color as femora. Spines along tibiae: absent. Lateral propodeal carinae: broadly separated, but parallel for a short distance anteriorly. Setae in metasomal depression: absent. Anterior sculpture of metasomal depression: absent. Median propodeal carina: absent.

T1 horn: absent. Number of longitudinal carinae of T1 midlobe: more than 6. T1 lateral carina: straight. T2 sculpture: with longitudinal striae or rugae, setiferous puncta present between them. T2 sublateral longitudinal foveae: absent. T3 metasomal flanges: absent. T4 sculpture: mostly smooth, with tiny umbilicate foveae. T4 metasomal flanges: absent. T5 sculpture: mostly smooth, with tiny umbilicate foveae. T5 metasomal flanges: absent. T6: broader than long. Major sculpture of T6: umbilicate punctate. Microsculpture of T6: granulate. T6 medially: flat and tapering to a rounded apex, not separated from apical rim. T6 metasomal flanges: absent. T6 raised peripheral rim: absent. S4 sculpture: smooth, with tiny umbilicate foveae. S5 sculpture: smooth, with tiny umbilicate foveae. S5 median carina: absent. S6 peripheral carina: absent. S6 apex in relation to T6: not exposed to dorsal view. S6 apex: rounded or acuminate.

*Male*. Body length 3.6–3.95 mm (n=20). A3: longer than pedicel. A5 tyloid shape: narrow, linear. A6: broader than long. A11: longer than broad. Major sculpture of mesoscutal midlobe anteriorly: irregularly rugose; umbilicate punctate. Major sculpture of mesoscutal midlobe posteriorly: umbilicate punctate; obliquely rugose. Microsculpture of mesoscutal midlobe anteriorly: granulate. Microsculpture of mesoscutal midlobe posteriorly: granulate. Major sculpture of mesoscutellum centrally: umbilicate punctate. Major sculpture of mesoscutellum peripherally: umbilicate punctate. Microsculpture of mesoscutellum centrally: absent; granulate. Microsculpture of mesoscutellum peripherally: absent; granulate. Fore wing apex at rest: exceeding metasomal apex. T1 midlobe longitudinal carinae: 3. T3 metasomal flanges: absent. T4 metasomal flanges: absent. T5 metasomal flanges: absent. T6 metasomal flanges: absent. T7: weakly emarginate.

##### Diagnosis.

Both sexes: Body and limbs entirely dark brown. Frontal depression nearly flat, not crossed by carinae; submedian carina weakly indicated by some rugae. Clypeus not elongate. Hyperoccipital carina indicated by rugae. Occipital carina complete, irregular or weakly convex medially. Notauli absent. Mesoscutellum smooth, with only some scattered setae. Metascutellum broad and short, concave dorsally, rugose. Postmarginal vein present. Metasomal depression sculptured antero-medially. T1 lateral carina not expanded. Metasomal sterna without longitudinal rugae or carinae. Metasomal flanges absent. Female: A3 longer than pedicel. A5 broader than long. T1 midlobe with 5 or more longitudinal carinae. T6 broader than long, apical rim not separated from main body of tergum. Male: All flagellomeres longer than broad. T1 midlobe with 3 longitudinal carinae. T7 very broad and short, without posterior lobes.

##### Etymology.

Latin noun, genitive case, meaning “smooth-abdomen.”

##### Link to distribution map.

[http://hol.osu.edu/map-full.html?id=307085]

##### Material examined.

Holotype, female: AUSTRALIA: TAS, Bathurst Harbour, margin of copse / heathy sedgeland, Melaleuca, 43°25'S, 146°10'E, 12.II–17.II.1990, malaise trap, I. D. Naumann, OSUC 438828 (deposited in ANIC). Paratypes: AUSTRALIA: 4 females, 21 males, OSUC 438809, 438826-438827, 438829-438843, 438845-438849 (ANIC); OSUC 227562 (CNCI); OSUC 438844 (MVMA).

##### Comments.

*Oxyscelio leviventris* is similar to some members of the *flavipes*-group, as indicated by the usual loss of notauli and the smooth mesoscutellum. However, the occiput is roughly sculptured, and the frontal depression is very weak. Some other species in southeast Australia also exhibit dark coloration and reduced sculpture, but at least some of these may be convergent. While the absence of notauli may be striking in some specimens, this feature appears to be variable in this and some other species.

#### Oxyscelio limbi

Burks sp. n.

http://zoobank.org/7F866CF9-58A6-4972-94FA-81F30B203CDD

urn:lsid:biosci.ohio-state.edu:osuc_concepts:307086

http://species-id.net/wiki/Oxyscelio_limbi

**Figures 159–164.**
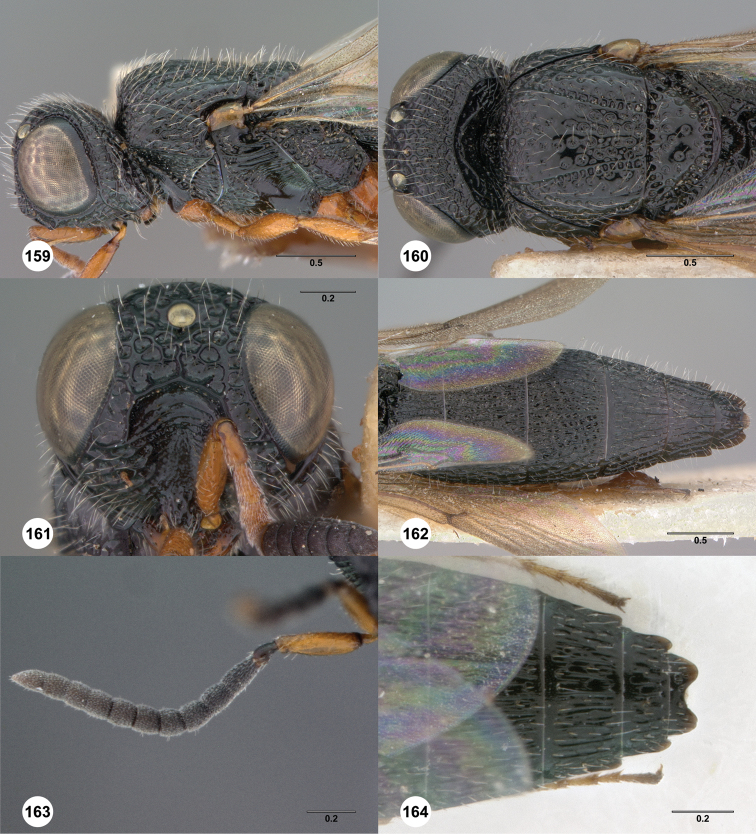
*Oxyscelio limbi* sp. n., paratype female (OSUC 436019) **159** Head and mesosoma, lateral view **160** Head and mesosoma, dorsal view **161** Head, anterior view **162** Metasoma, dorsal view. Paratype male (OSUC 376694) **163** Antenna **164** Metasoma, dorsal view. Morphbank^58^

Figures 159–164 ; Morphbank^58^

##### Description.

Female. Body length 9.1–11 mm (n=20).

Radicle color and shade: same as scape, both yellowish or reddish. Pedicel color: same as scape. A3: longer than pedicel. A4: broader than long. A5: broader than long.

Ventral clypeal margin: uniformly convex. Interantennal process: not elongate. Lower frons at dorsal margin of interantennal process: without transverse carina. Transverse curved rugae extending from frontal depression to eye: absent. Median longitudinal carina in frontal depression: absent. Ventral portion of frontal depression: with transverse carinae. Dorsal portion of frontal depression: without transverse carinae. Submedian carina: present. Frontal depression dorsally: hood-like, dorsally protruding. Upper frons major sculpture: umbilicate foveate. Upper frons microsculpture: absent. Hyperoccipital carina: absent. Carina connecting occipital carina to hyperoccipital carina: absent. Occipital carina: uniformly rounded dorsally. Occiput sculpture: irregularly sculptured. Extra carina ventral to occipital carina: present, complete. Gena length: shorter than eye. Major sculpture of gena anteroventrally: umbilicate foveate. Major sculpture of gena posteroventrally: umbilicate foveate. Microsculpture of gena anteroventrally: absent. Microsculpture of gena posteroventrally: absent.

Lateral pronotal area sculpture: with a series of arched carinae, posterodorsal corner with weak longitudinal rugae. Posterior border of central pronotal area: directed anteriorly, protruding at corner of epomial carina and transverse pronotal carina. Mesoscutum anteriorly: very steep and tall, descending at a right angle or protruding anteriorly. Major sculpture of mesoscutal midlobe anteriorly: umbilicate foveate; transversely rugose. Mesoscutal midlobe sculpture at midlength: not different from nearby sculpture. Major sculpture of mesoscutal midlobe posteriorly: umbilicate foveate. Microsculpture of mesoscutal midlobe anteriorly: granulate. Microsculpture of mesoscutal midlobe posteriorly: absent. Median mesoscutal carina: present as a vague, occasionally interrupted elevation. Major sculpture of mesoscutellum centrally: absent; umbilicate foveate. Major sculpture of mesoscutellum peripherally: umbilicate foveate. Microsculpture of mesoscutellum centrally: absent; punctate. Microsculpture of mesoscutellum peripherally: punctate. Mesoscutellar rim: expanded. Mesoscutellar rim medially: without notch. Mesofemoral depression: longitudinally striate dorsally, smooth ventrally. Metascutellum shape: not emarginate, concave but elevated posteriorly. Metascutellar setae: absent. Metascutellum sculpture: with large smooth posterior fovea. Postmarginal vein: absent. Fore wing apex at rest: not reaching base of T5. Coxae color brightness: same color as femora. Spines along tibiae: absent. Lateral propodeal carinae: broadly separated, not parallel anteriorly. Setae in metasomal depression: absent. Anterior sculpture of metasomal depression: absent. Median propodeal carina: absent.

T1 horn: absent. Number of longitudinal carinae of T1 midlobe: 4. T1 lateral carina: protruding laterally, visible from ventral view. T2 sculpture: with longitudinal striae or rugae, setiferous puncta present between them. T2 sublateral longitudinal foveae: absent. T3 metasomal flanges: present. T4 sculpture: longitudinally striate to rugose, setal pits spanning interspaces. T4 metasomal flanges: present and strongly protruding. T5 sculpture: longitudinally striate to rugose, setal pits spanning interspaces. T5 metasomal flanges: present as strongly protruding acuminate flanges. T6: broader than long. Major sculpture of T6: umbilicate punctate; longitudinally striate; umbilicate foveate. Microsculpture of T6: absent. T6 medially: with deep emargination that is V-shaped medially, separated from apical rim. T6 metasomal flanges: present, very broadly rounded, with rounded apices. T6 raised peripheral rim: absent. S4 sculpture: longitudinally striate or rugose, setal pits spanning interspaces. S5 sculpture: longitudinally striate to rugose, setal pits spanning interspaces. S5 median carina: present. S6 peripheral carina: present, posteriorly complete. S6 apex in relation to T6: exposed to dorsal view by T6 emargination. S6 apex: truncate.

*Male*. Body length 4.3–4.75 mm (n=14). A3: longer than pedicel. A5 tyloid shape: narrow, linear. A6: broader than long. A11: longer than broad. Major sculpture of mesoscutal midlobe anteriorly: umbilicate foveate; transversely rugose. Major sculpture of mesoscutal midlobe posteriorly: umbilicate foveate. Microsculpture of mesoscutal midlobe anteriorly: granulate. Microsculpture of mesoscutal midlobe posteriorly: absent. Major sculpture of mesoscutellum centrally: umbilicate foveate. Major sculpture of mesoscutellum peripherally: umbilicate foveate. Microsculpture of mesoscutellum centrally: punctate. Microsculpture of mesoscutellum peripherally: punctate. Fore wing apex at rest: reaching middle of T5. T1 midlobe longitudinal carinae: 5. T3 metasomal flanges: absent. T4 metasomal flanges: absent. T5 metasomal flanges: present as sharp corners that do not protrude. T6 metasomal flanges: present, rounded and lobe-like. T7: M-shaped, with a triangular median emargination.

##### Diagnosis.

Both sexes: Frontal depression deep, all carinae complete medially; no carinae present above dorsal separator, submedian carina sharp. Hyperoccipital carina absent. Occipital carina complete, medially weakly convex. Mesoscutellar rim expanded and strongly sculptured, without median notch. Metascutellum scoop-like, projecting dorsally. Coxa same color as rest of leg. Postmarginal vein absent. Female: A3 longer than pedicel. A5, A6 broader than long. T4, T5, T6 with broad, flat metasomal flanges. T6 apically broadly and deeply emarginate, angularly emarginate medially. S6 exposed to dorsal view, truncate apically. Male: A3 longer than broad. A4 broader than long, A11 as long or longer than broad. Mesoscutellum and mesoscutal midlobe posteriorly with small foveae, mesoscutellum with smooth area medially. T5, T6 with metasomal flanges. T7 with long postero-lateral lobes, deeply emarginate medially, posterior margin M-shaped.

##### Etymology.

Latin noun, genitive case, meaning “rim.”

##### Link to distribution map.

[http://hol.osu.edu/map-full.html?id=307086]

##### Material examined.

Holotype, female: AUSTRALIA: NSW, Fowlers Gap Research Station, 31°05'S, 141°42'E, 29.XI–2.XII.1981, J. C. Cardale, OSUC 436000 (deposited in ANIC). Paratypes: AUSTRALIA: 39 females, 14 males, Australian Museum K256282 (AMSA); OSUC 435995-435999, 436001-436003, 436009, 436011-436022, 436024-436029, 445337 (ANIC); OSUC 376685-376700, 376735 (MCZC); OSUC 436004, 436007 (MVMA); OSUC 448604 (QPIM); OSUC 436005 (UQIC); OSUC 449097-449099 (WINC).

##### Comments.

*Oxyscelio limbi* exhibits a variably expanded and sculptured mesoscutellar rim. This may be difficult to assess in some specimens, but identification can be verified using other discussed features.

#### Oxyscelio liminis

Burks sp. n.

http://zoobank.org/0DDF6836-8EED-4A60-ADEA-D708720CD508

urn:lsid:biosci.ohio-state.edu:osuc_concepts:307087

http://species-id.net/wiki/Oxyscelio_liminis

**Figures 165–168.**
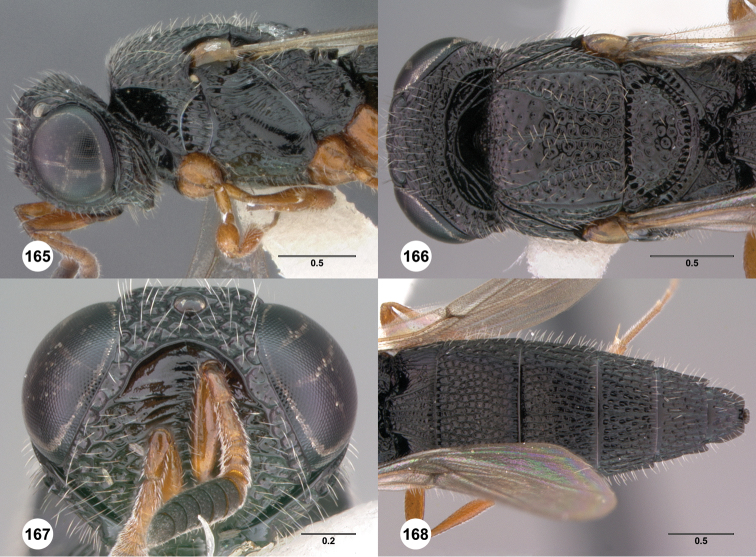
*Oxyscelio liminis* sp. n., holotype female (OSUC 376712) **165** Head and mesosoma, lateral view **166** Head and mesosoma, dorsal view **167** Head, anterior view **168** Metasoma, dorsal view. Morphbank^59^

Figures 165–168 ; Morphbank^59^

##### Description.

Female. Body length 3.75–5.65 mm (n=20).

Radicle color and shade: darker than scape. Pedicel color: same as scape. A3: longer than pedicel; as long as pedicel. A4: broader than long. A5: broader than long.

Ventral clypeal margin: uniformly convex. Interantennal process: not elongate. Lower frons at dorsal margin of interantennal process: without transverse carina. Transverse curved rugae extending from frontal depression to eye: absent. Median longitudinal carina in frontal depression: absent. Ventral portion of frontal depression: with transverse carinae. Dorsal portion of frontal depression: without transverse carinae. Submedian carina: present. Frontal depression dorsally: not hood-like, open dorsally. Upper frons major sculpture: umbilicate foveate. Upper frons microsculpture: absent. Hyperoccipital carina: absent. Carina connecting occipital carina to hyperoccipital carina: absent. Occipital carina: weakly arched dorsally, with rounded lateral corners. Occiput sculpture: irregularly sculptured. Extra carina ventral to occipital carina: absent. Gena length: shorter than eye. Major sculpture of gena anteroventrally: umbilicate foveate. Major sculpture of gena posteroventrally: umbilicate foveate; rugose. Microsculpture of gena anteroventrally: absent. Microsculpture of gena posteroventrally: absent.

Lateral pronotal area sculpture: with a series of arched carinae, posterodorsal corner with weak longitudinal rugae. Posterior border of central pronotal area: directed anteriorly, protruding at corner of epomial carina and transverse pronotal carina. Mesoscutum anteriorly: very steep and tall, descending at a right angle or protruding anteriorly. Major sculpture of mesoscutal midlobe anteriorly: umbilicate foveate. Mesoscutal midlobe sculpture at midlength: with large smooth areas. Major sculpture of mesoscutal midlobe posteriorly: umbilicate foveate. Microsculpture of mesoscutal midlobe anteriorly: absent; granulate. Microsculpture of mesoscutal midlobe posteriorly: absent; punctate. Median mesoscutal carina: present as a ruga. Major sculpture of mesoscutellum centrally: umbilicate foveate. Major sculpture of mesoscutellum peripherally: umbilicate foveate. Microsculpture of mesoscutellum centrally: punctate. Microsculpture of mesoscutellum peripherally: punctate. Mesoscutellar rim: not expanded. Mesoscutellar rim medially: without notch. Mesofemoral depression: longitudinally striate dorsally and ventrally. Metascutellum shape: not emarginate, concave but elevated posteriorly. Metascutellar setae: absent. Metascutellum sculpture: with large smooth posterior fovea. Postmarginal vein: absent. Fore wing apex at rest: not reaching base of T5. Coxae color brightness: same color as femora. Spines along tibiae: absent. Lateral propodeal carinae: broadly separated, not parallel anteriorly. Setae in metasomal depression: absent. Anterior sculpture of metasomal depression: absent. Median propodeal carina: absent.

T1 horn: absent. Number of longitudinal carinae of T1 midlobe: 6. T1 lateral carina: protruding laterally, visible from ventral view. T2 sculpture: with longitudinal striae or rugae, setiferous puncta present between them. T2 sublateral longitudinal foveae: absent. T3 metasomal flanges: present. T4 sculpture: longitudinally striate to rugose, setal pits spanning interspaces. T4 metasomal flanges: present and strongly protruding. T5 sculpture: longitudinally striate to rugose, setal pits spanning interspaces. T5 metasomal flanges: present as strongly protruding acuminate flanges. T6: broader than long. Major sculpture of T6: umbilicate punctate; longitudinally striate. Microsculpture of T6: absent. T6 medially: with deep emargination that is V-shaped medially, separated from apical rim. T6 metasomal flanges: present, very broadly rounded, with rounded apices. T6 raised peripheral rim: absent. S4 sculpture: longitudinally striate or rugose, setal pits spanning interspaces. S5 sculpture: longitudinally striate to rugose, setal pits spanning interspaces. S5 median carina: present. S6 peripheral carina: present, posteriorly complete. S6 apex in relation to T6: exposed to dorsal view by T6 emargination. S6 apex: truncate.

*Male*. Unknown.

##### Diagnosis.

Both sexes: Frontal depression deep, all carinae complete medially; no carinae present above dorsal separator, submedian carina sharp. Hyperoccipital carina absent. Occipital carina complete, medially weakly convex. Mesoscutellar rim not expanded, but sometimes rugose, without median notch. Metascutellum scoop-like, projecting dorsally. Coxa same color as rest of leg. Postmarginal vein absent. Female: A3 longer than pedicel. A4, A5, A6 broader than long. T4, T5, with sharp metasomal flanges. T6 with flat, expanded lateral margins. T6 apically shallowly emarginate, with a short incision medially. S6 exposed to dorsal view, truncate apically. *Oxyscelio liminis* is very similar to *Oxyscelio limbi*, but differs in having smaller, less rounded T6 metasomal flanges and a very shallowly and weakly emarginate T6. The mesoscutellar rim in *Oxyscelio liminis* is not expanded as in *Oxyscelio limbi*, but can have strong sculpture.

##### Etymology.

Latin noun, genitive case, meaning “apex.”

##### Link to distribution map.

[http://hol.osu.edu/map-full.html?id=307087]

##### Associations.

On flowering *Eucalyptus* L’Hér.: [Myrtales: Myrtaceae]; collected on *Eucalyptus populnea* F. Muell.: [Myrtales: Myrtaceae]; collected on *Eucalyptus redunca* Schauer: [Myrtales: Myrtaceae]

##### Material examined.

Holotype, female: AUSTRALIA: WA, Carnarvon Shire, Gascoyne Research Station, 3.X–7.X.1969, H. E. Evans & R. W. Matthews, OSUC 376712 (deposited in MCZC). Paratypes: AUSTRALIA: 66 females, OSUC 436006, 436010, 436041-436048, 436050, 436053, 437855, 437858-437859 (ANIC); OSUC 462738-462745 (CNCI); OSUC 376684, 376704-376705, 376707, 376710-376711, 376713-376715, 376722, 376733-376734 (MCZC); OSUC 436040 (MVMA); OSUC 148486 (QMBA); OSUC 448603 (QPIM); OSUC 148613-148614, 148616 (SAMA); OSUC 436039 (UQIC); OSUC 436038, 436049, 436051-436052, 437856, 449063-449080, 453990 (WINC).

#### Oxyscelio linguae

Burks sp. n.

http://zoobank.org/589E8E8E-4DFF-4B10-A832-CCBDF0A8E7DD

urn:lsid:biosci.ohio-state.edu:osuc_concepts:307126

http://species-id.net/wiki/Oxyscelio_linguae

**Figures 169–172.**
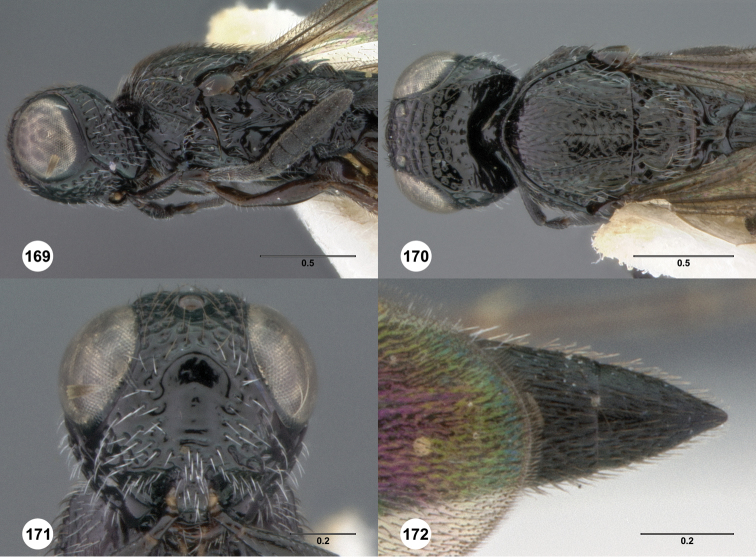
*Oxyscelio linguae* sp. n., holotype female (OSUC 448567) **169** Head and mesosoma, lateral view **170** Head and mesosoma, dorsal view **171** Head, anterior view **172** Metasoma, dorsal view. Morphbank^60^

Figures 169–172 ; Morphbank^60^

##### Description.

Female. Body length 4.1–4.2 mm (n=2).

Radicle color and shade: same as scape, both dark brown. Pedicel color: same as scape. A3: longer than pedicel. A4: broader than long. A5: broader than long.

Ventral clypeal margin: with slightly convex median lobe. Interantennal process: not elongate. Lower frons at dorsal margin of interantennal process: with oblique carina extending towards mouth corner. Transverse curved rugae extending from frontal depression to eye: absent. Median longitudinal carina in frontal depression: absent. Ventral portion of frontal depression: with transverse carinae. Dorsal portion of frontal depression: without transverse carinae. Submedian carina: present. Frontal depression dorsally: not hood-like, open dorsally. Upper frons major sculpture: umbilicate foveate; irregularly rugose. Upper frons microsculpture: absent. Hyperoccipital carina: indicated by a set of irregular elevations. Carina connecting occipital carina to hyperoccipital carina: absent. Occipital carina: present laterally, absent medially. Occiput sculpture: umbilicate foveate. Extra carina ventral to occipital carina: absent. Gena length: shorter than eye. Major sculpture of gena anteroventrally: umbilicate foveate. Major sculpture of gena posteroventrally: umbilicate foveate. Microsculpture of gena anteroventrally: punctate. Microsculpture of gena posteroventrally: punctate.

Lateral pronotal area sculpture: mostly granulate, ventral corner with irregular carinae. Posterior border of central pronotal area: directed posteriorly, epomial carina absent or meeting transverse pronotal carina at arch on lateral surface of pronotum. Mesoscutum anteriorly: not steep, forming less than a right angle. Major sculpture of mesoscutal midlobe anteriorly: umbilicate foveate. Mesoscutal midlobe sculpture at midlength: not different from nearby sculpture. Major sculpture of mesoscutal midlobe posteriorly: umbilicate foveate. Microsculpture of mesoscutal midlobe anteriorly: granulate. Microsculpture of mesoscutal midlobe posteriorly: absent. Median mesoscutal carina: present as a flattened or rounded elevation. Major sculpture of mesoscutellum centrally: umbilicate foveate. Major sculpture of mesoscutellum peripherally: umbilicate foveate. Microsculpture of mesoscutellum centrally: granulate. Microsculpture of mesoscutellum peripherally: granulate. Mesoscutellar rim: not expanded. Mesoscutellar rim medially: without notch. Mesofemoral depression: with slight, indistinct sculpture dorsally, smooth ventrally. Metascutellum shape: not emarginate, convex dorsally. Metascutellar setae: absent. Metascutellum sculpture: with many longitudinal rugae. Postmarginal vein: present. Fore wing apex at rest: reaching middle of T5. Coxae color brightness: same color as femora. Spines along tibiae: absent. Lateral propodeal carinae: broadly separated, not parallel anteriorly. Setae in metasomal depression: absent. Anterior sculpture of metasomal depression: unknown. Median propodeal carina: unknown.

T1 horn: present. Number of longitudinal carinae of T1 midlobe: obscured by other raised sculpture. T1 lateral carina: straight. T2 sculpture: with longitudinal striae or rugae, setiferous puncta present between them. T2 sublateral longitudinal foveae: absent. T3 metasomal flanges: absent. T4 sculpture: longitudinally striate to rugose, setal pits spanning interspaces. T4 metasomal flanges: absent. T5 sculpture: longitudinally striate to rugose, setal pits spanning interspaces. T5 metasomal flanges: absent. T6: longer than broad. Major sculpture of T6: umbilicate punctate; longitudinally striate. Microsculpture of T6: granulate. T6 medially: tapering to a sharp point, not separated from apical rim. T6 metasomal flanges: absent. T6 raised peripheral rim: absent. S4 sculpture: longitudinally striate or rugose, setal pits spanning interspaces. S5 sculpture: longitudinally striate to rugose, setal pits spanning interspaces. S5 median carina: present. S6 peripheral carina: absent. S6 apex in relation to T6: not exposed to dorsal view. S6 apex: rounded or acuminate.

*Male*. unknown.

##### Diagnosis.

Both sexes: Frontal depression concave, transverse carinae present ventrally; submedian carina weakly indicated by a single carina. Weak oblique carina extending from bottom of frontal depression towards lower margin of eye. Head directed downward. Hyperoccipital carina indicated by rugae. Occipital carina absent medially, with protruding lateral corners. Metascutellum tongue-like, very narrow, resting in long groove presented by propodeum medially. Postmarginal vein present. Coxa not darker than rest of leg. T1 lateral carina not expanded laterally. Metasomal flanges absent. Female: A3 longer than pedicel. A4 longer than broad, A5 broader than long. Mesoscutellum with granulate sculpture. T1 midlobe with strong anterior horn, longitudinal carinae obscured by raised area. Fore wing long enough to reach middle of T5. T6 longer than broad.

##### Etymology.

Latin noun, genitive case, meaning “tongue.”

##### Link to distribution map.

[http://hol.osu.edu/map-full.html?id=307126]

##### Material examined.

Holotype, female: PAPUA NEW GUINEA: Chimbu Prov., New Guinea Isl., Kerowagi, 25.III.1964, D. H. Colless, OSUC 448567 (deposited in ANIC). Paratype: PAPUA NEW GUINEA: 1 female, OSUC 448568 (ANIC).

##### Comments.

*Oxyscelio linguae* is unique in having a long, narrow metascutellum that rests in a groove caused by raised areas of the propodeum. In other respects it resembles species in the *foveatus*-group.

#### Oxyscelio lintris

Burks sp. n.

http://zoobank.org/F24E0251-2027-4282-B246-C6BF8FDB7C0D

urn:lsid:biosci.ohio-state.edu:osuc_concepts:307088

http://species-id.net/wiki/Oxyscelio_lintris

**Figures 173–176.**
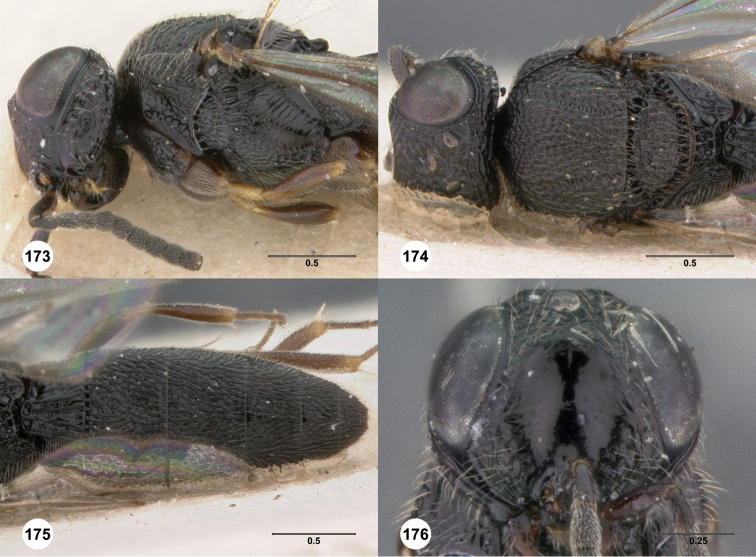
*Oxyscelio lintris* sp. n., holotype male (OSUC 453949) **173** Head and mesosoma, lateral view **174** Head and mesosoma, dorsal view **175** Metasoma, dorsal view. Paratype male (OSUC 453950) **176** Head, anterior view Morphbank^61^

Figures 173–176 ; Morphbank^61^

##### Description.

Female. unknown.

*Male*. Body length 4.1–4.15 mm (n=3).

Radicle color and shade: darker than scape. Pedicel color: same as scape. A3: longer than pedicel. A5 tyloid shape: narrow, linear. A6: broader than long. A11: longer than broad.

Ventral clypeal margin: with slightly convex median lobe. Interantennal process: not elongate. Lower frons at dorsal margin of interantennal process: without transverse carina. Transverse curved rugae extending from frontal depression to eye: absent. Median longitudinal carina in frontal depression: absent. Ventral portion of frontal depression: with medially interrupted transverse carinae. Dorsal portion of frontal depression: without transverse carinae. Submedian carina: present. Frontal depression dorsally: not hood-like, open dorsally. Upper frons major sculpture: umbilicate foveate; transversely rugose. Upper frons microsculpture: granulate. Hyperoccipital carina: indicated by a set of irregular elevations. Carina connecting occipital carina to hyperoccipital carina: absent. Occipital carina: broadly angular, with rounded median peak. Occiput sculpture: irregularly sculptured. Extra carina ventral to occipital carina: absent. Gena length: shorter than eye. Major sculpture of gena anteroventrally: umbilicate foveate. Major sculpture of gena posteroventrally: umbilicate foveate; rugose. Microsculpture of gena anteroventrally: absent. Microsculpture of gena posteroventrally: granulate.

Lateral pronotal area sculpture: anteriorly smooth, posterodorsal corner with dense microsculpture, ventral corner with irregular carinae. Posterior border of central pronotal area: directed posteriorly, epomial carina absent or meeting transverse pronotal carina at arch on lateral surface of pronotum. Mesoscutum anteriorly: not steep, forming less than a right angle. Major sculpture of mesoscutal midlobe anteriorly: umbilicate foveate. Major sculpture of mesoscutal midlobe posteriorly: umbilicate foveate. Microsculpture of mesoscutal midlobe anteriorly: granulate. Microsculpture of mesoscutal midlobe posteriorly: absent. Median mesoscutal carina: present as a ruga. Major sculpture of mesoscutellum centrally: umbilicate foveate. Major sculpture of mesoscutellum peripherally: umbilicate foveate. Microsculpture of mesoscutellum centrally: absent. Microsculpture of mesoscutellum peripherally: absent. Mesoscutellar rim: not expanded. Mesoscutellar rim medially: without notch. Mesofemoral depression: longitudinally striate dorsally, smooth ventrally. Metascutellum shape: not emarginate, concave but elevated posteriorly. Metascutellar setae: absent. Metascutellum sculpture: with many longitudinal rugae. Postmarginal vein: present. Fore wing apex at rest: reaching middle of T6. Coxae color brightness: same color as femora. Spines along tibiae: absent. Lateral propodeal carinae: broadly separated, not parallel anteriorly. Setae in metasomal depression: absent. Anterior sculpture of metasomal depression: absent. Median propodeal carina: absent.

T1 midlobe longitudinal carinae: 3. T3 metasomal flanges: absent. T4 metasomal flanges: absent. T5 metasomal flanges: absent. T6 metasomal flanges: absent. T7: broadly emarginate, with sharply pointed posterolateral lobes.

##### Diagnosis.

Male: A4, A11 longer than broad. Frontal depression moderately deep and mostly smooth, with interrupted transverse carinae ventrally and no carinae above dorsal separator; submedian carina sharp. Hyperoccipital carina represented by a set of rugae. Metascutellum subrectangular, with strong rugae. T1 midlobe with 3 complete longitudinal carinae. Fore wing long enough to reach middle of T6. T7 emarginate with tiny sharp posterolateral apices.

##### Etymology.

Latin noun, genitive case, meaning “trough.”

##### Link to distribution map.

[http://hol.osu.edu/map-full.html?id=307088]

##### Material examined.

Holotype, male: AUSTRALIA: NSW, Lord Howe Island, no date, A. M. Lea, OSUC 453949 (deposited in AMSA). Paratypes: AUSTRALIA: 2 males, OSUC 453950 (ANIC); OSUC 453951 (WINC).

##### Comments.

*Oxyscelio lintris* is an unusual species with a rugose metascutellum and short posterolateral corners on T7. This species is therefore of uncertain placement, but is provisionally placed in the *aciculae*-group because of its similarity to *Oxyscelio productionis*.

#### Oxyscelio livens

Burks sp. n.

http://zoobank.org/520356AA-7900-482F-9AEE-D40166420CA0

urn:lsid:biosci.ohio-state.edu:osuc_concepts:307089

http://species-id.net/wiki/Oxyscelio_livens

**Figures 177–182.**
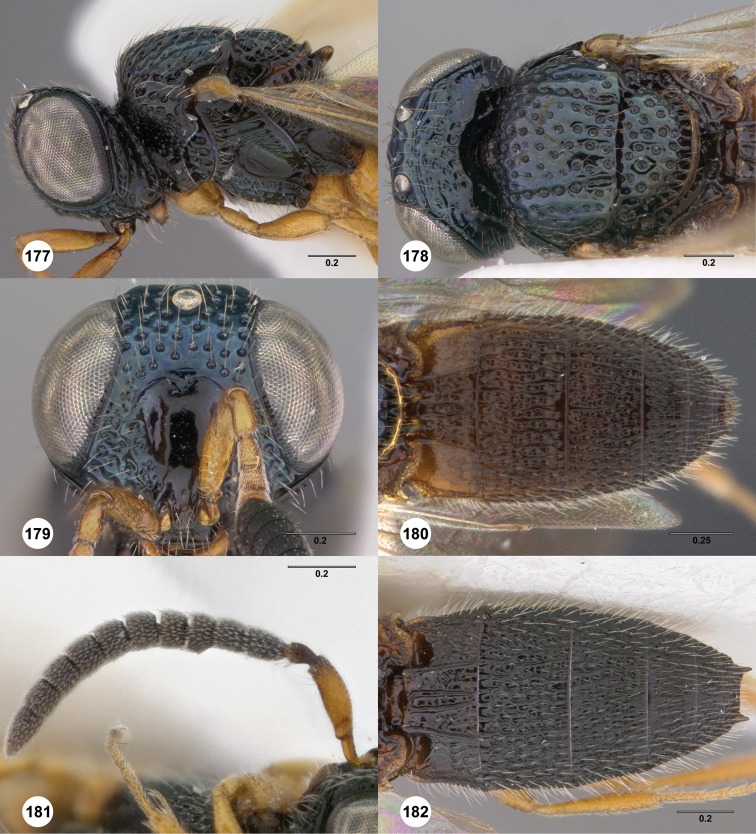
*Oxyscelio livens* sp. n., holotype female (OSUC 148485) **177** Head and mesosoma, lateral view **178** Head and mesosoma, dorsal view **179** Head, anterior view **180** Metasoma, dorsal view. Paratype male (OSUC 439598) **181** Antenna **182** Metasoma, dorsal view. Morphbank^62^

Figures 177–182 ; Morphbank^62^

##### Description.

Female. Body length 2.6–3.2 mm (n=20).

Radicle color and shade: same as scape, both yellowish or reddish. Pedicel color: same as scape. A3: shorter than pedicel. A4: broader than long. A5: broader than long.

Ventral clypeal margin: with slightly convex median lobe. Interantennal process: not elongate. Lower frons at dorsal margin of interantennal process: without transverse carina. Transverse curved rugae extending from frontal depression to eye: absent. Median longitudinal carina in frontal depression: absent. Ventral portion of frontal depression: smooth. Dorsal portion of frontal depression: without transverse carinae. Submedian carina: absent; present only as a weak shift in elevation. Frontal depression dorsally: not hood-like, open dorsally. Upper frons major sculpture: umbilicate foveate. Upper frons microsculpture: absent. Hyperoccipital carina: indicated by a set of irregular elevations. Carina connecting occipital carina to hyperoccipital carina: absent. Occipital carina: omicron-shaped, with sharp corners where median portion meets lateral portions. Occiput sculpture: transversely rugose. Extra carina ventral to occipital carina: absent. Gena length: shorter than eye. Major sculpture of gena anteroventrally: umbilicate foveate. Major sculpture of gena posteroventrally: umbilicate foveate; absent. Microsculpture of gena anteroventrally: absent. Microsculpture of gena posteroventrally: absent.

Lateral pronotal area sculpture: densely covered with setiferous puncta. Posterior border of central pronotal area: directed posteriorly, epomial carina absent or meeting transverse pronotal carina at arch on lateral surface of pronotum. Mesoscutum anteriorly: very steep and tall, descending at a right angle or protruding anteriorly. Major sculpture of mesoscutal midlobe anteriorly: umbilicate foveate. Mesoscutal midlobe sculpture at midlength: not different from nearby sculpture. Major sculpture of mesoscutal midlobe posteriorly: umbilicate foveate. Microsculpture of mesoscutal midlobe anteriorly: absent. Microsculpture of mesoscutal midlobe posteriorly: absent. Median mesoscutal carina: present as a vague, occasionally interrupted elevation. Major sculpture of mesoscutellum centrally: umbilicate foveate. Major sculpture of mesoscutellum peripherally: umbilicate foveate. Microsculpture of mesoscutellum centrally: punctate. Microsculpture of mesoscutellum peripherally: punctate. Mesoscutellar rim: expanded. Mesoscutellar rim medially: without notch. Mesofemoral depression: longitudinally striate dorsally, smooth ventrally. Metascutellum shape: slightly emarginate posteriorly, concave but elevated posteriorly. Metascutellar setae: absent. Metascutellum sculpture: with large smooth posterior fovea. Postmarginal vein: absent. Fore wing apex at rest: exceeding metasomal apex. Coxae color brightness: same color as femora. Spines along tibiae: absent. Lateral propodeal carinae: broadly separated, but parallel for a short distance anteriorly. Setae in metasomal depression: absent. Anterior sculpture of metasomal depression: absent. Median propodeal carina: absent.

T1 horn: absent. Number of longitudinal carinae of T1 midlobe: 4. T1 lateral carina: protruding laterally, visible from ventral view. T2 sculpture: with longitudinal striae or rugae, setiferous puncta present between them. T2 sublateral longitudinal foveae: absent. T3 metasomal flanges: absent. T4 sculpture: longitudinally striate to rugose, setal pits spanning interspaces. T4 metasomal flanges: present as slightly protruding sharp corners. T5 sculpture: longitudinally striate to rugose, setal pits spanning interspaces. T5 metasomal flanges: present as slightly protruding sharp corners. T6: broader than long. Major sculpture of T6: umbilicate punctate. Microsculpture of T6: absent. T6 medially: with broad emargination between protruding posterolateral corners, separated from apical rim. T6 metasomal flanges: present as spine-like structures posterolaterally. T6 raised peripheral rim: absent. S4 sculpture: longitudinally striate or rugose, setal pits spanning interspaces. S5 sculpture: longitudinally striate to rugose, setal pits spanning interspaces. S5 median carina: absent. S6 peripheral carina: absent. S6 apex in relation to T6: not exposed to dorsal view. S6 apex: rounded or acuminate.

*Male*. Body length 2.6–3.55 mm (n=20). A3: longer than pedicel. A5 tyloid shape: narrow, linear. A6: broader than long. A11: longer than broad. Major sculpture of mesoscutal midlobe anteriorly: umbilicate foveate. Major sculpture of mesoscutal midlobe posteriorly: umbilicate foveate. Microsculpture of mesoscutal midlobe anteriorly: granulate. Microsculpture of mesoscutal midlobe posteriorly: absent; granulate. Major sculpture of mesoscutellum centrally: umbilicate foveate. Major sculpture of mesoscutellum peripherally: umbilicate foveate. Microsculpture of mesoscutellum centrally: absent. Microsculpture of mesoscutellum peripherally: absent. Fore wing apex at rest: exceeding metasomal apex. T1 midlobe longitudinal carinae: 4. T3 metasomal flanges: absent. T4 metasomal flanges: absent. T5 metasomal flanges: absent. T6 metasomal flanges: present as sharp corners that do not protrude. T7: with a pair of sharply defined spine-like posterolateral projections.

##### Diagnosis.

Both sexes: Mesoscutum and mesoscutellum blue or green. Frontal depression shallow, transverse carinae absent; submedian carina absent or incomplete. Hyperoccipital carina indicated by strong rugae. Occipital carina complete, sinuate medially. Metascutellum deeply concave, emarginate apically, projecting dorsally. T1 lateral carina expanded laterally. Female: A3 not longer than pedicel. A4, A5 broader than long. T1 midlobe with 4 longitudinal carinae. T6 with narrow, sharp, subapical cornicle-like metasomal flanges. Fore wing long enough to reach middle of T6 or beyond metasomal apex. Main body of T6 not abruptly separated from apical rim. Male: A3 longer than pedicel. Most flagellomeres between A4 and A12 broader than long. T1 midlobe with 4 longitudinal carinae. Fore wing long enough to exceed metasomal apex. T7 with elongate spine-like posterior projections.

##### Etymology.

Latin participle, not changing with gender, meaning “envying.” Derived from the Latin word for blueness.

##### Link to distribution map.

[http://hol.osu.edu/map-full.html?id=307089]

##### Material examined.

Holotype, female: AUSTRALIA: N QLD, Red Road turnoff, rainforest, Tully Falls Road, 750m, 8.XII–5.I.1990, pitfall trap, Monteith, Thompson & Janetzki, OSUC 148485 (deposited in QMBA). Paratypes: AUSTRALIA: 24 females, 65 males, ANIC DB 32-020086, 32-020087, OSUC 439592, OSUC 439600, OSUC 439601, OSUC 439602, OSUC 439603, OSUC 439604, OSUC 439605, OSUC 439606, OSUC 439607, OSUC 439608, OSUC 439609, OSUC 439610, OSUC 439612, OSUC 439614, OSUC 439652, OSUC 439654 (ANIC); OSUC 451351-451352 (BMNH); OSUC 227565, 227621, 227626, 227635, 462755-462759 (CNCI); OSUC 439591, 439613, QDPC 0-165648, QDPC 0-165676, QDPC 0-165684, QDPC 0-165699, QDPC 0-165705, QDPC 0-165760, QDPC 0-165774 (QDPC); OSUC 148372, 148376, 439596-439599, 439611 (QMBA); OSUC 451348, 451350 (UQIC); OSUC 439593-439595, 439615-439638, 439640-439651, 439653, 451347, 451349 (WINC).

##### Comments.

*Oxyscelio livens* is a distinctive species because of the unusual spine-like subapical metasomal flanges of the females, and because of its usually metallic blue or green coloration. A few paratype females are shiny black instead of metallic, and some of these have slightly smaller T6 metasomal flanges than in other specimens. This species appears similar to some other small-bodied species such as *Oxyscelio nitoris* and *Oxyscelio palati*, which lack the T6 metasomal flanges.

#### Oxyscelio magniclava

(Dodd)

http://zoobank.org/A64FDA26-ED19-4E54-94EA-0ACDEF3C29EE

urn:lsid:biosci.ohio-state.edu:osuc_concepts:302870

http://species-id.net/wiki/Oxyscelio_magniclava

**Figures 183–186.**
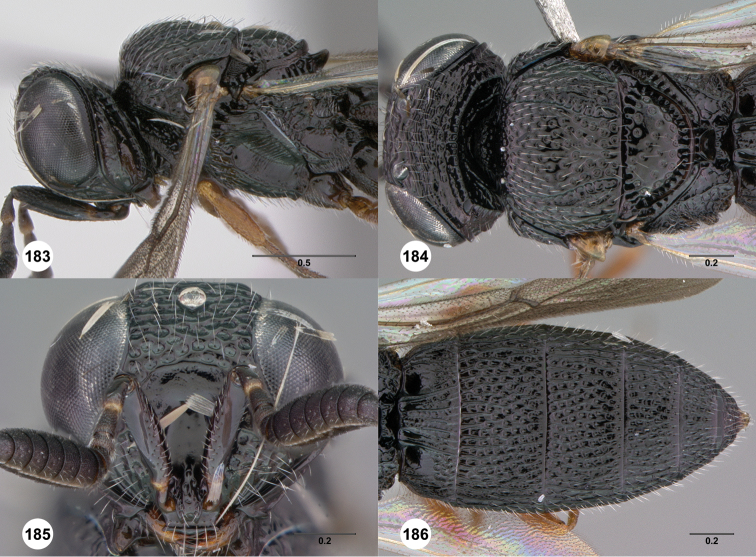
*Oxyscelio magniclava* (Dodd), female (OSUC 441388) **183** Head and mesosoma, lateral view **184** Head and mesosoma, dorsal view **185** Head, anterior view **186** Metasoma, dorsal view. Morphbank^63^

Figures 183–186 ; Morphbank^63^

Sceliomorpha magniclava Dodd, 1914: 103 (original description); Kieffer 1926: 303, 307 (description, keyed); Naumann et al. 1994: 71 (holotype transferred to ANIC).
Oxyscelio magniclavus (Dodd): Dodd 1931: 76 (generic transfer); Galloway 1976: 99 (type information).

##### Description.

Female. Body length 3.15–3.4 mm (n=3).

Radicle color and shade: same as scape, both dark brown. Pedicel color: same as scape. A3: shorter than pedicel; as long as pedicel. A4: broader than long. A5: broader than long.

Ventral clypeal margin: with slightly convex median lobe. Interantennal process: not elongate. Lower frons at dorsal margin of interantennal process: without transverse carina. Transverse curved rugae extending from frontal depression to eye: absent. Median longitudinal carina in frontal depression: absent. Ventral portion of frontal depression: with medially interrupted transverse carinae. Dorsal portion of frontal depression: without transverse carinae. Submedian carina: present. Frontal depression dorsally: not hood-like, open dorsally. Upper frons major sculpture: umbilicate foveate; transversely rugose. Upper frons microsculpture: absent. Hyperoccipital carina: indicated by a set of irregular elevations. Carina connecting occipital carina to hyperoccipital carina: absent. Occipital carina: weakly arched dorsally, with rounded lateral corners. Occiput sculpture: irregularly sculptured. Extra carina ventral to occipital carina: present, complete. Gena length: shorter than eye. Major sculpture of gena anteroventrally: umbilicate foveate. Major sculpture of gena posteroventrally: umbilicate foveate; absent. Microsculpture of gena anteroventrally: absent. Microsculpture of gena posteroventrally: granulate.

Lateral pronotal area sculpture: anteriorly smooth, posterodorsal corner with dense microsculpture, ventral corner with irregular carinae. Posterior border of central pronotal area: directed anteriorly, protruding at corner of epomial carina and transverse pronotal carina. Mesoscutum anteriorly: very steep and tall, descending at a right angle or protruding anteriorly. Major sculpture of mesoscutal midlobe anteriorly: umbilicate foveate. Mesoscutal midlobe sculpture at midlength: not different from nearby sculpture. Major sculpture of mesoscutal midlobe posteriorly: umbilicate foveate; irregularly rugose. Microsculpture of mesoscutal midlobe anteriorly: granulate. Microsculpture of mesoscutal midlobe posteriorly: absent. Median mesoscutal carina: present as a flattened or rounded elevation. Major sculpture of mesoscutellum centrally: umbilicate foveate. Major sculpture of mesoscutellum peripherally: umbilicate foveate. Microsculpture of mesoscutellum centrally: absent. Microsculpture of mesoscutellum peripherally: absent. Mesoscutellar rim: not expanded. Mesoscutellar rim medially: without notch. Mesofemoral depression: longitudinally striate dorsally, smooth ventrally. Metascutellum shape: slightly emarginate posteriorly, concave but elevated posteriorly. Metascutellar setae: absent. Metascutellum sculpture: with large smooth posterior fovea. Spines along tibiae: absent. Lateral propodeal carinae: broadly separated, but parallel for a short distance anteriorly. Setae in metasomal depression: absent. Anterior sculpture of metasomal depression: with median areole or pair of pits. Median propodeal carina: absent. Postmarginal vein: present. Fore wing apex at rest: reaching middle of T6. Coxae color brightness: darker than femora.

T1 horn: absent. Number of longitudinal carinae of T1 midlobe: 4. T1 lateral carina: protruding laterally, visible from ventral view. T2 sculpture: with longitudinal striae or rugae, setiferous puncta present between them. T2 sublateral longitudinal foveae: absent. T3 metasomal flanges: absent. T4 sculpture: longitudinally striate to rugose, setal pits spanning interspaces. T4 metasomal flanges: absent. T5 sculpture: longitudinally striate to rugose, setal pits spanning interspaces. T5 metasomal flanges: absent. T6: broader than long. Major sculpture of T6: umbilicate punctate. Microsculpture of T6: absent. T6 medially: flat and tapering to a rounded apex, not separated from apical rim. T6 metasomal flanges: absent. T6 raised peripheral rim: absent. S4 sculpture: longitudinally striate or rugose, setal pits spanning interspaces. S5 sculpture: longitudinally striate to rugose, setal pits spanning interspaces. S5 median carina: present. S6 peripheral carina: absent. S6 apex in relation to T6: not exposed to dorsal view. S6 apex: rounded or acuminate.

##### Diagnosis.

Both sexes: Frontal depression shallow and broad, not crossed by carinae; submedian carina very weak. Hyperoccipital carina indicated by rugae. Occipital carina complete, convex medially. Metascutellum deeply concave, truncate or slightly emarginate apically, projecting dorsally. Coxa darker than rest of leg. Postmarginal vein absent. T1 lateral carina expanded laterally. Metasomal flanges absent. Female: A3 not longer than pedicel. A4, A5, A6 broader than long. Mesoscutellum sparsely foveate, with broad smooth areas between foveae. Fore wing long enough to reach or exceed metasomal apex. T1 midlobe with 4 longitudinal carinae anteriorly. T6 broader than long, blunt or slightly concave apically. *Oxyscelio magniclava* is similar to *Oxyscelio nigricoxa*, but differs in its nearly smooth mesoscutellum. Males are unknown, but may be difficult to distinguish from those of *Oxyscelio nigricoxa*.

##### Link to distribution map.

[http://hol.osu.edu/map-full.html?id=302870]

##### Material examined.

Holotype, female, S. magniclava: AUSTRALIA: NSW, no date, ANIC DB 32-012479 (deposited in ANIC). Other material: AUSTRALIA: 3 females, 1 male, ANIC DB 32-020118, OSUC 439674, OSUC 441387, OSUC 441388 (ANIC).

#### Oxyscelio mirellus

(Dodd)

http://zoobank.org/466E4C8E-B0F7-4DD7-8FD7-A1A983FE1CB5

urn:lsid:biosci.ohio-state.edu:osuc_concepts:5028

http://species-id.net/wiki/Oxyscelio_mirellus

**Figures 187–192.**
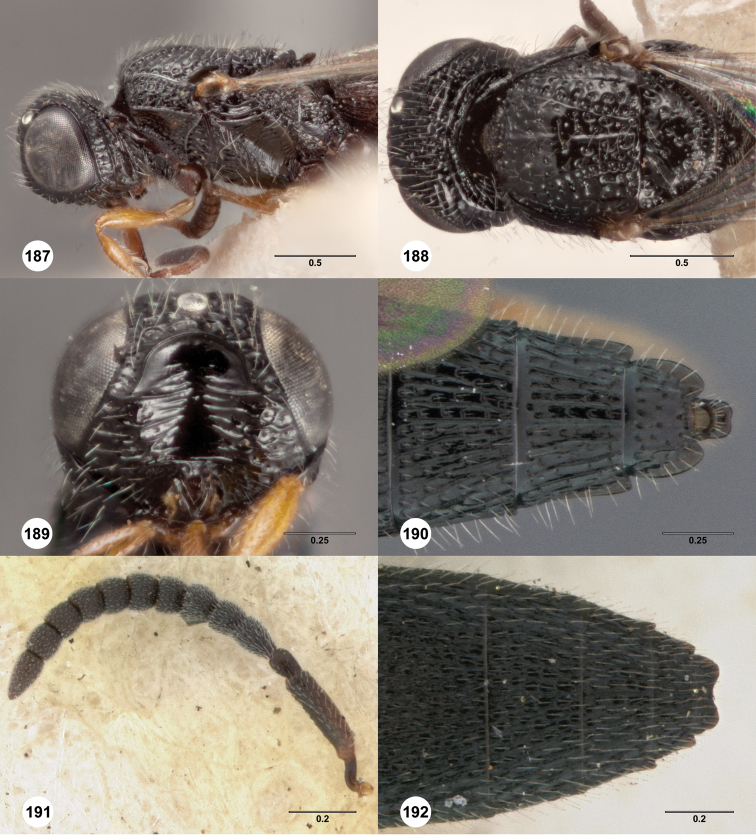
*Oxyscelio mirellus* (Dodd), female (B.M. TYPE HYM. 9.511) **187** Head and mesosoma, lateral view **188** Head and mesosoma, dorsal view **189** Head, anterior view. Female (OSUC 462747) **190** Metasomal apex, dorsal view. Paratype male (OSUC 462750) **191** Antenna **192** Metasoma, dorsal view. Morphbank^64^

Figures 187–192 ; Morphbank^64^

Sceliomorpha mirella Dodd, 1920: 349 (original description); Masner 1965: 96 (type information).
Oxyscelio mirellus (Dodd): Dodd 1931: 76 (generic transfer); Galloway 1976: 99 (type information); Masner 1976: 24 (description).

##### Description.

Female. Body length 4.2–4.55 mm (n=6).

Radicle color and shade: darker than scape. Pedicel color: same as scape. A3: longer than pedicel. A4: broader than long. A5: broader than long.

Ventral clypeal margin: with slightly convex median lobe. Interantennal process: not elongate. Lower frons at dorsal margin of interantennal process: without transverse carina. Transverse curved rugae extending from frontal depression to eye: absent. Median longitudinal carina in frontal depression: absent. Ventral portion of frontal depression: with medially interrupted transverse carinae. Dorsal portion of frontal depression: without transverse carinae. Submedian carina: present. Frontal depression dorsally: hood-like, dorsally protruding. Upper frons major sculpture: umbilicate foveate; irregularly rugose. Upper frons microsculpture: absent. Hyperoccipital carina: absent. Carina connecting occipital carina to hyperoccipital carina: absent. Occipital carina: broadly angular, with rounded median peak. Occiput sculpture: irregularly sculptured. Extra carina ventral to occipital carina: present, complete. Gena length: shorter than eye. Major sculpture of gena anteroventrally: umbilicate foveate. Major sculpture of gena posteroventrally: umbilicate foveate; rugose. Microsculpture of gena anteroventrally: absent. Microsculpture of gena posteroventrally: absent.

Lateral pronotal area sculpture: with shallow irregular carinae, without microsculpture. Posterior border of central pronotal area: directed anteriorly, protruding at corner of epomial carina and transverse pronotal carina. Mesoscutum anteriorly: very steep and tall, descending at a right angle or protruding anteriorly. Major sculpture of mesoscutal midlobe anteriorly: umbilicate foveate. Mesoscutal midlobe sculpture at midlength: not different from nearby sculpture. Major sculpture of mesoscutal midlobe posteriorly: umbilicate foveate. Microsculpture of mesoscutal midlobe anteriorly: granulate. Microsculpture of mesoscutal midlobe posteriorly: absent. Median mesoscutal carina: absent. Major sculpture of mesoscutellum centrally: umbilicate foveate. Major sculpture of mesoscutellum peripherally: umbilicate foveate; umbilicate punctate. Microsculpture of mesoscutellum centrally: punctate. Microsculpture of mesoscutellum peripherally: punctate. Mesoscutellar rim: not expanded. Mesoscutellar rim medially: without notch. Mesofemoral depression: longitudinally striate dorsally and ventrally. Metascutellum shape: not emarginate, concave but elevated posteriorly. Metascutellar setae: absent. Metascutellum sculpture: with large smooth posterior fovea. Spines along tibiae: absent. Lateral propodeal carinae: broadly separated, not parallel anteriorly. Setae in metasomal depression: absent. Anterior sculpture of metasomal depression: absent. Median propodeal carina: absent. Postmarginal vein: absent, but marginal vein curving slightly at apex. Fore wing apex at rest: not reaching base of T5. Coxae color brightness: darker than femora.

T1 horn: absent. Number of longitudinal carinae of T1 midlobe: 4. T1 lateral carina: protruding laterally, visible from ventral view. T2 sculpture: with longitudinal striae or rugae, setiferous puncta present between them. T2 sublateral longitudinal foveae: absent. T3 metasomal flanges: present. T4 sculpture: longitudinally striate to rugose, setal pits spanning interspaces. T4 metasomal flanges: present as slightly protruding sharp corners. T5 sculpture: longitudinally striate to rugose, setal pits spanning interspaces. T5 metasomal flanges: present as slightly protruding sharp corners. T6: broader than long. Major sculpture of T6: umbilicate punctate; longitudinally striate. Microsculpture of T6: absent. T6 medially: with medially truncate emargination, separated from apical rim. T6 metasomal flanges: present, very broadly rounded but with sharp apices. T6 raised peripheral rim: absent. S4 sculpture: longitudinally striate or rugose, setal pits spanning interspaces. S5 sculpture: longitudinally striate to rugose, setal pits spanning interspaces. S5 median carina: present. S6 peripheral carina: present, posteriorly complete. S6 apex in relation to T6: exposed to dorsal view by T6 emargination. S6 apex: truncate.

*Male*. Body length 3–3.9 mm (n=20). A3: longer than pedicel. A5 tyloid shape: narrow, linear. A6: broader than long. A11: as long as broad. Major sculpture of mesoscutal midlobe anteriorly: umbilicate foveate. Major sculpture of mesoscutal midlobe posteriorly: umbilicate foveate. Microsculpture of mesoscutal midlobe anteriorly: granulate. Microsculpture of mesoscutal midlobe posteriorly: absent. Major sculpture of mesoscutellum centrally: umbilicate foveate. Major sculpture of mesoscutellum peripherally: umbilicate foveate. Microsculpture of mesoscutellum centrally: absent. Microsculpture of mesoscutellum peripherally: absent. Fore wing apex at rest: reaching apex of T5. T1 midlobe longitudinal carinae: 4. T3 metasomal flanges: absent. T4 metasomal flanges: absent. T5 metasomal flanges: present as slightly protruding sharp corners. T6 metasomal flanges: present as slightly protruding acuminate flanges. T7: broadly and deeply emarginate, with rounded posterolateral margins.

##### Diagnosis.

Both sexes: Frontal depression deep, all carinae except dorsal separator interrupted medially, no carinae present above dorsal separator; submedian carina sharp. Hyperoccipital carina absent. Occipital carina complete, medially weakly convex or forming a vague median peak. Mesoscutellar rim not expanded, without median notch. Metascutellum scoop-like, projecting dorsally. Coxa darker than rest of leg. Postmarginal vein absent. Female: A3 not longer than pedicel. A4, A5, A6 broader than long. T4 with tiny metasomal flanges; T5, T6 with broad, flat metasomal flanges. T6 apically broadly and deeply emarginate, truncate medially. S6 exposed to dorsal view, truncate apically. Male: A3 longer than broad. A4, A11 broader than long. Mesoscutellum and mesoscutal midlobe posteriorly with broad foveae, not smooth medially. T5, T6 with sharp metasomal flanges. T7 with rounded postero-lateral lobes, broadly emarginate medially.

##### Link to distribution map.

[http://hol.osu.edu/map-full.html?id=5028]

##### Associations.

On flower of mallee: [Myrtales: Myrtaceae]

##### Material examined.

Holotype, female, S. mirella: AUSTRALIA: WA, Yallingup, 1.XII-12.XII.1913, R. E. Turner, B.M. TYPE HYM. 9.511 (deposited in BMNH). Paratypes: AUSTRALIA: 1 female, 4 males, OSUC 435935 (ANIC); OSUC 462562, 462750-462752 (BMNH). Other material: AUSTRALIA: 6 females, 33 males, OSUC 462748 (ANIC); OSUC 449030-449031, 449037-449044, 449049-449055, 449058-449062 (BMNH); OSUC 462746-462747 (CNCI); OSUC 435936 (QDPC); OSUC 449045-449048, 449057 (UQIC); WAM Ent. Reg No. 70285 (WAMP); OSUC 449032-449036, 449056, 462749 (WINC).

##### Comments.

*Oxyscelio mirellus* is distinctive in having a very broadly and deeply emarginate T6 with broad metasomal flanges, in combination with short flagellomeres in both sexes. South Australian specimens are slightly smaller than those from Western Australia, but otherwise seemed conspecific.

#### Oxyscelio montanus

(Dodd)

http://zoobank.org/E7DA5E79-CA36-47B4-A0BE-9FF9B6842A06

urn:lsid:biosci.ohio-state.edu:osuc_concepts:5029

http://species-id.net/wiki/Oxyscelio_montanus

**Figures 193–198.**
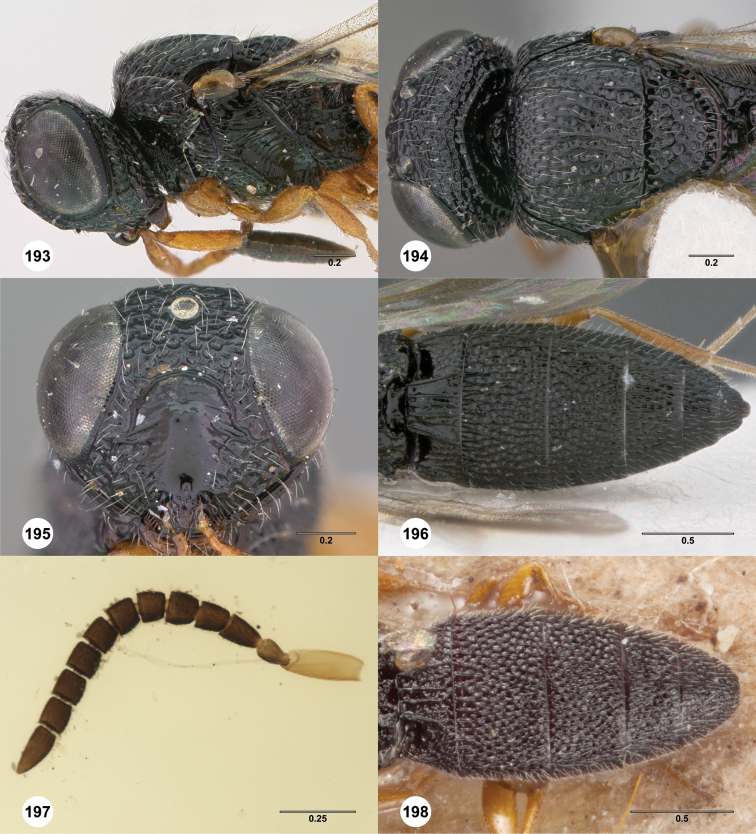
*Oxyscelio montanus* (Dodd), female (OSUC 441261) **193** Head and mesosoma, lateral view **194** Head and mesosoma, dorsal view **195** Head, anterior view **196** Metasoma, dorsal view. Holotype male (SAMA DB 32-001586) **197** Antenna **198** Metasoma, dorsal view. Morphbank^65^

Figures 193–198 ; Morphbank^65^

Sceliomorpha montana Dodd, 1913: 165 (original description); Kieffer 1926: 302, 307 (description, keyed).
Dicroteleia montana (Dodd): Dodd 1914: 107 (generic transfer).
Oxyscelio montanus (Dodd): Dodd 1931: 76 (generic transfer); Galloway 1976: 99 (type information).

##### Description.

Female. Body length 3.1–4.55 mm (n=20).

Radicle color and shade: same as scape, both yellowish or reddish; darker than scape. Pedicel color: same as scape. A3: shorter than pedicel. A4: broader than long. A5: broader than long.

Ventral clypeal margin: with slightly convex median lobe. Interantennal process: not elongate. Lower frons at dorsal margin of interantennal process: without transverse carina. Transverse curved rugae extending from frontal depression to eye: absent. Median longitudinal carina in frontal depression: absent. Ventral portion of frontal depression: smooth. Dorsal portion of frontal depression: with medially interrupted transverse carinae. Submedian carina: present. Frontal depression dorsally: not hood-like, open dorsally. Upper frons major sculpture: umbilicate foveate; irregularly rugose. Upper frons microsculpture: absent. Hyperoccipital carina: indicated by a set of irregular elevations. Carina connecting occipital carina to hyperoccipital carina: absent. Occipital carina: weakly arched dorsally, with rounded lateral corners. Occiput sculpture: irregularly sculptured. Extra carina ventral to occipital carina: present, complete. Gena length: shorter than eye. Major sculpture of gena anteroventrally: umbilicate foveate. Major sculpture of gena posteroventrally: umbilicate foveate; umbilicate punctate. Microsculpture of gena anteroventrally: absent. Microsculpture of gena posteroventrally: granulate.

Lateral pronotal area sculpture: anteriorly smooth, posterodorsal corner with dense microsculpture, ventral corner with irregular carinae. Posterior border of central pronotal area: directed anteriorly, protruding at corner of epomial carina and transverse pronotal carina. Mesoscutum anteriorly: very steep and tall, descending at a right angle or protruding anteriorly. Major sculpture of mesoscutal midlobe anteriorly: umbilicate foveate. Mesoscutal midlobe sculpture at midlength: not different from nearby sculpture. Major sculpture of mesoscutal midlobe posteriorly: umbilicate foveate. Microsculpture of mesoscutal midlobe anteriorly: granulate. Microsculpture of mesoscutal midlobe posteriorly: absent. Median mesoscutal carina: present as a vague, occasionally interrupted elevation. Major sculpture of mesoscutellum centrally: umbilicate foveate; longitudinally rugose. Major sculpture of mesoscutellum peripherally: umbilicate foveate; longitudinally rugose. Microsculpture of mesoscutellum centrally: absent. Microsculpture of mesoscutellum peripherally: absent. Mesoscutellar rim: not expanded. Mesoscutellar rim medially: without notch. Mesofemoral depression: longitudinally striate dorsally, smooth ventrally. Metascutellum shape: slightly emarginate posteriorly, concave but elevated posteriorly. Metascutellar setae: absent. Metascutellum sculpture: with large smooth posterior fovea. Spines along tibiae: absent. Lateral propodeal carinae: broadly separated, but parallel for a short distance anteriorly. Setae in metasomal depression: absent. Anterior sculpture of metasomal depression: absent. Median propodeal carina: absent. Postmarginal vein: absent. Fore wing apex at rest: reaching middle of T5. Coxae color brightness: same color as femora.

T1 horn: absent. Number of longitudinal carinae of T1 midlobe: obscured by other raised sculpture. T1 lateral carina: protruding laterally, visible from ventral view. T2 sculpture: with longitudinal striae or rugae, setiferous puncta present between them. T2 sublateral longitudinal foveae: absent. T3 metasomal flanges: absent. T4 sculpture: longitudinally striate to rugose, setal pits spanning interspaces. T4 metasomal flanges: absent. T5 sculpture: longitudinally striate to rugose, setal pits spanning interspaces. T5 metasomal flanges: present as slightly protruding sharp corners. T6: broader than long. Major sculpture of T6: longitudinally striate; umbilicate foveate. Microsculpture of T6: absent. T6 medially: strongly convex, tapering and sloping down to a rounded apex, not separated from apical rim. T6 metasomal flanges: absent. T6 raised peripheral rim: absent. S4 sculpture: longitudinally striate or rugose, setal pits spanning interspaces. S5 sculpture: longitudinally striate to rugose, setal pits spanning interspaces. S5 median carina: present. S6 peripheral carina: absent. S6 apex in relation to T6: not exposed to dorsal view. S6 apex: rounded or acuminate.

*Male*. Body length 3.1–4 mm (n=15). A3: longer than pedicel. A5 tyloid shape: narrow, linear. A6: broader than long. A11: longer than broad; broader than long; as long as broad. Major sculpture of mesoscutal midlobe anteriorly: umbilicate foveate. Major sculpture of mesoscutal midlobe posteriorly: umbilicate foveate. Microsculpture of mesoscutal midlobe anteriorly: absent. Microsculpture of mesoscutal midlobe posteriorly: absent. Major sculpture of mesoscutellum centrally: umbilicate foveate. Major sculpture of mesoscutellum peripherally: umbilicate foveate. Microsculpture of mesoscutellum centrally: absent. Microsculpture of mesoscutellum peripherally: absent. Fore wing apex at rest: reaching apex of T5; exceeding metasomal apex; reaching middle of T6. T1 midlobe longitudinal carinae: 4. T3 metasomal flanges: absent. T4 metasomal flanges: absent. T5 metasomal flanges: absent. T6 metasomal flanges: absent. T7: weakly emarginate; truncate.

##### Diagnosis.

Both sexes: Frontal depression deep and broad but not parallel-sided, interrupted transverse carinae present dorsally; submedian carina sharp and strong. Hyperoccipital carina indicated by rugae. Occipital carina complete, convex medially. Metascutellum deeply concave, not or hardly emarginate apically, projecting dorsally. Postmarginal vein absent. Coxa not darker than rest of leg. T1 lateral carina expanded laterally. Metasomal flanges absent or with only very tiny sharp posterior corners. Female: A3 longer than pedicel. A4, A5 much broader than long. Mesoscutellum without granulate sculpture. T1 midlobe with 4 longitudinal carinae or with these obscured by raised sculpture. T6 without metasomal flanges, main body of tergum raised and vaguely separated from apical rim. Fore wing long enough to reach middle of T4 or base of T5. Male: Flagellomeres beyond A3 broader than long or only slightly longer than broad. T1 midlobe with 4 longitudinal carinae. Fore wing long enough to reach to or beyond T7. T7 truncate or very weakly emarginate.

##### Link to distribution map.

[http://hol.osu.edu/map-full.html?id=5029]

##### Material examined.

Holotype, male, S. montana: AUSTRALIA: QLD, nr. Cairns, slopes of Walshs Pyramid, forest, Gordonvale (Nelson), 2500ft, 2.VI.1913, sweeping, A. P. Dodd, SAMA DB 32-001586 (deposited in SAMA). Other material: AUSTRALIA: 112 females, 16 males, ANIC DB 32-020080, 32-020121, 32-020122, 32-020123, 32-020149, 32-020152, OSUC 359702, OSUC 359703, OSUC 429983, OSUC 429985, OSUC 429986, OSUC 441262, OSUC 441263, OSUC 441264, OSUC 441265, OSUC 441266, OSUC 441267, OSUC 441268, OSUC 441269, OSUC 441270, OSUC 441272, OSUC 441274, OSUC 441275, OSUC 441276, OSUC 441277, OSUC 441278, OSUC 441279, OSUC 441280, OSUC 441281, OSUC 441282, OSUC 441283, OSUC 441284, OSUC 441285, OSUC 441286, OSUC 441287, OSUC 441288, OSUC 441290, OSUC 441291, OSUC 441292, OSUC 441313, OSUC 441314, OSUC 441315, OSUC 441316, OSUC 441317, OSUC 441318, OSUC 441319, OSUC 441320, OSUC 441321, OSUC 441322, OSUC 441323, OSUC 441324, OSUC 441325, OSUC 441326, OSUC 441327, OSUC 441328, OSUC 441329, OSUC 441330, OSUC 441333, OSUC 441334, OSUC 441335, OSUC 441336, OSUC 441337, OSUC 441338, OSUC 441341, OSUC 441342, OSUC 441343, OSUC 441354, OSUC 441357, OSUC 441358, OSUC 441359, OSUC 441360, OSUC 441361, OSUC 441362, OSUC 441363, OSUC 441364 (ANIC); OSUC 445342 (BMNH); OSUC 441339-441340, 441349-441350 (MVMA); OSUC 148364, 429987-429988, 441271, 441351, QM Reg. No. T35148, QM Reg. No. T35150, QM Reg. No. T35152 (QMBA); OSUC 429984, 441353, 441355, 445341, 445343 (UQIC); OSUC 268189, 268191-268193, 268195, 268200-268201, 268203, 268207, 268214-268215 (USNM); OSUC 359696-359701, 441261, 441273, 441289, 441331-441332, 441344-441348, 441352, 441356, 445338-445339, 445344-445345, 453935-453936 (WINC).

##### Comments.

Dodd (1913) described *Oxyscelio montanus* from a single male specimen, of which the face is buried in glue. Identification of males in the *atricoxa*-group is problematic, but female specimens have been assigned to this species through reasoning that they likely had relatively short A4 and A5, and a strong submedian carina. Our concept of *Oxyscelio montanus* includes variation in radicle color, but intermediates between the two states (having a partially dark radicle) imply that this is a safe conclusion.

#### Oxyscelio mystacis

Burks sp. n.

http://zoobank.org/C401D67D-2BA4-4F5C-8601-16226811B725

urn:lsid:biosci.ohio-state.edu:osuc_concepts:307090

http://species-id.net/wiki/Oxyscelio_mystacis

**Figures 199–202.**
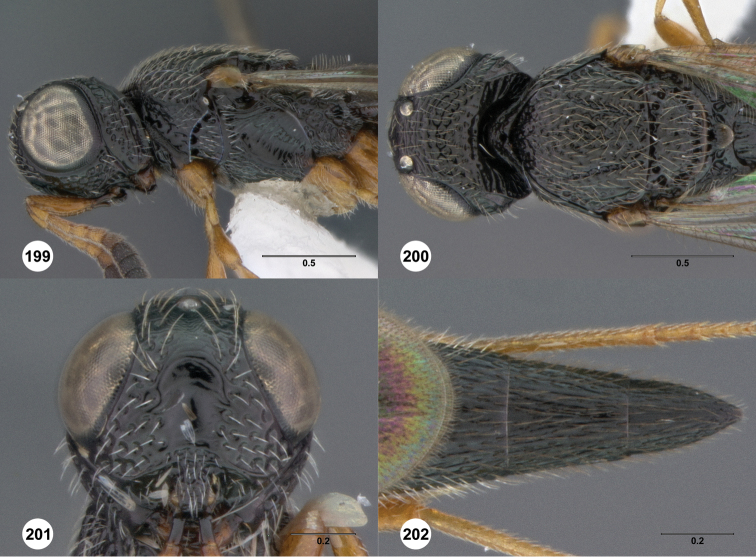
*Oxyscelio mystacis* sp. n., paratype female (OSUC 227614) **199** Head and mesosoma, lateral view **200** Head and mesosoma, dorsal view. Holotype female (OSUC 368224) **201** Head, anterior view **202** Metasoma, dorsal view. Morphbank^66^

Figures 199–202 ; Morphbank^66^

##### Description.

Female. Body length 4.35–4.6 mm (n=3).

Radicle color and shade: darker than scape. Pedicel color: same as scape. A3: longer than pedicel. A4: longer than broad. A5: longer than broad.

Ventral clypeal margin: with slightly convex median lobe. Interantennal process: not elongate. Lower frons at dorsal margin of interantennal process: with oblique carina extending towards mouth corner. Transverse curved rugae extending from frontal depression to eye: absent. Median longitudinal carina in frontal depression: absent. Ventral portion of frontal depression: with medially interrupted transverse carinae. Dorsal portion of frontal depression: without transverse carinae. Submedian carina: present only as a weak shift in elevation. Frontal depression dorsally: not hood-like, open dorsally. Upper frons major sculpture: umbilicate foveate; irregularly rugose. Upper frons microsculpture: granulate. Hyperoccipital carina: indicated by a set of irregular elevations. Carina connecting occipital carina to hyperoccipital carina: absent. Occipital carina: present laterally, absent medially. Occiput sculpture: umbilicate foveate. Extra carina ventral to occipital carina: absent. Gena length: shorter than eye. Major sculpture of gena anteroventrally: umbilicate foveate. Major sculpture of gena posteroventrally: umbilicate foveate. Microsculpture of gena anteroventrally: absent. Microsculpture of gena posteroventrally: absent.

Lateral pronotal area sculpture: mostly granulate, ventral corner with irregular carinae. Posterior border of central pronotal area: directed anteriorly, protruding at corner of epomial carina and transverse pronotal carina. Mesoscutum anteriorly: not steep, forming less than a right angle. Major sculpture of mesoscutal midlobe anteriorly: umbilicate foveate. Mesoscutal midlobe sculpture at midlength: not different from nearby sculpture. Major sculpture of mesoscutal midlobe posteriorly: umbilicate foveate. Microsculpture of mesoscutal midlobe anteriorly: granulate. Microsculpture of mesoscutal midlobe posteriorly: absent. Median mesoscutal carina: present as a flattened or rounded elevation. Major sculpture of mesoscutellum centrally: umbilicate foveate. Major sculpture of mesoscutellum peripherally: umbilicate foveate. Microsculpture of mesoscutellum centrally: absent. Microsculpture of mesoscutellum peripherally: absent. Mesoscutellar rim: not expanded. Mesoscutellar rim medially: without notch. Mesofemoral depression: longitudinally striate dorsally and ventrally. Metascutellum shape: not emarginate, convex dorsally. Metascutellar setae: absent. Metascutellum sculpture: with a median carina, otherwise weakly sculptured. Postmarginal vein: present. Fore wing apex at rest: not reaching base of T5. Coxae color brightness: same color as femora. Spines along tibiae: absent. Lateral propodeal carinae: broadly separated, not parallel anteriorly. Setae in metasomal depression: absent. Anterior sculpture of metasomal depression: absent. Median propodeal carina: absent.

T1 horn: present. Number of longitudinal carinae of T1 midlobe: obscured by other raised sculpture. T1 lateral carina: straight. T2 sculpture: with longitudinal striae or rugae, setiferous puncta present between them. T2 sublateral longitudinal foveae: absent. T3 metasomal flanges: absent. T4 sculpture: longitudinally striate to rugose, setal pits spanning interspaces. T4 metasomal flanges: absent. T5 sculpture: longitudinally striate to rugose, setal pits spanning interspaces. T5 metasomal flanges: absent. T6: longer than broad. Major sculpture of T6: umbilicate punctate; longitudinally striate. Microsculpture of T6: granulate. T6 medially: flat and tapering to a rounded apex, not separated from apical rim. T6 metasomal flanges: absent. T6 raised peripheral rim: absent. S4 sculpture: longitudinally striate or rugose, setal pits spanning interspaces. S5 sculpture: longitudinally striate to rugose, setal pits spanning interspaces. S5 median carina: absent. S6 peripheral carina: absent. S6 apex in relation to T6: not exposed to dorsal view. S6 apex: rounded or acuminate.

*Male*.

##### Diagnosis.

Both sexes: Frontal depression nearly flat, transverse carinae absent; submedian carina indicated by a few weak rugae. Oblique carina extending from bottom of frontal depression towards lower margin of eye. Head directed downward. Hyperoccipital carina indicated by rugae. Occipital carina absent medially, with protruding lateral corners. Metascutellum narrow, slightly convex. Postmarginal vein present. Coxa not darker than rest of leg. T1 lateral carina not expanded laterally. Metasomal flanges absent. Female: A3 longer than pedicel. A4, A5 about as long as broad. Mesoscutellum without granulate sculpture. T1 midlobe carinae obscured by raised smooth area. Fore wing long enough to reach middle of T4. T6 longer than broad. *Oxyscelio mystacis* is very similar to the Philippine species *Oxyscelio cupularis*, and very different from other known mainland Australian species. It differs from *Oxyscelio cupularis* in having a convex metascutellum and less granulate sculpture.

##### Etymology.

New Latin noun, genitive case, meaning “moustache.”

##### Link to distribution map.

[http://hol.osu.edu/map-full.html?id=307090]

##### Material examined.

Holotype, female: AUSTRALIA: QLD, light trap 18, 15km WNW South Johnstone, 1986, light trap, Fay & Halfpapp, OSUC 368224 (deposited in QMBA). Paratypes: AUSTRALIA: 2 females, OSUC 227614, 227623 (CNCI).

#### Oxyscelio nasi

Burks sp. n.

http://zoobank.org/C24E6923-18BD-4321-A253-531323A4FB91

urn:lsid:biosci.ohio-state.edu:osuc_concepts:275556

http://species-id.net/wiki/Oxyscelio_nasi

**Figures 203–206.**
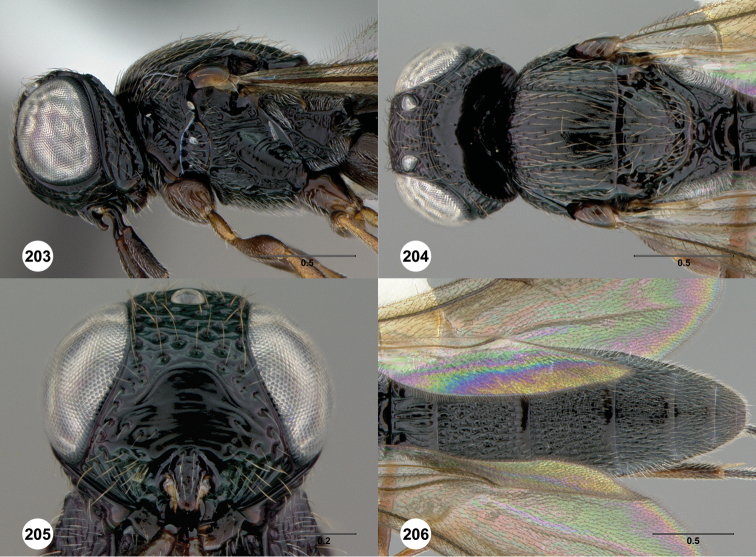
*Oxyscelio nasi* sp. n., holotype male (OSUC 368915) **203** Head and mesosoma, lateral view **204** Head and mesosoma, dorsal view **205** Head, anterior view **206** Metasoma, dorsal view. Morphbank^67^

Figures 203–206 ; Morphbank^67^

##### Description.

Female. unknown.

*Male*. Body length 3.45–4.05 mm (n=2).

Radicle color and shade: same as scape, both dark brown. Pedicel color: same as scape. A3: longer than pedicel. A5 tyloid shape: narrow, linear. A6: longer than broad. A11: longer than broad.

Ventral clypeal margin: with slightly convex median lobe. Interantennal process: elongate. Lower frons at dorsal margin of interantennal process: without transverse carina. Transverse curved rugae extending from frontal depression to eye: absent. Median longitudinal carina in frontal depression: absent. Ventral portion of frontal depression: with transverse carinae. Dorsal portion of frontal depression: without transverse carinae. Submedian carina: present only as a weak shift in elevation. Frontal depression dorsally: not hood-like, open dorsally. Upper frons major sculpture: umbilicate foveate; irregularly rugose. Upper frons microsculpture: absent. Hyperoccipital carina: indicated by a set of irregular elevations. Carina connecting occipital carina to hyperoccipital carina: absent. Occipital carina: present laterally, absent medially. Occiput sculpture: smooth. Extra carina ventral to occipital carina: absent. Gena length: shorter than eye. Major sculpture of gena anteroventrally: umbilicate foveate. Major sculpture of gena posteroventrally: umbilicate foveate; rugose. Microsculpture of gena anteroventrally: absent. Microsculpture of gena posteroventrally: absent.

Lateral pronotal area sculpture: anteriorly smooth, posterodorsal corner with dense microsculpture, ventral corner with irregular carinae. Posterior border of central pronotal area: directed posteriorly, epomial carina absent or meeting transverse pronotal carina at arch on lateral surface of pronotum. Mesoscutum anteriorly: not steep, forming less than a right angle. Major sculpture of mesoscutal midlobe anteriorly: umbilicate punctate. Major sculpture of mesoscutal midlobe posteriorly: umbilicate punctate. Microsculpture of mesoscutal midlobe anteriorly: granulate. Microsculpture of mesoscutal midlobe posteriorly: granulate. Median mesoscutal carina: present as a flattened or rounded elevation. Major sculpture of mesoscutellum centrally: umbilicate foveate. Major sculpture of mesoscutellum peripherally: umbilicate foveate. Microsculpture of mesoscutellum centrally: granulate. Microsculpture of mesoscutellum peripherally: granulate. Mesoscutellar rim: not expanded. Mesoscutellar rim medially: without notch. Mesofemoral depression: longitudinally striate dorsally, smooth ventrally. Metascutellum shape: slightly emarginate posteriorly, concave but elevated posteriorly. Metascutellar setae: absent. Metascutellum sculpture: with large smooth posterior fovea. Postmarginal vein: present. Fore wing apex at rest: exceeding metasomal apex. Coxae color brightness: same color as femora. Spines along tibiae: absent. Lateral propodeal carinae: broadly separated, not parallel anteriorly. Setae in metasomal depression: absent. Anterior sculpture of metasomal depression: with median areole or pair of pits. Median propodeal carina: absent.

T1 midlobe longitudinal carinae: 4. T3 metasomal flanges: absent. T4 metasomal flanges: absent. T5 metasomal flanges: absent. T6 metasomal flanges: absent. T7: broadly and deeply emarginate, with rounded posterolateral margins; weakly emarginate.

##### Diagnosis.

Male: Interantennal process elongate, extending far beyond antennal insertions. Frontal depression low and flat, with some rounded transverse carinae. Hyperoccipital carina indicated by a strong ruga; occipital carina incomplete medially; occiput almost entirely smooth. Mesoscutum and mesoscutellum granulate, without strong sculpture.

##### Etymology.

Latin noun, genitive case, meaning “nose.”

##### Link to distribution map.

[http://hol.osu.edu/map-full.html?id=275556]

##### Material examined.

Holotype, male: PAPUA NEW GUINEA: West New Britain Prov., New Britain Isl., primary forest edge, Cape Hoskins, 21.VI-27.VI.1973, malaise trap, Stibick, OSUC 368915 (deposited in CNCI). Paratype: PAPUA NEW GUINEA: 1 male, OSUC 369128 (CNCI).

##### Comments.

*Oxyscelio nasi* does not greatly resemble any other species of *Oxyscelio*. The elongate interantennal process is very distinctive, as is the weak mesoscutal and mesoscutellar sculpture. The two male specimens differ in the depth of the apical T7 emargination, but this is regarded here as intraspecific variation.

#### Oxyscelio nigriclava

(Dodd)

http://zoobank.org/DA87D466-FD60-493C-892F-266B3F30C935

urn:lsid:biosci.ohio-state.edu:osuc_concepts:302871

http://species-id.net/wiki/Oxyscelio_nigriclava

**Figures 207–212.**
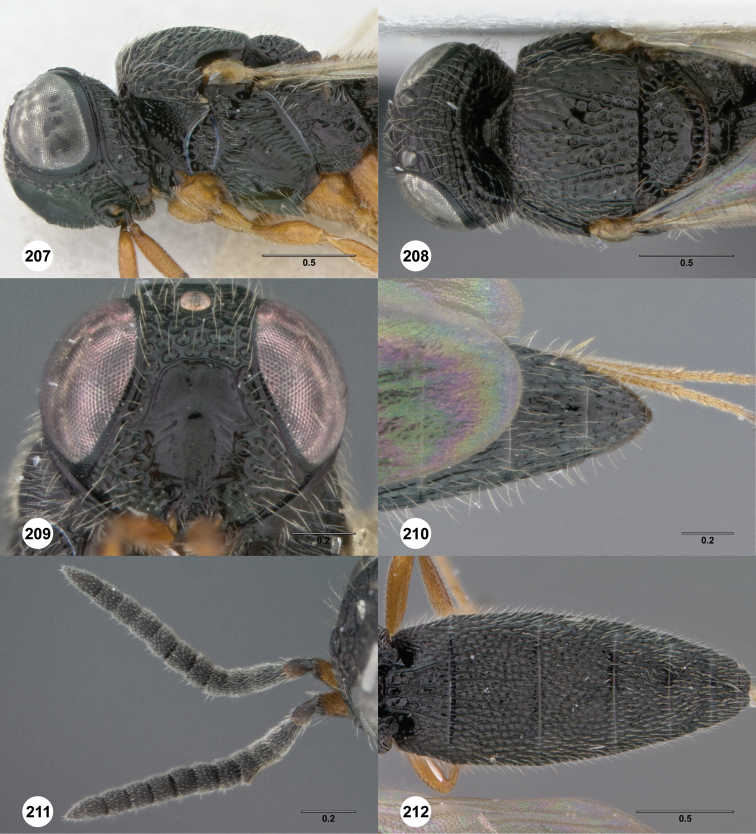
*Oxyscelio nigriclava* (Dodd), female (OSUC 441383) **207** Head and mesosoma, lateral view **208** Head and mesosoma, dorsal view. Female (OSUC 441384) **209** Head, anterior view. Female (OSUC 148370) **210** Metasoma, dorsal view. Male (OSUC 441386) **211** Antenna **212** Metasoma, dorsal view. Morphbank^68^

Figures 207–212 ; Morphbank^68^

Sceliomorpha nigriclava Dodd, 1914: 104 (original description); Kieffer 1926: 303, 307 (description, keyed); Naumann et al. 1994: 71 (holotype transferred to ANIC).
Oxyscelio nigriclavus (Dodd): Dodd 1931: 76 (generic transfer); Galloway 1976: 99 (type information).

##### Description.

Female. Body length 3.95–4.3 mm (n=6).

Radicle color and shade: darker than scape. Pedicel color: same as scape. A3: longer than pedicel. A4: broader than long. A5: broader than long.

Ventral clypeal margin: with slightly convex median lobe. Interantennal process: not elongate. Lower frons at dorsal margin of interantennal process: without transverse carina. Transverse curved rugae extending from frontal depression to eye: absent. Median longitudinal carina in frontal depression: absent. Ventral portion of frontal depression: with medially interrupted transverse foveae. Dorsal portion of frontal depression: with medially interrupted transverse carinae. Submedian carina: present. Frontal depression dorsally: not hood-like, open dorsally. Upper frons major sculpture: umbilicate foveate; irregularly rugose. Upper frons microsculpture: granulate. Hyperoccipital carina: indicated by a set of irregular elevations. Carina connecting occipital carina to hyperoccipital carina: absent. Occipital carina: weakly arched dorsally, with rounded lateral corners. Occiput sculpture: irregularly sculptured. Extra carina ventral to occipital carina: present, complete. Gena length: shorter than eye. Major sculpture of gena anteroventrally: umbilicate foveate. Major sculpture of gena posteroventrally: umbilicate foveate. Microsculpture of gena anteroventrally: absent. Microsculpture of gena posteroventrally: granulate.

Lateral pronotal area sculpture: anteriorly smooth, posterodorsal corner with dense microsculpture, ventral corner with irregular carinae. Posterior border of central pronotal area: directed anteriorly, protruding at corner of epomial carina and transverse pronotal carina. Mesoscutum anteriorly: very steep and tall, descending at a right angle or protruding anteriorly. Major sculpture of mesoscutal midlobe anteriorly: umbilicate foveate. Mesoscutal midlobe sculpture at midlength: not different from nearby sculpture. Major sculpture of mesoscutal midlobe posteriorly: umbilicate foveate. Microsculpture of mesoscutal midlobe anteriorly: granulate. Microsculpture of mesoscutal midlobe posteriorly: absent. Median mesoscutal carina: present as a flattened or rounded elevation. Major sculpture of mesoscutellum centrally: umbilicate foveate. Major sculpture of mesoscutellum peripherally: umbilicate foveate. Microsculpture of mesoscutellum centrally: punctate. Microsculpture of mesoscutellum peripherally: absent. Mesoscutellar rim: not expanded. Mesoscutellar rim medially: without notch. Mesofemoral depression: longitudinally striate dorsally, smooth ventrally. Metascutellum shape: slightly emarginate posteriorly, concave but elevated posteriorly. Metascutellar setae: absent. Metascutellum sculpture: with large smooth posterior fovea. Spines along tibiae: absent. Lateral propodeal carinae: broadly separated, not parallel anteriorly. Setae in metasomal depression: absent. Anterior sculpture of metasomal depression: absent. Median propodeal carina: absent. Postmarginal vein: present. Fore wing apex at rest: not reaching base of T5. Coxae color brightness: same color as femora.

T1 horn: absent. Number of longitudinal carinae of T1 midlobe: obscured by other raised sculpture. T1 lateral carina: protruding laterally, visible from ventral view. T2 sculpture: with longitudinal striae or rugae, setiferous puncta present between them. T2 sublateral longitudinal foveae: absent. T3 metasomal flanges: absent. T4 sculpture: longitudinally striate to rugose, setal pits spanning interspaces. T4 metasomal flanges: absent. T5 sculpture: longitudinally striate to rugose, setal pits spanning interspaces. T5 metasomal flanges: present as slightly protruding sharp corners. T6: broader than long. Major sculpture of T6: longitudinally striate; umbilicate foveate. Microsculpture of T6: absent. T6 medially: flat and tapering to a rounded apex, not separated from apical rim. T6 metasomal flanges: absent. T6 raised peripheral rim: present. S4 sculpture: longitudinally striate or rugose, setal pits spanning interspaces. S5 sculpture: longitudinally striate to rugose, setal pits spanning interspaces. S5 median carina: present. S6 peripheral carina: absent. S6 apex in relation to T6: not exposed to dorsal view. S6 apex: rounded or acuminate.

*Male*. Body length 3.4–3.7 mm (n=2). A3: longer than pedicel. A5 tyloid shape: narrow, linear. A6: broader than long. A11: longer than broad. Major sculpture of mesoscutal midlobe anteriorly: umbilicate foveate. Major sculpture of mesoscutal midlobe posteriorly: umbilicate foveate. Microsculpture of mesoscutal midlobe anteriorly: granulate. Microsculpture of mesoscutal midlobe posteriorly: absent. Major sculpture of mesoscutellum centrally: umbilicate foveate. Major sculpture of mesoscutellum peripherally: umbilicate foveate. Microsculpture of mesoscutellum centrally: absent. Microsculpture of mesoscutellum peripherally: absent. Fore wing apex at rest: reaching middle of T6. T1 midlobe longitudinal carinae: 4. T3 metasomal flanges: absent. T4 metasomal flanges: absent. T5 metasomal flanges: absent. T6 metasomal flanges: present as sharp corners that do not protrude. T7: truncate.

##### Diagnosis.

Both sexes: Frontal depression deep and broad, without transverse carinae; submedian carina sharp but irregular. Hyperoccipital carina indicated by weak rugae. Occipital carina complete, weakly convex or sinuate medially. Metascutellum deeply concave, truncate apically, projecting dorsally. Postmarginal vein absent. Coxa same color as rest of leg. T1 lateral carina expanded laterally. Female: A3 longer than pedicel. A4, A5 broader than long. Mesoscutellum without granulate sculpture. T1 midlobe carinae obscured by raised sculpture. T5 with tiny sharp posterior metasomal flanges, T6 with a narrow peripheral carina, main body of T6 raised and abruptly separated from apical rim. Fore wing long enough to reach middle of T4 or base of T5. Male: All flagellomeres past A3 as broad or broader than long. T1 midlobe with 4 longitudinal carinae. Fore wing long enough to reach T7. T6 with tiny sharp metasomal flanges. T7 truncate, arched.

##### Link to distribution map.

[http://hol.osu.edu/map-full.html?id=302871]

##### Material examined.

Holotype, female, S. nigriclava: AUSTRALIA: NSW, no date, ANIC DB 32-012569 (deposited in ANIC). Other material: AUSTRALIA: 7 females, 2 males, OSUC 441379-441382, 441385-441386 (ANIC); OSUC 148370, 441384 (QMBA); OSUC 441383 (WINC).

##### Comments.

*Oxyscelio nigriclava* is a problematic species that may be near *Oxyscelio mirellus* and other species with strong Metasomal flanges, but which has a very differently shaped T6 in females. The main body of T6 is distinctly raised above its apical rim, but is not concave apically and has a semi-translucent rim instead of truncated metasomal flanges.

#### Oxyscelio nigricoxa

(Dodd)

http://zoobank.org/B769D0CF-1DF0-4AB5-A6F1-2EEBF39B874A

urn:lsid:biosci.ohio-state.edu:osuc_concepts:5031

http://species-id.net/wiki/Oxyscelio_nigricoxa

**Figures 213–218.**
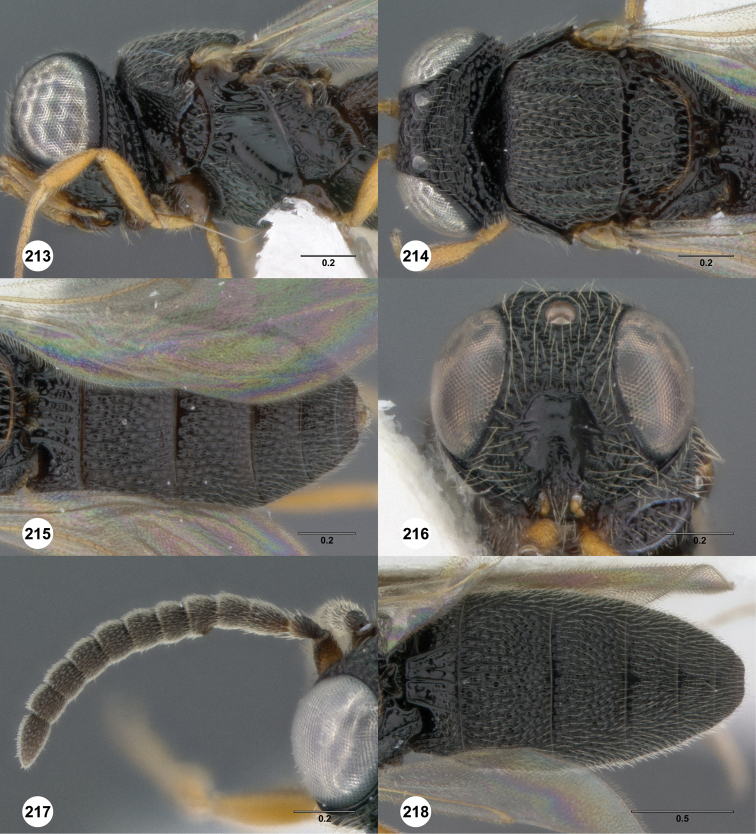
*Oxyscelio nigricoxa* (Dodd), female (OSUC 462599) **213** Head and mesosoma, lateral view **214** Head and mesosoma, dorsal view **215** Metasoma, dorsal view. Female (QDPC 0-165685) **216** Head, anterior view. Paratype male (OSUC 438140) **217** Antenna **218** Metasoma, dorsal view. Morphbank^69^

Figures 213–218 ; Morphbank^69^

Sceliomorpha nigricoxa Dodd, 1913: 165 (original description); Kieffer 1926: 302, 307 (description, keyed). *Dicroteleia nigricoxa* (Dodd): Dodd 1914: 107 (generic transfer).
Oxyscelio nigricoxa (Dodd): Dodd 1931: 76 (generic transfer); Galloway 1976: 99 (type information).

##### Description.

Female. Body length 2.35–3.45 mm (n=20).

Radicle color and shade: darker than scape. Pedicel color: same as scape; at least partially darker than scape. A3: shorter than pedicel. A4: longer than broad. A5: broader than long.

Ventral clypeal margin: with slightly convex median lobe. Interantennal process: not elongate. Lower frons at dorsal margin of interantennal process: without transverse carina. Transverse curved rugae extending from frontal depression to eye: absent. Median longitudinal carina in frontal depression: absent. Ventral portion of frontal depression: smooth. Dorsal portion of frontal depression: without transverse carinae. Submedian carina: absent. Frontal depression dorsally: not hood-like, open dorsally. Upper frons major sculpture: umbilicate foveate. Upper frons microsculpture: absent. Hyperoccipital carina: absent. Carina connecting occipital carina to hyperoccipital carina: absent. Occipital carina: uniformly rounded dorsally. Occiput sculpture: umbilicate foveate medially, becoming smooth laterally. Extra carina ventral to occipital carina: absent. Gena length: shorter than eye. Major sculpture of gena anteroventrally: umbilicate foveate. Major sculpture of gena posteroventrally: umbilicate foveate; rugose. Microsculpture of gena anteroventrally: absent. Microsculpture of gena posteroventrally: absent.

Lateral pronotal area sculpture: with shallow irregular carinae, posterodorsal corner with dense microsculpture. Posterior border of central pronotal area: directed anteriorly, protruding at corner of epomial carina and transverse pronotal carina. Mesoscutum anteriorly: very steep and tall, descending at a right angle or protruding anteriorly. Major sculpture of mesoscutal midlobe anteriorly: umbilicate foveate; umbilicate punctate. Mesoscutal midlobe sculpture at midlength: not different from nearby sculpture. Major sculpture of mesoscutal midlobe posteriorly: umbilicate foveate; longitudinally rugose. Microsculpture of mesoscutal midlobe anteriorly: absent. Microsculpture of mesoscutal midlobe posteriorly: absent. Median mesoscutal carina: present as a vague, occasionally interrupted elevation. Major sculpture of mesoscutellum centrally: absent; umbilicate foveate. Major sculpture of mesoscutellum peripherally: umbilicate foveate. Microsculpture of mesoscutellum centrally: absent; punctate. Microsculpture of mesoscutellum peripherally: absent; punctate. Mesoscutellar rim: not expanded. Mesoscutellar rim medially: without notch. Mesofemoral depression: longitudinally striate dorsally, smooth ventrally; smooth. Metascutellum shape: deeply emarginate, with the resulting pair of posterior processes subtriangular and directed dorsally. Metascutellar setae: absent. Metascutellum sculpture: with large smooth posterior fovea. Spines along tibiae: absent. Lateral propodeal carinae: broadly separated, not parallel anteriorly. Setae in metasomal depression: absent. Anterior sculpture of metasomal depression: absent. Median propodeal carina: absent. Postmarginal vein: absent. Fore wing apex at rest: exceeding metasomal apex. Coxae color brightness: darker than femora.

T1 horn: absent. Number of longitudinal carinae of T1 midlobe: 4. T1 lateral carina: protruding laterally, visible from ventral view. T2 sculpture: with longitudinal striae or rugae, setiferous puncta present between them. T2 sublateral longitudinal foveae: absent. T3 metasomal flanges: absent. T4 sculpture: longitudinally striate to rugose, setal pits spanning interspaces. T4 metasomal flanges: absent. T5 sculpture: longitudinally striate to rugose, setal pits spanning interspaces. T5 metasomal flanges: absent. T6: broader than long. Major sculpture of T6: umbilicate punctate. Microsculpture of T6: absent. T6 medially: slightly emarginate, not separated from apical rim. T6 metasomal flanges: absent. T6 raised peripheral rim: absent. S4 sculpture: densely setose, setal pits between very weak longitudinal rugae. S5 sculpture: densely setose, setal pits between very weak longitudinal rugae. S5 median carina: absent. S6 peripheral carina: absent. S6 apex in relation to T6: not exposed to dorsal view. S6 apex: rounded or acuminate.

*Male*. Body length 2.15–3.4 mm (n=20). A3: longer than pedicel. A5 tyloid shape: narrow, linear. A6: broader than long. A11: broader than long; as long as broad. Major sculpture of mesoscutal midlobe anteriorly: umbilicate foveate. Major sculpture of mesoscutal midlobe posteriorly: umbilicate foveate. Microsculpture of mesoscutal midlobe anteriorly: granulate. Microsculpture of mesoscutal midlobe posteriorly: absent. Major sculpture of mesoscutellum centrally: umbilicate foveate. Major sculpture of mesoscutellum peripherally: umbilicate foveate. Microsculpture of mesoscutellum centrally: absent. Microsculpture of mesoscutellum peripherally: absent. Fore wing apex at rest: exceeding metasomal apex. T1 midlobe longitudinal carinae: 4. T3 metasomal flanges: absent. T4 metasomal flanges: absent. T5 metasomal flanges: absent. T6 metasomal flanges: absent. T7: weakly emarginate.

##### Diagnosis.

Both sexes: Frontal depression shallow, transverse carinae absent or broadly interrupted ventrally, no carinae present above dorsal separator; submedian carina absent medially. Hyperoccipital carina indicated by weak rugae. Occipital carina complete, weakly convex medially. Metascutellum deeply concave, emarginate apically, projecting dorsally. Postmarginal vein absent. Coxa darker than rest of leg. T1 lateral carina expanded laterally. Metasomal flanges absent. Female: A3 longer than pedicel. A4 about as long as broad, A5 broader than long. Mesoscutellum without granulate sculpture. T1 midlobe with 4 longitudinal carinae. T6 without metasomal flanges, emarginate apically. Fore wing long enough to exceed metasomal apex. Male: A4 broader than long, A11 longer than broad. T1 midlobe with 4 longitudinal carinae. Fore wing long enough to reach beyond T7. T7 emarginate apically. The smaller-bodied *Oxyscelio exiguitatis* is similar to *Oxyscelio nigricoxa*, but has a deep frontal depression and even deeper T6 emargination.

##### Link to distribution map.

[http://hol.osu.edu/map-full.html?id=5031]

##### Associations.

On sticky seed of *Pisonia brunoniana* Endl.: [Caryophyllales: Nyctaginaceae]

##### Material examined.

Holotype, male, S. nigricoxa: AUSTRALIA: QLD, summit of mountain range, grass / forest, Gordonvale (Nelson), 1500ft, 30.V.1913, sweeping, A. A. Girault, SAMA DB 32-001587 (deposited in SAMA). Other material: AUSTRALIA: 62 females, 152 males, ANIC DB 32-020124, 32-020125, 32-020144, OSUC 359709, OSUC 359710, OSUC 359711, OSUC 438058, OSUC 438059, OSUC 438060, OSUC 438061, OSUC 438062, OSUC 438063, OSUC 438064, OSUC 438067, OSUC 438069, OSUC 438072, OSUC 438093, OSUC 438094, OSUC 438095, OSUC 438096, OSUC 438097, OSUC 438098, OSUC 438099, OSUC 438100, OSUC 438102, OSUC 438103, OSUC 438110, OSUC 438111, OSUC 438112, OSUC 438113, OSUC 438114, OSUC 438115, OSUC 438116, OSUC 438117, OSUC 438118, OSUC 438119, OSUC 438120, OSUC 438121, OSUC 438122, OSUC 438123, OSUC 438133, OSUC 438134, OSUC 438135, OSUC 438136, OSUC 438137, OSUC 438138, OSUC 438139, OSUC 438140, OSUC 438141, OSUC 438142, OSUC 438143, OSUC 438152 (ANIC); OSUC 449022, 451335, 451359 (BMNH); OSUC 462598-462601 (CNCI); OSUC 438068, 438104, 438108, 438182, QDPC 0-165634, QDPC 0-165641, QDPC 0-165645, QDPC 0-165651, QDPC 0-165662, QDPC 0-165668, QDPC 0-165670, QDPC 0-165680, QDPC 0-165681, QDPC 0-165683, QDPC 0-165685, QDPC 0-165689, QDPC 0-165697, QDPC 0-165700, QDPC 0-165710, QDPC 0-165712, QDPC 0-165716, QDPC 0-165717, QDPC 0-165722, QDPC 0-165733, QDPC 0-165742, QDPC 0-165743, QDPC 0-165744, QDPC 0-165748, QDPC 0-165755, QDPC 0-165763, QDPC 0-165764, QDPC 0-165766, QDPC 0-165767, QDPC 0-165783 (QDPC); OSUC 438073, 438092, 438101 (QMBA); OSUC 448607 (QPIM); OSUC 438070, 449028, 451329-451334, 451339-451340 (UQIC); OSUC 359704-359708, 359712, 436936, 438039-438057, 438065-438066, 438071, 438074-438091, 438105-438107, 438109, 438124-438132, 438144-438147, 438149-438151, 438153-438181, 438183-438186, 449029, 451336-451338, 451357-451358, 451360 (WINC).

#### Oxyscelio nitoris

Burks sp. n.

http://zoobank.org/BAD32776-3ECD-4EB9-9139-1C5C902909B7

urn:lsid:biosci.ohio-state.edu:osuc_concepts:307091

http://species-id.net/wiki/Oxyscelio_nitoris

**Figures 219–224.**
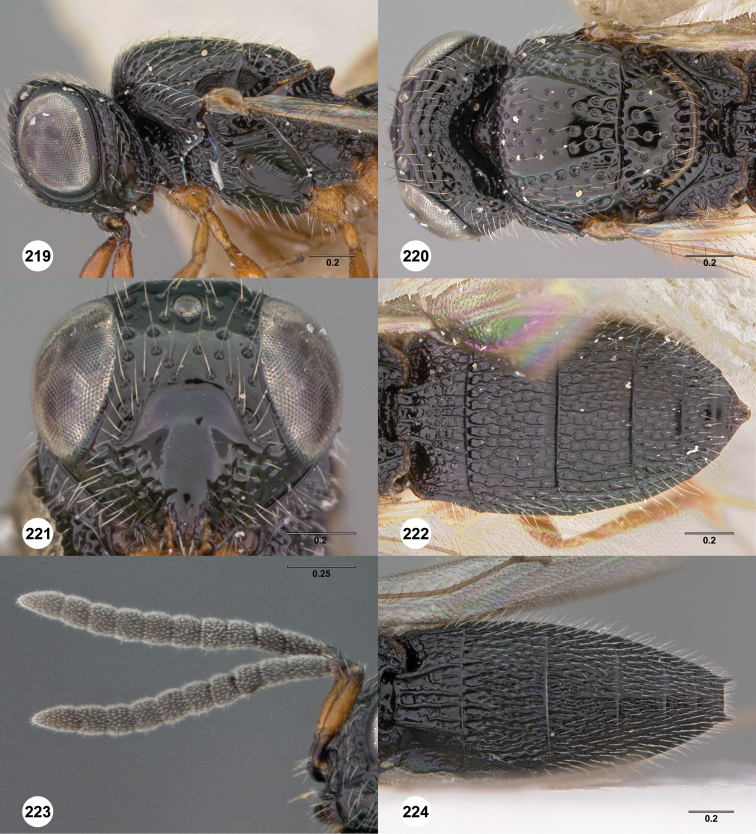
*Oxyscelio nitoris* sp. n., female (OSUC 359541) **219** Head and mesosoma, lateral view **220** Head and mesosoma, dorsal view **221** Head, anterior view **222** Metasoma, dorsal view. Paratype male (QM Reg. No. T35136) **223** Antenna **224** Metasoma, dorsal view. Morphbank^70^

Figures 219–224 ; Morphbank^70^

##### Description.

Female. Body length 2.9–3.55 mm (n=20).

Radicle color and shade: darker than scape. Pedicel color: at least partially darker than scape. A3: longer than pedicel. A4: broader than long. A5: broader than long.

Ventral clypeal margin: with slightly convex median lobe. Interantennal process: not elongate. Lower frons at dorsal margin of interantennal process: without transverse carina. Transverse curved rugae extending from frontal depression to eye: absent. Median longitudinal carina in frontal depression: absent. Ventral portion of frontal depression: smooth. Dorsal portion of frontal depression: without transverse carinae. Submedian carina: present only as a weak shift in elevation. Frontal depression dorsally: not hood-like, open dorsally. Upper frons major sculpture: umbilicate foveate. Upper frons microsculpture: absent. Hyperoccipital carina: indicated by a set of irregular elevations. Carina connecting occipital carina to hyperoccipital carina: absent. Occipital carina: omicron-shaped, with sharp corners where median portion meets lateral portions. Occiput sculpture: transversely rugose. Extra carina ventral to occipital carina: absent. Gena length: shorter than eye. Major sculpture of gena anteroventrally: umbilicate foveate. Major sculpture of gena posteroventrally: umbilicate foveate; absent. Microsculpture of gena anteroventrally: absent. Microsculpture of gena posteroventrally: absent.

Lateral pronotal area sculpture: anteriorly smooth, posterodorsal corner with dense microsculpture, ventral corner with irregular carinae. Posterior border of central pronotal area: directed posteriorly, epomial carina absent or meeting transverse pronotal carina at arch on lateral surface of pronotum. Mesoscutum anteriorly: very steep and tall, descending at a right angle or protruding anteriorly. Major sculpture of mesoscutal midlobe anteriorly: umbilicate foveate. Mesoscutal midlobe sculpture at midlength: not different from nearby sculpture. Major sculpture of mesoscutal midlobe posteriorly: umbilicate foveate. Microsculpture of mesoscutal midlobe anteriorly: absent. Microsculpture of mesoscutal midlobe posteriorly: absent. Median mesoscutal carina: present as a vague, occasionally interrupted elevation. Major sculpture of mesoscutellum centrally: absent; umbilicate foveate. Major sculpture of mesoscutellum peripherally: umbilicate foveate. Microsculpture of mesoscutellum centrally: absent. Microsculpture of mesoscutellum peripherally: absent. Mesoscutellar rim: not expanded. Mesoscutellar rim medially: without notch. Mesofemoral depression: longitudinally striate dorsally, smooth ventrally. Metascutellum shape: deeply emarginate, with the resulting pair of posterior processes subtriangular and directed dorsally. Metascutellar setae: absent. Metascutellum sculpture: with large smooth posterior fovea. Postmarginal vein: absent. Fore wing apex at rest: exceeding metasomal apex. Coxae color brightness: same color as femora. Spines along tibiae: absent. Lateral propodeal carinae: broadly separated, but parallel for a short distance anteriorly. Setae in metasomal depression: absent. Anterior sculpture of metasomal depression: with median areole or pair of pits. Median propodeal carina: present.

T1 horn: absent. Number of longitudinal carinae of T1 midlobe: 4. T1 lateral carina: protruding laterally, visible from ventral view. T2 sculpture: with longitudinal striae or rugae, setiferous puncta present between them. T2 sublateral longitudinal foveae: absent. T3 metasomal flanges: absent. T4 sculpture: longitudinally striate to rugose, setal pits spanning interspaces. T4 metasomal flanges: absent. T5 sculpture: longitudinally striate to rugose, setal pits spanning interspaces. T5 metasomal flanges: absent. T6: broader than long. Major sculpture of T6: umbilicate punctate. Microsculpture of T6: absent. T6 medially: slightly emarginate, not separated from apical rim. T6 metasomal flanges: absent. T6 raised peripheral rim: absent. S4 sculpture: longitudinally striate or rugose, setal pits spanning interspaces. S5 sculpture: longitudinally striate to rugose, setal pits spanning interspaces. S5 median carina: absent. S6 peripheral carina: absent. S6 apex in relation to T6: not exposed to dorsal view. S6 apex: rounded or acuminate.

*Male*. Body length 2.7–3.55 mm (n=20).A3: longer than pedicel. A5 tyloid shape: narrow, linear. A6: broader than long. A11: longer than broad. Major sculpture of mesoscutal midlobe anteriorly: umbilicate foveate. Major sculpture of mesoscutal midlobe posteriorly: umbilicate foveate. Microsculpture of mesoscutal midlobe anteriorly: granulate. Microsculpture of mesoscutal midlobe posteriorly: absent. Major sculpture of mesoscutellum centrally: umbilicate foveate. Major sculpture of mesoscutellum peripherally: umbilicate foveate. Microsculpture of mesoscutellum centrally: absent. Microsculpture of mesoscutellum peripherally: absent; punctate. Fore wing apex at rest: exceeding metasomal apex. T1 midlobe longitudinal carinae: 4. T3 metasomal flanges: absent. T4 metasomal flanges: absent. T5 metasomal flanges: absent. T6 metasomal flanges: absent. T7: with a pair of sharply defined spine-like posterolateral projections.

##### Diagnosis.

Both sexes: Mesoscutum and mesoscutellum black. Frontal depression shallow, transverse carinae absent or interrupted; submedian carina absent. Hyperoccipital carina indicated by rugae. Occipital carina complete, sinuate medially. Metascutellum deeply concave, emarginate apically, projecting dorsally. Coxa same color as rest of leg. T1 lateral carina expanded laterally. Female: A3 not shorter than pedicel. A4, A5 broader than long. T1 midlobe with 4 longitudinal carinae. T6 without metasomal flanges. Fore wing long enough to reach middle of T6 or beyond metasomal apex. Main body of T6 not abruptly separated from apical rim. Male: A3 longer than pedicel. A4, A11 longer than broad. T1 midlobe with 4 longitudinal carinae. Fore wing long enough to exceed metasomal apex. T7 with elongate spine-like posterior projections. *Oxyscelio nitoris* is very similar to *Oxyscelio livens* and *Oxyscelio palati*, but has a moderate-length antenna in combination with a lack of T6 metasomal flanges.

##### Etymology.

Latin noun, genitive case, meaning “brightness.”

##### Link to distribution map.

[http://hol.osu.edu/map-full.html?id=307091]

##### Material examined.

Holotype, female: AUSTRALIA: QLD, Maroochy Horticultural Research Station, Nambour, 8.III-15.III.1985, malaise trap, OSUC 359530 (deposited in QMBA). Paratypes: AUSTRALIA: 55 females, 24 males, ANIC DB 32-020089, 32-020091, 32-020092, 32-020093, 32-020101, 32-020102, 32-020143, OSUC 359540, OSUC 359541, OSUC 359543, OSUC 359544, OSUC 359545, OSUC 359547, OSUC 359548, OSUC 359552, OSUC 359553, OSUC 359555, OSUC 359558, OSUC 359559, OSUC 359561 (ANIC); OSUC 449087 (BMNH); OSUC 227550, 227632-227633 (CNCI); OSUC 359549-359550, QDPC 0-165631, QDPC 0-165677 (QDPC); OSUC 148382, 359551, 435937, QM Reg. No. T35135, QM Reg. No. T35137, QM Reg. No. T35138, QM Reg. No. T35141, QM Reg. No. T35143, QM Reg. No. T35144, QM Reg. No. T35146 (QMBA); OSUC 359527-359529, 359531-359539, 359542, 359546, 359554, 359556-359557, 359560, 359562-359574, 436937-436938, 449081-449086, 449088-449089 (WINC).

#### Oxyscelio obliquiatis

Burks sp. n.

http://zoobank.org/5193D8BF-EF12-4DDC-9CD8-0C5C28E34D62

urn:lsid:biosci.ohio-state.edu:osuc_concepts:307092

http://species-id.net/wiki/Oxyscelio_obliquiatis

**Figures 225–230.**
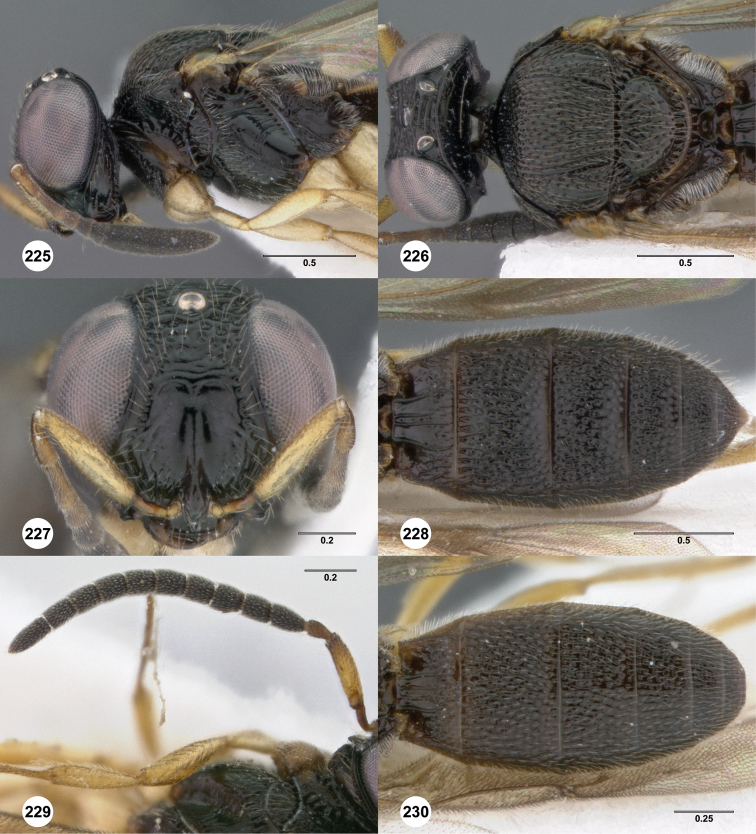
*Oxyscelio obliquiatis* sp. n., holotype female (OSUC 359747) **225** Head and mesosoma, lateral view **226** Head and mesosoma, dorsal view **227** Head, anterior view **228** Metasoma, dorsal view. Paratype male (OSUC 359780) **229** Antenna **230** Metasoma, dorsal view. Morphbank^71^

Figures 225–230 ; Morphbank^71^

##### Description.

Female. Body length 2.65–3.7 mm (n=20).

Radicle color and shade: same as scape, both yellowish or reddish. Pedicel color: same as scape. A3: longer than pedicel. A4: broader than long. A5: broader than long.

Ventral clypeal margin: with slightly convex median lobe. Interantennal process: not elongate. Lower frons at dorsal margin of interantennal process: without transverse carina. Transverse curved rugae extending from frontal depression to eye: absent. Median longitudinal carina in frontal depression: present. Ventral portion of frontal depression: smooth. Dorsal portion of frontal depression: without transverse carinae. Submedian carina: present only as a weak shift in elevation. Frontal depression dorsally: not hood-like, open dorsally. Upper frons major sculpture: umbilicate foveate. Upper frons microsculpture: absent. Hyperoccipital carina: present as a single carina. Carina connecting occipital carina to hyperoccipital carina: absent. Occipital carina: present laterally, absent medially. Occiput sculpture: smooth. Extra carina ventral to occipital carina: present, complete. Gena length: shorter than eye. Major sculpture of gena anteroventrally: rugose; umbilicate punctate; absent. Major sculpture of gena posteroventrally: umbilicate punctate; absent. Microsculpture of gena anteroventrally: absent. Microsculpture of gena posteroventrally: absent.

Lateral pronotal area sculpture: anteriorly smooth, posterodorsal corner with dense microsculpture, ventral corner with irregular carinae. Posterior border of central pronotal area: directed posteriorly, epomial carina absent or meeting transverse pronotal carina at arch on lateral surface of pronotum. Mesoscutum anteriorly: very steep and tall, descending at a right angle or protruding anteriorly. Major sculpture of mesoscutal midlobe anteriorly: umbilicate punctate. Mesoscutal midlobe sculpture at midlength: not different from nearby sculpture. Major sculpture of mesoscutal midlobe posteriorly: longitudinally rugose; umbilicate punctate. Microsculpture of mesoscutal midlobe anteriorly: absent. Microsculpture of mesoscutal midlobe posteriorly: absent. Median mesoscutal carina: absent. Major sculpture of mesoscutellum centrally: longitudinally rugose; obliquely rugose; umbilicate punctate. Major sculpture of mesoscutellum peripherally: obliquely rugose; umbilicate punctate. Microsculpture of mesoscutellum centrally: absent. Microsculpture of mesoscutellum peripherally: absent. Mesoscutellar rim: not expanded. Mesoscutellar rim medially: without notch. Mesofemoral depression: longitudinally striate dorsally, smooth ventrally. Metascutellum shape: deeply emarginate, with the resulting pair of posterior processes subtriangular and directed dorsally. Metascutellar setae: absent. Metascutellum sculpture: with large smooth posterior fovea. Postmarginal vein: absent. Fore wing apex at rest: exceeding metasomal apex. Coxae color brightness: same color as femora. Spines along tibiae: absent. Lateral propodeal carinae: broadly separated, not parallel anteriorly. Setae in metasomal depression: absent. Anterior sculpture of metasomal depression: with median areole or pair of pits. Median propodeal carina: present.

T1 horn: absent. Number of longitudinal carinae of T1 midlobe: 4. T1 lateral carina: straight; protruding laterally, visible from ventral view. T2 sculpture: with longitudinal striae or rugae, setiferous puncta present between them. T2 sublateral longitudinal foveae: absent. T3 metasomal flanges: absent. T4 sculpture: densely foveate, longitudinal sculpture irregular. T4 metasomal flanges: absent. T5 sculpture: densely foveate, longitudinal sculpture irregular. T5 metasomal flanges: absent. T6: broader than long. Major sculpture of T6: umbilicate punctate. Microsculpture of T6: absent. T6 medially: flat and tapering to a rounded apex, not separated from apical rim. T6 metasomal flanges: absent. T6 raised peripheral rim: absent. S4 sculpture: longitudinally striate or rugose, setal pits spanning interspaces. S5 sculpture: longitudinally striate to rugose, setal pits spanning interspaces. S5 median carina: absent. S6 peripheral carina: absent. S6 apex in relation to T6: not exposed to dorsal view. S6 apex: rounded or acuminate.

*Male*. Body length 2.5–3.4 mm (n=20). A3: longer than pedicel. A5 tyloid shape: narrow, linear. A6: longer than broad. A11: longer than broad. Major sculpture of mesoscutal midlobe anteriorly: umbilicate foveate. Major sculpture of mesoscutal midlobe posteriorly: umbilicate foveate; longitudinally rugose. Microsculpture of mesoscutal midlobe anteriorly: granulate. Microsculpture of mesoscutal midlobe posteriorly: absent. Major sculpture of mesoscutellum centrally: umbilicate punctate; irregularly rugose. Major sculpture of mesoscutellum peripherally: umbilicate punctate; irregularly rugose. Microsculpture of mesoscutellum centrally: absent; granulate. Microsculpture of mesoscutellum peripherally: granulate. Fore wing apex at rest: exceeding metasomal apex. T1 midlobe longitudinal carinae: 4. T3 metasomal flanges: absent. T4 metasomal flanges: absent. T5 metasomal flanges: absent. T6 metasomal flanges: absent. T7: weakly emarginate.

**Diagnosis**. Both sexes: Frontal depression shallow; submedian carina indicated by a set of weak rugae, flat or only weakly rounded dorsally. Hyperoccipital carina sharp and strong. Occipital carina incomplete, lateral portions short and not approaching hyperoccipital carina. Occiput mostly smooth, with many rugae dorsally and with a row of weak setiferous puncta. Metascutellum broad and concave, strongly emarginate apically, projecting dorsally. Postmarginal vein present. Coxa not darker than rest of leg. T1 lateral carina slightly expanded laterally, sometimes visible from ventral view. Metasomal flanges absent. Female: A3 longer than pedicel. A4 as long or longer than broad, A5 broader than long. Mesoscutellum with strong oblique rugae. T1 midlobe with 4 longitudinal carinae. Fore wing long enough to reach to or beyond metasomal apex. T6 broader than long. Male: A4, A11 longer than broad. T1 midlobe with 4 longitudinal carinae. Mesoscutellum with extensive granulate sculpture. Fore wing long enough to reach far beyond metasomal apex. T7 tiny, truncate.

##### Etymology.

Latin noun, genitive case, meaning “obliqueness.”

##### Link to distribution map.

[http://hol.osu.edu/map-full.html?id=307092]

##### Material examined.

Holotype, female: AUSTRALIA: QLD, GS1, Mount Haig, 17°06'S, 145°36'E, 1150m, 1.XII–3.I.1995, flight intercept trap, P. Zborowski, OSUC 359747 (deposited in ANIC). Paratypes: AUSTRALIA: 27 females, 27 males, OSUC 359746, 359748-359749, 359767, 359769-359774, 359779, 437003 (ANIC); OSUC 359778, 451291, QDPC 0-165649, QDPC 0-165666, QDPC 0-165667, QDPC 0-165671, QDPC 0-165776, QDPC 0-165777, QDPC 0-165785, QDPC 0-165786 (QDPC); OSUC 148358, 148373, 148375, 148384, 148473-148474, 359750-359754, 359756-359761, 359775-359776, 359780-359781 (QMBA); OSUC 359745, 359755, 359762-359766, 359768, 359777, 451289-451290 (WINC).

##### Comments.

*Oxyscelio obliquiatis* is unusual within the *flavipes*-group in having a slightly expanded lateral carina of T1. However, all other features support its placement in the *flavipes*-group. The very strong oblique mesoscutellar rugae in females and granulate sculpture on the mesoscutellum in males, make this species relative easy to recognize.

#### Oxyscelio oblongiclypei

Burks sp. n.

http://zoobank.org/8748F43B-0F29-4708-A7C4-59788E05146A

urn:lsid:biosci.ohio-state.edu:osuc_concepts:307093

http://species-id.net/wiki/Oxyscelio_oblongiclypei

**Figures 231–234.**
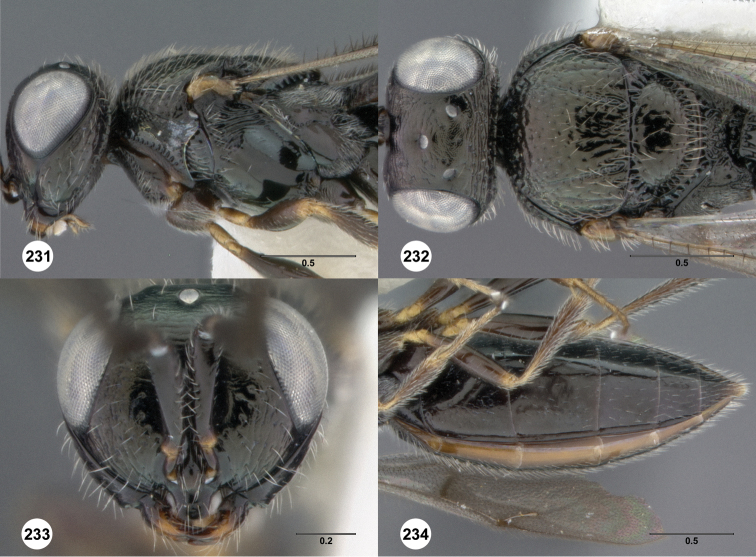
*Oxyscelio oblongiclypei* sp. n., paratype female (OSUC 438851) **231** Head and mesosoma, lateral view **232** Head and mesosoma, dorsal view **233** Lower face, anterior view **234** Metasomal apex, dorsal view. Morphbank^72^

Figures 231–234 ; Morphbank^72^

##### Description.

Female. Body length 4–4.15 mm (n=5).

Radicle color and shade: same as scape, both dark brown. Pedicel color: same as scape. A3: longer than pedicel. A4: broader than long. A5: broader than long.

Ventral clypeal margin: with elongate median lobe. Interantennal process: not elongate. Lower frons at dorsal margin of interantennal process: without transverse carina. Transverse curved rugae extending from frontal depression to eye: absent. Median longitudinal carina in frontal depression: absent. Ventral portion of frontal depression: smooth. Dorsal portion of frontal depression: with medially interrupted transverse carinae. Submedian carina: present only as a weak shift in elevation. Frontal depression dorsally: not hood-like, open dorsally. Upper frons major sculpture: umbilicate foveate; transversely rugose. Upper frons microsculpture: granulate. Hyperoccipital carina: indicated by a set of irregular elevations. Carina connecting occipital carina to hyperoccipital carina: absent. Occipital carina: uniformly rounded dorsally. Occiput sculpture: irregularly sculptured. Extra carina ventral to occipital carina: absent. Gena length: shorter than eye. Major sculpture of gena anteroventrally: rugose; umbilicate punctate. Major sculpture of gena posteroventrally: rugose; umbilicate punctate. Microsculpture of gena anteroventrally: absent. Microsculpture of gena posteroventrally: absent.

Lateral pronotal area sculpture: anteriorly smooth, posterodorsal corner with dense microsculpture, ventral corner with irregular carinae. Posterior border of central pronotal area: directed posteriorly, epomial carina absent or meeting transverse pronotal carina at arch on lateral surface of pronotum. Mesoscutum anteriorly: not steep, forming less than a right angle. Major sculpture of mesoscutal midlobe anteriorly: umbilicate punctate; irregularly rugose. Mesoscutal midlobe sculpture at midlength: not different from nearby sculpture. Major sculpture of mesoscutal midlobe posteriorly: longitudinally rugose; umbilicate punctate. Microsculpture of mesoscutal midlobe anteriorly: absent. Microsculpture of mesoscutal midlobe posteriorly: absent. Median mesoscutal carina: absent. Major sculpture of mesoscutellum centrally: umbilicate punctate. Major sculpture of mesoscutellum peripherally: umbilicate punctate. Microsculpture of mesoscutellum centrally: absent. Microsculpture of mesoscutellum peripherally: absent. Mesoscutellar rim: not expanded. Mesoscutellar rim medially: without notch. Mesofemoral depression: smooth. Metascutellum shape: slightly emarginate posteriorly, concave but elevated posteriorly. Metascutellar setae: present. Metascutellum sculpture: dense and irregular. Postmarginal vein: present. Fore wing apex at rest: reaching middle of T5. Coxae color brightness: same color as femora. Spines along tibiae: absent. Lateral propodeal carinae: broadly separated, but parallel for a short distance anteriorly. Setae in metasomal depression: absent. Anterior sculpture of metasomal depression: with median areole or pair of pits. Median propodeal carina: present.

T1 horn: absent. Number of longitudinal carinae of T1 midlobe: more than 6. T1 lateral carina: straight. T2 sculpture: mostly smooth with setiferous puncta medially, laterally with some longitudinal carinae. T2 sublateral longitudinal foveae: absent. T3 metasomal flanges: absent. T4 sculpture: mostly smooth, with tiny umbilicate foveae. T4 metasomal flanges: absent. T5 sculpture: mostly smooth, with tiny umbilicate foveae. T5 metasomal flanges: absent. T6: broader than long. Major sculpture of T6: umbilicate punctate. Microsculpture of T6: granulate. T6 medially: flat and tapering to a rounded apex, not separated from apical rim. T6 metasomal flanges: absent. T6 raised peripheral rim: absent. S4 sculpture: smooth, with tiny umbilicate foveae. S5 sculpture: smooth, with tiny umbilicate foveae. S5 median carina: absent. S6 peripheral carina: absent. S6 apex in relation to T6: not exposed to dorsal view. S6 apex: rounded or acuminate.

*Male*. unknown.

##### Diagnosis.

Both sexes: Body and limbs entirely dark brown. Frontal depression nearly flat, not crossed by carinae; submedian carina absent or weakly indicated by some rugae. Clypeus elongate. Hyperoccipital carina indicated by rugae. Occipital carina complete convex medially. Mesoscutellum smooth, with only some scattered setae. Metascutellum dorsally setose, broad and short, rugose. Postmarginal vein present. Metasomal depression sculptured antero-medially. T1 lateral carina not expanded. Metasomal sterna without longitudinal rugae or carinae. Metasomal flanges absent. Female: A3 longer than pedicel. A5 broader than long. T1 midlobe with 6 or more longitudinal carinae. T6 broader than long, apical rim not separated from main body of tergum. *Oxyscelio oblongiclypei* resembles *Oxyscelio leviventris* in many ways, but is very distinctive due to its setose metascutellum and elongate clypeus.

##### Etymology.

Latin noun, genitive case, meaning “oblong clypeus.”

##### Link to distribution map.

[http://hol.osu.edu/map-full.html?id=307093]

##### Material examined.

Holotype, female: AUSTRALIA: TAS, 14km S Bronte Park, 42°15'S, 146°29'E, 15.I–3.II.1983, malaise trap, I. Naumann & J. Cardale, OSUC 438850 (deposited in ANIC). Paratypes: AUSTRALIA: 5 females, OSUC 438851-438853, 453958 (ANIC); OSUC 438854 (TDAH).

#### Oxyscelio obturationis

Burks sp. n.

http://zoobank.org/9D754777-E030-44AA-927F-60E971B43DD7

urn:lsid:biosci.ohio-state.edu:osuc_concepts:307094

http://species-id.net/wiki/Oxyscelio_obturationis

**Figures 235–240.**
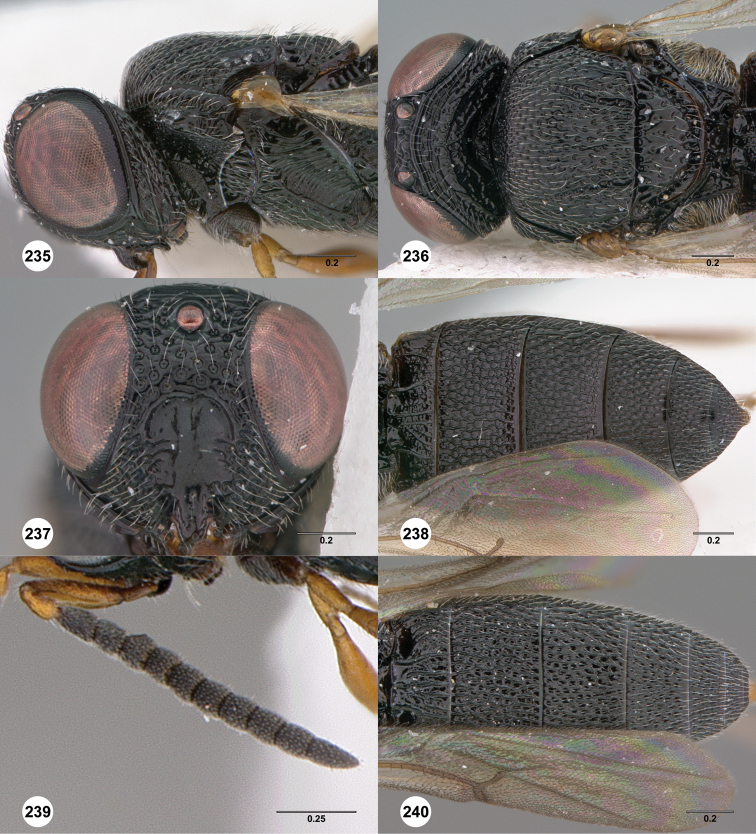
*Oxyscelio obturationis* sp. n., holotype female (OSUC 439017) **235** Head and mesosoma, lateral view **236** Head and mesosoma, dorsal view **237** Head, anterior view **238** Metasoma, dorsal view. Paratype male (OSUC 442295) **239** Antenna **240** Metasoma, dorsal view. Morphbank^73^

Figures 235–240 ; Morphbank^73^

##### Description.

Female. Body length 2.8–3.8 mm (n=20).

Radicle color and shade: same as scape, both yellowish or reddish. Pedicel color: same as scape. A3: shorter than pedicel; as long as pedicel. A4: broader than long. A5: broader than long.

Ventral clypeal margin: with slightly convex median lobe. Interantennal process: not elongate. Lower frons at dorsal margin of interantennal process: without transverse carina. Transverse curved rugae extending from frontal depression to eye: absent. Median longitudinal carina in frontal depression: absent. Ventral portion of frontal depression: smooth. Dorsal portion of frontal depression: without transverse carinae. Submedian carina: present only as a weak shift in elevation. Frontal depression dorsally: not hood-like, open dorsally. Upper frons major sculpture: umbilicate foveate; irregularly rugose. Upper frons microsculpture: absent. Hyperoccipital carina: present as multiple regular elevations. Carina connecting occipital carina to hyperoccipital carina: absent. Occipital carina: uniformly rounded dorsally. Occiput sculpture: irregularly sculptured. Extra carina ventral to occipital carina: absent. Gena length: shorter than eye. Major sculpture of gena anteroventrally: umbilicate foveate. Major sculpture of gena posteroventrally: umbilicate foveate; rugose. Microsculpture of gena anteroventrally: absent. Microsculpture of gena posteroventrally: absent.

Lateral pronotal area sculpture: anteriorly smooth, posterodorsal corner with dense microsculpture, ventral corner with irregular carinae. Posterior border of central pronotal area: directed posteriorly, epomial carina absent or meeting transverse pronotal carina at arch on lateral surface of pronotum. Mesoscutum anteriorly: very steep and tall, descending at a right angle or protruding anteriorly. Major sculpture of mesoscutal midlobe anteriorly: umbilicate foveate. Mesoscutal midlobe sculpture at midlength: not different from nearby sculpture. Major sculpture of mesoscutal midlobe posteriorly: longitudinally rugose. Microsculpture of mesoscutal midlobe anteriorly: granulate. Microsculpture of mesoscutal midlobe posteriorly: absent. Median mesoscutal carina: present as a ruga. Major sculpture of mesoscutellum centrally: umbilicate foveate. Major sculpture of mesoscutellum peripherally: umbilicate foveate. Microsculpture of mesoscutellum centrally: absent. Microsculpture of mesoscutellum peripherally: absent. Mesoscutellar rim: not expanded. Mesoscutellar rim medially: without notch. Mesofemoral depression: longitudinally striate dorsally, smooth ventrally. Metascutellum shape: not emarginate, concave but elevated posteriorly. Metascutellar setae: absent. Metascutellum sculpture: with large smooth posterior fovea. Postmarginal vein: present. Fore wing apex at rest: reaching middle of T6. Coxae color brightness: darker than femora. Spines along tibiae: absent. Lateral propodeal carinae: broadly separated, but parallel for a short distance anteriorly. Setae in metasomal depression: absent. Anterior sculpture of metasomal depression: absent. Median propodeal carina: absent.

T1 horn: absent. Number of longitudinal carinae of T1 midlobe: obscured by other raised sculpture. T1 lateral carina: protruding laterally, visible from ventral view. T2 sculpture: densely foveolate, longitudinal sculpture irregular. T2 sublateral longitudinal foveae: absent. T3 metasomal flanges: absent. T4 sculpture: longitudinally striate to rugose, setal pits spanning interspaces. T4 metasomal flanges: absent. T5 sculpture: longitudinally striate to rugose, setal pits spanning interspaces. T5 metasomal flanges: absent. T6: broader than long. Major sculpture of T6: umbilicate punctate. Microsculpture of T6: absent. T6 medially: flat and tapering to a rounded apex, not separated from apical rim. T6 metasomal flanges: absent. T6 raised peripheral rim: absent. S4 sculpture: longitudinally striate or rugose, setal pits spanning interspaces. S5 sculpture: longitudinally striate to rugose, setal pits spanning interspaces. S5 median carina: absent. S6 peripheral carina: absent. S6 apex in relation to T6: not exposed to dorsal view. S6 apex: rounded or acuminate.

*Male*. Body length 3.3 mm (n=1). A3: longer than pedicel. A5 tyloid shape: narrow, linear. A6: broader than long. A11: longer than broad. Major sculpture of mesoscutal midlobe anteriorly: umbilicate foveate. Major sculpture of mesoscutal midlobe posteriorly: umbilicate foveate; longitudinally rugose. Microsculpture of mesoscutal midlobe anteriorly: granulate. Microsculpture of mesoscutal midlobe posteriorly: absent. Major sculpture of mesoscutellum centrally: umbilicate foveate. Major sculpture of mesoscutellum peripherally: umbilicate foveate. Microsculpture of mesoscutellum centrally: punctate. Microsculpture of mesoscutellum peripherally: absent. Fore wing apex at rest: reaching apex of T5. T1 midlobe longitudinal carinae: 4. T3 metasomal flanges: absent. T4 metasomal flanges: absent. T5 metasomal flanges: absent. T6 metasomal flanges: absent. T7: weakly emarginate.

##### Diagnosis.

Both sexes: Mesoscutum and mesoscutellum black. Frontal depression shallow, transverse carinae absent or interrupted; submedian carina indicated by a set of irregular elevations or weak carinae. Hyperoccipital carina indicated by sharp carina. Occipital carina complete, convex medially. Mesoscutal midlobe with many fine longitudinal rugae posteriorly. Metascutellum deeply concave, broad and short, projecting dorsally. Coxa darker than rest of leg. T1 lateral carina expanded laterally. Metasomal flanges absent. Female: A3 longer than pedicel. A4, A5 broader than long. T1 midlobe with 4 longitudinal carinae. T6 without metasomal flanges. Fore wing long enough to reach middle of T6 or exceed metasomal apex. Male: A4, A11 longer than broad. T1 midlobe with 4 longitudinal carinae. Fore wing long enough to reach metasomal apex. T7 much broader than long, slightly concave apically, without posterior projections. *Oxyscelio obturationis* is very similar to *Oxyscelio scutorum*, and in some specimens can have raised mesoscutellar sculpture similar to that species. However, the strong rows of longitudinal rugae and overall stouter body shape can help distinguish these species.

##### Etymology.

Latin noun, genitive case, meaning “a plug.” Refers to the compact, stout body shape.

##### Link to distribution map.

[http://hol.osu.edu/map-full.html?id=307094]

##### Material examined.

Holotype, female: AUSTRALIA: QLD, via Samford, open forest, Stony Creek, 27°20'S, 152°48'E, 2.II–8.IV.1995, interception trap, H. Janetzki & G. Monteith, OSUC 439017 (deposited in QMBA). Paratypes: AUSTRALIA: 26 females, 1 male, ANIC DB 32-020096, 32-020114, 32-020115, 32-020116, 32-020117, OSUC 439004, OSUC 439005, OSUC 439006, OSUC 439007, OSUC 439008, OSUC 439009, OSUC 439010, OSUC 439011, OSUC 439012, OSUC 439013, OSUC 439014, OSUC 439015, OSUC 439016, OSUC 439018, OSUC 439019, OSUC 439022 (ANIC); OSUC 451324 (QDPC); OSUC 439020, QM Reg. No. T35153 (QMBA); OSUC 439021, 439023, 451323 (WINC).

#### Oxyscelio oculi

Burks sp. n.

http://zoobank.org/4BC03313-37D8-4322-9FED-245C696C8607

urn:lsid:biosci.ohio-state.edu:osuc_concepts:307095

http://species-id.net/wiki/Oxyscelio_oculi

**Figures 241–246.**
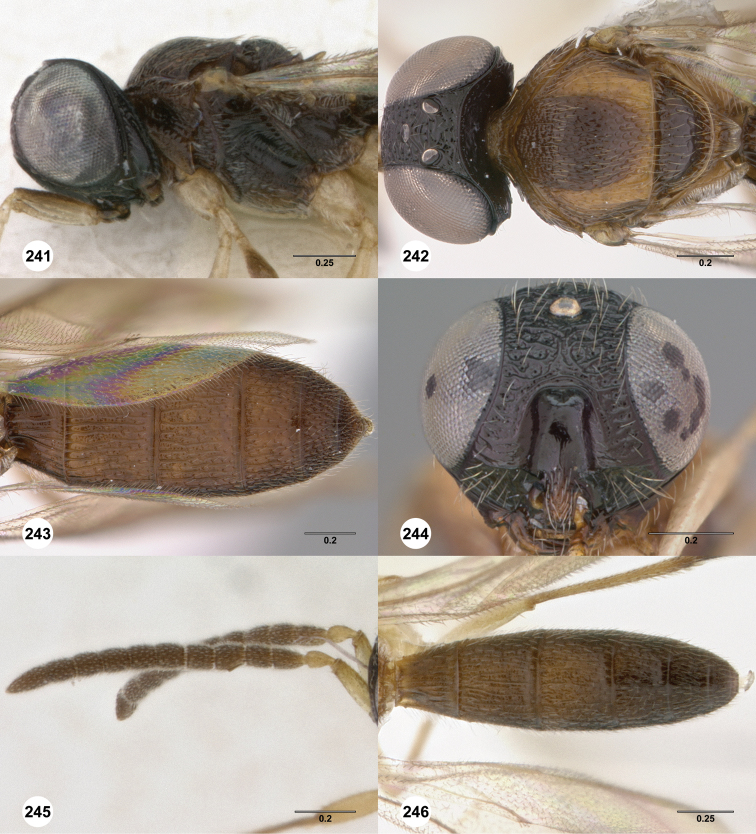
*Oxyscelio oculi* sp. n., holotype female (OSUC 442277) **241** Head and mesosoma, lateral view **242** Head and mesosoma, dorsal view **243** Head, anterior view **244** Metasoma, dorsal view. Paratype male (OSUC 442295) **245** Antenna **246** Mesosoma, dorsal view. Morphbank^74^

Figures 241–246 ; Morphbank^74^

##### Description.

Female. Body length 2.7–3.2 mm (n=20).

Radicle color and shade: same as scape, both yellowish or reddish. Pedicel color: same as scape. A3: shorter than pedicel; as long as pedicel. A4: longer than broad. A5: longer than broad.

Ventral clypeal margin: concave. Interantennal process: not elongate. Lower frons at dorsal margin of interantennal process: without transverse carina. Transverse curved rugae extending from frontal depression to eye: absent. Median longitudinal carina in frontal depression: absent. Ventral portion of frontal depression: smooth. Dorsal portion of frontal depression: without transverse carinae. Submedian carina: present. Frontal depression dorsally: not hood-like, open dorsally. Upper frons major sculpture: umbilicate foveate; transversely rugose. Upper frons microsculpture: granulate. Hyperoccipital carina: present as a single carina. Carina connecting occipital carina to hyperoccipital carina: absent. Occipital carina: present laterally, absent medially. Occiput sculpture: smooth. Extra carina ventral to occipital carina: present, complete. Gena length: shorter than eye. Major sculpture of gena anteroventrally: rugose; umbilicate punctate. Major sculpture of gena posteroventrally: rugose; umbilicate punctate. Microsculpture of gena anteroventrally: granulate. Microsculpture of gena posteroventrally: granulate.

Lateral pronotal area sculpture: irregularly sculptured. Posterior border of central pronotal area: directed posteriorly, epomial carina absent or meeting transverse pronotal carina at arch on lateral surface of pronotum. Mesoscutum anteriorly: very steep and tall, descending at a right angle or protruding anteriorly. Major sculpture of mesoscutal midlobe anteriorly: umbilicate punctate. Mesoscutal midlobe sculpture at midlength: not different from nearby sculpture. Major sculpture of mesoscutal midlobe posteriorly: umbilicate foveate; longitudinally rugose. Microsculpture of mesoscutal midlobe anteriorly: granulate. Microsculpture of mesoscutal midlobe posteriorly: absent. Median mesoscutal carina: absent. Major sculpture of mesoscutellum centrally: umbilicate foveate; umbilicate punctate. Major sculpture of mesoscutellum peripherally: umbilicate punctate. Microsculpture of mesoscutellum centrally: absent. Microsculpture of mesoscutellum peripherally: absent; granulate. Mesoscutellar rim: not expanded. Mesoscutellar rim medially: without notch. Mesofemoral depression: longitudinally striate dorsally, smooth ventrally. Metascutellum shape: slightly emarginate posteriorly, concave but elevated posteriorly. Metascutellar setae: absent. Metascutellum sculpture: with large smooth posterior fovea. Postmarginal vein: present. Fore wing apex at rest: reaching middle of T6. Coxae color brightness: same color as femora. Spines along tibiae: absent. Lateral propodeal carinae: meeting near propodeal midlength. Setae in metasomal depression: absent. Anterior sculpture of metasomal depression: with series of longitudinal carinae extending to lateral propodeal carinae. Median propodeal carina: absent.

T1 horn: absent. Number of longitudinal carinae of T1 midlobe: 4. T1 lateral carina: straight. T2 sculpture: densely foveolate, longitudinal sculpture irregular. T2 sublateral longitudinal foveae: absent. T3 metasomal flanges: absent. T4 sculpture: longitudinally striate to rugose, setal pits spanning interspaces. T4 metasomal flanges: absent. T5 sculpture: longitudinally striate to rugose, setal pits spanning interspaces. T5 metasomal flanges: absent. T6: broader than long. Major sculpture of T6: umbilicate punctate. Microsculpture of T6: absent. T6 medially: flat and tapering to a rounded apex, not separated from apical rim. T6 metasomal flanges: absent. T6 raised peripheral rim: absent. S4 sculpture: densely setose, setal pits between very weak longitudinal rugae. S5 sculpture: densely setose, setal pits between very weak longitudinal rugae. S5 median carina: absent. S6 peripheral carina: absent. S6 apex in relation to T6: not exposed to dorsal view. S6 apex: rounded or acuminate.

*Male*. Body length 2.65–3.15 mm (n=20). A3: longer than pedicel. A5 tyloid shape: narrow, linear. A6: longer than broad. A11: longer than broad. Major sculpture of mesoscutal midlobe anteriorly: umbilicate punctate. Major sculpture of mesoscutal midlobe posteriorly: umbilicate foveate; longitudinally rugose. Microsculpture of mesoscutal midlobe anteriorly: granulate. Microsculpture of mesoscutal midlobe posteriorly: absent. Major sculpture of mesoscutellum centrally: umbilicate foveate; longitudinally rugose. Major sculpture of mesoscutellum peripherally: umbilicate foveate; longitudinally rugose. Microsculpture of mesoscutellum centrally: absent. Microsculpture of mesoscutellum peripherally: absent. Fore wing apex at rest: exceeding metasomal apex. T1 midlobe longitudinal carinae: 3. T3 metasomal flanges: absent. T4 metasomal flanges: absent. T5 metasomal flanges: absent. T6 metasomal flanges: absent. T7: truncate.

##### Diagnosis.

Both sexes: Frontal depression nearly parallel-sided, transverse carinae absent; submedian carina sharp. Hyperoccipital carina sharp and strong. Occipital carina incomplete, lateral portions almost reaching hyperoccipital carina; occiput smooth, with only a few setiferous puncta and fine rugae. Metascutellum with a small concave postero-medial area, laterally with broad longitudinally striate area, weakly emarginate, projecting dorsally. Postmarginal vein present. Coxa not darker than rest of leg. T1 lateral carina not expanded laterally. Female: A3 longer than pedicel. A4 longer than broad, A5 nearly as long as broad. Mesoscutellum nearly smooth, with a few setiferous puncta. T1 midlobe with 5 longitudinal carinae. Fore wing long enough to reach or exceed T6. Male: All flagellomeres longer than broad. T1 midlobe with 3 longitudinal carinae, hardly raised above sidelobes. Mesoscutellum with strong longitudinal rugae and no granulate sculpture. Fore wing long enough to reach to or beyond T7. T7 truncate, steeply sloping.

##### Etymology.

Latin noun, genitive case, meaning “eye.”

##### Link to distribution map.

[http://hol.osu.edu/map-full.html?id=307095]

##### Material examined.

Holotype, female: AUSTRALIA: QLD, Mount Glorious, 1977, malaise trap, OSUC 442277 (deposited in QMBA). Paratypes: AUSTRALIA: 35 females, 36 males, Australian Museum K245262, K245267, K245268, K245270, K245274 (AMSA); ANIC DB 32-020075, 32-020076 (ANIC); OSUC 227548, 227608, 462563-462566, 462727-462728 (CNCI); OSUC 442273, 442278, 442301-442302, 451276-451277, QDPC 0-165656, QDPC 0-165658 (QDPC); OSUC 442298 (QMBA); OSUC 359782-359787, 442271-442272, 442274-442276, 442279-442297, 442299-442300, 442303, 442305-442310, 451274-451275, 451278-451283 (WINC).

##### Comments.

*Oxyscelio oculi* is part of a species complex characterized by a weakly developed T1 midlobe in males but vaulted T1 in females, a very smooth occiput, a short and convex mesoscutellum, and large eyes. Distinction between *Oxyscelio oculi* and *Oxyscelio croci* is chiefly based on mesoscutellar surface sculpture. Males in this complex but not fitting the above diagnosis occur, and are not currently assigned to species. These imply that undetected sibling species in this complex may exist.

#### Oxyscelio palati

Burks sp. n.

http://zoobank.org/55FF021F-1929-4505-BA9E-5DD92CB9DD9C

urn:lsid:biosci.ohio-state.edu:osuc_concepts:307096

http://species-id.net/wiki/Oxyscelio_palati

**Figures 247–252.**
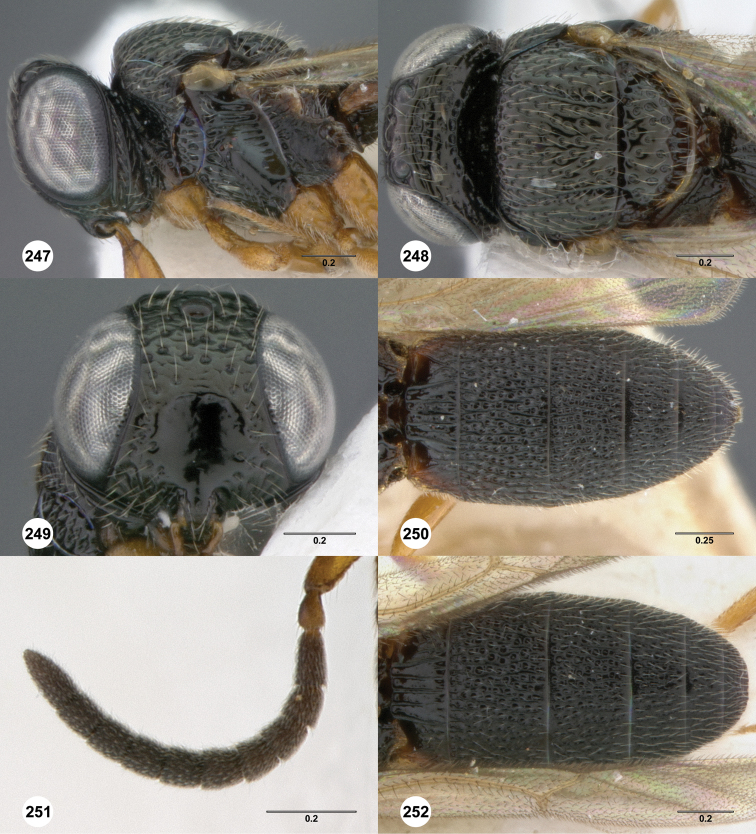
*Oxyscelio palati* sp. n., paratype female (OSUC 359587) **247** Head and mesosoma, lateral view **248** Head and mesosoma, dorsal view **249** Head, anterior view. Paratype female (ANIC DB 32-020100) **250** Metasoma, dorsal view. Paratype male (OSUC 359596) **251** Antenna **252** Metasoma, dorsal view. Morphbank^75^

Figures 247–252 ; Morphbank^75^

##### Description.

Female. Body length 2.5–3.4 mm (n=18).

Radicle color and shade: same as scape, both yellowish or reddish. Pedicel color: same as scape. A3: longer than pedicel. A4: longer than broad. A5: broader than long.

Ventral clypeal margin: with slightly convex median lobe. Interantennal process: not elongate. Lower frons at dorsal margin of interantennal process: without transverse carina. Transverse curved rugae extending from frontal depression to eye: absent. Median longitudinal carina in frontal depression: absent. Ventral portion of frontal depression: smooth. Dorsal portion of frontal depression: without transverse carinae. Submedian carina: present only as a weak shift in elevation. Frontal depression dorsally: not hood-like, open dorsally. Upper frons major sculpture: umbilicate foveate; transversely rugose. Upper frons microsculpture: absent. Hyperoccipital carina: indicated by a set of irregular elevations. Carina connecting occipital carina to hyperoccipital carina: absent. Occipital carina: weakly arched dorsally, with sharp lateral corners; omicron-shaped, with sharp corners where median portion meets lateral portions. Occiput sculpture: transversely rugose. Extra carina ventral to occipital carina: absent. Gena length: shorter than eye. Major sculpture of gena anteroventrally: umbilicate foveate. Major sculpture of gena posteroventrally: umbilicate foveate; rugose; absent. Microsculpture of gena anteroventrally: absent. Microsculpture of gena posteroventrally: absent.

Lateral pronotal area sculpture: densely covered with setiferous puncta. Posterior border of central pronotal area: directed anteriorly, protruding at corner of epomial carina and transverse pronotal carina. Mesoscutum anteriorly: very steep and tall, descending at a right angle or protruding anteriorly. Major sculpture of mesoscutal midlobe anteriorly: umbilicate foveate; irregularly rugose. Mesoscutal midlobe sculpture at midlength: not different from nearby sculpture. Major sculpture of mesoscutal midlobe posteriorly: umbilicate foveate; irregularly rugose. Microsculpture of mesoscutal midlobe anteriorly: absent. Microsculpture of mesoscutal midlobe posteriorly: absent. Median mesoscutal carina: present as a vague, occasionally interrupted elevation. Major sculpture of mesoscutellum centrally: umbilicate foveate; irregularly rugose. Major sculpture of mesoscutellum peripherally: umbilicate foveate; irregularly rugose. Microsculpture of mesoscutellum centrally: absent; punctate. Microsculpture of mesoscutellum peripherally: absent. Mesoscutellar rim: not expanded. Mesoscutellar rim medially: without notch. Mesofemoral depression: with slight, indistinct sculpture dorsally, smooth ventrally. Metascutellum shape: deeply emarginate, with the resulting pair of posterior processes subtriangular and directed dorsally. Metascutellar setae: absent. Metascutellum sculpture: with large smooth posterior fovea. Postmarginal vein: absent. Fore wing apex at rest: exceeding metasomal apex. Coxae color brightness: same color as femora. Spines along tibiae: absent. Lateral propodeal carinae: broadly separated, not parallel anteriorly. Setae in metasomal depression: absent. Anterior sculpture of metasomal depression: absent. Median propodeal carina: absent.

T1 horn: absent. Number of longitudinal carinae of T1 midlobe: 5. T1 lateral carina: protruding laterally, visible from ventral view. T2 sculpture: densely foveolate, longitudinal sculpture irregular. T2 sublateral longitudinal foveae: absent. T3 metasomal flanges: absent. T4 sculpture: longitudinally striate to rugose, setal pits spanning interspaces. T4 metasomal flanges: absent. T5 sculpture: longitudinally striate to rugose, setal pits spanning interspaces. T5 metasomal flanges: absent. T6: broader than long. Major sculpture of T6: umbilicate punctate. Microsculpture of T6: absent. T6 medially: slightly emarginate, not separated from apical rim. T6 metasomal flanges: absent. T6 raised peripheral rim: absent. S4 sculpture: longitudinally striate or rugose, setal pits spanning interspaces. S5 sculpture: longitudinally striate to rugose, setal pits spanning interspaces. S5 median carina: absent. S6 peripheral carina: absent. S6 apex in relation to T6: not exposed to dorsal view. S6 apex: rounded or acuminate.

*Male*. Body length 2.45–3.25 mm (n=19). A3: longer than pedicel. A5 tyloid shape: narrow, linear. A6: broader than long. A11: longer than broad. Major sculpture of mesoscutal midlobe anteriorly: umbilicate foveate. Major sculpture of mesoscutal midlobe posteriorly: umbilicate foveate; irregularly rugose. Microsculpture of mesoscutal midlobe anteriorly: absent; granulate. Microsculpture of mesoscutal midlobe posteriorly: absent. Major sculpture of mesoscutellum centrally: umbilicate foveate; irregularly rugose. Major sculpture of mesoscutellum peripherally: umbilicate foveate; irregularly rugose. Microsculpture of mesoscutellum centrally: absent. Microsculpture of mesoscutellum peripherally: absent. Fore wing apex at rest: exceeding metasomal apex. T1 midlobe longitudinal carinae: 4. T3 metasomal flanges: absent. T4 metasomal flanges: absent. T5 metasomal flanges: absent. T6 metasomal flanges: absent. T7: weakly emarginate.

##### Diagnosis.

Both sexes: Frontal depression shallow; transverse carinae present ventrally, interrupted; submedian carina very weak or absent medially. Hyperoccipital carina indicated by rugae. Occipital carina complete, broadly omicron-shaped medially. Metascutellum deeply concave, emarginate apically, projecting dorsally. Postmarginal vein absent. Coxa not darker than rest of leg. T1 lateral carina expanded laterally. Metasomal flanges absent. Female: A4 longer than broad, A5 almost as long as broad. Mesoscutellum without granulate sculpture. T1 midlobe with 4 longitudinal carinae. Main body of T6 not abruptly separated from apical rim, T6 slightly concave apically. Fore wing long enough to reach beyond metasomal apex. Male: All flagellomeres longer than broad. T1 midlobe with 4 longitudinal carinae. Fore wing long enough to reach beyond metasomal apex. T7 tiny, truncate. *Oxyscelio palati* is very similar to *Oxyscelio hyalinipennis*, but differs in having an omicron-shaped occipital carina that has straight connections between the median and lateral portions. It also differs in having a longer antenna, especially in males, and in completely lacking a postmarginal vein. In *Oxyscelio hyalinipennis*, the venation reaches the wing margin and has a more or less distinct posterior bend indicating the postmarginal vein, but in *Oxyscelio palati* the venation does not quite reach the anterior wing margin. In known specimens of *Oxyscelio palati*, the metanotum and posterior rim of the mesoscutellum are amber-colored, in stark contrast to the darker surrounding areas.

##### Etymology.

Latin noun, genitive case, meaning “vault.”

##### Link to distribution map.

[http://hol.osu.edu/map-full.html?id=307096]

##### Material examined.

Holotype, female: AUSTRALIA: QLD, cableway base station, Bellenden Ker Range, 100m, 17.X–9.XI.1981, window trap, OSUC 359578 (deposited in QMBA). Paratypes: AUSTRALIA: 17 females, 19 males, ANIC DB 32-020100, 32-020145, OSUC 359583, OSUC 359584, OSUC 359585, OSUC 359586, OSUC 359587, OSUC 359602, OSUC 359603, OSUC 359604, OSUC 436939 (ANIC); QDPC 0-165695 (QDPC); OSUC 148367, 359582, 359588, 359600-359601 (QMBA); OSUC 359605 (UQIC); OSUC 359575-359577, 359579-359581, 359589-359599, 451356 (WINC).

#### Oxyscelio pectinis

Burks sp. n.

http://zoobank.org/5E8F95AB-A2BD-46B7-B3EC-1602D260EBDE

urn:lsid:biosci.ohio-state.edu:osuc_concepts:307097

http://species-id.net/wiki/Oxyscelio_pectinis

**Figures 253–256.**
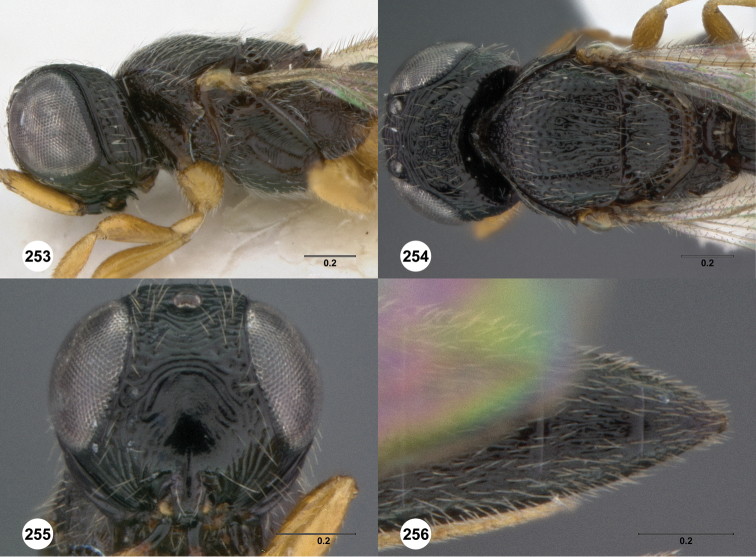
*Oxyscelio pectinis* sp. n., paratype female (OSUC 449001) **253** Head and mesosoma, lateral view. Holotype female (OSUC 368207) **254** Head and mesosoma, dorsal view **255** Head, anterior view **256** Metasoma, dorsal view. Morphbank^76^

Figures 253–256 ; Morphbank^76^

##### Description.

Female. Body length 3.2–3.3 mm (n=2).

Radicle color and shade: darker than scape. Pedicel color: same as scape. A3: shorter than pedicel. A4: as long as broad. A5: broader than long.

Ventral clypeal margin: with slightly convex median lobe. Interantennal process: not elongate. Lower frons at dorsal margin of interantennal process: without transverse carina. Transverse curved rugae extending from frontal depression to eye: absent. Median longitudinal carina in frontal depression: absent. Ventral portion of frontal depression: with medially interrupted transverse foveae. Dorsal portion of frontal depression: without transverse carinae. Submedian carina: present. Frontal depression dorsally: not hood-like, open dorsally. Upper frons major sculpture: umbilicate foveate; transversely rugose. Upper frons microsculpture: absent. Hyperoccipital carina: present as a single carina. Carina connecting occipital carina to hyperoccipital carina: present. Occipital carina: present laterally, absent medially. Occiput sculpture: irregularly sculptured. Extra carina ventral to occipital carina: absent. Gena length: shorter than eye. Major sculpture of gena anteroventrally: rugose; umbilicate punctate. Major sculpture of gena posteroventrally: rugose; umbilicate punctate. Microsculpture of gena anteroventrally: absent. Microsculpture of gena posteroventrally: absent.

Lateral pronotal area sculpture: anteriorly smooth, posterodorsal corner with dense microsculpture, ventral corner with irregular carinae. Posterior border of central pronotal area: directed posteriorly, epomial carina absent or meeting transverse pronotal carina at arch on lateral surface of pronotum. Mesoscutum anteriorly: not steep, forming less than a right angle. Major sculpture of mesoscutal midlobe anteriorly: umbilicate foveate. Mesoscutal midlobe sculpture at midlength: not different from nearby sculpture. Major sculpture of mesoscutal midlobe posteriorly: umbilicate foveate; longitudinally rugose. Microsculpture of mesoscutal midlobe anteriorly: granulate. Microsculpture of mesoscutal midlobe posteriorly: absent. Median mesoscutal carina: present as a ruga. Major sculpture of mesoscutellum centrally: longitudinally rugose; umbilicate punctate. Major sculpture of mesoscutellum peripherally: longitudinally rugose; umbilicate punctate. Microsculpture of mesoscutellum centrally: absent. Microsculpture of mesoscutellum peripherally: absent. Mesoscutellar rim: not expanded. Mesoscutellar rim medially: without notch. Mesofemoral depression: longitudinally striate dorsally and ventrally. Metascutellum shape: slightly emarginate posteriorly, concave but elevated posteriorly. Metascutellar setae: absent. Metascutellum sculpture: with large smooth posterior fovea. Postmarginal vein: present. Fore wing apex at rest: reaching near apex of T5. Coxae color brightness: same color as femora. Spines along tibiae: absent. Lateral propodeal carinae: broadly separated, not parallel anteriorly. Setae in metasomal depression: absent. Anterior sculpture of metasomal depression: absent. Median propodeal carina: absent.

T1 horn: present. Number of longitudinal carinae of T1 midlobe: 5. T1 lateral carina: straight. T2 sculpture: densely foveolate, longitudinal sculpture irregular. T2 sublateral longitudinal foveae: absent. T3 metasomal flanges: absent. T4 sculpture: longitudinally striate to rugose, setal pits spanning interspaces. T4 metasomal flanges: absent. T5 sculpture: longitudinally striate to rugose, setal pits spanning interspaces. T5 metasomal flanges: absent. T6: broader than long. Major sculpture of T6: umbilicate punctate. Microsculpture of T6: absent. T6 medially: flat and tapering to a rounded apex, not separated from apical rim. T6 metasomal flanges: absent. T6 raised peripheral rim: absent. S4 sculpture: longitudinally striate or rugose, setal pits spanning interspaces. S5 sculpture: longitudinally striate to rugose, setal pits spanning interspaces. S5 median carina: absent. S6 peripheral carina: absent. S6 apex in relation to T6: not exposed to dorsal view. S6 apex: rounded or acuminate.

*Male*. unknown.

##### Diagnosis.

Frontal depression shallow, transverse carinae absent; submedian carina weak. Hyperoccipital carina indicated by rugae. Occipital carina connected to hyperoccipital carina by a weak longitudinal carina, laterally with strong corners and medially absent; area between occipital and hyperoccipital carinae densely sculptured and having many short setae. Mesoscutellum and mesoscutal midlobe posteriorly with strong longitudinal rugae. Metascutellum exceedingly tiny, concave. Postmarginal vein present. Coxa not darker than rest of leg. Metasomal depression smooth. T1 lateral carina not expanded laterally. Metasomal flanges absent. Female only: A3 shorter than pedicel. A4 about as long as broad, A5 broader than long. T1 midlobe slight smooth area obscuring its longitudinal carinae. Fore wing long enough to reach middle of T6. T6 slightly broader than long.

##### Etymology.

Latin noun, genitive case, meaning “comb.”

##### Link to distribution map.

[http://hol.osu.edu/map-full.html?id=307097]

##### Material examined.

Holotype, female: AUSTRALIA: QLD, rainforest, Mount Glorious, 28.II.1984, sweeping, I. D. Galloway, OSUC 368207 (deposited in QMBA). Paratypes: AUSTRALIA: 2 females, QDPC 0-165773 (QDPC); OSUC 449001 (WINC).

##### Comments.

*Oxyscelio pectinis* is easily recognized by its very striking metascutellar sculpture. Other features reinforce its distinction from other members of the *aciculae*-group, but it is most similar to *Oxyscelio divisionis*.

#### Oxyscelio pollicis

Burks sp. n.

http://zoobank.org/0BA34EB7-1375-4AD2-B851-14791514C00F

urn:lsid:biosci.ohio-state.edu:osuc_concepts:307098

http://species-id.net/wiki/Oxyscelio_pollicis

**Figures 257–262.**
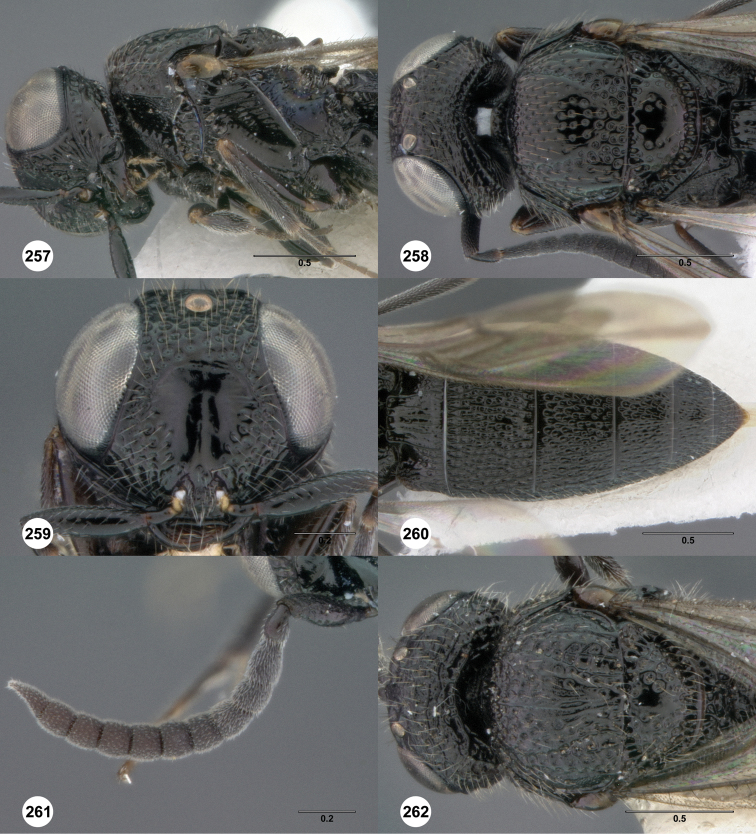
*Oxyscelio pollicis* sp. n., paratype female (OSUC 439551) **257** Head and mesosoma, lateral view. Paratype female (OSUC 439550) **258** Head and mesosoma, dorsal view **259** Head, anterior view **260** Metasoma, dorsal view. Paratype male (OSUC 439553) **261** Antenna **262** Metasoma, dorsal view. Morphbank^77^

Figures 257–262 ; Morphbank^77^

##### Description.

Female. Body length 3.25–3.9 mm (n=14).

Radicle color and shade: same as scape, both dark brown. Pedicel color: same as scape. A3: longer than pedicel. A4: longer than broad. A5: broader than long.

Ventral clypeal margin: with slightly convex median lobe. Interantennal process: not elongate. Lower frons at dorsal margin of interantennal process: without transverse carina. Transverse curved rugae extending from frontal depression to eye: absent. Median longitudinal carina in frontal depression: absent. Ventral portion of frontal depression: smooth. Dorsal portion of frontal depression: without transverse carinae. Submedian carina: present only as a weak shift in elevation. Frontal depression dorsally: not hood-like, open dorsally. Upper frons major sculpture: umbilicate foveate; transversely rugose. Upper frons microsculpture: absent. Hyperoccipital carina: absent. Carina connecting occipital carina to hyperoccipital carina: absent. Occipital carina: uniformly rounded dorsally. Occiput sculpture: umbilicate foveate. Extra carina ventral to occipital carina: absent. Gena length: shorter than eye. Major sculpture of gena anteroventrally: umbilicate foveate. Major sculpture of gena posteroventrally: umbilicate punctate. Microsculpture of gena anteroventrally: absent. Microsculpture of gena posteroventrally: absent.

Lateral pronotal area sculpture: anteriorly smooth, posterodorsal corner with dense microsculpture, ventral corner with irregular carinae. Posterior border of central pronotal area: directed anteriorly, protruding at corner of epomial carina and transverse pronotal carina. Mesoscutum anteriorly: very steep and tall, descending at a right angle or protruding anteriorly. Major sculpture of mesoscutal midlobe anteriorly: umbilicate foveate. Mesoscutal midlobe sculpture at midlength: not different from nearby sculpture. Major sculpture of mesoscutal midlobe posteriorly: umbilicate foveate. Microsculpture of mesoscutal midlobe anteriorly: granulate. Microsculpture of mesoscutal midlobe posteriorly: absent. Median mesoscutal carina: absent; present as a vague, occasionally interrupted elevation. Major sculpture of mesoscutellum centrally: absent. Major sculpture of mesoscutellum peripherally: umbilicate foveate. Microsculpture of mesoscutellum centrally: absent. Microsculpture of mesoscutellum peripherally: absent. Mesoscutellar rim: not expanded. Mesoscutellar rim medially: without notch. Mesofemoral depression: longitudinally striate dorsally, smooth ventrally. Metascutellum shape: deeply emarginate, with the resulting pair of posterior processes subtriangular and directed dorsally. Metascutellar setae: absent. Metascutellum sculpture: with large smooth posterior fovea. Postmarginal vein: absent. Fore wing apex at rest: exceeding metasomal apex. Coxae color brightness: same color as femora. Spines along tibiae: absent. Lateral propodeal carinae: broadly separated, not parallel anteriorly. Setae in metasomal depression: absent. Anterior sculpture of metasomal depression: absent. Median propodeal carina: absent.

T1 horn: absent. Number of longitudinal carinae of T1 midlobe: obscured by other raised sculpture. T1 lateral carina: protruding laterally, visible from ventral view. T2 sculpture: with longitudinal striae or rugae, setiferous puncta present between them. T2 sublateral longitudinal foveae: absent. T3 metasomal flanges: absent. T4 sculpture: longitudinally striate to rugose, setal pits spanning interspaces. T4 metasomal flanges: absent. T5 sculpture: longitudinally striate to rugose, setal pits spanning interspaces. T5 metasomal flanges: absent. T6: broader than long. Major sculpture of T6: umbilicate foveate. Microsculpture of T6: absent. T6 medially: flat and tapering to a rounded apex, not separated from apical rim. T6 metasomal flanges: absent. T6 raised peripheral rim: absent. S4 sculpture: longitudinally striate or rugose, setal pits spanning interspaces. S5 sculpture: longitudinally striate to rugose, setal pits spanning interspaces. S5 median carina: present. S6 peripheral carina: absent. S6 apex in relation to T6: not exposed to dorsal view. S6 apex: rounded or acuminate.

*Male*. Body length 3.4–3.55 mm (n=3). A3: longer than pedicel. A5 tyloid shape: narrow, linear. A6: broader than long. A11: longer than broad. Major sculpture of mesoscutal midlobe anteriorly: umbilicate foveate; irregularly rugose. Major sculpture of mesoscutal midlobe posteriorly: umbilicate foveate; longitudinally rugose. Microsculpture of mesoscutal midlobe anteriorly: granulate. Microsculpture of mesoscutal midlobe posteriorly: absent. Major sculpture of mesoscutellum centrally: absent. Major sculpture of mesoscutellum peripherally: umbilicate foveate. Microsculpture of mesoscutellum centrally: absent; granulate. Microsculpture of mesoscutellum peripherally: absent. Fore wing apex at rest: exceeding metasomal apex. T1 midlobe longitudinal carinae: 4. T3 metasomal flanges: absent. T4 metasomal flanges: absent. T5 metasomal flanges: absent. T6 metasomal flanges: absent. T7: weakly emarginate.

##### Diagnosis.

Both sexes: Body and limbs entirely dark brown. Frontal depression shallow and broad, transverse carinae absent or interrupted; submedian carina weak. Hyperoccipital carina indicated by rugae. Occipital carina complete, convex medially. Metascutellum subrectangular, deeply concave, emarginate apically, projecting dorsally. T1 lateral carina expanded laterally. Metasomal flanges absent. Female: A3 longer than pedicel. A4 longer than broad; A5, A6 broader than long. T1 midlobe with a slight smooth bump obscuring anterior carinae. Fore wing long enough to reach middle of T5. T6 broader than long, rounded apically. Male: A3 longer than pedicel. most flagellomeres between A4 and A12 broader than long. T1 midlobe with 4 longitudinal midlobe carinae. Fore wing long enough to exceed metasomal apex. T7 much broader than long, blunt apically, without posterior projections.

##### Etymology.

Latin noun, genitive case, meaning “thumb.” Refers to the shape of the fore wing venation.

##### Link to distribution map.

[http://hol.osu.edu/map-full.html?id=307098]

##### Associations.

Under bark of *Acacia* Mill.: [Fabales: Fabaceae]

##### Material examined.

Holotype, female: AUSTRALIA: ACT, Piccadilly Circus, 35°22'S, 148°48'E, 1240m, IV-1984, flight intercept trap/window trough trap, J. Lawrence, T. Weir & M.-L. Johnson, OSUC 439540 (deposited in ANIC). Paratypes: AUSTRALIA: 13 females, 17 males, OSUC 437015, 439541-439554, 445316-445325, 445327, 445329-445332 (ANIC). Other material: AUSTRALIA: 1 female, OSUC 437014 (ANIC).

##### Comments.

*Oxyscelio pollicis* is similar to some other species in the *atricoxa*-group, but it is also one of a set of melanistic species known from New South Wales and the Australian Capital Territory. Other melanistic species from this area include the *concoloripes*-group, and some additional melanistic species occur in Tasmania. It is unlikely that these melanistic species form a monophyletic group.

#### Oxyscelio proceritatis

Burks sp. n.

http://zoobank.org/2F5EF212-9A7A-4662-ACFA-4AE43C2263E9

urn:lsid:biosci.ohio-state.edu:osuc_concepts:307099

http://species-id.net/wiki/Oxyscelio_proceritatis

**Figures 263–266.**
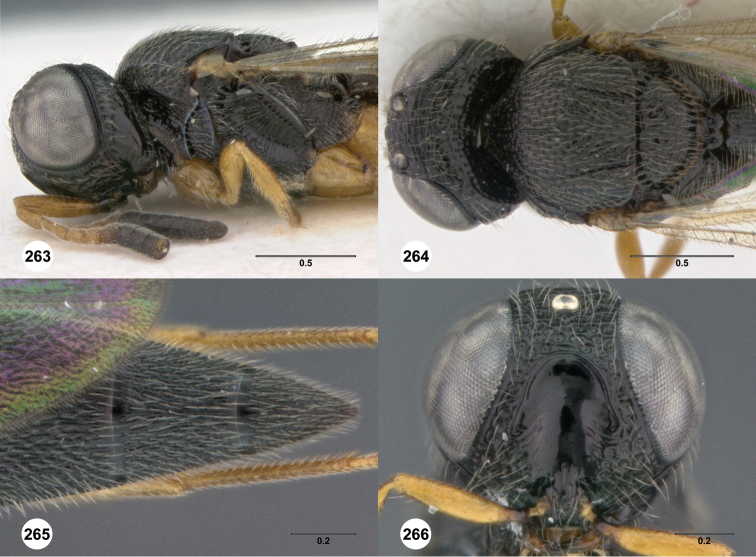
*Oxyscelio proceritatis* sp. n., holotype female (OSUC 368221) **263** Head and mesosoma, lateral view **264** Head and mesosoma, dorsal view **265** Metasoma, dorsal view. Paratype female (OSUC 462572) **266** Head, anterior view. Morphbank^78^

Figures 263–266 ; Morphbank^78^

##### Description.

Female. Body length 4.5–4.7 mm (n=4).

Radicle color and shade: darker than scape. Pedicel color: same as scape. A3: longer than pedicel. A4: longer than broad. A5: longer than broad.

Ventral clypeal margin: concave. Interantennal process: not elongate. Lower frons at dorsal margin of interantennal process: without transverse carina. Transverse curved rugae extending from frontal depression to eye: absent. Median longitudinal carina in frontal depression: absent. Ventral portion of frontal depression: with medially interrupted transverse carinae. Dorsal portion of frontal depression: without transverse carinae. Submedian carina: present. Frontal depression dorsally: not hood-like, open dorsally. Upper frons major sculpture: umbilicate foveate; transversely rugose. Upper frons microsculpture: absent. Hyperoccipital carina: indicated by a set of irregular elevations. Carina connecting occipital carina to hyperoccipital carina: absent. Occipital carina: uniformly rounded dorsally. Occiput sculpture: irregularly sculptured. Extra carina ventral to occipital carina: absent. Gena length: shorter than eye. Major sculpture of gena anteroventrally: umbilicate foveate; absent. Major sculpture of gena posteroventrally: umbilicate foveate; absent. Microsculpture of gena anteroventrally: absent. Microsculpture of gena posteroventrally: absent.

Lateral pronotal area sculpture: irregularly sculptured. Posterior border of central pronotal area: directed posteriorly, epomial carina absent or meeting transverse pronotal carina at arch on lateral surface of pronotum. Mesoscutum anteriorly: not steep, forming less than a right angle. Major sculpture of mesoscutal midlobe anteriorly: umbilicate foveate. Mesoscutal midlobe sculpture at midlength: not different from nearby sculpture. Major sculpture of mesoscutal midlobe posteriorly: umbilicate foveate; longitudinally rugose. Microsculpture of mesoscutal midlobe anteriorly: granulate. Microsculpture of mesoscutal midlobe posteriorly: absent. Median mesoscutal carina: present as a ruga. Major sculpture of mesoscutellum centrally: umbilicate foveate. Major sculpture of mesoscutellum peripherally: umbilicate foveate. Microsculpture of mesoscutellum centrally: absent. Microsculpture of mesoscutellum peripherally: absent. Mesoscutellar rim: not expanded. Mesoscutellar rim medially: without notch. Mesofemoral depression: longitudinally striate dorsally and ventrally. Metascutellum shape: not emarginate, forming a flat, concave shelf. Metascutellar setae: absent. Metascutellum sculpture: dense and irregular. Postmarginal vein: present. Fore wing apex at rest: reaching base of T5. Coxae color brightness: same color as femora. Spines along tibiae: absent. Lateral propodeal carinae: broadly separated, not parallel anteriorly. Setae in metasomal depression: absent. Anterior sculpture of metasomal depression: absent. Median propodeal carina: absent.

T1 horn: present. Number of longitudinal carinae of T1 midlobe: obscured by other raised sculpture. T1 lateral carina: straight. T2 sculpture: densely foveolate, longitudinal sculpture irregular. T2 sublateral longitudinal foveae: absent. T3 metasomal flanges: absent. T4 sculpture: longitudinally striate to rugose, setal pits spanning interspaces. T4 metasomal flanges: absent. T5 sculpture: longitudinally striate to rugose, setal pits spanning interspaces. T5 metasomal flanges: absent. T6: longer than broad. Major sculpture of T6: umbilicate punctate; longitudinally striate. Microsculpture of T6: absent. T6 medially: flat and tapering to a rounded apex, not separated from apical rim. T6 metasomal flanges: absent. T6 raised peripheral rim: absent. S4 sculpture: longitudinally striate or rugose, setal pits spanning interspaces. S5 sculpture: longitudinally striate to rugose, setal pits spanning interspaces. S5 median carina: present. S6 peripheral carina: absent. S6 apex in relation to T6: not exposed to dorsal view. S6 apex: rounded or acuminate.

*Male*. unknown.

##### Diagnosis.

Both sexes: Frontal depression with broadly interrupted transverse carinae; submedian carina sharp. Hyperoccipital carina indicated by rugae. Occipital carina convex medially. Postmarginal vein present. Coxa not darker than rest of leg. T1 lateral carina not expanded laterally. Metasomal flanges absent. Female only: A3 longer than pedicel. A4 longer than broad, A5 slightly longer than broad. Mesoscutellum without granulate sculpture. Metascutellum with irregular rugose sculpture. T1 midlobe carinae obscured by raised area. Fore wing long enough to reach base of T5. *Oxyscelio proceritatis* is one of the few Australian species with a strongly raised T1 horn in females. It differs from *Oxyscelio radii* and *Oxyscelio aciculae* in having a deeper frontal depression.

##### Etymology.

Latin noun, genitive case, meaning “length.”

##### Link to distribution map.

[http://hol.osu.edu/map-full.html?id=307099]

##### Material examined.

Holotype, female: AUSTRALIA: QLD, Mount Glorious, VII-1977 - XII-1977, malaise trap, A. Hiller, OSUC 368221 (deposited in QMBA). Paratypes: AUSTRALIA: 3 females, OSUC 227567, 462572 (CNCI); OSUC 437002 (WINC).

#### Oxyscelio productionis

Burks sp. n.

http://zoobank.org/F0A75A1A-2479-48B7-869F-4BFDA1F492C7

urn:lsid:biosci.ohio-state.edu:osuc_concepts:307100

http://species-id.net/wiki/Oxyscelio_productionis

**Figures 267–270.**
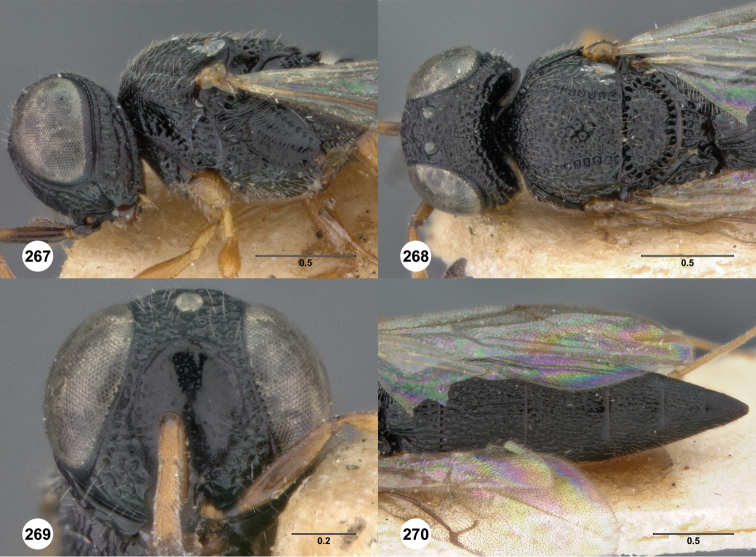
*Oxyscelio productionis* sp. n., holotype female (ANIC DB 32-020073) **267** Head and mesosoma, lateral view **268** Head and mesosoma, dorsal view **269** Head, anterior view **270** Metasoma, dorsal view. Morphbank^79^

Figures 267–270 ; Morphbank^79^

##### Description.

Female. Body length 3.95 mm (n=1).

Radicle color and shade: same as scape, both dark brown. Pedicel color: same as scape. A3: longer than pedicel. A4: longer than broad. A5: longer than broad.

Ventral clypeal margin: concave. Interantennal process: not elongate. Lower frons at dorsal margin of interantennal process: without transverse carina. Transverse curved rugae extending from frontal depression to eye: absent. Median longitudinal carina in frontal depression: absent. Ventral portion of frontal depression: with medially interrupted transverse carinae. Dorsal portion of frontal depression: without transverse carinae. Submedian carina: present. Frontal depression dorsally: not hood-like, open dorsally. Upper frons major sculpture: umbilicate foveate; irregularly rugose. Upper frons microsculpture: absent. Hyperoccipital carina: indicated by a set of irregular elevations. Carina connecting occipital carina to hyperoccipital carina: absent. Occipital carina: present laterally, absent medially. Occiput sculpture: umbilicate foveate. Extra carina ventral to occipital carina: absent. Gena length: shorter than eye. Major sculpture of gena anteroventrally: umbilicate foveate. Major sculpture of gena posteroventrally: umbilicate foveate; rugose; umbilicate punctate. Microsculpture of gena anteroventrally: absent. Microsculpture of gena posteroventrally: granulate.

Lateral pronotal area sculpture: anteriorly smooth, posterodorsal corner with dense microsculpture, ventral corner with irregular carinae. Posterior border of central pronotal area: directed anteriorly, protruding at corner of epomial carina and transverse pronotal carina. Mesoscutum anteriorly: not steep, forming less than a right angle. Major sculpture of mesoscutal midlobe anteriorly: umbilicate foveate. Mesoscutal midlobe sculpture at midlength: not different from nearby sculpture. Major sculpture of mesoscutal midlobe posteriorly: umbilicate foveate; obliquely rugose. Microsculpture of mesoscutal midlobe anteriorly: granulate. Microsculpture of mesoscutal midlobe posteriorly: absent; granulate. Median mesoscutal carina: present as a vague, occasionally interrupted elevation. Major sculpture of mesoscutellum centrally: umbilicate foveate. Major sculpture of mesoscutellum peripherally: umbilicate foveate. Microsculpture of mesoscutellum centrally: absent. Microsculpture of mesoscutellum peripherally: absent. Mesoscutellar rim: not expanded. Mesoscutellar rim medially: without notch. Mesofemoral depression: longitudinally striate dorsally and ventrally. Metascutellum shape: not emarginate, forming a flat, concave shelf. Metascutellar setae: absent. Metascutellum sculpture: with three parallel and evenly spaced longitudinal carinae. Postmarginal vein: present. Fore wing apex at rest: reaching near apex of T5. Coxae color brightness: same color as femora. Spines along tibiae: absent. Lateral propodeal carinae: broadly separated, not parallel anteriorly. Setae in metasomal depression: absent. Anterior sculpture of metasomal depression: absent. Median propodeal carina: absent.

T1 horn: present. Number of longitudinal carinae of T1 midlobe: obscured by other raised sculpture. T1 lateral carina: straight. T2 sculpture: densely foveolate, longitudinal sculpture irregular. T2 sublateral longitudinal foveae: absent. T3 metasomal flanges: absent. T4 sculpture: longitudinally striate to rugose, setal pits spanning interspaces. T4 metasomal flanges: absent. T5 sculpture: longitudinally striate to rugose, setal pits spanning interspaces. T5 metasomal flanges: absent. T6: longer than broad. Major sculpture of T6: umbilicate punctate; longitudinally striate. Microsculpture of T6: absent. T6 medially: flat and tapering to a rounded apex, not separated from apical rim. T6 metasomal flanges: absent. T6 raised peripheral rim: absent. S4 sculpture: longitudinally striate or rugose, setal pits spanning interspaces. S5 sculpture: longitudinally striate to rugose, setal pits spanning interspaces. S5 median carina: present. S6 peripheral carina: absent. S6 apex in relation to T6: not exposed to dorsal view. S6 apex: rounded or acuminate.

*Male*. unknown.

##### Diagnosis.

Both sexes: Frontal depression shallow, transverse carinae weak and interrupted; submedian carina strong but irregular. Hyperoccipital carina indicated by rugae. Occipital carina becoming weak or absent medially. Postmarginal vein present. Coxa not darker than rest of leg. Metasomal depression smooth. T1 lateral carina not expanded laterally. Metasomal flanges absent. Female only: A3 longer than pedicel. A4 longer than broad, A5 as long or longer than broad. Mesoscutellum without granulate sculpture. Metascutellum with 3 longitudinal carinae and some weak sculpture underlying them. T1 midlobe carinae obscured by raised smooth area. Fore wing long enough to reach middle of T5. *Oxyscelio productionis* is unusual in having 3 longitudinal carinae on the metascutellum. Otherwise it is very similar to some other species of the *aciculae*-group. Some other species of *Oxyscelio* may have a set of rugae on the metascutellum, but not 3 straight carinae.

##### Etymology.

Latin noun, genitive case, meaning “an extension.”

##### Link to distribution map.

[http://hol.osu.edu/map-full.html?id=307100]

##### Material examined.

Holotype, female: AUSTRALIA: QLD, Mount Tamborine, XII-1925, A. P. Dodd, ANIC DB 32-020073 (deposited in ANIC).

#### Oxyscelio radii

Burks sp. n.

http://zoobank.org/78AF0228-0097-4F90-89AB-B80796180375

urn:lsid:biosci.ohio-state.edu:osuc_concepts:307101

http://species-id.net/wiki/Oxyscelio_radii

**Figures 271–276.**
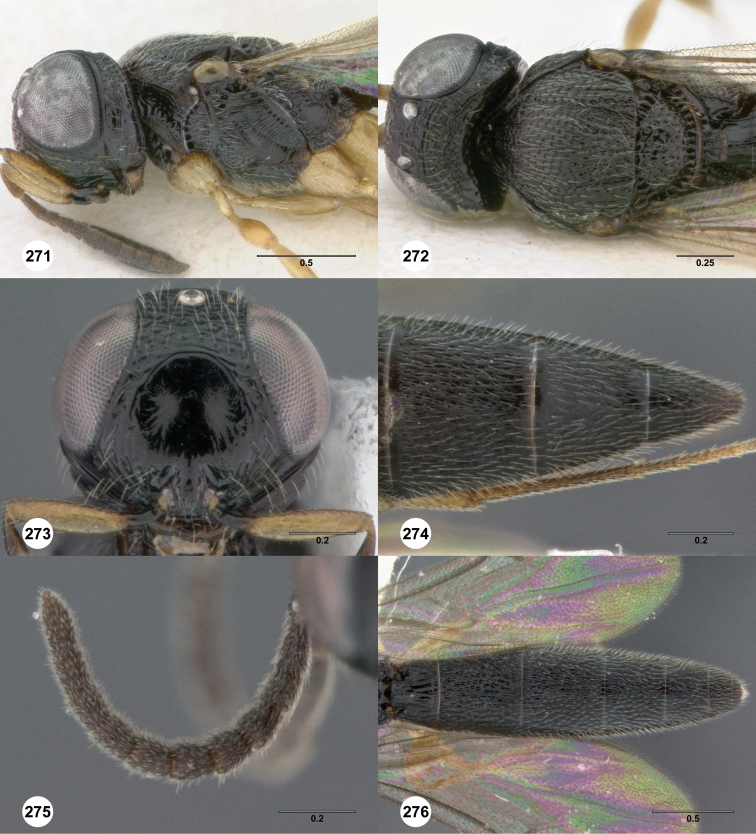
*Oxyscelio radii* sp. n., holotype female (OSUC 368185) **271** Head and mesosoma, lateral view **272** Head and mesosoma, dorsal view. Paratype female (OSUC 368178) **273** Head, anterior view **274** Metasoma, dorsal view. Paratype male (OSUC 368190) **275** Antenna **276** Metasoma, dorsal view. Morphbank^80^

Figures 271–276 ; Morphbank^80^

##### Description.

Female. Body length 4–4.65 mm (n=19).

Radicle color and shade: darker than scape. Pedicel color: at least partially darker than scape. A3: longer than pedicel. A4: broader than long; as long as broad. A5: broader than long.

Ventral clypeal margin: with slightly convex median lobe. Interantennal process: not elongate. Lower frons at dorsal margin of interantennal process: without transverse carina. Transverse curved rugae extending from frontal depression to eye: absent. Median longitudinal carina in frontal depression: absent. Ventral portion of frontal depression: smooth. Dorsal portion of frontal depression: without transverse carinae. Submedian carina: present. Frontal depression dorsally: not hood-like, open dorsally. Upper frons major sculpture: umbilicate foveate; transversely rugose. Upper frons microsculpture: absent. Hyperoccipital carina: indicated by a set of irregular elevations. Carina connecting occipital carina to hyperoccipital carina: present. Occipital carina: present laterally, absent medially. Occiput sculpture: irregularly sculptured. Extra carina ventral to occipital carina: absent. Gena length: shorter than eye. Major sculpture of gena anteroventrally: umbilicate foveate; rugose; umbilicate punctate. Major sculpture of gena posteroventrally: rugose; umbilicate punctate. Microsculpture of gena anteroventrally: absent. Microsculpture of gena posteroventrally: absent.

Lateral pronotal area sculpture: anteriorly smooth, posterodorsal corner with dense microsculpture, ventral corner with irregular carinae. Posterior border of central pronotal area: directed posteriorly, epomial carina absent or meeting transverse pronotal carina at arch on lateral surface of pronotum. Mesoscutum anteriorly: not steep, forming less than a right angle. Major sculpture of mesoscutal midlobe anteriorly: umbilicate foveate. Mesoscutal midlobe sculpture at midlength: not different from nearby sculpture. Major sculpture of mesoscutal midlobe posteriorly: umbilicate foveate; longitudinally rugose. Microsculpture of mesoscutal midlobe anteriorly: granulate. Microsculpture of mesoscutal midlobe posteriorly: absent. Median mesoscutal carina: present as a ruga. Major sculpture of mesoscutellum centrally: umbilicate foveate. Major sculpture of mesoscutellum peripherally: umbilicate foveate. Microsculpture of mesoscutellum centrally: absent. Microsculpture of mesoscutellum peripherally: absent. Mesoscutellar rim: not expanded. Mesoscutellar rim medially: without notch. Mesofemoral depression: longitudinally striate dorsally and ventrally. Metascutellum shape: not emarginate, forming a flat, concave shelf. Metascutellar setae: absent. Metascutellum sculpture: with a longitudinal median ruga and some weak transverse carinae. Postmarginal vein: present. Fore wing apex at rest: reaching middle of T5. Coxae color brightness: same color as femora. Spines along tibiae: absent. Lateral propodeal carinae: broadly separated, not parallel anteriorly. Setae in metasomal depression: absent. Anterior sculpture of metasomal depression: absent. Median propodeal carina: absent.

T1 horn: present. Number of longitudinal carinae of T1 midlobe: obscured by other raised sculpture. T1 lateral carina: straight. T2 sculpture: densely foveolate, longitudinal sculpture irregular. T2 sublateral longitudinal foveae: absent. T3 metasomal flanges: absent. T4 sculpture: longitudinally striate to rugose, setal pits spanning interspaces. T4 metasomal flanges: absent. T5 sculpture: longitudinally striate to rugose, setal pits spanning interspaces. T5 metasomal flanges: absent. T6: longer than broad. Major sculpture of T6: umbilicate punctate; longitudinally striate. Microsculpture of T6: absent. T6 medially: flat and tapering to a rounded apex, not separated from apical rim. T6 metasomal flanges: absent. T6 raised peripheral rim: absent. S4 sculpture: longitudinally striate or rugose, setal pits spanning interspaces. S5 sculpture: longitudinally striate to rugose, setal pits spanning interspaces. S5 median carina: present. S6 peripheral carina: absent. S6 apex in relation to T6: not exposed to dorsal view. S6 apex: rounded or acuminate.

*Male*. Body length 3.5–4.1 mm (n=14). A3: longer than pedicel. A5 tyloid shape: narrow, linear. A6: broader than long. A11: longer than broad. Major sculpture of mesoscutal midlobe anteriorly: umbilicate foveate. Major sculpture of mesoscutal midlobe posteriorly: umbilicate foveate; longitudinally rugose. Microsculpture of mesoscutal midlobe anteriorly: granulate. Microsculpture of mesoscutal midlobe posteriorly: absent. Major sculpture of mesoscutellum centrally: umbilicate foveate; irregularly rugose. Major sculpture of mesoscutellum peripherally: umbilicate foveate. Microsculpture of mesoscutellum centrally: absent. Microsculpture of mesoscutellum peripherally: absent. Fore wing apex at rest: reaching apex of T5. T1 midlobe longitudinal carinae: 3. T3 metasomal flanges: absent. T4 metasomal flanges: absent. T5 metasomal flanges: absent. T6 metasomal flanges: absent. T7: truncate.

##### Diagnosis.

Both sexes: Frontal depression flat, with oblique interrupted carinae and sometimes with an incomplete longitudinal carina; submedian carina weak. Hyperoccipital carina indicated by sharp rugae. Occipital carina connected to hyperoccipital carina by a weak longitudinal carina or irregular ruga, laterally with strong corners and medially sinuate; area between occipital and hyperoccipital carinae densely sculptured. Mesoscutellum densely foveolate. Metascutellum flat, rugose. Postmarginal vein present. Coxa not darker than rest of leg. T1 lateral carina not expanded laterally. Metasomal flanges absent. Female: A3 not longer than pedicel. A4 slightly longer than broad, A5 broader than long. T1 with raised horn obscuring longitudinal carinae. Fore wing long enough to reach middle of T5 or base of T6. T6 slightly longer than broad. Male: A4 slightly longer than broad, A11 longer than broad. T1 midlobe with 3 longitudinal carinae. Fore wing not long enough to exceed metasomal apex. T7 truncate or slightly emarginate apically. *Oxyscelio radii* is very similar to *Oxyscelio aciculae*. These two species are best distinguished using metasomal length in both males and females.

##### Etymology.

Latin noun, genitive case, meaning “rod.”

##### Link to distribution map.

[http://hol.osu.edu/map-full.html?id=307101]

##### Material examined.

Holotype, female: AUSTRALIA: QLD, via Mount Carbine, Mount Windsor Tableland, 28.I–5.III.1981, malaise trap, OSUC 368185 (deposited in QMBA). Paratypes: AUSTRALIA: 20 females, 14 males, Australian Museum K256238 (AMSA); OSUC 368176, 368178-368184, 368186-368189 (ANIC); OSUC 448966 (BMNH); OSUC 462582, 462585-462586 (CNCI); OSUC 368177, QDPC 0-165630, QDPC 0-165750, QDPC 0-165784, QDPC 0-165790 (QDPC); OSUC 148478, 368191 (QMBA); OSUC 448601 (QPIM); OSUC 448955, 448957 (UQIC); OSUC 368175, 368190, 368192-368194, 448949-448950 (WINC).

##### Comments.

Distinction between *Oxyscelio radii* and *Oxyscelio aciculae* is very difficult in some cases. These may represent a single species with strong variation due to differently-shaped hosts, which could be tested with further research. However, at present there is some justification in maintaining them as separate taxa.

#### Oxyscelio rami

Burks sp. n.

http://zoobank.org/E521A5E7-684A-4766-9C72-07B5C52D2C01

urn:lsid:biosci.ohio-state.edu:osuc_concepts:307102

http://species-id.net/wiki/Oxyscelio_rami

**Figures 277–282.**
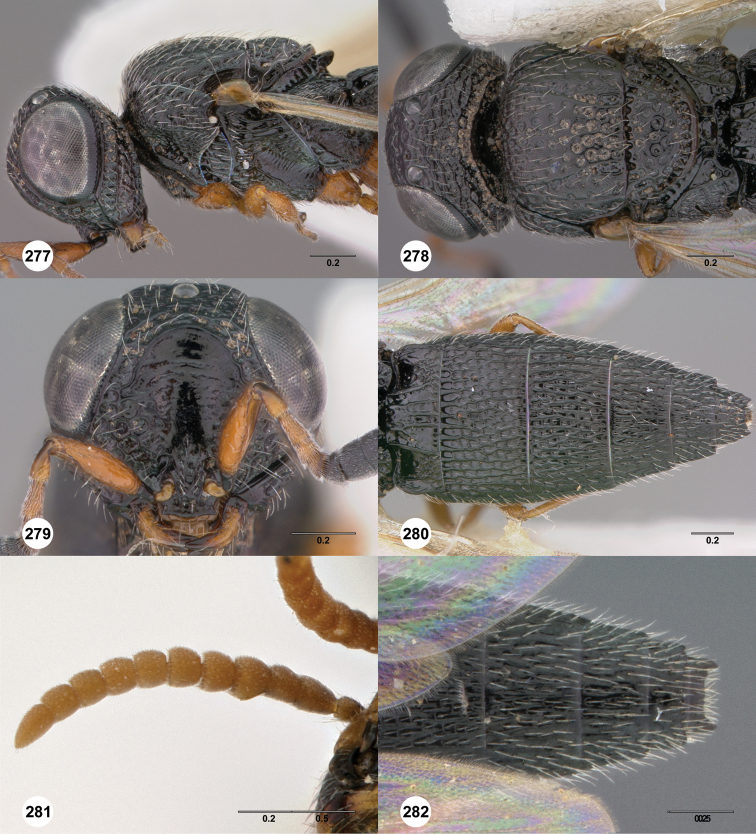
*Oxyscelio rami* sp. n., holotype female (OSUC 148356) **277** Head and mesosoma, lateral view **278** Head and mesosoma, dorsal view **279** Head, anterior view **280** Metasoma, dorsal view. Paratype male (OSUC 448602) **281** Antenna **282** Metasoma, dorsal view. Morphbank^81^

Figures 277–282 ; Morphbank^81^

##### Description.

Female. Body length 2.8–3.85 mm (n=12).

Radicle color and shade: darker than scape. Pedicel color: same as scape; at least partially darker than scape. A3: as long as pedicel. A4: broader than long. A5: broader than long.

Ventral clypeal margin: with slightly convex median lobe. Interantennal process: not elongate. Lower frons at dorsal margin of interantennal process: without transverse carina. Transverse curved rugae extending from frontal depression to eye: absent. Median longitudinal carina in frontal depression: absent. Ventral portion of frontal depression: with medially interrupted transverse foveae. Dorsal portion of frontal depression: with some transverse carinae. Submedian carina: present. Frontal depression dorsally: not hood-like, open dorsally. Upper frons major sculpture: umbilicate foveate; irregularly rugose. Upper frons microsculpture: absent. Hyperoccipital carina: absent. Carina connecting occipital carina to hyperoccipital carina: absent. Occipital carina: broadly angular, with rounded median peak. Occiput sculpture: umbilicate foveate. Extra carina ventral to occipital carina: present, complete. Gena length: shorter than eye. Major sculpture of gena anteroventrally: umbilicate foveate. Major sculpture of gena posteroventrally: umbilicate foveate; rugose. Microsculpture of gena anteroventrally: absent. Microsculpture of gena posteroventrally: absent.

Lateral pronotal area sculpture: with shallow irregular carinae, posterodorsal corner with dense microsculpture. Posterior border of central pronotal area: directed anteriorly, protruding at corner of epomial carina and transverse pronotal carina. Mesoscutum anteriorly: very steep and tall, descending at a right angle or protruding anteriorly. Major sculpture of mesoscutal midlobe anteriorly: umbilicate foveate. Mesoscutal midlobe sculpture at midlength: not different from nearby sculpture. Major sculpture of mesoscutal midlobe posteriorly: umbilicate foveate. Microsculpture of mesoscutal midlobe anteriorly: granulate. Microsculpture of mesoscutal midlobe posteriorly: absent. Median mesoscutal carina: present as a vague, occasionally interrupted elevation. Major sculpture of mesoscutellum centrally: umbilicate foveate. Major sculpture of mesoscutellum peripherally: umbilicate foveate. Microsculpture of mesoscutellum centrally: absent; punctate. Microsculpture of mesoscutellum peripherally: absent; punctate. Mesoscutellar rim: not expanded. Mesoscutellar rim medially: without notch. Mesofemoral depression: longitudinally striate dorsally, smooth ventrally. Metascutellum shape: not emarginate, concave but elevated posteriorly. Metascutellar setae: absent. Metascutellum sculpture: with large smooth posterior fovea. Postmarginal vein: absent. Fore wing apex at rest: reaching base of T5. Coxae color brightness: same color as femora. Spines along tibiae: absent. Lateral propodeal carinae: broadly separated, not parallel anteriorly. Setae in metasomal depression: absent. Anterior sculpture of metasomal depression: absent. Median propodeal carina: absent.

T1 horn: absent. Number of longitudinal carinae of T1 midlobe: 4. T1 lateral carina: protruding laterally, visible from ventral view. T2 sculpture: with longitudinal striae or rugae, setiferous puncta present between them. T2 sublateral longitudinal foveae: absent. T3 metasomal flanges: absent. T4 sculpture: longitudinally striate to rugose, setal pits spanning interspaces. T4 metasomal flanges: present as slightly protruding sharp corners. T5 sculpture: longitudinally striate to rugose, setal pits spanning interspaces. T5 metasomal flanges: present as slightly protruding sharp corners. T6: broader than long. Major sculpture of T6: umbilicate foveate. Microsculpture of T6: absent. T6 medially: with medially truncate emargination, sloping down to apical rim. T6 metasomal flanges: present as spine-like structures posterolaterally. T6 raised peripheral rim: absent. S4 sculpture: longitudinally striate or rugose, setal pits spanning interspaces. S5 sculpture: longitudinally striate to rugose, setal pits spanning interspaces. S5 median carina: present. S6 peripheral carina: absent. S6 apex in relation to T6: exposed to dorsal view by T6 emargination. S6 apex: rounded or acuminate.

*Male*. Body length 3.2–4 mm (n=3). A3: longer than pedicel. A5 tyloid shape: narrow, linear. A6: broader than long. A11: broader than long. Major sculpture of mesoscutal midlobe anteriorly: umbilicate foveate. Major sculpture of mesoscutal midlobe posteriorly: umbilicate foveate. Microsculpture of mesoscutal midlobe anteriorly: granulate. Microsculpture of mesoscutal midlobe posteriorly: absent. Major sculpture of mesoscutellum centrally: umbilicate foveate. Major sculpture of mesoscutellum peripherally: umbilicate foveate. Microsculpture of mesoscutellum centrally: absent. Microsculpture of mesoscutellum peripherally: absent. Fore wing apex at rest: reaching middle of T5. T1 midlobe longitudinal carinae: 4. T3 metasomal flanges: absent. T4 metasomal flanges: absent. T5 metasomal flanges: present as sharp corners that do not protrude. T6 metasomal flanges: present as sharp corners that do not protrude. T7: with a pair of sharply defined spine-like posterolateral projections.

##### Diagnosis.

Both sexes: Frontal depression deep, with interrupted carinae ventrally, dorsal separator complete medially, carinae present above dorsal separator; submedian carina distinct but only weakly protruding. Hyperoccipital carina indicated by vague rugae. Occipital carina complete, medially sinuate. Mesoscutellar rim not expanded, without median notch. Metascutellum small, weakly emarginate apically, projecting dorsally. Coxa same color as rest of leg. Postmarginal vein absent. Female: A4, A5 broader than long. T4, T5 with sharp posterior corners. T6 abruptly narrower than T5, without expanded lateral margins but with narrow and sharp elongate posterior metasomal flanges. Main surface of T6 strongly emarginate medially, sharply raised above apical rim. S6 not exposed to dorsal view. Male: A4, A11 broader than long. Mesoscutellum and mesoscutal midlobe posteriorly with small and densely set foveae, mesoscutellum without smooth area medially. T5, T6 with sharp posterior corners; T6, T7 abruptly narrower than preceding tergum. T7 with narrow and sharp postero-lateral lobes, deeply emarginate, truncate medially. *Oxyscelio rami* differs from *Oxyscelio caudarum* and some other species in its stronger frontal depression sculpture and in a slightly different metascutellum and T6 apex.

##### Etymology.

Latin noun, genitive case, meaning “branch” or “prong.”

##### Link to distribution map.

[http://hol.osu.edu/map-full.html?id=307102]

##### Associations.

Collected on *Eucalyptus* L’Hér.: [Myrtales: Myrtaceae]

##### Material examined.

Holotype, female: AUSTRALIA: NE QLD, Tea Tree Cave, 4km SE Chillagoe, 17°11'S, 144°34'E, 25.IV.1997, C. J. Burwell, OSUC 148356 (deposited in QMBA). Paratypes: AUSTRALIA: 11 females, 3 males, OSUC 437864-437865, 437867, 437871 (ANIC); OSUC 148381, 148483, 437866, 437870 (QMBA); OSUC 448602 (QPIM); OSUC 268211-268212 (USNM); OSUC 437868-437869, 437872 (WINC).

#### Oxyscelio rugulosus

(Dodd)

http://zoobank.org/7662C30F-0110-481C-8C4E-A7BFDA3D3EE7

urn:lsid:biosci.ohio-state.edu:osuc_concepts:5033

http://species-id.net/wiki/Oxyscelio_rugulosus

**Figures 283–288.**
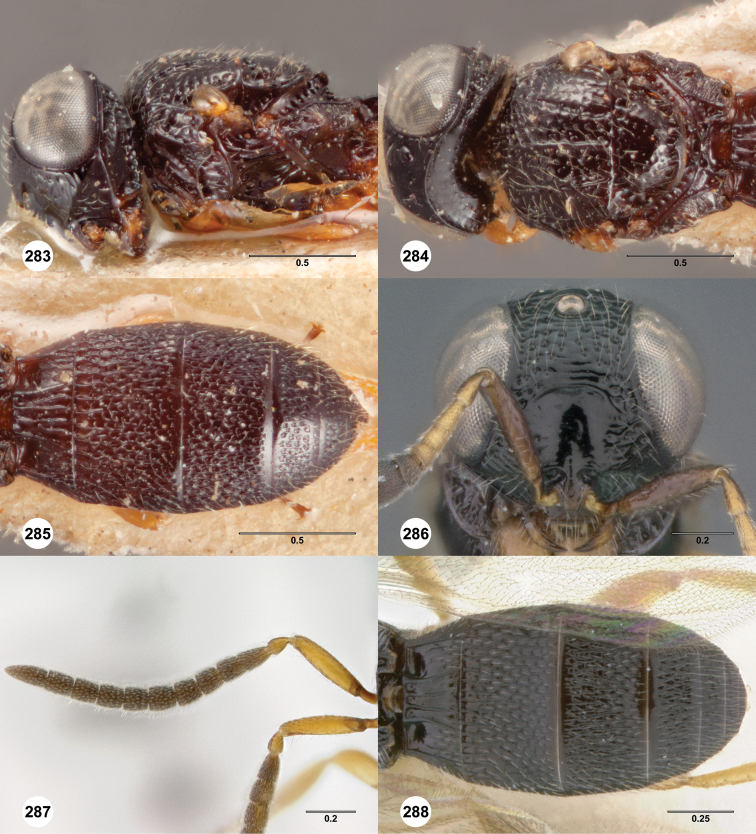
*Oxyscelio rugulosus* (Dodd), syntype female (SAMA DB 32-001590) **283** Head and mesosoma, lateral view **284** Head and mesosoma, dorsal view **285** Metasoma, dorsal view. Female (OSUC 442410) **286** Head, anterior view. Male (OSUC 442459) **287** Antenna **288** Metasoma, dorsal view. Morphbank^82^

Figures 283–288 ; Morphbank^82^

Sceliomorpha rugulosa Dodd, 1913: 139 (original description); Kieffer 1926: 302, 306 (description, keyed).
Dicroteleia glabriscutellum Dodd, 1914: 106 (original description); Kieffer 1926: 387, 390 (description, keyed). **syn. n.**
Dicroteleia rugulosa (Dodd): Dodd 1914: 107 (generic transfer).
Oxyscelio rugulosus (Dodd): Dodd 1931: 76 (generic transfer); Galloway 1976: 100 (type information); Masner 1976: 24 (description).

##### Description.

Female. Body length 2.55–3 mm (n=20).

Radicle color and shade: same as scape, both yellowish or reddish. Pedicel color: same as scape. A3: shorter than pedicel; as long as pedicel. A4: broader than long. A5: broader than long.

Ventral clypeal margin: concave. Interantennal process: not elongate. Lower frons at dorsal margin of interantennal process: with transverse ledge, face sharply receding below it. Transverse curved rugae extending from frontal depression to eye: present. Median longitudinal carina in frontal depression: absent. Ventral portion of frontal depression: with transverse carinae. Dorsal portion of frontal depression: without transverse carinae. Submedian carina: present only as a weak shift in elevation. Frontal depression dorsally: not hood-like, open dorsally. Upper frons major sculpture: umbilicate foveate; transversely rugose. Upper frons microsculpture: absent. Hyperoccipital carina: present as a single carina. Carina connecting occipital carina to hyperoccipital carina: absent. Occipital carina: present laterally, absent medially. Occiput sculpture: smooth. Extra carina ventral to occipital carina: present, complete. Gena length: shorter than eye. Major sculpture of gena anteroventrally: umbilicate foveate; umbilicate punctate. Major sculpture of gena posteroventrally: umbilicate foveate; umbilicate punctate. Microsculpture of gena anteroventrally: absent. Microsculpture of gena posteroventrally: absent.

Lateral pronotal area sculpture: smooth anteriorly, densely setose posteriorly. Posterior border of central pronotal area: directed posteriorly, epomial carina absent or meeting transverse pronotal carina at arch on lateral surface of pronotum. Mesoscutum anteriorly: very steep and tall, descending at a right angle or protruding anteriorly. Major sculpture of mesoscutal midlobe anteriorly: umbilicate foveate; transversely rugose. Mesoscutal midlobe sculpture at midlength: not different from nearby sculpture. Major sculpture of mesoscutal midlobe posteriorly: umbilicate foveate. Microsculpture of mesoscutal midlobe anteriorly: absent. Microsculpture of mesoscutal midlobe posteriorly: absent. Median mesoscutal carina: present as a vague, occasionally interrupted elevation. Major sculpture of mesoscutellum centrally: umbilicate punctate. Major sculpture of mesoscutellum peripherally: umbilicate foveate; umbilicate punctate. Microsculpture of mesoscutellum centrally: absent. Microsculpture of mesoscutellum peripherally: absent. Mesoscutellar rim: not expanded. Mesoscutellar rim medially: without notch. Mesofemoral depression: longitudinally striate dorsally, smooth ventrally. Metascutellum shape: slightly emarginate posteriorly, concave but elevated posteriorly. Metascutellar setae: absent. Metascutellum sculpture: with large smooth posterior fovea. Spines along tibiae: absent. Lateral propodeal carinae: narrowly separated, angled anteriorly to become parallel. Setae in metasomal depression: absent. Anterior sculpture of metasomal depression: with median areole or pair of pits. Median propodeal carina: present. Postmarginal vein: absent. Fore wing apex at rest: exceeding metasomal apex. Coxae color brightness: same color as femora.

T1 horn: absent. Number of longitudinal carinae of T1 midlobe: 4. T1 lateral carina: straight. T2 sculpture: with longitudinal striae or rugae, setiferous puncta present between them. T2 sublateral longitudinal foveae: absent. T3 metasomal flanges: absent. T4 sculpture: longitudinally striate to rugose, setal pits spanning interspaces. T4 metasomal flanges: absent. T5 sculpture: longitudinally striate to rugose, setal pits spanning interspaces. T5 metasomal flanges: absent. T6: broader than long. Major sculpture of T6: umbilicate punctate. Microsculpture of T6: absent. T6 medially: slightly emarginate, not separated from apical rim. T6 metasomal flanges: absent. T6 raised peripheral rim: absent. S4 sculpture: densely setose, setal pits between very weak longitudinal rugae. S5 sculpture: densely setose, setal pits between very weak longitudinal rugae. S5 median carina: absent. S6 peripheral carina: absent. S6 apex in relation to T6: not exposed to dorsal view. S6 apex: rounded or acuminate.

*Male*. Body length 2.5–3 mm (n=20). A3: longer than pedicel. A5 tyloid shape: narrow, linear. A6: longer than broad. A11: longer than broad. Major sculpture of mesoscutal midlobe anteriorly: umbilicate foveate; transversely rugose. Major sculpture of mesoscutal midlobe posteriorly: umbilicate foveate. Microsculpture of mesoscutal midlobe anteriorly: absent. Microsculpture of mesoscutal midlobe posteriorly: absent. Major sculpture of mesoscutellum centrally: umbilicate foveate; umbilicate punctate. Major sculpture of mesoscutellum peripherally: umbilicate foveate. Microsculpture of mesoscutellum centrally: absent. Microsculpture of mesoscutellum peripherally: absent. Fore wing apex at rest: exceeding metasomal apex. T1 midlobe longitudinal carinae: 4. T3 metasomal flanges: absent. T4 metasomal flanges: absent. T5 metasomal flanges: absent. T6 metasomal flanges: absent. T7: truncate.

##### Diagnosis.

Both sexes: Frontal depression shallow, weak transverse carinae present; submedian carina indicated by a set of weak rugae, flat or only weakly rounded dorsally. Hyperoccipital carina sharp and strong. Occipital carina incomplete, lateral portions nearly reaching hyperoccipital carina. Occiput almost entirely smooth, with a row of setiferous puncta. Mesoscutum with median carina. Metascutellum broad and concave, truncate or slightly concave apically, projecting dorsally. Postmarginal vein present. Coxa not darker than rest of leg. T1 lateral carina not expanded laterally. Metasomal flanges absent. Female: A3 as long or longer than pedicel. A4, A5 broader than long. Mesoscutellum weakly sculptured, with only setiferous puncta. T1 midlobe with 4 longitudinal carinae. Fore wing long enough to reach beyond T6. T6 broader than long. Male: All flagellomeres past A3 about as long as broad. Frontal depression without complete transverse carinae. T1 midlobe with 4 longitudinal carinae. Fore wing long enough to reach beyond metasomal apex. T7 tiny, truncate.

##### Link to distribution map.

[http://hol.osu.edu/map-full.html?id=5033]

##### Material examined.

Syntype, male, S. rugulosa: AUSTRALIA: QLD, nr. Cairns, jungle edge, Gordonvale (Nelson), no date, sweeping, A. P. Dodd, SAMA DB 32-001589 (deposited in SAMA). Syntype, female, S. rugulosa: Female holotype of, D. glabriscutellum: AUSTRALIA: QLD, nr. Cairns, plant foliage / jungle country, Harvey Creek, 12.X.1914, A. P. Dodd, SAMA DB 32-001585 (deposited in SAMA). AUSTRALIA: QLD, jungle edge, nr. Gordonvale (Nelson), no date, sweeping, A. P. Dodd, SAMA DB 32-001590 (deposited in SAMA). Paratype: AUSTRALIA: 1 female, ANIC DB 32-020109 (ANIC). Other material: AUSTRALIA: 49 females, 68 males, 442441b, ANIC DB 32-020104, ANIC DB 32-020105, ANIC DB 32-020106, ANIC DB 32-020107, ANIC DB 32-020108, OSUC 442402, OSUC 442403, OSUC 442404, OSUC 442405, OSUC 442406, OSUC 442407, OSUC 442408, OSUC 442409, OSUC 442413, OSUC 442414, OSUC 442415, OSUC 442416, OSUC 442417, OSUC 442418, OSUC 442419, OSUC 442420, OSUC 442421, OSUC 442422, OSUC 442423, OSUC 442424, OSUC 442425, OSUC 442426, OSUC 442427, OSUC 442428, OSUC 442429, OSUC 442430, OSUC 442431, OSUC 442432, OSUC 442433, OSUC 442434, OSUC 442435, OSUC 442436, OSUC 442438, OSUC 442439, OSUC 442440, OSUC 442442, OSUC 442443, OSUC 442444, OSUC 442445, OSUC 442446, OSUC 442448, OSUC 442449, OSUC 442450, OSUC 442451, OSUC 442452, OSUC 442453, OSUC 442454, OSUC 442455, OSUC 442456, OSUC 442457, OSUC 442458, OSUC 442459, OSUC 442460, OSUC 442461, OSUC 442462, OSUC 442463, OSUC 442464, OSUC 442465, OSUC 442466, OSUC 442467, OSUC 442468, OSUC 442469, OSUC 442470, OSUC 442471, OSUC 442473, OSUC 442474, OSUC 442475, OSUC 442477, OSUC 442478, OSUC 442479, OSUC 442480, OSUC 442481, OSUC 442482, OSUC 442484, OSUC 442488, OSUC 451299, OSUC 451300, OSUC 451302 (ANIC); OSUC 451301, 451303, 451305 (BMNH); OSUC 324331, 58660 (OSUC); OSUC 451304, QDPC 0-165688, QDPC 0-165691, QDPC 0-165694, QDPC 0-165701, QDPC 0-165703, QDPC 0-165713, QDPC 0-165724, QDPC 0-165731, QDPC 0-165735, QDPC 0-165737, QDPC 0-165745, QDPC 0-165749 (QDPC); OSUC 442437, 442485-442486 (QMBA); OSUC 448597, 448600 (QPIM); UCRC ENT 151376-151377 (UCRC); OSUC 442410-442412, 442447, 442472, 442476, 442483, 442487 (WINC).

##### Comments.

*Oxyscelio rugulosus* differs from most members of the *flavipes*-group in having a median mesoscutal carina. This is accompanied by weak and sparse mesoscutal sculpture, which is also unusual in the *flavipes*-group. The type specimen of *Oxyscelio glabriscutellum* does not at all differ from our concept of *Oxyscelio rugulosus*.

#### Oxyscelio rupturae

Burks sp. n.

http://zoobank.org/9A49C5D5-7AEA-48A7-81E3-13F804160DC4

urn:lsid:biosci.ohio-state.edu:osuc_concepts:307103

http://species-id.net/wiki/Oxyscelio_rupturae

**Figures 289–294.**
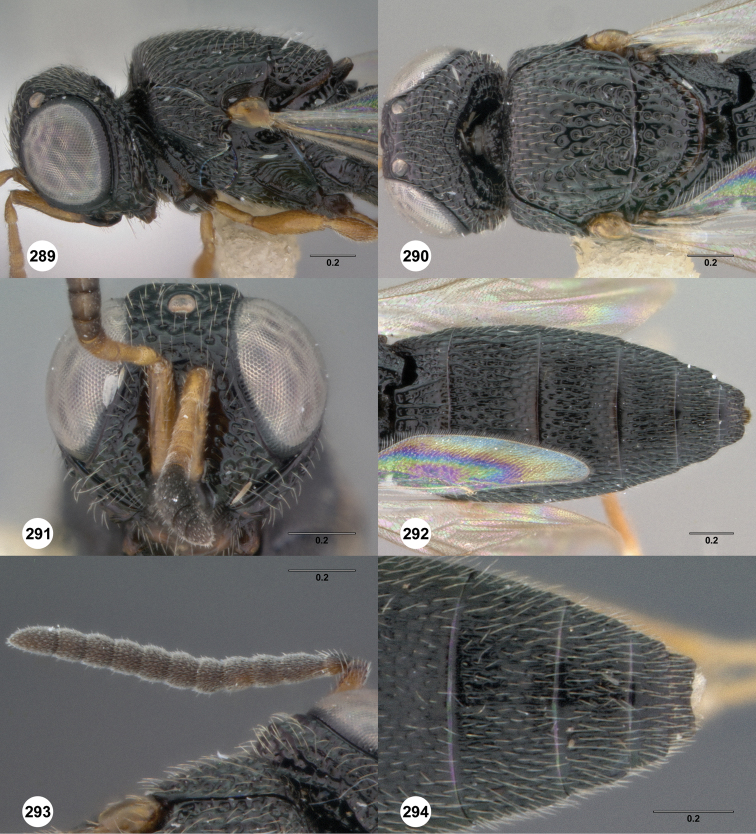
*Oxyscelio rupturae* sp. n., holotype female (OSUC 268197) **289** Head and mesosoma, lateral view **290** Head and mesosoma, dorsal view **291** Head, anterior view **292** Metasoma, dorsal view. Paratype male (OSUC 268190) **293** Antenna **294** Metasoma, dorsal view. Morphbank^83^

Figures 289–294 ; Morphbank^83^

##### Description.

Female. Body length 3.25–3.65 mm (n=4).

Radicle color and shade: darker than scape. Pedicel color: same as scape. A3: longer than pedicel. A4: longer than broad. A5: broader than long.

Ventral clypeal margin: with slightly convex median lobe. Interantennal process: not elongate. Lower frons at dorsal margin of interantennal process: without transverse carina. Transverse curved rugae extending from frontal depression to eye: absent. Median longitudinal carina in frontal depression: absent. Ventral portion of frontal depression: smooth. Dorsal portion of frontal depression: with some transverse carinae. Submedian carina: present. Frontal depression dorsally: not hood-like, open dorsally. Upper frons major sculpture: umbilicate foveate. Upper frons microsculpture: absent. Hyperoccipital carina: indicated by a set of irregular elevations. Carina connecting occipital carina to hyperoccipital carina: absent. Occipital carina: omicron-shaped, with sharp corners where median portion meets lateral portions. Occiput sculpture: irregularly sculptured. Extra carina ventral to occipital carina: present, medially incomplete. Gena length: shorter than eye. Major sculpture of gena anteroventrally: umbilicate foveate. Major sculpture of gena posteroventrally: umbilicate foveate. Microsculpture of gena anteroventrally: absent. Microsculpture of gena posteroventrally: absent.

Lateral pronotal area sculpture: anteriorly smooth, posterodorsal corner with dense microsculpture, ventral corner with irregular carinae. Posterior border of central pronotal area: directed anteriorly, protruding at corner of epomial carina and transverse pronotal carina. Mesoscutum anteriorly: very steep and tall, descending at a right angle or protruding anteriorly. Major sculpture of mesoscutal midlobe anteriorly: umbilicate foveate. Mesoscutal midlobe sculpture at midlength: not different from nearby sculpture. Major sculpture of mesoscutal midlobe posteriorly: umbilicate foveate; longitudinally rugose. Microsculpture of mesoscutal midlobe anteriorly: granulate. Microsculpture of mesoscutal midlobe posteriorly: absent. Median mesoscutal carina: present as a vague, occasionally interrupted elevation. Major sculpture of mesoscutellum centrally: umbilicate foveate; longitudinally rugose. Major sculpture of mesoscutellum peripherally: umbilicate foveate; longitudinally rugose. Microsculpture of mesoscutellum centrally: absent. Microsculpture of mesoscutellum peripherally: absent. Mesoscutellar rim: not expanded. Mesoscutellar rim medially: without notch. Mesofemoral depression: longitudinally striate dorsally, smooth ventrally. Metascutellum shape: slightly emarginate posteriorly, concave but elevated posteriorly. Metascutellar setae: absent. Metascutellum sculpture: with large smooth posterior fovea. Postmarginal vein: present. Fore wing apex at rest: reaching middle of T5. Coxae color brightness: darker than femora. Spines along tibiae: absent. Lateral propodeal carinae: broadly separated, not parallel anteriorly. Setae in metasomal depression: absent. Anterior sculpture of metasomal depression: absent. Median propodeal carina: absent.

T1 horn: absent. Number of longitudinal carinae of T1 midlobe: 4. T1 lateral carina: protruding laterally, visible from ventral view. T2 sculpture: with longitudinal striae or rugae, setiferous puncta present between them. T2 sublateral longitudinal foveae: absent. T3 metasomal flanges: absent. T4 sculpture: longitudinally striate to rugose, setal pits spanning interspaces. T4 metasomal flanges: absent. T5 sculpture: longitudinally striate to rugose, setal pits spanning interspaces. T5 metasomal flanges: present as strong posterior corners. T6: broader than long. Major sculpture of T6: umbilicate punctate. Microsculpture of T6: absent. T6 medially: with medially truncate emargination, sloping down to apical rim. T6 metasomal flanges: present as slightly expanded lateral rims, truncate posteriorly. T6 raised peripheral rim: absent. S4 sculpture: longitudinally striate or rugose, setal pits spanning interspaces. S5 sculpture: longitudinally striate to rugose, setal pits spanning interspaces. S5 median carina: present. S6 peripheral carina: absent. S6 apex in relation to T6: not exposed to dorsal view. S6 apex: rounded or acuminate.

*Male*. Body length 3.2–3.45 mm (n=6). A3: longer than pedicel. A5 tyloid shape: narrow, linear. A6: broader than long. A11: longer than broad. Major sculpture of mesoscutal midlobe anteriorly: umbilicate foveate. Major sculpture of mesoscutal midlobe posteriorly: umbilicate foveate. Microsculpture of mesoscutal midlobe anteriorly: granulate. Microsculpture of mesoscutal midlobe posteriorly: absent. Major sculpture of mesoscutellum centrally: umbilicate foveate. Major sculpture of mesoscutellum peripherally: umbilicate foveate. Microsculpture of mesoscutellum centrally: absent. Microsculpture of mesoscutellum peripherally: absent. Fore wing apex at rest: reaching middle of T6. T1 midlobe longitudinal carinae: 4. T3 metasomal flanges: absent. T4 metasomal flanges: absent. T5 metasomal flanges: absent. T6 metasomal flanges: present as subapical tubercles. T7: broadly and deeply emarginate, with rounded posterolateral margins.

##### Diagnosis.

Both sexes: Frontal depression deep, carinae interrupted medially, carinae present above dorsal separator; submedian carina absent or weak and irregular. Hyperoccipital carina indicated by rugae. Occipital carina complete, sinuate medially. Mesoscutellar rim not expanded. Metascutellum scoop-shaped, deeply concave, slightly emarginate apically, projecting dorsally. Coxa darker than rest of leg. T1 lateral carina expanded laterally. Female: A3 longer than pedicel. A4 longer than broad, A5, A6 broader than long. T1 midlobe with 4 longitudinal midlobe carinae anteriorly. Fore wing long enough to reach middle of T5. T4, T5 without metasomal flanges. Main body of T6 raised above apical rim and laterally abruptly separated from it, but medially not abruptly separated from it; lateral margins of T6 slightly expanded, forming a pair of lateral lobe-like shelves. Male: A3 longer than pedicel. A4 as broad or broader than long. A11 longer than broad. Mesoscutellum and mesoscutal midlobe posteriorly with small and densely set foveae, mesoscutellum without smooth area medially. T5, T6 without metasomal flanges. T7 with broad postero-lateral lobes, deeply emarginate medially, posterior margin roughly sculptured. *Oxyscelio rupturae* is very similar to *Oxyscelio truncationis*, differing in frontal depression sculpture, T6 shape, and mesosomal sculpture.

##### Etymology.

Latin noun, genitive case, meaning “breach.”

##### Link to distribution map.

[http://hol.osu.edu/map-full.html?id=307103]

##### Material examined.

Holotype, female: AUSTRALIA: QLD, Townsville, 10.XII–17.XII.1987, malaise trap, T. Goertemiller, OSUC 268197 (deposited in USNM). Paratypes: AUSTRALIA: 7 females, 2 males, OSUC 268190, 268196, 268198-268199, 268202, 268204-268206, 268216 (USNM).

#### Oxyscelio sarcinae

Burks sp. n.

http://zoobank.org/2C5D5799-5923-4998-B59E-1FB721818E57

urn:lsid:biosci.ohio-state.edu:osuc_concepts:307104

http://species-id.net/wiki/Oxyscelio_sarcinae

**Figures 295–300.**
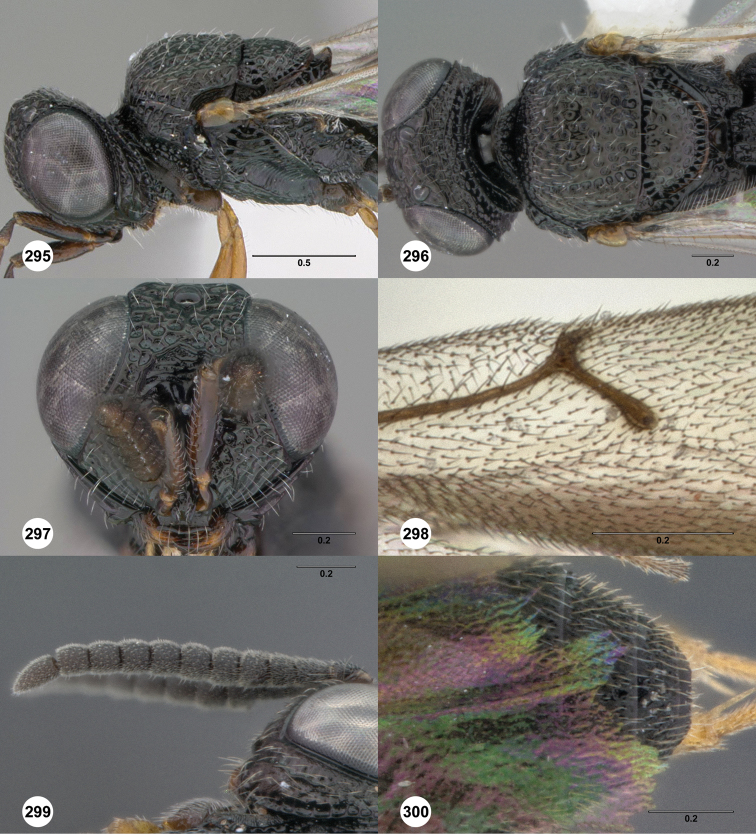
*Oxyscelio sarcinae* sp. n., holotype female (OSUC 439555) **295** Head and mesosoma, lateral view **296** Head and mesosoma, dorsal view **297** Head, anterior view **298** Fore wing venation. Paratype male (OSUC 439566) **299** Antenna **300** Metasoma, dorsal view. Morphbank^84^

Figures 295–300 ; Morphbank^84^

##### Description.

Female. Body length 3.25–3.5 mm (n=8).

Radicle color and shade: darker than scape; same as scape, both dark brown. Pedicel color: same as scape. A3: shorter than pedicel; as long as pedicel. A4: broader than long. A5: broader than long.

Ventral clypeal margin: with slightly convex median lobe. Interantennal process: not elongate. Lower frons at dorsal margin of interantennal process: without transverse carina. Transverse curved rugae extending from frontal depression to eye: absent. Median longitudinal carina in frontal depression: absent. Ventral portion of frontal depression: smooth. Dorsal portion of frontal depression: without transverse carinae. Submedian carina: absent. Frontal depression dorsally: not hood-like, open dorsally. Upper frons major sculpture: umbilicate foveate. Upper frons microsculpture: absent. Hyperoccipital carina: indicated by a set of irregular elevations. Carina connecting occipital carina to hyperoccipital carina: absent. Occipital carina: weakly arched dorsally, with rounded lateral corners. Occiput sculpture: irregularly sculptured. Extra carina ventral to occipital carina: present, complete. Gena length: shorter than eye. Major sculpture of gena anteroventrally: umbilicate foveate. Major sculpture of gena posteroventrally: umbilicate foveate; rugose. Microsculpture of gena anteroventrally: absent. Microsculpture of gena posteroventrally: granulate.

Lateral pronotal area sculpture: densely covered with setiferous puncta. Posterior border of central pronotal area: directed anteriorly, protruding at corner of epomial carina and transverse pronotal carina. Mesoscutum anteriorly: very steep and tall, descending at a right angle or protruding anteriorly. Major sculpture of mesoscutal midlobe anteriorly: umbilicate foveate. Mesoscutal midlobe sculpture at midlength: not different from nearby sculpture. Major sculpture of mesoscutal midlobe posteriorly: umbilicate foveate. Microsculpture of mesoscutal midlobe anteriorly: granulate. Microsculpture of mesoscutal midlobe posteriorly: absent; granulate. Median mesoscutal carina: present as a ruga. Major sculpture of mesoscutellum centrally: umbilicate foveate. Major sculpture of mesoscutellum peripherally: umbilicate foveate. Microsculpture of mesoscutellum centrally: punctate. Microsculpture of mesoscutellum peripherally: punctate. Mesoscutellar rim: not expanded. Mesoscutellar rim medially: without notch. Mesofemoral depression: longitudinally striate dorsally, smooth ventrally. Metascutellum shape: slightly emarginate posteriorly, concave but elevated posteriorly. Metascutellar setae: absent. Metascutellum sculpture: with large smooth posterior fovea. Postmarginal vein: present. Fore wing apex at rest: exceeding metasomal apex. Coxae color brightness: darker than femora. Spines along tibiae: absent. Lateral propodeal carinae: broadly separated, but parallel for a short distance anteriorly. Setae in metasomal depression: absent. Anterior sculpture of metasomal depression: absent. Median propodeal carina: absent.

T1 horn: absent. Number of longitudinal carinae of T1 midlobe: 4; obscured by other raised sculpture. T1 lateral carina: protruding laterally, visible from ventral view. T2 sculpture: with longitudinal striae or rugae, setiferous puncta present between them. T2 sublateral longitudinal foveae: absent. T3 metasomal flanges: absent. T4 sculpture: longitudinally striate to rugose, setal pits spanning interspaces. T4 metasomal flanges: absent. T5 sculpture: longitudinally striate to rugose, setal pits spanning interspaces. T5 metasomal flanges: absent. T6: broader than long. Major sculpture of T6: umbilicate foveate. Microsculpture of T6: absent. T6 medially: strongly convex, tapering and sloping down to a rounded apex, not separated from apical rim. T6 metasomal flanges: absent. T6 raised peripheral rim: absent. S4 sculpture: longitudinally striate or rugose, setal pits spanning interspaces. S5 sculpture: longitudinally striate to rugose, setal pits spanning interspaces. S5 median carina: present. S6 peripheral carina: absent. S6 apex in relation to T6: not exposed to dorsal view. S6 apex: truncate.

*Male*. Body length 3.2–3.3 mm (n=3). A3: shorter than pedicel; as long as pedicel. A5 tyloid shape: narrow, linear. A6: broader than long. A11: longer than broad. Major sculpture of mesoscutal midlobe anteriorly: umbilicate foveate. Major sculpture of mesoscutal midlobe posteriorly: umbilicate foveate. Microsculpture of mesoscutal midlobe anteriorly: granulate. Microsculpture of mesoscutal midlobe posteriorly: absent. Major sculpture of mesoscutellum centrally: umbilicate foveate. Major sculpture of mesoscutellum peripherally: umbilicate foveate. Microsculpture of mesoscutellum centrally: absent. Microsculpture of mesoscutellum peripherally: absent. Fore wing apex at rest: reaching apex of T5. T1 midlobe longitudinal carinae: 4. T3 metasomal flanges: absent. T4 metasomal flanges: absent. T5 metasomal flanges: absent. T6 metasomal flanges: absent. T7: truncate.

##### Diagnosis.

Both sexes: Frontal depression shallow and broad, crossed by some carinae above dorsal separator; submedian carina weak and irregular. Hyperoccipital carina indicated by rugae. Occipital carina complete, weakly convex medially. Metascutellum subrectangular, deeply concave, slightly emarginate apically, projecting dorsally. Coxa darker than rest of leg. T1 lateral carina expanded laterally. Metasomal flanges absent. Female: A3 not longer than pedicel. A4, A5, A6 broader than long. T1 midlobe with 4 longitudinal carinae anteriorly or with a slight smooth bump obscuring them. Fore wing long enough to reach or exceed metasomal apex. T6 broader than long, blunt apically. Male: A3 not longer than pedicel. A4, A11 broader than long. Fore wing long enough to reach T1 midlobe with 4 longitudinal midlobe carinae. T6 or to metasomal apex. T7 much broader than long, blunt apically, without posterior projections.

##### Etymology.

Latin noun, genitive case, meaning “a pack.”

##### Link to distribution map.

[http://hol.osu.edu/map-full.html?id=307104]

##### Material examined.

Holotype, female: AUSTRALIA: WA, Mount Cooke, 13.I-28.I.1991, malaise trap, M. S. Harvey & J. M. Waldock, OSUC 439555 (deposited in WAMP). Paratypes: AUSTRALIA: 7 females, OSUC 439556, 439558-439563 (WINC).

#### Oxyscelio scismatis

Burks sp. n.

http://zoobank.org/6C67C732-6999-453D-A452-4469771CC2D8

urn:lsid:biosci.ohio-state.edu:osuc_concepts:307105

http://species-id.net/wiki/Oxyscelio_scismatis

**Figures 301–306.**
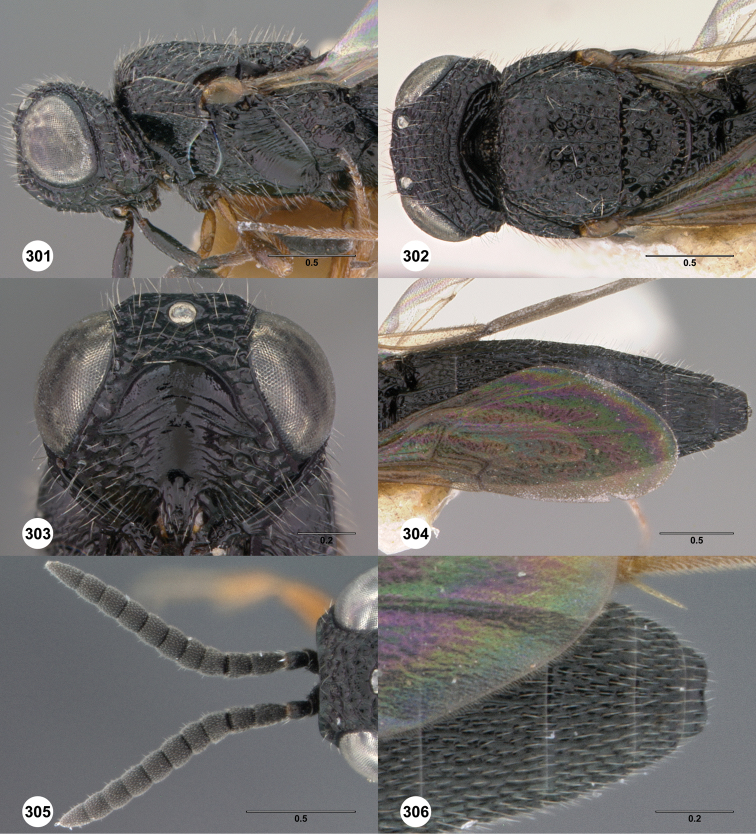
*Oxyscelio scismatis* sp. n., holotype female (OSUC 436037) **301** Head and mesosoma, lateral view **302** Head and mesosoma, dorsal view **303** Head, anterior view **304** Metasoma, dorsal view. Paratype male (OSUC 456181) **305** Antenna **306** Metasoma, dorsal view. Morphbank^85^

Figures 301–306 ; Morphbank^85^

##### Description.

Female. Body length 4.4–4.65 mm (n=3).

Radicle color and shade: same as scape, both dark brown. Pedicel color: same as scape. A3: longer than pedicel. A4: broader than long. A5: broader than long.

Ventral clypeal margin: uniformly convex. Interantennal process: not elongate. Lower frons at dorsal margin of interantennal process: without transverse carina. Transverse curved rugae extending from frontal depression to eye: absent. Median longitudinal carina in frontal depression: absent. Ventral portion of frontal depression: with transverse carinae. Dorsal portion of frontal depression: without transverse carinae. Submedian carina: present. Frontal depression dorsally: hood-like, dorsally protruding. Upper frons major sculpture: umbilicate foveate. Upper frons microsculpture: absent. Hyperoccipital carina: absent. Carina connecting occipital carina to hyperoccipital carina: absent. Occipital carina: weakly arched dorsally, with rounded lateral corners. Occiput sculpture: irregularly sculptured. Extra carina ventral to occipital carina: present, complete. Gena length: shorter than eye. Major sculpture of gena anteroventrally: umbilicate foveate. Major sculpture of gena posteroventrally: umbilicate foveate; absent. Microsculpture of gena anteroventrally: absent. Microsculpture of gena posteroventrally: granulate.

Lateral pronotal area sculpture: anteriorly smooth, posterodorsal corner with dense microsculpture, ventral corner with irregular carinae. Posterior border of central pronotal area: directed anteriorly, protruding at corner of epomial carina and transverse pronotal carina. Mesoscutum anteriorly: very steep and tall, descending at a right angle or protruding anteriorly. Major sculpture of mesoscutal midlobe anteriorly: umbilicate foveate. Mesoscutal midlobe sculpture at midlength: with narrow curved smooth elevations. Major sculpture of mesoscutal midlobe posteriorly: umbilicate foveate. Microsculpture of mesoscutal midlobe anteriorly: granulate. Microsculpture of mesoscutal midlobe posteriorly: punctate. Median mesoscutal carina: present as a ruga. Major sculpture of mesoscutellum centrally: umbilicate foveate. Major sculpture of mesoscutellum peripherally: umbilicate foveate. Microsculpture of mesoscutellum centrally: absent; punctate. Microsculpture of mesoscutellum peripherally: absent; punctate. Mesoscutellar rim: not expanded. Mesoscutellar rim medially: without notch. Mesofemoral depression: longitudinally striate dorsally, smooth ventrally. Metascutellum shape: not emarginate, concave but elevated posteriorly. Metascutellar setae: absent. Metascutellum sculpture: with large smooth posterior fovea. Postmarginal vein: absent. Fore wing apex at rest: reaching base of T5. Coxae color brightness: darker than femora. Spines along tibiae: absent. Lateral propodeal carinae: broadly separated, not parallel anteriorly. Setae in metasomal depression: absent. Anterior sculpture of metasomal depression: absent. Median propodeal carina: absent.

T1 horn: absent. Number of longitudinal carinae of T1 midlobe: obscured by other raised sculpture. T1 lateral carina: protruding laterally, visible from ventral view. T2 sculpture: with longitudinal striae or rugae, setiferous puncta present between them. T2 sublateral longitudinal foveae: absent. T3 metasomal flanges: absent. T4 sculpture: longitudinally striate to rugose, setal pits spanning interspaces. T4 metasomal flanges: present as slightly protruding sharp corners. T5 sculpture: longitudinally striate to rugose, setal pits spanning interspaces. T5 metasomal flanges: present as strongly protruding acuminate flanges. T6: broader than long. Major sculpture of T6: umbilicate punctate; longitudinally striate. Microsculpture of T6: absent. T6 medially: truncate, separated from apical rim. T6 metasomal flanges: present as slightly expanded lateral rims, truncate posteriorly. T6 raised peripheral rim: absent. S4 sculpture: longitudinally striate or rugose, setal pits spanning interspaces. S5 sculpture: longitudinally striate to rugose, setal pits spanning interspaces. S5 median carina: present. S6 peripheral carina: present, posteriorly complete. S6 apex in relation to T6: exposed to dorsal view by T6 emargination. S6 apex: rounded or acuminate.

*Male*. unknown.

##### Diagnosis.

Both sexes: Frontal depression deep, with interrupted carinae ventrally, dorsal separator complete medially, no carinae present above dorsal separator; submedian carina strongly defined. Hyperoccipital carina absent. Occipital carina complete, medially weakly convex. Mesoscutellar rim not expanded, without median notch. Metascutellum broad and concave, projecting dorsally. Coxa darker than rest of leg. Postmarginal vein absent. Female: A3 longer than pedicel. A4, A5 broader than long. T4 without metasomal flanges, T5 with lobe-like metasomal flanges. Main surface of T6 with slightly expanded lateral margins with sharp posterior corners, truncate or very slightly emarginate apically, but sharply separated from apical rim. S6 exposed to dorsal view, rounded apically.

##### Etymology.

Latin noun, genitive case, meaning “a separation.”

##### Link to distribution map.

[http://hol.osu.edu/map-full.html?id=307105]

##### Material examined.

Holotype, female: AUSTRALIA: ACT, Canberra, 2.II.1950, E. F. Riek, OSUC 436037 (deposited in ANIC). Paratypes: AUSTRALIA: 3 females, 2 males, OSUC 436035-436036 (ANIC); OSUC 456180-456182 (WINC).

#### Oxyscelio sciuri

Burks sp. n.

http://zoobank.org/2788E3BD-0493-40A3-A7A9-9A54E6146215

urn:lsid:biosci.ohio-state.edu:osuc_concepts:307106

http://species-id.net/wiki/Oxyscelio_sciuri

**Figures 307–312.**
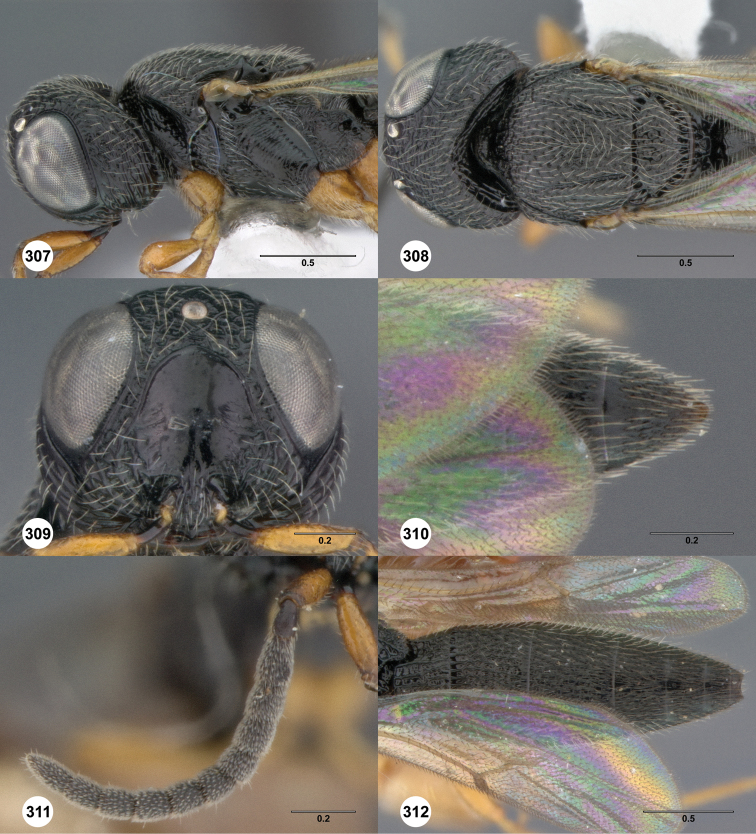
*Oxyscelio sciuri* sp. n., paratype female (OSUC 462577) **307** Head and mesosoma, lateral view **308** Head and mesosoma, dorsal view. Paratype female (OSUC 462576) **309** Head, anterior view **310** Metasoma, dorsal view. Paratype male (OSUC 368208) **311** Antenna **312** Metasoma, dorsal view. Morphbank^86^

Figures 307–312 ; Morphbank^86^

##### Description.

Female. Body length 4.05–4.5 mm (n=6).

Radicle color and shade: darker than scape. Pedicel color: same as scape. A3: shorter than pedicel; as long as pedicel. A4: longer than broad. A5: broader than long.

Ventral clypeal margin: with slightly convex median lobe. Interantennal process: not elongate. Lower frons at dorsal margin of interantennal process: without transverse carina. Transverse curved rugae extending from frontal depression to eye: absent. Median longitudinal carina in frontal depression: absent. Ventral portion of frontal depression: smooth. Dorsal portion of frontal depression: without transverse carinae. Submedian carina: present. Frontal depression dorsally: not hood-like, open dorsally. Upper frons major sculpture: umbilicate foveate; irregularly rugose. Upper frons microsculpture: absent. Hyperoccipital carina: indicated by a set of irregular elevations. Carina connecting occipital carina to hyperoccipital carina: absent. Occipital carina: broadly angular, with rounded median peak. Occiput sculpture: irregularly sculptured. Extra carina ventral to occipital carina: absent. Gena length: as long as eye. Major sculpture of gena anteroventrally: rugose; umbilicate punctate. Major sculpture of gena posteroventrally: rugose; umbilicate punctate. Microsculpture of gena anteroventrally: absent; granulate. Microsculpture of gena posteroventrally: absent; granulate.

Lateral pronotal area sculpture: with shallow irregular carinae, posterodorsal corner with dense microsculpture. Posterior border of central pronotal area: directed anteriorly, protruding at corner of epomial carina and transverse pronotal carina. Mesoscutum anteriorly: very steep and tall, descending at a right angle or protruding anteriorly. Major sculpture of mesoscutal midlobe anteriorly: umbilicate foveate. Mesoscutal midlobe sculpture at midlength: not different from nearby sculpture. Major sculpture of mesoscutal midlobe posteriorly: umbilicate foveate; longitudinally rugose. Microsculpture of mesoscutal midlobe anteriorly: granulate. Microsculpture of mesoscutal midlobe posteriorly: absent. Median mesoscutal carina: present as a ruga. Major sculpture of mesoscutellum centrally: umbilicate foveate; irregularly rugose. Major sculpture of mesoscutellum peripherally: umbilicate foveate; irregularly rugose. Microsculpture of mesoscutellum centrally: absent. Microsculpture of mesoscutellum peripherally: absent. Mesoscutellar rim: not expanded. Mesoscutellar rim medially: without notch. Mesofemoral depression: longitudinally striate dorsally, smooth ventrally. Metascutellum shape: not emarginate, concave but elevated posteriorly. Metascutellar setae: absent. Metascutellum sculpture: with large smooth posterior fovea. Postmarginal vein: present. Fore wing apex at rest: reaching middle of T5. Coxae color brightness: same color as femora. Spines along tibiae: absent. Lateral propodeal carinae: broadly separated, not parallel anteriorly. Setae in metasomal depression: absent. Anterior sculpture of metasomal depression: absent. Median propodeal carina: absent.

T1 horn: present. Number of longitudinal carinae of T1 midlobe: obscured by other raised sculpture. T1 lateral carina: straight. T2 sculpture: densely foveolate, longitudinal sculpture irregular. T2 sublateral longitudinal foveae: absent. T3 metasomal flanges: absent. T4 sculpture: longitudinally striate to rugose, setal pits spanning interspaces. T4 metasomal flanges: absent. T5 sculpture: longitudinally striate to rugose, setal pits spanning interspaces. T5 metasomal flanges: absent. T6: broader than long. Major sculpture of T6: umbilicate punctate; longitudinally striate. Microsculpture of T6: granulate. T6 medially: flat and tapering to a rounded apex, not separated from apical rim. T6 metasomal flanges: absent. T6 raised peripheral rim: absent. S4 sculpture: longitudinally striate or rugose, setal pits spanning interspaces. S5 sculpture: longitudinally striate to rugose, setal pits spanning interspaces. S5 median carina: present. S6 peripheral carina: absent. S6 apex in relation to T6: not exposed to dorsal view. S6 apex: rounded or acuminate.

*Male*. Body length 3.8–3.95 mm (n=3). A3: longer than pedicel. A5 tyloid shape: narrow, linear. A6: broader than long. A11: longer than broad. Major sculpture of mesoscutal midlobe anteriorly: umbilicate foveate. Major sculpture of mesoscutal midlobe posteriorly: umbilicate foveate. Microsculpture of mesoscutal midlobe anteriorly: granulate. Microsculpture of mesoscutal midlobe posteriorly: absent. Major sculpture of mesoscutellum centrally: umbilicate foveate; irregularly rugose. Major sculpture of mesoscutellum peripherally: umbilicate foveate; irregularly rugose. Microsculpture of mesoscutellum centrally: absent. Microsculpture of mesoscutellum peripherally: absent. Fore wing apex at rest: reaching apex of T5. T1 midlobe longitudinal carinae: 4. T3 metasomal flanges: absent. T4 metasomal flanges: absent. T5 metasomal flanges: absent. T6 metasomal flanges: absent. T7: broadly emarginate, with sharply pointed posterolateral lobes.

##### Diagnosis.

Both sexes: Frontal depression broad, transverse carinae absent; submedian carina strong. Gena greatly enlarged, about as long as the eye, strongly rugose. Hyperoccipital carina indicated by rugae. Occipital carina convex, forming a rounded peak medially. Metascutellum tiny, concave. Postmarginal vein present. Coxa not darker than rest of leg. Metasomal depression smooth. T1 lateral carina not expanded laterally. Metasomal flanges absent. Female: A3 not longer than pedicel. A4 about as long as broad, A5 broader than long. Mesoscutellum without granulate sculpture. T1 midlobe carinae obscured by raised smooth area. Fore wing long enough to reach middle of T5. Male: A4 broader than long, A11 slightly longer than broad. T1 midlobe with 4 longitudinal carinae. Fore wing long enough to reach middle of T6. T7 emarginate apically, with sharp posterior corners angled medially.

##### Etymology.

Latin noun, genitive case, meaning “squirrel.”

##### Link to distribution map.

[http://hol.osu.edu/map-full.html?id=307106]

##### Material examined.

Holotype, female: AUSTRALIA: QLD, Mt. William, rainforest edge, Eungella National Park, 18.IV.1979, E Dahms, OSUC 368212 (deposited in QMBA). Paratypes: AUSTRALIA: 5 females, 3 males, OSUC 368208, 368213 (ANIC); OSUC 227564, 462576-462577 (CNCI); OSUC 448572, 451355 (QDPC); OSUC 368214 (WINC).

##### Comments.

*Oxyscelio sciuri* is the only known species in its genus with a greatly expanded gena. Otherwise, it exhibits features of the *aciculae*-group.

#### Oxyscelio scutorum

Burks sp. n.

http://zoobank.org/00FC118E-552B-4F38-ACDC-410AB49F05EB

urn:lsid:biosci.ohio-state.edu:osuc_concepts:307107

http://species-id.net/wiki/Oxyscelio_scutorum

**Figures 313–318.**
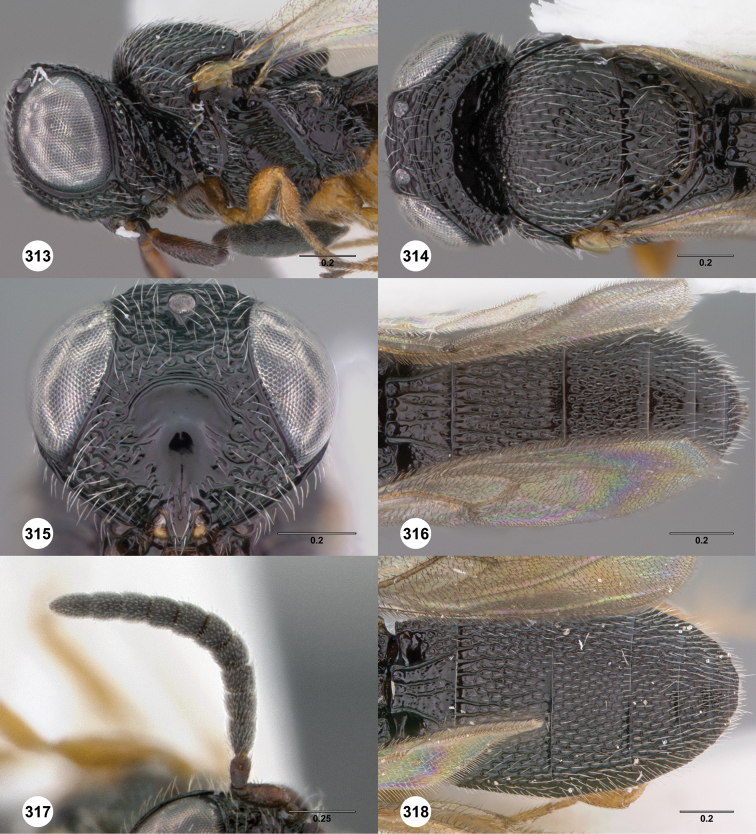
*Oxyscelio scutorum* sp. n., holotype female (OSUC 359721) **313** Head and mesosoma, lateral view **314** Head and mesosoma, dorsal view **315** Head, anterior view **316** Metasoma, dorsal view. Paratype male (OSUC 359730) **317** Antenna **318** Metasoma, dorsal view. Morphbank^87^

Figures 313–318 ; Morphbank^87^

##### Description.

Female. Body length 2.45–3.4 mm (n=20).

Radicle color and shade: darker than scape. Pedicel color: same as scape. A3: shorter than pedicel; as long as pedicel. A4: broader than long. A5: broader than long.

Ventral clypeal margin: with slightly convex median lobe. Interantennal process: not elongate. Lower frons at dorsal margin of interantennal process: without transverse carina. Transverse curved rugae extending from frontal depression to eye: absent. Median longitudinal carina in frontal depression: absent. Ventral portion of frontal depression: with medially interrupted transverse carinae. Dorsal portion of frontal depression: without transverse carinae. Submedian carina: present. Frontal depression dorsally: not hood-like, open dorsally. Upper frons major sculpture: umbilicate foveate; irregularly rugose. Upper frons microsculpture: absent. Hyperoccipital carina: present as a single carina. Carina connecting occipital carina to hyperoccipital carina: absent. Occipital carina: present laterally, absent medially. Occiput sculpture: transversely rugose. Extra carina ventral to occipital carina: absent. Gena length: shorter than eye. Major sculpture of gena anteroventrally: umbilicate foveate. Major sculpture of gena posteroventrally: umbilicate foveate; rugose. Microsculpture of gena anteroventrally: absent. Microsculpture of gena posteroventrally: absent.

Lateral pronotal area sculpture: anteriorly smooth, posterodorsal corner with dense microsculpture, ventral corner with irregular carinae. Posterior border of central pronotal area: directed posteriorly, epomial carina absent or meeting transverse pronotal carina at arch on lateral surface of pronotum. Mesoscutum anteriorly: very steep and tall, descending at a right angle or protruding anteriorly. Major sculpture of mesoscutal midlobe anteriorly: umbilicate foveate. Mesoscutal midlobe sculpture at midlength: not different from nearby sculpture. Major sculpture of mesoscutal midlobe posteriorly: umbilicate foveate; longitudinally rugose. Microsculpture of mesoscutal midlobe anteriorly: granulate. Microsculpture of mesoscutal midlobe posteriorly: absent. Median mesoscutal carina: present as a ruga. Major sculpture of mesoscutellum centrally: umbilicate foveate; obliquely rugose. Major sculpture of mesoscutellum peripherally: umbilicate foveate; obliquely rugose. Microsculpture of mesoscutellum centrally: absent. Microsculpture of mesoscutellum peripherally: absent. Mesoscutellar rim: not expanded. Mesoscutellar rim medially: without notch. Mesofemoral depression: longitudinally striate dorsally, smooth ventrally. Metascutellum shape: deeply emarginate, with the resulting pair of posterior processes subtriangular and directed dorsally. Metascutellar setae: absent. Metascutellum sculpture: with large smooth posterior fovea. Postmarginal vein: present. Fore wing apex at rest: exceeding metasomal apex. Coxae color brightness: same color as femora. Spines along tibiae: absent. Lateral propodeal carinae: broadly separated, not parallel anteriorly. Setae in metasomal depression: absent. Anterior sculpture of metasomal depression: absent. Median propodeal carina: absent.

T1 horn: absent. Number of longitudinal carinae of T1 midlobe: obscured by other raised sculpture. T1 lateral carina: protruding laterally, visible from ventral view. T2 sculpture: with longitudinal striae or rugae, setiferous puncta present between them. T2 sublateral longitudinal foveae: absent. T3 metasomal flanges: absent. T4 sculpture: longitudinally striate to rugose, setal pits spanning interspaces. T4 metasomal flanges: absent. T5 sculpture: longitudinally striate to rugose, setal pits spanning interspaces. T5 metasomal flanges: absent. T6: broader than long. Major sculpture of T6: umbilicate punctate. Microsculpture of T6: absent. T6 medially: flat and tapering to a rounded apex, not separated from apical rim. T6 metasomal flanges: absent. T6 raised peripheral rim: absent. S4 sculpture: longitudinally striate or rugose, setal pits spanning interspaces. S5 sculpture: longitudinally striate to rugose, setal pits spanning interspaces. S5 median carina: present. S6 peripheral carina: absent. S6 apex in relation to T6: not exposed to dorsal view. S6 apex: rounded or acuminate.

*Male*. Body length 2.35–3.35 mm (n=13). A3: longer than pedicel. A5 tyloid shape: narrow, linear. A6: broader than long. A11: longer than broad. Major sculpture of mesoscutal midlobe anteriorly: umbilicate foveate. Major sculpture of mesoscutal midlobe posteriorly: umbilicate foveate; irregularly rugose. Microsculpture of mesoscutal midlobe anteriorly: granulate. Microsculpture of mesoscutal midlobe posteriorly: absent. Major sculpture of mesoscutellum centrally: umbilicate foveate. Major sculpture of mesoscutellum peripherally: umbilicate foveate. Microsculpture of mesoscutellum centrally: absent. Microsculpture of mesoscutellum peripherally: absent. Fore wing apex at rest: exceeding metasomal apex. T1 midlobe longitudinal carinae: 4. T3 metasomal flanges: absent. T4 metasomal flanges: absent. T5 metasomal flanges: absent. T6 metasomal flanges: absent. T7: weakly emarginate.

##### Diagnosis.

Both sexes: Mesoscutum and mesoscutellum black. Frontal depression shallow, transverse carinae absent; submedian carina indicated by a set of irregular elevations or weak carinae. Hyperoccipital carina indicated by sharp carina. Occipital carina with a faintly indicated convex medial portion. Metascutellum concave, broad and short, projecting dorsally. Coxa not darker than rest of leg. T1 lateral carina expanded laterally. Metasomal flanges absent. Female: A3 shorter than pedicel. A4, A5 much broader than long. Mesoscutellum with obliquely raised sculpture. T1 midlobe with 4 longitudinal carinae. T6 without metasomal flanges. Fore wing long enough to reach or exceed metasomal apex. Male: A4 broader than long, A11 longer than broad. T1 midlobe with 4 longitudinal carinae. Fore wing long enough to exceed metasomal apex. T7 much broader than long, blunt apically, without posterior projections. *Oxyscelio scutorum* is very similar to *Oxyscelio brevitas* in having a roughly sculptured occiput and strong hyperoccipital carina. It differs having a stronger hyperoccipital carina and an indicated submedian carina. The raised oblique mesoscutellar sculpture is also distinctive, but it may be difficult to distinguish in some specimens. It sometimes accompanies a flat, broad median mesoscutellar carina.

##### Etymology.

Latin noun, genitive case, meaning “shields.”

##### Link to distribution map.

[http://hol.osu.edu/map-full.html?id=307107]

##### Material examined.

Holotype, female: AUSTRALIA: QLD, 3.5km SSW Mount Baird, 15°10'S, 145°07'E, 3.V–5.V.1981, malaise trap, I. D. Naumann, OSUC 359721 (deposited in ANIC). Paratypes: AUSTRALIA: 22 females, 13 males, ANIC DB 32-020146, OSUC 359716, OSUC 359717, OSUC 359718, OSUC 359719, OSUC 359722, OSUC 359724, OSUC 359730, OSUC 359732, OSUC 359733, OSUC 359734, OSUC 359739, OSUC 359743 (ANIC); OSUC 451368 (BMNH); OSUC 227634, 462754 (CNCI); OSUC 359725 (MVMA); OSUC 451320, QDPC 0-165674 (QDPC); OSUC 359720 (QMBA); OSUC 359713-359715, 359723, 359726, 359728-359729, 359731, 359735, 445346, 448925-448927, 451321-451322 (WINC). Other material: AUSTRALIA: 1 female, OSUC 359727 (WINC).

#### Oxyscelio sepisessor

Burks sp. n.

http://zoobank.org/D798D4E2-4822-4F23-8735-ABA7EA088346

urn:lsid:biosci.ohio-state.edu:osuc_concepts:307108

http://species-id.net/wiki/Oxyscelio_sepisessor

**Figures 319–324.**
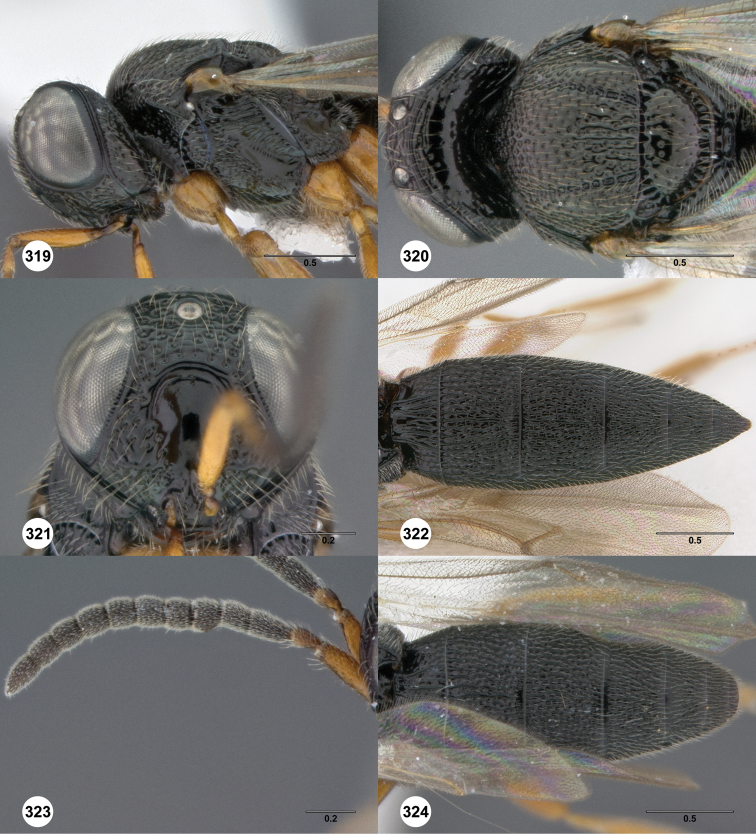
*Oxyscelio sepisessor* sp. n., holotype female (OSUC 438936) **319** Head and mesosoma, lateral view **320** Head and mesosoma, dorsal view **321** Metasoma, dorsal view. Paratype female (OSUC 227542) **322** Head, anterior view. Paratype male (OSUC 438946) **323** Antenna **324** Metasoma, dorsal view. Morphbank^88^

Figures 319–324 ; Morphbank^88^

##### Description.

Female. Body length 3.75–4.3 mm (n=21).

Radicle color and shade: darker than scape. Pedicel color: same as scape. A3: shorter than pedicel. A4: longer than broad. A5: broader than long.

Ventral clypeal margin: with slightly convex median lobe. Interantennal process: not elongate. Lower frons at dorsal margin of interantennal process: with transverse ledge, face sharply receding below it. Transverse curved rugae extending from frontal depression to eye: absent. Median longitudinal carina in frontal depression: absent. Ventral portion of frontal depression: smooth. Dorsal portion of frontal depression: without transverse carinae. Submedian carina: present only as a weak shift in elevation. Frontal depression dorsally: not hood-like, open dorsally. Upper frons major sculpture: umbilicate foveate; transversely rugose. Upper frons microsculpture: absent. Hyperoccipital carina: present as a single carina. Carina connecting occipital carina to hyperoccipital carina: absent. Occipital carina: present laterally, absent medially. Occiput sculpture: transversely rugose. Extra carina ventral to occipital carina: present, medially incomplete. Gena length: shorter than eye. Major sculpture of gena anteroventrally: umbilicate punctate; absent. Major sculpture of gena posteroventrally: umbilicate foveate; absent. Microsculpture of gena anteroventrally: absent. Microsculpture of gena posteroventrally: absent.

Lateral pronotal area sculpture: anteriorly smooth, posterodorsal corner with dense microsculpture, ventral corner with irregular carinae. Posterior border of central pronotal area: directed posteriorly, epomial carina absent or meeting transverse pronotal carina at arch on lateral surface of pronotum. Mesoscutum anteriorly: very steep and tall, descending at a right angle or protruding anteriorly. Major sculpture of mesoscutal midlobe anteriorly: umbilicate foveate. Mesoscutal midlobe sculpture at midlength: not different from nearby sculpture. Major sculpture of mesoscutal midlobe posteriorly: umbilicate foveate; longitudinally rugose. Microsculpture of mesoscutal midlobe anteriorly: absent; granulate. Microsculpture of mesoscutal midlobe posteriorly: absent. Median mesoscutal carina: absent; present as a vague, occasionally interrupted elevation. Major sculpture of mesoscutellum centrally: umbilicate foveate. Major sculpture of mesoscutellum peripherally: umbilicate foveate; umbilicate punctate. Microsculpture of mesoscutellum centrally: absent. Microsculpture of mesoscutellum peripherally: absent. Mesoscutellar rim: not expanded. Mesoscutellar rim medially: without notch. Mesofemoral depression: longitudinally striate dorsally, smooth ventrally. Metascutellum shape: not emarginate, concave but elevated posteriorly. Metascutellar setae: absent. Metascutellum sculpture: with large smooth posterior fovea. Postmarginal vein: present. Fore wing apex at rest: reaching middle of T6. Coxae color brightness: same color as femora. Spines along tibiae: absent. Lateral propodeal carinae: broadly separated, not parallel anteriorly. Setae in metasomal depression: absent. Anterior sculpture of metasomal depression: absent. Median propodeal carina: absent.

T1 horn: absent. Number of longitudinal carinae of T1 midlobe: obscured by other raised sculpture. T1 lateral carina: straight. T2 sculpture: densely foveolate, longitudinal sculpture irregular. T2 sublateral longitudinal foveae: absent. T3 metasomal flanges: absent. T4 sculpture: longitudinally striate to rugose, setal pits spanning interspaces. T4 metasomal flanges: absent. T5 sculpture: longitudinally striate to rugose, setal pits spanning interspaces. T5 metasomal flanges: absent. T6: broader than long. Major sculpture of T6: longitudinally striate; umbilicate foveate. Microsculpture of T6: absent. T6 medially: flat and tapering to a rounded apex, not separated from apical rim. T6 metasomal flanges: absent. T6 raised peripheral rim: absent. S4 sculpture: longitudinally striate or rugose, setal pits spanning interspaces. S5 sculpture: longitudinally striate to rugose, setal pits spanning interspaces. S5 median carina: present. S6 peripheral carina: absent. S6 apex in relation to T6: not exposed to dorsal view. S6 apex: rounded or acuminate.

*Male*. Body length 3.6–4 mm (n=5). A3: shorter than pedicel. A5 tyloid shape: expanded, ovate or sinuate. A6: broader than long. A11: longer than broad. Major sculpture of mesoscutal midlobe anteriorly: umbilicate foveate. Major sculpture of mesoscutal midlobe posteriorly: umbilicate foveate; longitudinally rugose. Microsculpture of mesoscutal midlobe anteriorly: granulate. Microsculpture of mesoscutal midlobe posteriorly: absent. Major sculpture of mesoscutellum centrally: umbilicate foveate. Major sculpture of mesoscutellum peripherally: umbilicate foveate. Microsculpture of mesoscutellum centrally: absent. Microsculpture of mesoscutellum peripherally: absent. Fore wing apex at rest: exceeding metasomal apex. T1 midlobe longitudinal carinae: 3. T3 metasomal flanges: absent. T4 metasomal flanges: absent. T5 metasomal flanges: absent. T6 metasomal flanges: absent. T7: truncate.

##### Diagnosis.

Both sexes: Frontal depression shallow, transverse carinae absent; submedian carina indicated by a set of weak rugae. Hyperoccipital carina sharp and strong. Occipital carina incomplete (difficult to discern due to rough sculpture). Metascutellum broad and concave, truncate or slightly emarginate apically, projecting dorsally. Postmarginal vein present. Coxa not darker than rest of leg. T1 lateral carina not expanded laterally. Metasomal flanges absent. Female: A3 much longer than pedicel. A4 about as long as broad, A5 broader than long. Mesoscutellum without granulate sculpture. T1 midlobe carinae obscured by raised smooth area. T6 without metasomal flanges, main body of tergum sloping down to apical rim. Fore wing long enough to reach base of T6. Male: Flagellomeres beyond A3 broader than long or only slightly longer than broad. T1 midlobe with 3 longitudinal carinae. Fore wing long enough to reach to or beyond T7. T7 truncate or very weakly emarginate.

##### Etymology.

Latin noun in apposition, meaning “the fence-sitter.”

##### Link to distribution map.

[http://hol.osu.edu/map-full.html?id=307108]

##### Material examined.

Holotype, female: AUSTRALIA: QLD, Brisbane, DPI Indooroopilly site, 8.II.1971, malaise trap, OSUC 438936 (deposited in QMBA). Paratypes: AUSTRALIA: 20 females, 5 males, OSUC 438930, 438946 (ANIC); OSUC 227542, 462561 (CNCI); QDPC 0-165646 (QDPC); OSUC 438931-438935, 438937-438945, 438947-438949, 438951, 451325-451326 (WINC).

##### Comments.

*Oxyscelio sepisessor* is provisionally placed in the *flavipes*-group because of its incomplete occipital carina and simple T1 lateral carina that is not expanded. It resembles some *atricoxa*-group species in having a roughly-sculptured occiput and relatively broad metasoma.

#### Oxyscelio shakespearei

(Girault)

http://zoobank.org/EFF58B60-816A-41DA-BE68-B8B040CB83DC

urn:lsid:biosci.ohio-state.edu:osuc_concepts:5034

http://species-id.net/wiki/Oxyscelio_shakespearei

**Figures 325–330.**
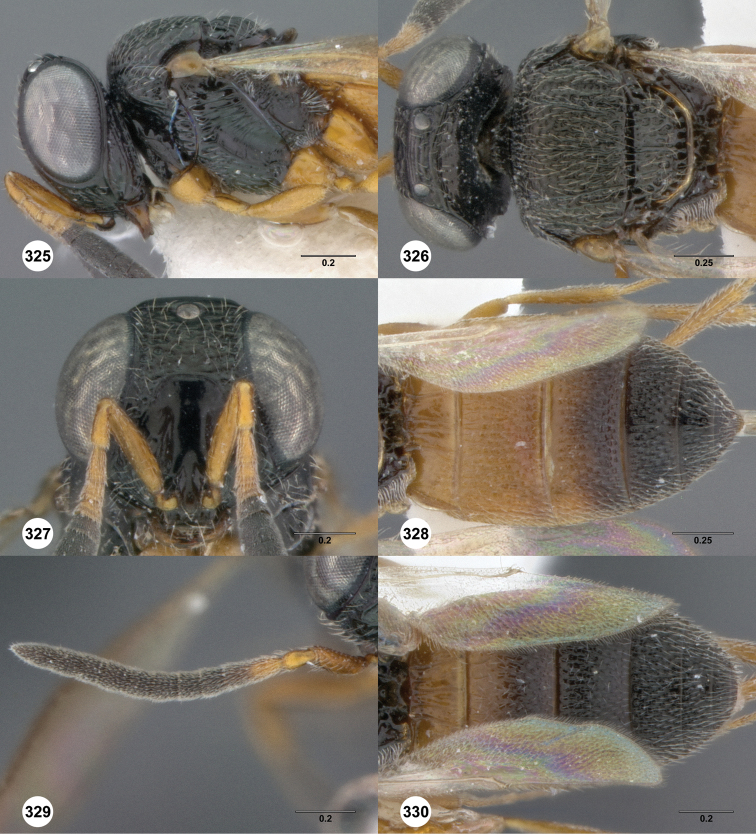
*Oxyscelio shakespearei* (Girault), female (OSUC 442355) **325** Head and mesosoma, lateral view. Female (OSUC 227547) **326** Head and mesosoma, dorsal view **327** Head, anterior view **328** Metasoma, dorsal view. Paratype male (OSUC 227546) **329** Antenna **330** Metasoma, dorsal view. Morphbank^89^

Figures 325–330 ; Morphbank^89^

Oxyscelio shakespearei *Scelio Shakespearei* Girault, 1926: 1 (original description); Gordh et al. 1979: 197 (reprint of Girault (1926)).
Sceliomorpha shakespearei (Girault): Dodd 1927: 128 (generic transfer).
Oxyscelio shakespearei (Girault): Dodd 1931: 76 (generic transfer); Galloway 1976: 100 (type information).

##### Description.

Female. Body length 2.25–3.15 mm (n=20).

Radicle color and shade: same as scape, both yellowish or reddish. Pedicel color: same as scape. A3: shorter than pedicel. A4: as long as broad. A5: broader than long.

Ventral clypeal margin: concave. Interantennal process: not elongate. Lower frons at dorsal margin of interantennal process: without transverse carina. Transverse curved rugae extending from frontal depression to eye: absent. Median longitudinal carina in frontal depression: absent. Ventral portion of frontal depression: smooth. Dorsal portion of frontal depression: without transverse carinae. Submedian carina: present only as a weak shift in elevation. Frontal depression dorsally: not hood-like, open dorsally. Upper frons major sculpture: umbilicate foveate; transversely rugose. Upper frons microsculpture: granulate. Hyperoccipital carina: present as a single carina. Carina connecting occipital carina to hyperoccipital carina: absent. Occipital carina: present laterally, absent medially. Occiput sculpture: smooth. Extra carina ventral to occipital carina: present, complete. Gena length: shorter than eye. Major sculpture of gena anteroventrally: umbilicate foveate. Major sculpture of gena posteroventrally: umbilicate foveate; rugose. Microsculpture of gena anteroventrally: granulate. Microsculpture of gena posteroventrally: granulate.

Lateral pronotal area sculpture: smooth anteriorly, densely setose posteriorly. Posterior border of central pronotal area: directed posteriorly, epomial carina absent or meeting transverse pronotal carina at arch on lateral surface of pronotum. Mesoscutum anteriorly: very steep and tall, descending at a right angle or protruding anteriorly. Major sculpture of mesoscutal midlobe anteriorly: umbilicate foveate. Mesoscutal midlobe sculpture at midlength: not different from nearby sculpture. Major sculpture of mesoscutal midlobe posteriorly: umbilicate foveate; longitudinally rugose. Microsculpture of mesoscutal midlobe anteriorly: granulate. Microsculpture of mesoscutal midlobe posteriorly: absent. Median mesoscutal carina: present as a vague, occasionally interrupted elevation. Major sculpture of mesoscutellum centrally: umbilicate foveate; longitudinally rugose. Major sculpture of mesoscutellum peripherally: umbilicate foveate; longitudinally rugose. Microsculpture of mesoscutellum centrally: absent. Microsculpture of mesoscutellum peripherally: absent. Mesoscutellar rim: not expanded. Mesoscutellar rim medially: without notch. Mesofemoral depression: longitudinally striate dorsally, smooth ventrally. Metascutellum shape: slightly emarginate posteriorly, concave but elevated posteriorly. Metascutellar setae: absent. Metascutellum sculpture: with large smooth posterior fovea. Spines along tibiae: absent. Lateral propodeal carinae: broadly separated, not parallel anteriorly. Setae in metasomal depression: absent. Anterior sculpture of metasomal depression: absent. Median propodeal carina: absent. Postmarginal vein: absent. Fore wing apex at rest: reaching middle of T6. Coxae color brightness: same color as femora.

T1 horn: absent. Number of longitudinal carinae of T1 midlobe: obscured by other raised sculpture. T1 lateral carina: protruding laterally, visible from ventral view. T2 sculpture: with longitudinal striae or rugae, setiferous puncta present between them. T2 sublateral longitudinal foveae: absent. T3 metasomal flanges: absent. T4 sculpture: longitudinally striate to rugose, setal pits spanning interspaces. T4 metasomal flanges: absent. T5 sculpture: longitudinally striate to rugose, setal pits spanning interspaces. T5 metasomal flanges: absent. T6: broader than long. Major sculpture of T6: umbilicate punctate. Microsculpture of T6: absent. T6 medially: strongly convex, tapering and sloping down to a rounded apex, not separated from apical rim. T6 metasomal flanges: absent. T6 raised peripheral rim: absent. S4 sculpture: longitudinally striate or rugose, setal pits spanning interspaces. S5 sculpture: longitudinally striate to rugose, setal pits spanning interspaces. S5 median carina: absent. S6 peripheral carina: absent. S6 apex in relation to T6: not exposed to dorsal view. S6 apex: rounded or acuminate.

*Male*. Body length 2.15–3 mm (n=20). A3: longer than pedicel. A5 tyloid shape: narrow, linear. A6: broader than long. A11: longer than broad. Major sculpture of mesoscutal midlobe anteriorly: umbilicate foveate. Major sculpture of mesoscutal midlobe posteriorly: umbilicate foveate; longitudinally rugose. Microsculpture of mesoscutal midlobe anteriorly: granulate. Microsculpture of mesoscutal midlobe posteriorly: absent. Major sculpture of mesoscutellum centrally: umbilicate foveate; irregularly rugose. Major sculpture of mesoscutellum peripherally: umbilicate foveate; irregularly rugose. Microsculpture of mesoscutellum centrally: absent. Microsculpture of mesoscutellum peripherally: absent. Fore wing apex at rest: exceeding metasomal apex. T1 midlobe longitudinal carinae: 4. T3 metasomal flanges: absent. T4 metasomal flanges: absent. T5 metasomal flanges: absent. T6 metasomal flanges: absent. T7: truncate.

##### Diagnosis.

Both sexes: Frontal depression shallow; submedian carina absent or weakly indicated by a ruga. Hyperoccipital carina indicated by set of strong rugae. Occipital carina incomplete, nearly reaching hyperoccipital carina laterally. Metascutellum concave, emarginate apically, projecting dorsally. Postmarginal vein absent. Coxa not darker than rest of leg. T1 lateral carina expanded laterally. Metasomal flanges absent. Female: A4, A5 broader than long. Mesoscutellum with strong longitudinal rugae. T1 midlobe carinae obscured by raised smooth area. T6 without metasomal flanges, main body of tergum not abruptly separated from apical rim. Fore wing long enough to reach beyond metasomal apex. Male: A4, A11 as long or slightly longer than broad. Mesoscutellum with very strong rugose sculpture. T1 midlobe with 4 longitudinal carinae. Fore wing long enough to reach beyond metasomal apex. T7 tiny, truncate.

##### Link to distribution map.

[http://hol.osu.edu/map-full.html?id=5034]

##### Material examined.

Holotype, female, S. Shakespearei: AUSTRALIA: QLD, Gordonvale (Nelson), no date, QMBA HY3118 (deposited in QMBA). Other material: AUSTRALIA: 23 females, 29 males, OSUC 442355-442357, 442365, 442367-442372, 442375-442398 (ANIC); OSUC 227545-227547 (CNCI); OSUC 451327-451328 (QDPC); UCRC ENT 76364 (UCRC); OSUC 442373 (UQIC); OSUC 442358-442364, 442366, 442399-442401 (WINC).

##### Comments.

*Oxyscelio shakespearei* is a very distinctive species recognizable by its strong mesoscutellar sculpture. The combination of an expanded T1 lateral carina, incomplete occipital carina, mostly smooth occiput, and sharp hyperoccipital carina also occurs in *Oxyscelio fluctuum*. It is difficult to be sure of the species group placement of *Oxyscelio shakespearei*. The lateral carina of T1 and overall body shape and proportions favor placing it in the *atricoxa*-group, but the sharp occipital carina and smooth occiput suggest that it may be closely related to the *flavipes*-group instead.

#### Oxyscelio sinuationis

Burks sp. n.

http://zoobank.org/46ED629A-DBDF-4408-89BB-E228DD3C93D9

urn:lsid:biosci.ohio-state.edu:osuc_concepts:307109

http://species-id.net/wiki/Oxyscelio_sinuationis

**Figures 331–334.**
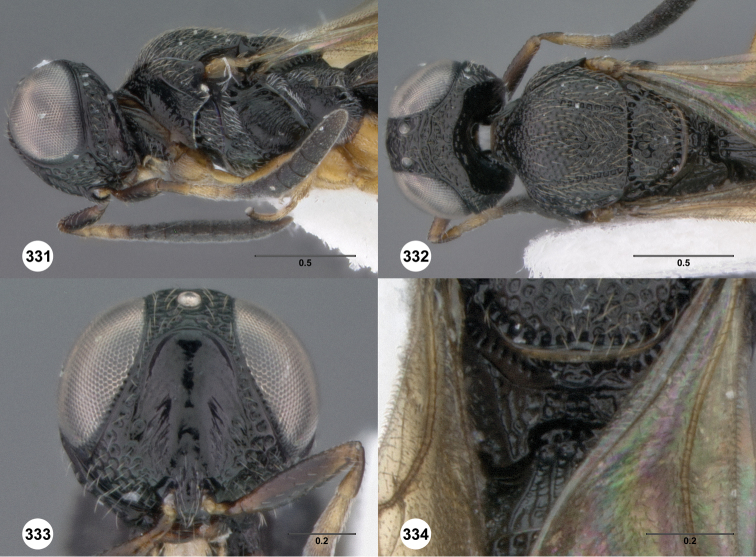
*Oxyscelio sinuationis* sp. n., holotype female (OSUC 368209) **331** Head and mesosoma, lateral view **332** Head and mesosoma, dorsal view **333** Head, anterior view **334** Propodeum, posterodorsal view. Morphbank^90^

Figures 331–334 ; Morphbank^90^

##### Description.

Female. Body length 2.75–3.65 mm (n=6).

Radicle color and shade: same as scape, both dark brown. Pedicel color: same as scape. A3: shorter than pedicel. A4: longer than broad. A5: broader than long.

Ventral clypeal margin: concave. Interantennal process: not elongate. Lower frons at dorsal margin of interantennal process: without transverse carina. Transverse curved rugae extending from frontal depression to eye: absent. Median longitudinal carina in frontal depression: absent. Ventral portion of frontal depression: with medially interrupted transverse carinae. Dorsal portion of frontal depression: without transverse carinae. Submedian carina: present. Frontal depression dorsally: not hood-like, open dorsally. Upper frons major sculpture: umbilicate foveate; transversely rugose. Upper frons microsculpture: absent. Hyperoccipital carina: indicated by a set of irregular elevations. Carina connecting occipital carina to hyperoccipital carina: present. Occipital carina: omicron-shaped, with sharp corners where median portion meets lateral portions. Occiput sculpture: irregularly sculptured. Extra carina ventral to occipital carina: absent. Gena length: shorter than eye. Major sculpture of gena anteroventrally: umbilicate foveate. Major sculpture of gena posteroventrally: absent. Microsculpture of gena anteroventrally: absent. Microsculpture of gena posteroventrally: granulate.

Lateral pronotal area sculpture: anteriorly smooth, posterodorsal corner with dense microsculpture, ventral corner with irregular carinae. Posterior border of central pronotal area: directed posteriorly, epomial carina absent or meeting transverse pronotal carina at arch on lateral surface of pronotum. Mesoscutum anteriorly: not steep, forming less than a right angle. Major sculpture of mesoscutal midlobe anteriorly: umbilicate foveate. Mesoscutal midlobe sculpture at midlength: not different from nearby sculpture. Major sculpture of mesoscutal midlobe posteriorly: umbilicate foveate. Microsculpture of mesoscutal midlobe anteriorly: granulate. Microsculpture of mesoscutal midlobe posteriorly: absent. Median mesoscutal carina: absent. Major sculpture of mesoscutellum centrally: umbilicate foveate. Major sculpture of mesoscutellum peripherally: umbilicate foveate. Microsculpture of mesoscutellum centrally: absent. Microsculpture of mesoscutellum peripherally: absent. Mesoscutellar rim: not expanded. Mesoscutellar rim medially: without notch. Mesofemoral depression: longitudinally striate dorsally and ventrally. Metascutellum shape: slightly emarginate posteriorly, concave but elevated posteriorly. Metascutellar setae: absent. Metascutellum sculpture: with large smooth posterior fovea. Postmarginal vein: present. Fore wing apex at rest: reaching middle of T6. Coxae color brightness: same color as femora. Spines along tibiae: absent. Lateral propodeal carinae: narrowly separated, angled anteriorly to become parallel. Setae in metasomal depression: absent. Anterior sculpture of metasomal depression: with median areole or pair of pits. Median propodeal carina: present.

T1 horn: absent. Number of longitudinal carinae of T1 midlobe: 6. T1 lateral carina: straight. T2 sculpture: densely foveolate, longitudinal sculpture irregular. T2 sublateral longitudinal foveae: absent. T3 metasomal flanges: absent. T4 sculpture: longitudinally striate to rugose, setal pits spanning interspaces. T4 metasomal flanges: absent. T5 sculpture: longitudinally striate to rugose, setal pits spanning interspaces. T5 metasomal flanges: absent. T6: broader than long. Major sculpture of T6: umbilicate punctate. Microsculpture of T6: absent. T6 medially: flat and tapering to a rounded apex, not separated from apical rim. T6 metasomal flanges: absent. T6 raised peripheral rim: absent. S4 sculpture: longitudinally striate or rugose, setal pits spanning interspaces. S5 sculpture: longitudinally striate to rugose, setal pits spanning interspaces. S5 median carina: absent. S6 peripheral carina: absent. S6 apex in relation to T6: not exposed to dorsal view. S6 apex: rounded or acuminate.

*Male*. unknown.

##### Diagnosis.

Both sexes: Frontal depression deep, transverse carinae very weak and interrupted; submedian carina strong. Hyperoccipital carina indicated by rugae. Occipital carina complete, laterally with strong corners and medially sinuate. Metascutellum exceedingly tiny, concave. Postmarginal vein present. Coxa not darker than rest of leg. Metasomal depression roughly sculptured. T1 lateral carina not expanded laterally. Metasomal flanges absent. Female only: A4 longer than broad, A5 slightly broader than long. Mesoscutellum without granulate sculpture. T1 midlobe with 5 longitudinal carinae. Fore wing long enough to reach middle of middle or apex of T6. T6 broader than long.

##### Etymology.

Latin noun, genitive case, meaning “a curve.”

##### Link to distribution map.

[http://hol.osu.edu/map-full.html?id=307109]

##### Material examined.

Holotype, female: AUSTRALIA: QLD, GS2, Mount Edith, 17°06'S, 145°38'E, 1050m, 3.I–4.II.1995, malaise trap, P. Zborowski, OSUC 368209 (deposited in ANIC). Paratypes: AUSTRALIA: 6 females, OSUC 449003 (BMNH); OSUC 462575 (CNCI); OSUC 449004 (UQIC); OSUC 368210-368211, 449002 (WINC).

##### Comments.

*Oxyscelio sinuationis* is among the species in the *aciculae*-group with a roughly sculptured metasomal depression and without a T1 horn. It can otherwise be recognized by its tall, sinuate occipital carina and tiny metascutellum.

#### Oxyscelio solitarius

(Dodd)

http://zoobank.org/9D21D17A-7AE4-4803-A722-6D0BF7419E8C

urn:lsid:biosci.ohio-state.edu:osuc_concepts:5035

http://species-id.net/wiki/Oxyscelio_solitarius

**Figures 335–340.**
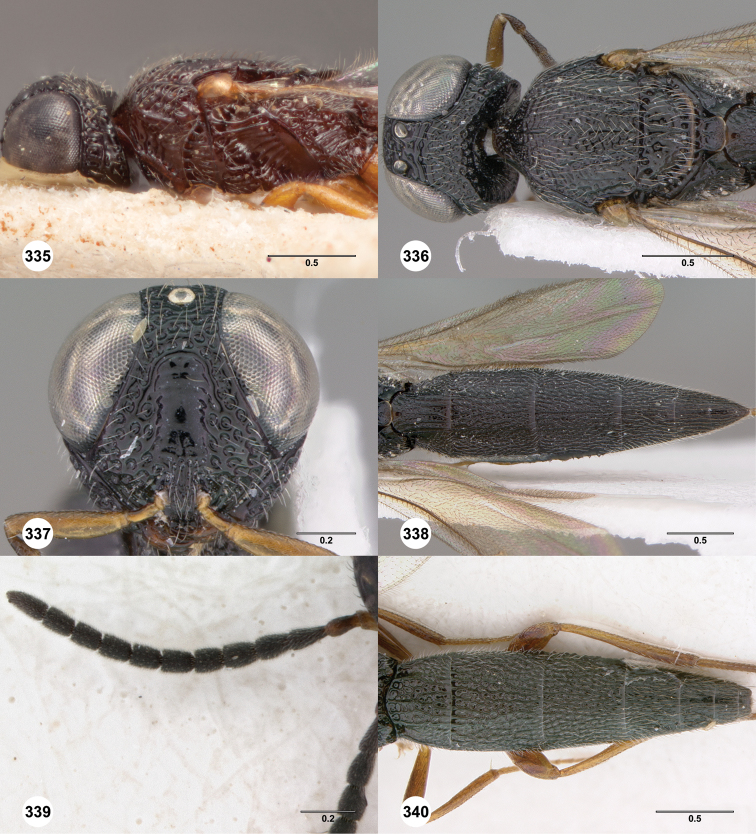
*Oxyscelio solitarius* (Dodd), female (OSUC 368084) **335** Head and mesosoma, lateral view **336** Head and mesosoma, dorsal view **337** Head, anterior view **338** Metasoma, dorsal view. Paratype male (OSUC 368104) **339** Antenna **340** Metasoma, dorsal view. Morphbank^91^

Figures 335–340 ; Morphbank^91^

Dicroteleia solitaria Dodd, 1914: 105 (original description); Kieffer 1926: 387, 390 (description, keyed).
Oxyscelio solitarius (Dodd): Dodd 1931: 76 (generic transfer); Galloway 1976: 100 (type information).

##### Description.

Female. Body length 4.1–4.95 mm (n=20).

Radicle color and shade: same as scape, both yellowish or reddish. Pedicel color: at least partially darker than scape. A3: longer than pedicel. A4: longer than broad. A5: longer than broad.

Ventral clypeal margin: with slightly convex median lobe. Interantennal process: not elongate. Lower frons at dorsal margin of interantennal process: without transverse carina. Transverse curved rugae extending from frontal depression to eye: absent. Median longitudinal carina in frontal depression: absent. Ventral portion of frontal depression: smooth. Dorsal portion of frontal depression: with some transverse carinae. Submedian carina: present only as a weak shift in elevation. Frontal depression dorsally: not hood-like, open dorsally. Upper frons major sculpture: umbilicate foveate; transversely rugose. Upper frons microsculpture: absent. Hyperoccipital carina: indicated by a set of irregular elevations. Carina connecting occipital carina to hyperoccipital carina: present. Occipital carina: present laterally, absent medially. Occiput sculpture: umbilicate foveate. Extra carina ventral to occipital carina: absent. Gena length: shorter than eye. Major sculpture of gena anteroventrally: umbilicate foveate. Major sculpture of gena posteroventrally: umbilicate foveate. Microsculpture of gena anteroventrally: punctate. Microsculpture of gena posteroventrally: punctate.

Lateral pronotal area sculpture: anteriorly smooth, posterodorsal corner with dense microsculpture, ventral corner with irregular carinae. Posterior border of central pronotal area: directed anteriorly, protruding at corner of epomial carina and transverse pronotal carina. Mesoscutum anteriorly: not steep, forming less than a right angle. Major sculpture of mesoscutal midlobe anteriorly: umbilicate foveate; transversely rugose. Mesoscutal midlobe sculpture at midlength: not different from nearby sculpture. Major sculpture of mesoscutal midlobe posteriorly: umbilicate foveate. Microsculpture of mesoscutal midlobe anteriorly: absent. Microsculpture of mesoscutal midlobe posteriorly: absent. Median mesoscutal carina: present as a flattened or rounded elevation. Major sculpture of mesoscutellum centrally: umbilicate foveate. Major sculpture of mesoscutellum peripherally: umbilicate foveate; umbilicate punctate. Microsculpture of mesoscutellum centrally: absent. Microsculpture of mesoscutellum peripherally: absent. Mesoscutellar rim: not expanded. Mesoscutellar rim medially: without notch. Mesofemoral depression: longitudinally striate dorsally and ventrally. Metascutellum shape: not emarginate, forming a flat, concave shelf. Metascutellar setae: absent. Metascutellum sculpture: with a median carina ending in a broad granulate patch posteriorly. Spines along tibiae: absent. Lateral propodeal carinae: broadly separated, not parallel anteriorly. Setae in metasomal depression: absent. Anterior sculpture of metasomal depression: absent. Median propodeal carina: absent. Postmarginal vein: present. Fore wing apex at rest: reaching middle of T5. Coxae color brightness: same color as femora.

T1 horn: present. Number of longitudinal carinae of T1 midlobe: obscured by other raised sculpture. T1 lateral carina: straight. T2 sculpture: densely foveolate with granulate microsculpture, longitudinal sculpture irregular. T2 sublateral longitudinal foveae: present. T3 metasomal flanges: absent. T4 sculpture: longitudinally striate to rugose, setal pits spanning interspaces. T4 metasomal flanges: absent. T5 sculpture: longitudinally striate to rugose, setal pits spanning interspaces. T5 metasomal flanges: absent. T6: longer than broad. Major sculpture of T6: umbilicate punctate. Microsculpture of T6: granulate. T6 medially: flat and tapering to a rounded apex, not separated from apical rim. T6 metasomal flanges: absent. T6 raised peripheral rim: absent. S4 sculpture: longitudinally striate or rugose, setal pits spanning interspaces. S5 sculpture: longitudinally striate to rugose, setal pits spanning interspaces. S5 median carina: absent. S6 peripheral carina: absent. S6 apex in relation to T6: not exposed to dorsal view. S6 apex: rounded or acuminate.

*Male*. Body length 8.1–9.2 mm (n=11). A3: longer than pedicel. A5 tyloid shape: narrow, linear. A6: longer than broad. A11: longer than broad. Major sculpture of mesoscutal midlobe anteriorly: umbilicate foveate; transversely rugose. Major sculpture of mesoscutal midlobe posteriorly: umbilicate foveate; transversely rugose. Microsculpture of mesoscutal midlobe anteriorly: absent. Microsculpture of mesoscutal midlobe posteriorly: absent. Major sculpture of mesoscutellum centrally: umbilicate foveate. Major sculpture of mesoscutellum peripherally: umbilicate foveate. Microsculpture of mesoscutellum centrally: absent. Microsculpture of mesoscutellum peripherally: absent. Fore wing apex at rest: reaching middle of T5. T1 midlobe longitudinal carinae: 5. T3 metasomal flanges: absent. T4 metasomal flanges: absent. T5 metasomal flanges: absent. T6 metasomal flanges: absent. T7: M-shaped, with a triangular median emargination.

##### Diagnosis.

Both sexes: Frontal depression nearly flat, dorsally crossed by some weak arched carinae. Mesoscutum and mesoscutellum without granulate sculpture. Metascutellum flat with concave areas anteriorly and granulate sculpture posteriorly. Postmarginal vein present. Female: A4 longer than broad. T1 midlobe with broad horn, without longitudinal carinae. T2, T3 with sublateral longitudinal depressions. T6 not sharply pointed apically. Male: All flagellomeres longer than broad. Metasomal depression with antero-medial sculpture. T1 midlobe with 5 longitudinal carinae. T2 with slight longitudinal sublateral depressions. T7 with rounded posterior lobes.

##### Link to distribution map.

[http://hol.osu.edu/map-full.html?id=5035]

##### Material examined.

Holotype, female, D. solitaria: AUSTRALIA: QLD, nr. Cairns, forest, Gordonvale (Nelson), 20.VI.1914, sweeping, A. A. Girault, SAMA DB 32-001591 (deposited in SAMA). Other material: AUSTRALIA: 30 females, 12 males, ANIC DB 32-020103, 32-020155, OSUC 367990, OSUC 367992, OSUC 368088, OSUC 368089, OSUC 368092, OSUC 368093, OSUC 368094, OSUC 368095, OSUC 368096, OSUC 368099, OSUC 368100, OSUC 368101, OSUC 368102, OSUC 368103, OSUC 368105 (ANIC); OSUC 227618-227619 (CNCI); OSUC 368106 (MVMA); OSUC 367991 (QDPC); OSUC 148481, 367989, 368104 (QMBA); UCRC ENT 121056 (UCRC); OSUC 368084-368087, 368097-368098, 451307-451317 (WINC).

##### Comments.

*Oxyscelio solitarius* belongs to the *fossarum*-group, which is much more speciose in Asia. This species group is characterized by the elongate sublateral depressions on T2-T3, which correspond with the lateral lobes of T1. Asian species of the *fossarum*-group differ from *Oxyscelio solitarius* in having either extensive granulate mesoscutal and mesoscutellar sculpture, a sharply pointed T6, or in lacking the T1 anterior horn.

#### Oxyscelio sordes

Burks sp. n.

http://zoobank.org/493C22B7-F5D3-489C-B61E-1D9495364367

urn:lsid:biosci.ohio-state.edu:osuc_concepts:307110

http://species-id.net/wiki/Oxyscelio_sordes

**Figures 341–346.**
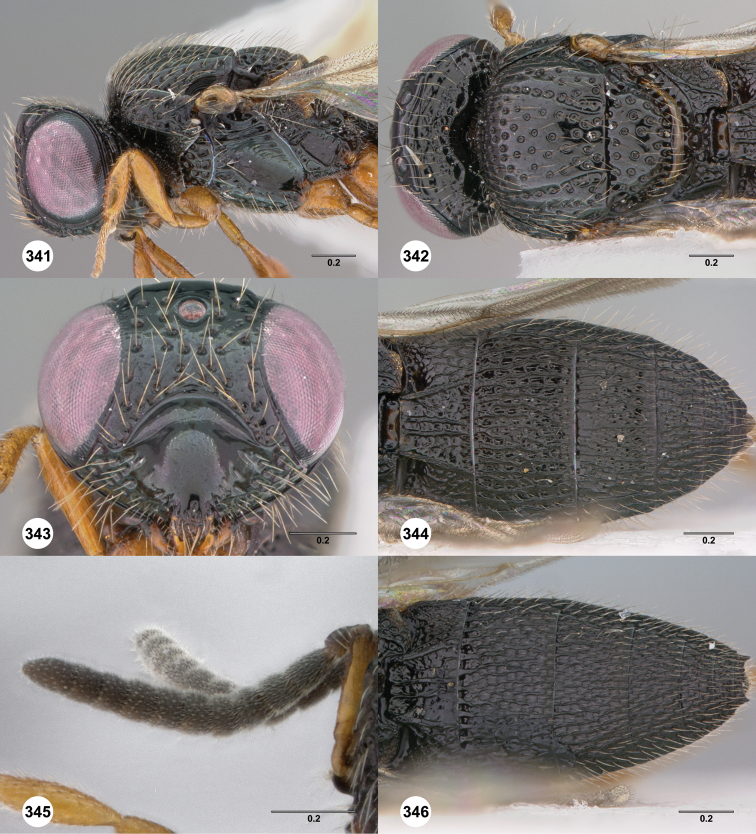
*Oxyscelio sordes* sp. n., paratype female (OSUC 439664) **341** Head and mesosoma, lateral view **342** Head and mesosoma, dorsal view **343** Head, anterior view **344** Metasoma, dorsal view. Paratype male (OSUC 439677) **345** Antenna **346** Metasoma, dorsal view. Morphbank^92^

Figures 341–346 ; Morphbank^92^

##### Description.

Female. Body length 2.5–3.5 mm (n=20).

Radicle color and shade: same as scape, both yellowish or reddish. Pedicel color: same as scape. A3: shorter than pedicel. A4: broader than long. A5: broader than long.

Ventral clypeal margin: concave. Interantennal process: not elongate. Lower frons at dorsal margin of interantennal process: without transverse carina. Transverse curved rugae extending from frontal depression to eye: absent. Median longitudinal carina in frontal depression: absent. Ventral portion of frontal depression: smooth. Dorsal portion of frontal depression: without transverse carinae. Submedian carina: present. Frontal depression dorsally: hood-like, dorsally protruding. Upper frons major sculpture: umbilicate foveate. Upper frons microsculpture: absent. Hyperoccipital carina: indicated by a set of irregular elevations. Carina connecting occipital carina to hyperoccipital carina: absent. Occipital carina: omicron-shaped, with sharp corners where median portion meets lateral portions. Occiput sculpture: transversely rugose. Extra carina ventral to occipital carina: absent. Gena length: shorter than eye. Major sculpture of gena anteroventrally: umbilicate foveate. Major sculpture of gena posteroventrally: umbilicate foveate. Microsculpture of gena anteroventrally: absent. Microsculpture of gena posteroventrally: absent.

Lateral pronotal area sculpture: anteriorly smooth, posterodorsal corner with dense microsculpture, ventral corner with irregular carinae. Posterior border of central pronotal area: directed anteriorly, protruding at corner of epomial carina and transverse pronotal carina. Mesoscutum anteriorly: very steep and tall, descending at a right angle or protruding anteriorly. Major sculpture of mesoscutal midlobe anteriorly: umbilicate foveate. Mesoscutal midlobe sculpture at midlength: not different from nearby sculpture. Major sculpture of mesoscutal midlobe posteriorly: umbilicate foveate. Microsculpture of mesoscutal midlobe anteriorly: absent. Microsculpture of mesoscutal midlobe posteriorly: absent. Median mesoscutal carina: present as a vague, occasionally interrupted elevation. Major sculpture of mesoscutellum centrally: absent; umbilicate foveate. Major sculpture of mesoscutellum peripherally: umbilicate foveate. Microsculpture of mesoscutellum centrally: absent. Microsculpture of mesoscutellum peripherally: absent; punctate. Mesoscutellar rim: not expanded. Mesoscutellar rim medially: without notch. Mesofemoral depression: longitudinally striate dorsally, smooth ventrally. Metascutellum shape: deeply emarginate, with the resulting pair of posterior processes subtriangular and directed dorsally. Metascutellar setae: absent. Metascutellum sculpture: with large smooth posterior fovea. Postmarginal vein: absent. Fore wing apex at rest: exceeding metasomal apex. Coxae color brightness: same color as femora. Spines along tibiae: present. Lateral propodeal carinae: broadly separated, but parallel for a short distance anteriorly. Setae in metasomal depression: absent. Anterior sculpture of metasomal depression: absent. Median propodeal carina: absent.

T1 horn: absent. Number of longitudinal carinae of T1 midlobe: 4. T1 lateral carina: protruding laterally, visible from ventral view. T2 sculpture: with longitudinal striae or rugae, setiferous puncta present between them. T2 sublateral longitudinal foveae: absent. T3 metasomal flanges: absent. T4 sculpture: longitudinally striate to rugose, setal pits spanning interspaces. T4 metasomal flanges: absent. T5 sculpture: longitudinally striate to rugose, setal pits spanning interspaces. T5 metasomal flanges: present as slightly protruding sharp corners. T6: broader than long. Major sculpture of T6: umbilicate punctate. Microsculpture of T6: absent. T6 medially: with medially truncate emargination, sloping down to apical rim. T6 metasomal flanges: absent. T6 raised peripheral rim: absent. S4 sculpture: longitudinally striate or rugose, setal pits spanning interspaces. S5 sculpture: longitudinally striate to rugose, setal pits spanning interspaces. S5 median carina: absent. S6 peripheral carina: absent. S6 apex in relation to T6: not exposed to dorsal view. S6 apex: rounded or acuminate.

*Male*. Body length 2.8–3.45 mm (n=16). A3: longer than pedicel. A5 tyloid shape: narrow, linear. A6: broader than long. A11: broader than long; as long as broad. Major sculpture of mesoscutal midlobe anteriorly: umbilicate foveate. Major sculpture of mesoscutal midlobe posteriorly: umbilicate foveate; longitudinally rugose. Microsculpture of mesoscutal midlobe anteriorly: granulate. Microsculpture of mesoscutal midlobe posteriorly: absent. Major sculpture of mesoscutellum centrally: umbilicate foveate. Major sculpture of mesoscutellum peripherally: umbilicate foveate. Microsculpture of mesoscutellum centrally: absent. Microsculpture of mesoscutellum peripherally: absent. Fore wing apex at rest: exceeding metasomal apex; reaching middle of T6. T1 midlobe longitudinal carinae: 4. T3 metasomal flanges: absent. T4 metasomal flanges: absent. T5 metasomal flanges: absent. T6 metasomal flanges: present as sharp corners that do not protrude. T7: with a pair of sharply defined spine-like posterolateral projections.

##### Diagnosis.

Both sexes: Mesoscutum and mesoscutellum black. Frontal depression deep, transverse carinae absent; submedian carina strong and sharp. Hyperoccipital carina indicated by strong rugae. Occipital carina complete, sinuate medially. Metascutellum concave, nearly square, slightly emarginate apically, projecting dorsally. Coxa not darker than rest of leg. Tibiae with spines. T1 lateral carina expanded laterally. Metasomal flanges absent. Female: A3 shorter than pedicel. A4, A5 much broader than long. T1 midlobe with 4 longitudinal carinae. T6 without metasomal flanges, blunt or slightly concave apically. Fore wing long enough to reach middle of T6 or exceed metasomal apex. Male: A4, A11 broader than long. T1 midlobe with 4 longitudinal carinae. Fore wing long enough to exceed metasomal apex. T7 with sharp, broad, flat posterior projections.

##### Etymology.

Latin noun in apposition, meaning “the dirty.”

##### Link to distribution map.

[http://hol.osu.edu/map-full.html?id=307110]

##### Material examined.

Holotype, female: AUSTRALIA: QLD, via Taroom, FIT 035, Boggomoss No. 8, 25°27'S, 150°02'E, 14-XI-1996 - I-1997, flight intercept trap, Cook & Monteith, OSUC 439667 (deposited in QMBA). Paratypes: AUSTRALIA: 25 females, 17 males, OSUC 436935, 439655-439661, 439675, 448599 (ANIC); OSUC 448935 (NMNH); OSUC 448939, QDPC 0-163072, QDPC 0-165628 (QDPC); OSUC 148361, 148472, 439662-439666, 439668-439672, 439676-439679 (QMBA); OSUC 448598 (QPIM); OSUC 448933 (UQIC); OSUC 439673, 448928-448932, 448934, 448936-448938 (WINC).

##### Comments.

*Oxyscelio sordes* is one of a few species of *Oxyscelio* with tibial spines. These may be difficult to see, as they are the same color as the tibia. It can also be recognized by the strong frontal depression, unusually sparse sculpture of females, and flattened T7 projections in males.

#### Oxyscelio spatulae

Burks sp. n.

http://zoobank.org/23C83172-E17D-4ABC-B42E-7345D43DE483

urn:lsid:biosci.ohio-state.edu:osuc_concepts:275540

http://species-id.net/wiki/Oxyscelio_spatulae

**Figures 347–350.**
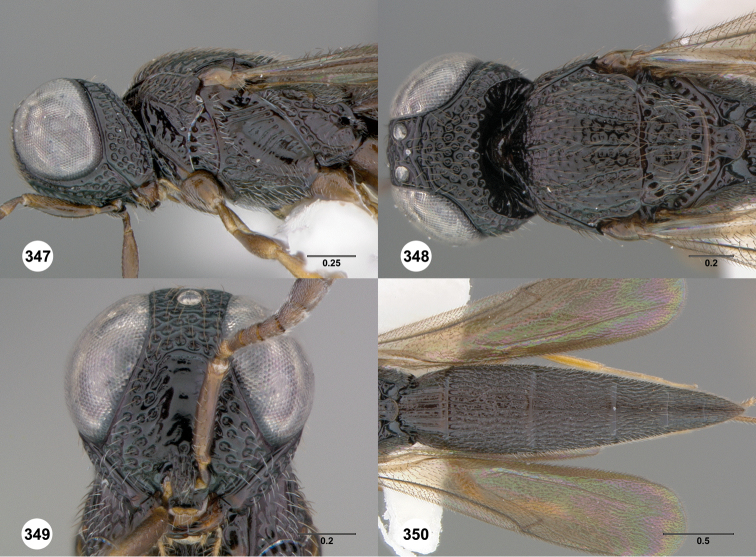
*Oxyscelio spatulae* sp. n., holotype female (OSUC 368914) **347** Head and mesosoma, lateral view **348** Head and mesosoma, dorsal view **349** Head, anterior view **350** Metasoma, dorsal view. Morphbank^93^

Figures 347–350 ; Morphbank^93^

##### Description.

Female. Body length 4.2 mm (n=1).

Radicle color and shade: same as scape, both dark brown. Pedicel color: same as scape. A3: longer than pedicel. A4: longer than broad. A5: broader than long.

Ventral clypeal margin: with slightly convex median lobe. Interantennal process: not elongate. Lower frons at dorsal margin of interantennal process: without transverse carina. Transverse curved rugae extending from frontal depression to eye: absent. Median longitudinal carina in frontal depression: absent. Ventral portion of frontal depression: with medially interrupted transverse carinae. Dorsal portion of frontal depression: with medially interrupted transverse carinae. Submedian carina: present only as a weak shift in elevation. Frontal depression dorsally: not hood-like, open dorsally. Upper frons major sculpture: umbilicate foveate; transversely rugose. Upper frons microsculpture: absent. Hyperoccipital carina: indicated by a set of irregular elevations. Carina connecting occipital carina to hyperoccipital carina: absent. Occipital carina: present laterally, absent medially. Occiput sculpture: umbilicate foveate. Extra carina ventral to occipital carina: absent. Gena length: shorter than eye. Major sculpture of gena anteroventrally: umbilicate foveate. Major sculpture of gena posteroventrally: umbilicate foveate; rugose. Microsculpture of gena anteroventrally: absent. Microsculpture of gena posteroventrally: absent.

Lateral pronotal area sculpture: with shallow irregular carinae, posterodorsal corner with dense microsculpture. Posterior border of central pronotal area: directed posteriorly, epomial carina absent or meeting transverse pronotal carina at arch on lateral surface of pronotum. Mesoscutum anteriorly: not steep, forming less than a right angle. Major sculpture of mesoscutal midlobe anteriorly: umbilicate foveate; transversely rugose. Mesoscutal midlobe sculpture at midlength: not different from nearby sculpture. Major sculpture of mesoscutal midlobe posteriorly: umbilicate foveate. Microsculpture of mesoscutal midlobe anteriorly: granulate. Microsculpture of mesoscutal midlobe posteriorly: absent. Median mesoscutal carina: present as a flattened or rounded elevation. Major sculpture of mesoscutellum centrally: umbilicate foveate; irregularly rugose. Major sculpture of mesoscutellum peripherally: umbilicate foveate. Microsculpture of mesoscutellum centrally: granulate. Microsculpture of mesoscutellum peripherally: granulate. Mesoscutellar rim: not expanded. Mesoscutellar rim medially: without notch. Mesofemoral depression: longitudinally striate dorsally, smooth ventrally. Metascutellum shape: not emarginate, forming a flat, concave shelf. Metascutellar setae: absent. Metascutellum sculpture: with a median carina ending in a broad granulate patch posteriorly. Postmarginal vein: present. Fore wing apex at rest: reaching middle of T5. Coxae color brightness: same color as femora. Spines along tibiae: absent. Lateral propodeal carinae: broadly separated, not parallel anteriorly. Setae in metasomal depression: unknown. Anterior sculpture of metasomal depression: unknown. Median propodeal carina: unknown.

T1 horn: present. Number of longitudinal carinae of T1 midlobe: obscured by other raised sculpture. T1 lateral carina: straight. T2 sculpture: with longitudinal striae or rugae, setiferous puncta present between them. T2 sublateral longitudinal foveae: absent. T3 metasomal flanges: absent. T4 sculpture: longitudinally striate to rugose, setal pits spanning interspaces. T4 metasomal flanges: absent. T5 sculpture: longitudinally striate to rugose, setal pits spanning interspaces. T5 metasomal flanges: absent. T6: longer than broad. Major sculpture of T6: umbilicate punctate. Microsculpture of T6: granulate. T6 medially: flat and tapering to a rounded apex, not separated from apical rim. T6 metasomal flanges: absent. T6 raised peripheral rim: absent. S4 sculpture: longitudinally striate or rugose, setal pits spanning interspaces. S5 sculpture: longitudinally striate to rugose, setal pits spanning interspaces. S5 median carina: present. S6 peripheral carina: absent. S6 apex in relation to T6: not exposed to dorsal view. S6 apex: rounded or acuminate.

*Male*. unknown.

##### Diagnosis.

Female: A3 longer than pedicel. Submedian carina weakly indicated by a rounded carina. Hyperoccipital carina indicated by rugae; occipital carina incomplete medially. Mesoscutellum with weak granulate sculpture. Metascutellum spatulate, with a median carina that ends in a broad granulate patch. T1 with moderate anterior horn. T2 without longitudinal foveae or dense striae. T6 longer than broad.

##### Etymology.

Latin noun, genitive case, meaning “broad flat structure.”

##### Link to distribution map.

[http://hol.osu.edu/map-full.html?id=275540]

##### Material examined.

Holotype, female: PAPUA NEW GUINEA: West New Britain Prov., New Britain Isl., primary forest edge, Cape Hoskins, 21.VI-27.VI.1973, malaise trap, Stibick, OSUC 368914 (deposited in CNCI).

##### Comments.

*Oxyscelio spatulae* is unusual in having a mildly spatulate metascutellum with a faint median carina that ends in a granulate area. It otherwise has few distinctive features, but is very different from *Oxyscelio nasi*, the other known species of *Oxyscelio* from New Britain.

#### Oxyscelio stipulae

Burks sp. n.

http://zoobank.org/9129186A-29EB-425B-AB3F-1C3F6AEAD367

urn:lsid:biosci.ohio-state.edu:osuc_concepts:307111

http://species-id.net/wiki/Oxyscelio_stipulae

**Figures 351–354.**
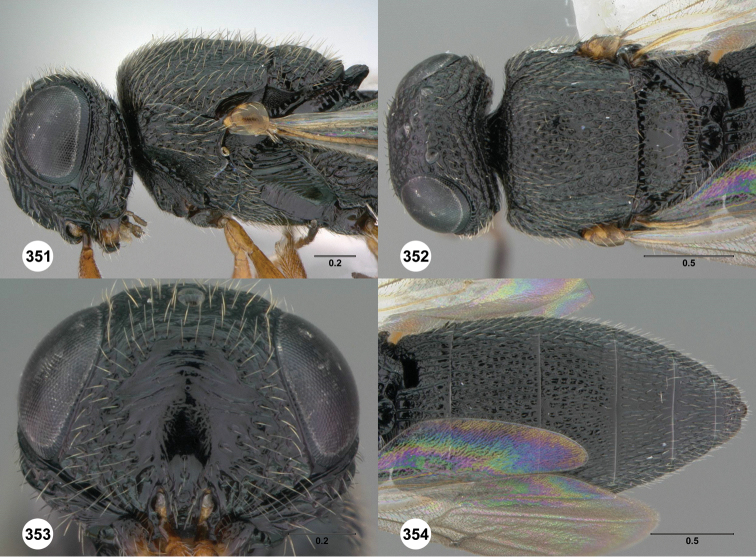
*Oxyscelio stipulae* sp. n., holotype female (OSUC 439590) **351** Head and mesosoma, lateral view **352** Head and mesosoma, dorsal view **353** Head, anterior view **354** Metasoma, dorsal view. Morphbank^94^

Figures 351–354 ; Morphbank^94^

##### Description.

Female. Body length 4.05–4.2 mm (n=2).

Radicle color and shade: darker than scape. Pedicel color: at least partially darker than scape. A3: longer than pedicel. A4: broader than long. A5: broader than long.

Ventral clypeal margin: with slightly convex median lobe. Interantennal process: not elongate. Lower frons at dorsal margin of interantennal process: without transverse carina. Transverse curved rugae extending from frontal depression to eye: absent. Median longitudinal carina in frontal depression: absent. Ventral portion of frontal depression: smooth. Dorsal portion of frontal depression: with medially interrupted transverse carinae. Submedian carina: present only as a weak shift in elevation. Frontal depression dorsally: not hood-like, open dorsally. Upper frons major sculpture: umbilicate foveate; irregularly rugose. Upper frons microsculpture: granulate. Hyperoccipital carina: absent. Carina connecting occipital carina to hyperoccipital carina: absent. Occipital carina: broadly angular, with rounded median peak. Occiput sculpture: umbilicate foveate. Extra carina ventral to occipital carina: present, medially incomplete. Gena length: shorter than eye. Major sculpture of gena anteroventrally: umbilicate foveate; rugose. Major sculpture of gena posteroventrally: umbilicate foveate; rugose. Microsculpture of gena anteroventrally: absent. Microsculpture of gena posteroventrally: absent.

Lateral pronotal area sculpture: anteriorly smooth, posterodorsal corner with dense microsculpture, ventral corner with irregular carinae. Posterior border of central pronotal area: directed anteriorly, protruding at corner of epomial carina and transverse pronotal carina. Mesoscutum anteriorly: very steep and tall, descending at a right angle or protruding anteriorly. Major sculpture of mesoscutal midlobe anteriorly: umbilicate foveate. Mesoscutal midlobe sculpture at midlength: not different from nearby sculpture. Major sculpture of mesoscutal midlobe posteriorly: umbilicate foveate; longitudinally rugose. Microsculpture of mesoscutal midlobe anteriorly: granulate. Microsculpture of mesoscutal midlobe posteriorly: granulate. Median mesoscutal carina: absent. Major sculpture of mesoscutellum centrally: absent. Major sculpture of mesoscutellum peripherally: umbilicate foveate. Microsculpture of mesoscutellum centrally: absent. Microsculpture of mesoscutellum peripherally: absent. Mesoscutellar rim: not expanded. Mesoscutellar rim medially: with notch. Mesofemoral depression: longitudinally striate dorsally, smooth ventrally. Metascutellum shape: slightly emarginate posteriorly, concave but elevated posteriorly. Metascutellar setae: absent. Metascutellum sculpture: with large smooth posterior fovea. Postmarginal vein: absent. Fore wing apex at rest: reaching middle of T5. Coxae color brightness: darker than femora. Spines along tibiae: absent. Lateral propodeal carinae: broadly separated, not parallel anteriorly. Setae in metasomal depression: absent. Anterior sculpture of metasomal depression: absent. Median propodeal carina: absent.

T1 horn: absent. Number of longitudinal carinae of T1 midlobe: 4. T1 lateral carina: protruding laterally, visible from ventral view. T2 sculpture: with longitudinal striae or rugae, setiferous puncta present between them. T2 sublateral longitudinal foveae: absent. T3 metasomal flanges: absent. T4 sculpture: longitudinally striate to rugose, setal pits spanning interspaces. T4 metasomal flanges: absent. T5 sculpture: longitudinally striate to rugose, setal pits spanning interspaces. T5 metasomal flanges: absent. T6: broader than long. Major sculpture of T6: longitudinally striate; umbilicate foveate. Microsculpture of T6: absent. T6 medially: with medially truncate emargination, sloping down to apical rim. T6 metasomal flanges: present as slightly expanded lateral rims, truncate posteriorly. T6 raised peripheral rim: absent. S4 sculpture: longitudinally striate or rugose, setal pits spanning interspaces. S5 sculpture: longitudinally striate to rugose, setal pits spanning interspaces. S5 median carina: present. S6 peripheral carina: absent. S6 apex in relation to T6: not exposed to dorsal view. S6 apex: rounded or acuminate.

*Male*. unknown.

##### Diagnosis.

Both sexes: Frontal depression deep, without carinae ventrally, dorsal separator complete, some complete carinae present above dorsal separator; submedian carina very weak. Hyperoccipital carina absent. Occipital carina complete, medially sinuate. Mesoscutellar rim not expanded, with median notch. Metascutellum broad, weakly emarginate apically, projecting dorsally. Coxa darker than rest of leg. Postmarginal vein absent. Female: A4, A5 broader than long. T4, T5 without metasomal flanges. T6 with narrowly expanded lateral margins. Main surface of T6 weakly emarginate medially, not sloping down to apical rim. S6 not exposed to dorsal view.

##### Etymology.

Latin noun, genitive case, meaning “stubble.”

##### Link to distribution map.

[http://hol.osu.edu/map-full.html?id=307111]

##### Material examined.

Holotype, female: AUSTRALIA: SA, Belair National Park, 4.II–11.II.1996, malaise trap, J. T. Jennings, OSUC 439590 (deposited in SAMA). Paratype: AUSTRALIA: 1 female, OSUC 148617 (SAMA).

#### Oxyscelio stringerae

Burks sp. n.

http://zoobank.org/F5C89E8C-6C5A-4D32-95E2-CF33CA4C804B

urn:lsid:biosci.ohio-state.edu:osuc_concepts:307112

http://species-id.net/wiki/Oxyscelio_stringerae

**Figures 355–358.**
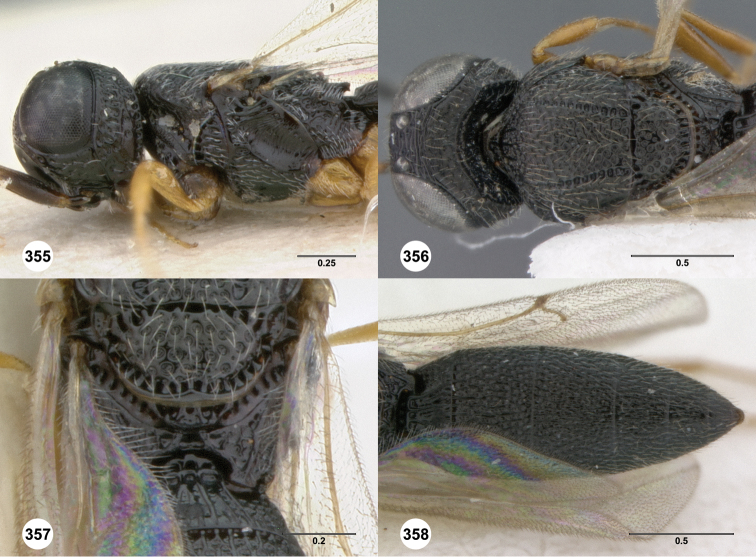
*Oxyscelio stringerae* sp. n., holotype female (OSUC 148369) **355** Head and mesosoma, lateral view **356** Head and mesosoma, dorsal view. Paratype female (368219) **357** Propodeum, posterodorsal view **358** Metasoma, dorsal view. Morphbank^95^

Figures 355–358 ; Morphbank^95^

##### Description.

Female. Body length 3.25–3.9 mm (n=6).

Radicle color and shade: darker than scape. Pedicel color: same as scape. A3: longer than pedicel. A4: broader than long. A5: broader than long.

Ventral clypeal margin: concave. Interantennal process: not elongate. Lower frons at dorsal margin of interantennal process: without transverse carina. Transverse curved rugae extending from frontal depression to eye: absent. Median longitudinal carina in frontal depression: absent. Ventral portion of frontal depression: with medially interrupted transverse carinae. Dorsal portion of frontal depression: without transverse carinae. Submedian carina: present. Frontal depression dorsally: not hood-like, open dorsally. Upper frons major sculpture: umbilicate foveate; irregularly rugose. Upper frons microsculpture: absent. Hyperoccipital carina: indicated by a set of irregular elevations. Carina connecting occipital carina to hyperoccipital carina: present. Occipital carina: omicron-shaped, with sharp corners where median portion meets lateral portions. Occiput sculpture: irregularly sculptured. Extra carina ventral to occipital carina: present, medially incomplete. Gena length: shorter than eye. Major sculpture of gena anteroventrally: umbilicate foveate. Major sculpture of gena posteroventrally: umbilicate punctate. Microsculpture of gena anteroventrally: absent. Microsculpture of gena posteroventrally: absent; granulate.

Lateral pronotal area sculpture: anteriorly smooth, posterodorsal corner with dense microsculpture, ventral corner with irregular carinae. Posterior border of central pronotal area: directed posteriorly, epomial carina absent or meeting transverse pronotal carina at arch on lateral surface of pronotum. Mesoscutum anteriorly: not steep, forming less than a right angle. Major sculpture of mesoscutal midlobe anteriorly: umbilicate foveate. Mesoscutal midlobe sculpture at midlength: not different from nearby sculpture. Major sculpture of mesoscutal midlobe posteriorly: umbilicate foveate. Microsculpture of mesoscutal midlobe anteriorly: granulate. Microsculpture of mesoscutal midlobe posteriorly: absent. Median mesoscutal carina: present as a vague, occasionally interrupted elevation. Major sculpture of mesoscutellum centrally: umbilicate foveate. Major sculpture of mesoscutellum peripherally: umbilicate foveate. Microsculpture of mesoscutellum centrally: absent. Microsculpture of mesoscutellum peripherally: absent. Mesoscutellar rim: not expanded. Mesoscutellar rim medially: without notch. Mesofemoral depression: longitudinally striate dorsally and ventrally. Metascutellum shape: not emarginate, concave but elevated posteriorly. Metascutellar setae: absent. Metascutellum sculpture: with large smooth posterior fovea. Postmarginal vein: present. Fore wing apex at rest: reaching middle of T6. Coxae color brightness: same color as femora. Spines along tibiae: absent. Lateral propodeal carinae: narrowly separated, angled anteriorly to become parallel. Setae in metasomal depression: absent. Anterior sculpture of metasomal depression: with median areole or pair of pits. Median propodeal carina: present.

T1 horn: absent. Number of longitudinal carinae of T1 midlobe: 4. T1 lateral carina: straight. T2 sculpture: densely foveolate, longitudinal sculpture irregular. T2 sublateral longitudinal foveae: absent. T3 metasomal flanges: absent. T4 sculpture: densely foveate, longitudinal sculpture irregular. T4 metasomal flanges: absent. T5 sculpture: longitudinally striate to rugose, setal pits spanning interspaces. T5 metasomal flanges: absent. T6: broader than long. Major sculpture of T6: umbilicate punctate. Microsculpture of T6: granulate. T6 medially: flat and tapering to a rounded apex, not separated from apical rim. T6 metasomal flanges: absent. T6 raised peripheral rim: absent. S4 sculpture: longitudinally striate or rugose, setal pits spanning interspaces. S5 sculpture: longitudinally striate to rugose, setal pits spanning interspaces. S5 median carina: present. S6 peripheral carina: absent. S6 apex in relation to T6: not exposed to dorsal view. S6 apex: rounded or acuminate.

*Male*. unknown.

##### Diagnosis.

Both sexes: Frontal depression deep, transverse carinae absent; submedian carina strong. Hyperoccipital carina indicated by rugae. Occipital carina complete, laterally with strong corners and medially broadly sinuate. Metascutellum short, concave, rugose. Postmarginal vein present. Coxa not darker than rest of leg. Metasomal depression with a pair of submedian foveae. T1 lateral carina not expanded laterally. Metasomal flanges absent. Female only: A4 about as long as broad, A5 broader than long. Mesoscutellum without granulate sculpture. T1 midlobe with 4 longitudinal carinae. Fore wing long enough to reach T6. T6 broader than long. *Oxyscelio stringerae* is similar to *Oxyscelio sinuationis*, but has a shorter and more broadly curved occipital carina, a broader metascutellum, only a pair of submedian foveae in the metasomal depression anteriorly, a slightly shorter flagellum, and 4 T1 midlobe carinae.

##### Etymology.

Named in honor of Danielle Stringer, for the large amount of excellent work that she contributed in packing and shipping specimens for this project.

##### Link to distribution map.

[http://hol.osu.edu/map-full.html?id=307112]

##### Material examined.

Holotype, female: AUSTRALIA: QLD, Mount Moffatt Section, Carnarvon National Park, creek bed, Peawaddy Gorge, 24°55’S, 148°04’E, 27.II.1996, Pyrethrum knockdown, G. Monteith, OSUC 148369 (deposited in QMBA). Paratypes: AUSTRALIA: 5 females, OSUC 368219-368220, 437010-437011, 448632 (WINC).

#### Oxyscelio tenuitatis

Burks sp. n.

http://zoobank.org/B82EF96C-90F3-4C7F-86E8-BAF19435493E

urn:lsid:biosci.ohio-state.edu:osuc_concepts:307113

http://species-id.net/wiki/Oxyscelio_tenuitatis

**Figures 359–364.**
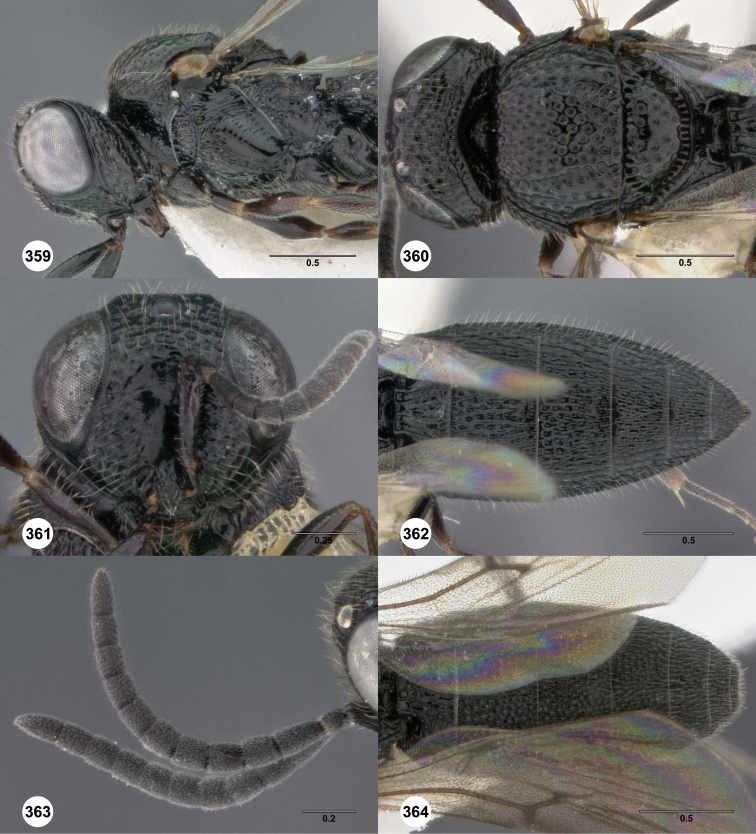
*Oxyscelio tenuitatis* sp. n., paratype female (OSUC 438285) **359** Head and mesosoma, lateral view. Holotype female (OSUC 438303) **360** Head and mesosoma, dorsal view **361** Head, anterior view **362** Metasoma, dorsal view. Paratype male (OSUC 438340) **363** Antenna **364** Metasoma, dorsal view. Morphbank^96^

Figures 359–364 ; Morphbank^96^

##### Description.

Female. Body length 3.3–4 mm (n=20).

Radicle color and shade: same as scape, both dark brown. Pedicel color: same as scape. A3: longer than pedicel. A4: broader than long. A5: broader than long.

Ventral clypeal margin: with slightly convex median lobe. Interantennal process: not elongate. Lower frons at dorsal margin of interantennal process: without transverse carina. Transverse curved rugae extending from frontal depression to eye: absent. Median longitudinal carina in frontal depression: absent. Ventral portion of frontal depression: smooth. Dorsal portion of frontal depression: with medially interrupted transverse carinae. Submedian carina: present only as a weak shift in elevation. Frontal depression dorsally: not hood-like, open dorsally. Upper frons major sculpture: umbilicate foveate; transversely rugose. Upper frons microsculpture: absent. Hyperoccipital carina: absent. Carina connecting occipital carina to hyperoccipital carina: absent. Occipital carina: weakly arched dorsally, with rounded lateral corners. Occiput sculpture: umbilicate foveate. Extra carina ventral to occipital carina: absent. Gena length: shorter than eye. Major sculpture of gena anteroventrally: umbilicate foveate. Major sculpture of gena posteroventrally: umbilicate foveate; rugose. Microsculpture of gena anteroventrally: absent. Microsculpture of gena posteroventrally: absent.

Lateral pronotal area sculpture: anteriorly smooth, posterodorsal corner with dense microsculpture, ventral corner with irregular carinae. Posterior border of central pronotal area: directed anteriorly, protruding at corner of epomial carina and transverse pronotal carina. Mesoscutum anteriorly: very steep and tall, descending at a right angle or protruding anteriorly. Major sculpture of mesoscutal midlobe anteriorly: umbilicate foveate. Mesoscutal midlobe sculpture at midlength: not different from nearby sculpture. Major sculpture of mesoscutal midlobe posteriorly: umbilicate foveate. Microsculpture of mesoscutal midlobe anteriorly: granulate. Microsculpture of mesoscutal midlobe posteriorly: absent. Median mesoscutal carina: present as a vague, occasionally interrupted elevation. Major sculpture of mesoscutellum centrally: absent; umbilicate foveate. Major sculpture of mesoscutellum peripherally: umbilicate foveate. Microsculpture of mesoscutellum centrally: absent. Microsculpture of mesoscutellum peripherally: absent. Mesoscutellar rim: not expanded. Mesoscutellar rim medially: without notch. Mesofemoral depression: longitudinally striate dorsally, smooth ventrally. Metascutellum shape: slightly emarginate posteriorly, concave but elevated posteriorly. Metascutellar setae: absent. Metascutellum sculpture: with large smooth posterior fovea. Postmarginal vein: present. Fore wing apex at rest: reaching middle of T6. Coxae color brightness: same color as femora. Spines along tibiae: absent. Lateral propodeal carinae: broadly separated, not parallel anteriorly. Setae in metasomal depression: absent. Anterior sculpture of metasomal depression: absent. Median propodeal carina: absent.

T1 horn: absent. Number of longitudinal carinae of T1 midlobe: obscured by other raised sculpture. T1 lateral carina: protruding laterally, visible from ventral view. T2 sculpture: with longitudinal striae or rugae, setiferous puncta present between them. T2 sublateral longitudinal foveae: absent. T3 metasomal flanges: absent. T4 sculpture: longitudinally striate to rugose, setal pits spanning interspaces. T4 metasomal flanges: absent. T5 sculpture: longitudinally striate to rugose, setal pits spanning interspaces. T5 metasomal flanges: absent. T6: broader than long. Major sculpture of T6: umbilicate punctate. Microsculpture of T6: absent. T6 medially: flat and tapering to a rounded apex, not separated from apical rim. T6 metasomal flanges: absent. T6 raised peripheral rim: absent. S4 sculpture: longitudinally striate or rugose, setal pits spanning interspaces. S5 sculpture: longitudinally striate to rugose, setal pits spanning interspaces. S5 median carina: present. S6 peripheral carina: absent. S6 apex in relation to T6: not exposed to dorsal view. S6 apex: rounded or acuminate.

*Male*. Body length 3.25–3.85 mm (n=). A3: longer than pedicel. A5 tyloid shape: narrow, linear. A6: longer than broad. A11: longer than broad. Major sculpture of mesoscutal midlobe anteriorly: umbilicate foveate. Major sculpture of mesoscutal midlobe posteriorly: umbilicate foveate; longitudinally rugose. Microsculpture of mesoscutal midlobe anteriorly: granulate. Microsculpture of mesoscutal midlobe posteriorly: absent. Major sculpture of mesoscutellum centrally: umbilicate foveate; absent. Major sculpture of mesoscutellum peripherally: umbilicate foveate. Microsculpture of mesoscutellum centrally: absent. Microsculpture of mesoscutellum peripherally: absent. Fore wing apex at rest: reaching middle of T6. T1 midlobe longitudinal carinae: 4. T3 metasomal flanges: absent. T4 metasomal flanges: absent. T5 metasomal flanges: absent. T6 metasomal flanges: absent. T7: weakly emarginate.

##### Diagnosis.

Both sexes: Body entirely dark brown, including antennae and legs. Frontal depression shallow, transverse carinae absent or broadly interrupted ventrally, absent dorsally; submedian carina absent medially. Hyperoccipital carina absent. Occipital carina complete, weakly convex or forming a rounded peak medially. Metascutellum deeply concave, emarginate apically, projecting dorsally. Postmarginal vein absent. T1 lateral carina expanded laterally. Metasomal flanges absent. Female: A3 longer than pedicel. A4 as long or slightly longer than broad, A5 nearly as long as broad. Mesoscutellum without granulate sculpture. T1 midlobe with 4 longitudinal carinae. T6 without metasomal flanges, main body of tergum not abruptly separated from apical rim. Fore wing long enough to reach middle of T6. Male: All flagellomeres longer than broad. T1 midlobe with 4 longitudinal carinae. Fore wing long enough to reach to or beyond T7. T7 emarginate apically, with rounded posterior lobes. *Oxyscelio tenuitatis* is very similar to *Oxyscelio densitatis*, but differs in having dark brown antennae and legs, and in having weaker, more sparse sculpture on the metasomal sterna.

##### Etymology.

Latin noun, meaning “thinness.”

##### Link to distribution map.

[http://hol.osu.edu/map-full.html?id=307113]

##### Material examined.

Holotype, female: AUSTRALIA: TAS, Collinsvale, 12.II.1983, malaise trap, M. A. Williams, OSUC 438303 (deposited in ANIC). Paratypes: AUSTRALIA: 32 females, 54 males, Australian Museum K245265 (AMSA); ANIC DB 32-020902, 32-020903, OSUC 438282, OSUC 438283, OSUC 438284, OSUC 438285, OSUC 438286, OSUC 438287, OSUC 438288, OSUC 438289, OSUC 438290, OSUC 438291, OSUC 438292, OSUC 438293, OSUC 438294, OSUC 438295, OSUC 438296, OSUC 438297, OSUC 438298, OSUC 438299, OSUC 438300, OSUC 438301, OSUC 438311, OSUC 438312, OSUC 438313, OSUC 438314, OSUC 438315, OSUC 438316, OSUC 438317, OSUC 438318, OSUC 438319, OSUC 438320, OSUC 438321, OSUC 438322, OSUC 438324, OSUC 438325, OSUC 438326, OSUC 438327, OSUC 438328, OSUC 438329, OSUC 438330, OSUC 438331, OSUC 438332, OSUC 438333, OSUC 438334, OSUC 438335, OSUC 438336, OSUC 438337, OSUC 438338, OSUC 438339, OSUC 438340, OSUC 438341, OSUC 438343, OSUC 448574, OSUC 448575, OSUC 448576, OSUC 448577, OSUC 448578, OSUC 448579, OSUC 448580, OSUC 448581, OSUC 448582, OSUC 448583, OSUC 448584, OSUC 448585, OSUC 448586, OSUC 453952, OSUC 453953, OSUC 453954, OSUC 453955, OSUC 453956 (ANIC); OSUC 438345-438346 (MVMA); OSUC 438304-438310, 438323, 448587 (TDAH); OSUC 438302, 438344, 453957 (WINC).

##### Comments.

While *Oxyscelio tenuitatis* may be only a melanistic form of *Oxyscelio densitatis*, we are recognizing these are separate species because they do differ in minor but consistent ways.

#### Oxyscelio truncationis

Burks sp. n.

http://zoobank.org/214262FD-9E98-499D-A063-E9648A130DE1

urn:lsid:biosci.ohio-state.edu:osuc_concepts:307114

http://species-id.net/wiki/Oxyscelio_truncationis

**Figures 365–368.**
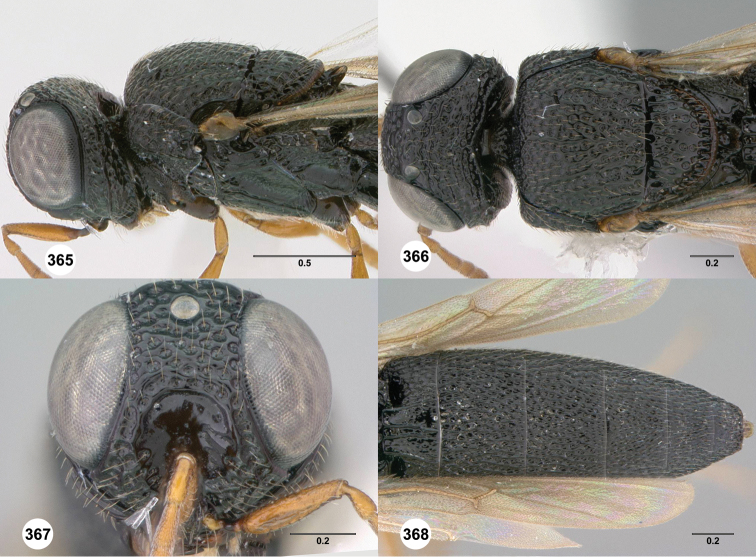
*Oxyscelio truncationis* sp. n., holotype female (OSUC 429933) **365** Head and mesosoma, lateral view **366** Head and mesosoma, dorsal view **367** Head, anterior view **368** Metasoma, dorsal view. Morphbank^97^

Figures 365–368 ; Morphbank^97^

##### Description.

Female. Body length 2.95–4 mm (n=20).

Radicle color and shade: darker than scape. Pedicel color: same as scape. A3: longer than pedicel. A4: broader than long; as long as broad. A5: broader than long.

Ventral clypeal margin: with slightly convex median lobe. Interantennal process: not elongate. Lower frons at dorsal margin of interantennal process: without transverse carina. Transverse curved rugae extending from frontal depression to eye: absent. Median longitudinal carina in frontal depression: absent. Ventral portion of frontal depression: with transverse carinae. Dorsal portion of frontal depression: without transverse carinae. Submedian carina: absent. Frontal depression dorsally: not hood-like, open dorsally. Upper frons major sculpture: umbilicate foveate; transversely rugose. Upper frons microsculpture: granulate. Hyperoccipital carina: indicated by a set of irregular elevations. Carina connecting occipital carina to hyperoccipital carina: absent. Occipital carina: broadly angular, with rounded median peak. Occiput sculpture: irregularly sculptured. Extra carina ventral to occipital carina: present, medially incomplete. Gena length: shorter than eye. Major sculpture of gena anteroventrally: umbilicate foveate. Major sculpture of gena posteroventrally: umbilicate foveate; rugose. Microsculpture of gena anteroventrally: absent. Microsculpture of gena posteroventrally: absent.

Lateral pronotal area sculpture: anteriorly smooth, posterodorsal corner with dense microsculpture, ventral corner with irregular carinae. Posterior border of central pronotal area: directed anteriorly, protruding at corner of epomial carina and transverse pronotal carina. Mesoscutum anteriorly: very steep and tall, descending at a right angle or protruding anteriorly. Major sculpture of mesoscutal midlobe anteriorly: umbilicate foveate. Mesoscutal midlobe sculpture at midlength: not different from nearby sculpture. Major sculpture of mesoscutal midlobe posteriorly: umbilicate foveate. Microsculpture of mesoscutal midlobe anteriorly: granulate. Microsculpture of mesoscutal midlobe posteriorly: absent. Median mesoscutal carina: present as a vague, occasionally interrupted elevation. Major sculpture of mesoscutellum centrally: umbilicate foveate. Major sculpture of mesoscutellum peripherally: umbilicate foveate. Microsculpture of mesoscutellum centrally: absent. Microsculpture of mesoscutellum peripherally: absent. Mesoscutellar rim: not expanded. Mesoscutellar rim medially: without notch. Mesofemoral depression: longitudinally striate dorsally, smooth ventrally. Metascutellum shape: slightly emarginate posteriorly, concave but elevated posteriorly. Metascutellar setae: absent. Metascutellum sculpture: with large smooth posterior fovea. Postmarginal vein: absent. Fore wing apex at rest: reaching middle of T5. Coxae color brightness: darker than femora. Spines along tibiae: absent. Lateral propodeal carinae: broadly separated, not parallel anteriorly. Setae in metasomal depression: absent. Anterior sculpture of metasomal depression: absent. Median propodeal carina: absent.

T1 horn: absent. Number of longitudinal carinae of T1 midlobe: 4. T1 lateral carina: protruding laterally, visible from ventral view. T2 sculpture: densely foveolate, longitudinal sculpture irregular. T2 sublateral longitudinal foveae: absent. T3 metasomal flanges: absent. T4 sculpture: longitudinally striate to rugose, setal pits spanning interspaces. T4 metasomal flanges: absent. T5 sculpture: longitudinally striate to rugose, setal pits spanning interspaces. T5 metasomal flanges: present as strong posterior corners. T6: broader than long. Major sculpture of T6: umbilicate punctate; longitudinally striate. Microsculpture of T6: absent. T6 medially: with medially truncate emargination, sloping down to apical rim. T6 metasomal flanges: absent. T6 raised peripheral rim: absent. S4 sculpture: longitudinally striate or rugose, setal pits spanning interspaces. S5 sculpture: longitudinally striate to rugose, setal pits spanning interspaces. S5 median carina: present. S6 peripheral carina: absent. S6 apex in relation to T6: not exposed to dorsal view. S6 apex: rounded or acuminate.

*Male*. unknown.

##### Diagnosis.

Both sexes: Frontal depression deep, carinae interrupted medially, no carinae above dorsal separator; submedian carina absent or weak and irregular. Hyperoccipital carina indicated by rugae. Occipital carina complete, sinuate medially. Mesoscutellar rim not expanded, without median notch. Metascutellum scoop-shaped, deeply concave, slightly emarginate apically, projecting dorsally. Coxa darker than rest of leg. T1 lateral carina expanded laterally. Female: A3 longer than pedicel. A4 as long or longer than broad, A5, A6 broader than long. T1 midlobe with 4 longitudinal midlobe carinae anteriorly or with dense sculpture obscuring them. Fore wing long enough to reach middle of T5. T4, T5 without metasomal flanges. Main body of T6 raised above apical rim and abruptly separated from it; lateral margins of T6 slightly expanded, forming a truncate or slightly emarginate shelf-like apex.

##### Etymology.

Latin noun, genitive case, meaning “a truncation.”

##### Link to distribution map.

[http://hol.osu.edu/map-full.html?id=307114]

##### Material examined.

Holotype, female: AUSTRALIA: QLD, 15km NE Mareeba, 20.XII–7.I.1985, Storey & Titmarsh, OSUC 429933 (deposited in QMBA). Paratypes: AUSTRALIA: 46 females, ANIC DB 32-020082, 32-020153, OSUC 359643, OSUC 429944, OSUC 429945, OSUC 429946, OSUC 429950, OSUC 429952, OSUC 429956, OSUC 429957, OSUC 429958, OSUC 429959, OSUC 429960, OSUC 429961, OSUC 453995 (ANIC); OSUC 148377, 429948-429949, 429955, QM Reg. No. T35151 (QMBA); OSUC 449090 (UQIC); OSUC 429922-429932, 429934-429943, 429947, 429951, 429953, 453994 (WINC).

#### Oxyscelio tubi

Burks sp. n.

http://zoobank.org/9B46017D-19DE-433E-AE7C-29A43F7094E9

urn:lsid:biosci.ohio-state.edu:osuc_concepts:307121

http://species-id.net/wiki/Oxyscelio_tubi

**Figures 369–374.**
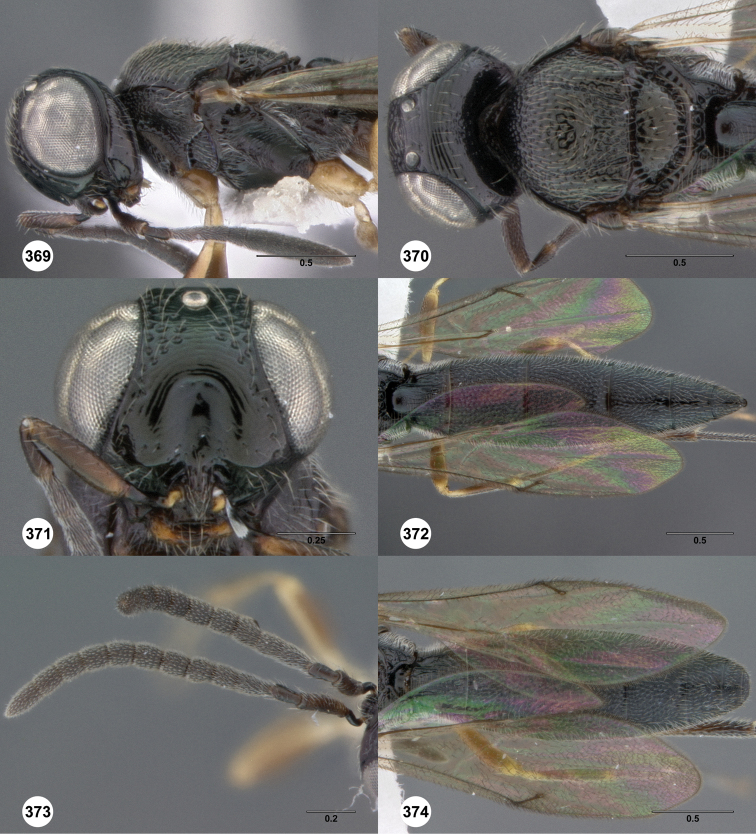
*Oxyscelio tubi* sp. n., holotype female (OSUC 225607) **369** Head and mesosoma, lateral view **370** Head and mesosoma, dorsal view **371** Head, anterior view **372** Metasoma, dorsal view. Paratype male (OSUC 221668) **373** Antenna. Paratype male (OSUC 221669) **374** Metasoma, dorsal view. Morphbank^98^

Figures 369–374 ; Morphbank^98^

##### Description.

Female. Body length 4.35–4.8 mm (n=2).

Radicle color and shade: darker than scape. Pedicel color: same as scape. A3: longer than pedicel. A4: longer than broad. A5: longer than broad.

Ventral clypeal margin: concave. Interantennal process: not elongate. Lower frons at dorsal margin of interantennal process: with transverse ledge, face sharply receding below it. Transverse curved rugae extending from frontal depression to eye: present. Median longitudinal carina in frontal depression: absent. Ventral portion of frontal depression: with medially interrupted transverse carinae. Dorsal portion of frontal depression: without transverse carinae. Submedian carina: present. Frontal depression dorsally: not hood-like, open dorsally. Upper frons major sculpture: umbilicate foveate; transversely rugose. Upper frons microsculpture: absent. Hyperoccipital carina: present as multiple regular elevations. Carina connecting occipital carina to hyperoccipital carina: absent. Occipital carina: present laterally, absent medially. Occiput sculpture: smooth; transversely rugose; umbilicate punctate. Extra carina ventral to occipital carina: absent. Gena length: shorter than eye. Major sculpture of gena anteroventrally: umbilicate punctate. Major sculpture of gena posteroventrally: umbilicate punctate. Microsculpture of gena anteroventrally: absent. Microsculpture of gena posteroventrally: absent.

Lateral pronotal area sculpture: anteriorly smooth, posterodorsal corner with dense microsculpture, ventral corner with irregular carinae. Posterior border of central pronotal area: directed posteriorly, epomial carina absent or meeting transverse pronotal carina at arch on lateral surface of pronotum. Mesoscutum anteriorly: very steep and tall, descending at a right angle or protruding anteriorly. Major sculpture of mesoscutal midlobe anteriorly: umbilicate punctate. Mesoscutal midlobe sculpture at midlength: not different from nearby sculpture. Major sculpture of mesoscutal midlobe posteriorly: umbilicate foveate; irregularly rugose. Microsculpture of mesoscutal midlobe anteriorly: absent; granulate. Microsculpture of mesoscutal midlobe posteriorly: absent. Median mesoscutal carina: absent. Major sculpture of mesoscutellum centrally: umbilicate punctate. Major sculpture of mesoscutellum peripherally: umbilicate punctate. Microsculpture of mesoscutellum centrally: absent. Microsculpture of mesoscutellum peripherally: absent. Mesoscutellar rim: not expanded. Mesoscutellar rim medially: without notch. Mesofemoral depression: longitudinally striate dorsally, smooth ventrally. Metascutellum shape: slightly emarginate posteriorly, concave but elevated posteriorly. Metascutellar setae: absent. Metascutellum sculpture: with a median carina, otherwise weakly sculptured. Postmarginal vein: present. Fore wing apex at rest: reaching middle of T5. Coxae color brightness: same color as femora. Spines along tibiae: absent. Lateral propodeal carinae: broadly separated, not parallel anteriorly. Setae in metasomal depression: absent. Anterior sculpture of metasomal depression: absent. Median propodeal carina: absent.

T1 horn: present. Number of longitudinal carinae of T1 midlobe: obscured by other raised sculpture. T1 lateral carina: straight. T2 sculpture: densely foveolate, longitudinal sculpture irregular. T2 sublateral longitudinal foveae: absent. T3 metasomal flanges: absent. T4 sculpture: longitudinally striate to rugose, setal pits spanning interspaces. T4 metasomal flanges: absent. T5 sculpture: longitudinally striate to rugose, setal pits spanning interspaces. T5 metasomal flanges: absent. T6: longer than broad. Major sculpture of T6: umbilicate punctate; longitudinally striate. Microsculpture of T6: absent. T6 medially: flat and tapering to a rounded apex, not separated from apical rim. T6 metasomal flanges: absent. T6 raised peripheral rim: absent. S4 sculpture: longitudinally striate or rugose, setal pits spanning interspaces. S5 sculpture: longitudinally striate to rugose, setal pits spanning interspaces. S5 median carina: absent. S6 peripheral carina: absent. S6 apex in relation to T6: not exposed to dorsal view. S6 apex: rounded or acuminate.

*Male*. Body length 3.65–3.9 mm (n=2). A3: longer than pedicel. A5 tyloid shape: narrow, linear. A6: broader than long. A11: longer than broad. Major sculpture of mesoscutal midlobe anteriorly: umbilicate foveate. Major sculpture of mesoscutal midlobe posteriorly: umbilicate foveate; irregularly rugose. Microsculpture of mesoscutal midlobe anteriorly: granulate. Microsculpture of mesoscutal midlobe posteriorly: granulate. Major sculpture of mesoscutellum centrally: umbilicate foveate. Major sculpture of mesoscutellum peripherally: umbilicate foveate. Microsculpture of mesoscutellum centrally: absent. Microsculpture of mesoscutellum peripherally: granulate. Fore wing apex at rest: reaching apex of T5. T1 midlobe longitudinal carinae: obscured by other raised sculpture. T3 metasomal flanges: absent. T4 metasomal flanges: absent. T5 metasomal flanges: absent. T6 metasomal flanges: absent. T7: weakly emarginate; truncate.

##### Diagnosis.

Both sexes: Frontal depression nearly flat, surrounded by many transverse curved rugae; submedian carina indicated by a set of rugae. Hyperoccipital carina indicated by many transverse carinae. Occipital carina incomplete, lateral portions approaching hyperoccipital carina. Occiput mostly smooth, with many rugae dorsally, otherwise smooth and with setiferous puncta. Metascutellum broad and very short, concave. Postmarginal vein present. Coxa and rest of leg bicolored. T1 lateral carina not expanded laterally. Metasomal flanges absent. Female: A3 longer than pedicel. A4, A5 longer than broad. Mesoscutellum with setiferous puncta. T1 midlobe with strong anterior horn, no complete longitudinal carinae. Fore wing long enough to reach to middle of T5. T6 longer than broad. Male: A4, A11 longer than broad. Mesoscutellum without extensive granulate sculpture. Fore wing long enough to reach apex of T5. T7 tiny, truncate or very weakly emarginate.

##### Etymology.

Latin noun, genitive case, meaning “tube.”

##### Link to distribution map.

[http://hol.osu.edu/map-full.html?id=307121]

##### Material examined.

Holotype, female: NEW CALEDONIA: Nord Prov., 5km WSW Pouebo, Mount Mandjelia, 20.397°S, 164.528°E, 780m, 9.XII–15.XII.2000, malaise trap, M. E. Irwin, OSUC 225607 (deposited in MNHN). Paratypes: NEW CALEDONIA: 1 female, 2 males, OSUC 185889, 221668-221669 (OSUC).

##### Comments.

*Oxyscelio tubi* is unique within the *flavipes*-group in having a very strong anterior T1 horn in females.

#### Oxyscelio umbonis

Burks sp. n.

http://zoobank.org/DA93B45D-9768-497F-BB65-A95DCC1706B6

urn:lsid:biosci.ohio-state.edu:osuc_concepts:307115

http://species-id.net/wiki/Oxyscelio_umbonis

**Figures 375–380.**
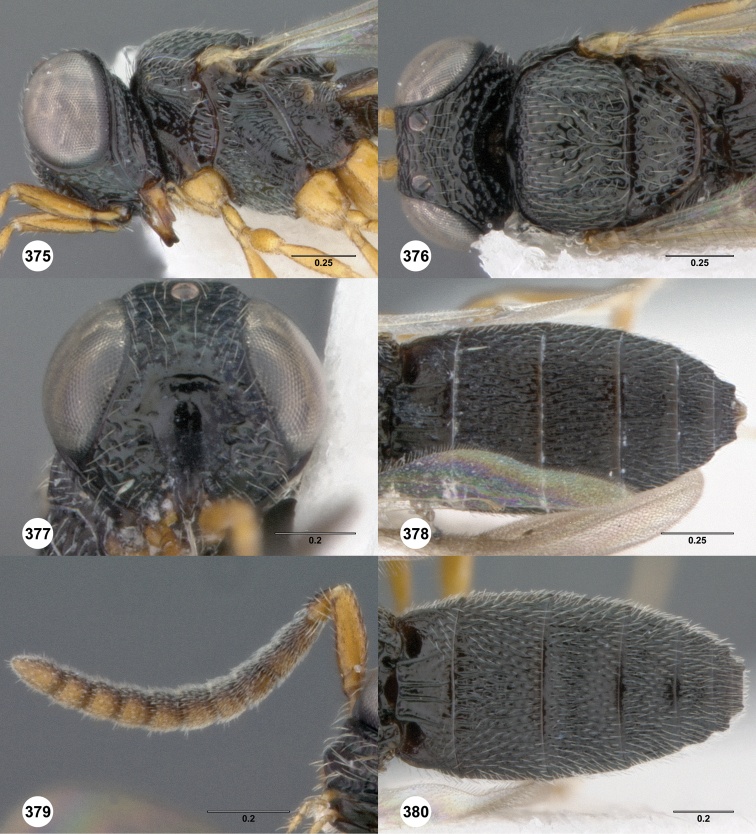
*Oxyscelio umbonis* sp. n., holotype female (OSUC 438236) **375** Head and mesosoma, lateral view **376** Head and mesosoma, dorsal view **377** Head, anterior view **378** Metasoma, dorsal view. Paratype male (OSUC 438271) **379** Antenna **380** Metasoma, dorsal view. Morphbank^99^

Figures 375–380 ; Morphbank^99^

##### Description.

Female. Body length 2.4–3.4 mm (n=20).

Radicle color and shade: same as scape, both yellowish or reddish. Pedicel color: same as scape; at least partially darker than scape. A3: shorter than pedicel. A4: broader than long. A5: broader than long.

Ventral clypeal margin: with slightly convex median lobe. Interantennal process: not elongate. Lower frons at dorsal margin of interantennal process: without transverse carina. Transverse curved rugae extending from frontal depression to eye: absent. Median longitudinal carina in frontal depression: absent. Ventral portion of frontal depression: smooth. Dorsal portion of frontal depression: without transverse carinae. Submedian carina: present only as a weak shift in elevation. Frontal depression dorsally: not hood-like, open dorsally. Upper frons major sculpture: umbilicate foveate; transversely rugose. Upper frons microsculpture: absent. Hyperoccipital carina: indicated by a set of irregular elevations. Carina connecting occipital carina to hyperoccipital carina: absent. Occipital carina: uniformly rounded dorsally. Occiput sculpture: umbilicate foveate. Extra carina ventral to occipital carina: absent. Gena length: shorter than eye. Major sculpture of gena anteroventrally: umbilicate foveate. Major sculpture of gena posteroventrally: umbilicate punctate; absent. Microsculpture of gena anteroventrally: absent. Microsculpture of gena posteroventrally: granulate.

Lateral pronotal area sculpture: with shallow irregular carinae, posterodorsal corner with dense microsculpture. Posterior border of central pronotal area: directed anteriorly, protruding at corner of epomial carina and transverse pronotal carina. Mesoscutum anteriorly: very steep and tall, descending at a right angle or protruding anteriorly. Major sculpture of mesoscutal midlobe anteriorly: umbilicate foveate. Mesoscutal midlobe sculpture at midlength: not different from nearby sculpture. Major sculpture of mesoscutal midlobe posteriorly: umbilicate foveate. Microsculpture of mesoscutal midlobe anteriorly: absent. Microsculpture of mesoscutal midlobe posteriorly: absent. Median mesoscutal carina: present as a vague, occasionally interrupted elevation. Major sculpture of mesoscutellum centrally: umbilicate foveate. Major sculpture of mesoscutellum peripherally: umbilicate foveate. Microsculpture of mesoscutellum centrally: absent. Microsculpture of mesoscutellum peripherally: absent. Mesoscutellar rim: not expanded. Mesoscutellar rim medially: without notch. Mesofemoral depression: longitudinally striate dorsally, smooth ventrally. Metascutellum shape: deeply emarginate, with the resulting pair of posterior processes subtriangular and directed dorsally. Metascutellar setae: absent. Metascutellum sculpture: with large smooth posterior fovea. Postmarginal vein: present. Fore wing apex at rest: exceeding metasomal apex; reaching middle of T6. Coxae color brightness: same color as femora. Spines along tibiae: absent. Lateral propodeal carinae: broadly separated, not parallel anteriorly. Setae in metasomal depression: absent. Anterior sculpture of metasomal depression: absent. Median propodeal carina: absent.

T1 horn: absent. Number of longitudinal carinae of T1 midlobe: 6. T1 lateral carina: protruding laterally, visible from ventral view. T2 sculpture: with longitudinal striae or rugae, setiferous puncta present between them. T2 sublateral longitudinal foveae: absent. T3 metasomal flanges: absent. T4 sculpture: longitudinally striate to rugose, setal pits spanning interspaces. T4 metasomal flanges: absent. T5 sculpture: longitudinally striate to rugose, setal pits spanning interspaces. T5 metasomal flanges: present as strong posterior corners. T6: broader than long. Major sculpture of T6: umbilicate punctate. Microsculpture of T6: absent. T6 medially: with broad emargination between protruding posterolateral corners, separated from apical rim. T6 metasomal flanges: present as spine-like structures posterolaterally. T6 raised peripheral rim: absent. S4 sculpture: densely setose, setal pits between very weak longitudinal rugae. S5 sculpture: densely setose, setal pits between very weak longitudinal rugae. S5 median carina: absent. S6 peripheral carina: absent. S6 apex in relation to T6: exposed to dorsal view by T6 emargination. S6 apex: rounded or acuminate.

*Male*. Body length 2.45–3.3 mm (n=20). A3: longer than pedicel. A5 tyloid shape: narrow, linear. A6: broader than long. A11: broader than long. Major sculpture of mesoscutal midlobe anteriorly: umbilicate foveate. Major sculpture of mesoscutal midlobe posteriorly: umbilicate foveate. Microsculpture of mesoscutal midlobe anteriorly: granulate. Microsculpture of mesoscutal midlobe posteriorly: absent; granulate. Major sculpture of mesoscutellum centrally: umbilicate foveate. Major sculpture of mesoscutellum peripherally: umbilicate foveate. Microsculpture of mesoscutellum centrally: absent. Microsculpture of mesoscutellum peripherally: absent. Fore wing apex at rest: exceeding metasomal apex. T1 midlobe longitudinal carinae: 4. T3 metasomal flanges: absent. T4 metasomal flanges: absent. T5 metasomal flanges: absent. T6 metasomal flanges: present as sharp corners that do not protrude. T7: with a pair of sharply defined spine-like posterolateral projections.

##### Diagnosis.

Mesoscutum and mesoscutellum black. Frontal depression shallow, transverse carinae absent; submedian carina absent or incomplete. Hyperoccipital carina indicated by strong rugae. Occipital carina complete, convex medially. Metascutellum deeply concave, projecting dorsally. T1 lateral carina expanded laterally. Female: A4, A5 broader than long. T1 midlobe with 6 or more longitudinal carinae, or these obscured by a smooth elevation. T5 with strong but rounded posterior corners. T6 abruptly narrower than T5, with sharp protruding posterior corners and a steep median slope leading to apical rim. Fore wing long enough to reach middle of T6 or beyond metasomal apex. Main body of T6 not abruptly separated from apical rim. Male: A3 not longer than pedicel. All flagellomeres between A4 and A12 broader than long. T1 midlobe with 4 longitudinal carinae. Fore wing long enough to exceed metasomal apex. T6 with strong posterior corners. T7 abruptly narrower than T6, with sharp, protruding posterior corners.

##### Etymology.

Latin noun, genitive case, meaning “a bump.”

##### Link to distribution map.

[http://hol.osu.edu/map-full.html?id=307115]

##### Material examined.

Holotype, female: AUSTRALIA: QLD, Heathlands, 11°45'S, 142°35'E, 25.VII–18.VIII.1992, malaise trap, P. Zborowski & J. Cardale, OSUC 438236 (deposited in ANIC). Paratypes: AUSTRALIA: 25 females, 77 males, ANIC DB 32-020147, 32-020150, OSUC 438187, OSUC 438188, OSUC 438189, OSUC 438190, OSUC 438191, OSUC 438192, OSUC 438193, OSUC 438194, OSUC 438195, OSUC 438196, OSUC 438197, OSUC 438198, OSUC 438199, OSUC 438200, OSUC 438201, OSUC 438202, OSUC 438203, OSUC 438204, OSUC 438205, OSUC 438206, OSUC 438207, OSUC 438208, OSUC 438209, OSUC 438210, OSUC 438211, OSUC 438212, OSUC 438213, OSUC 438214, OSUC 438215, OSUC 438216, OSUC 438217, OSUC 438218, OSUC 438219, OSUC 438220, OSUC 438221, OSUC 438222, OSUC 438223, OSUC 438224, OSUC 438225, OSUC 438226, OSUC 438227, OSUC 438229, OSUC 438231, OSUC 438232, OSUC 438233, OSUC 438234, OSUC 438235, OSUC 438237, OSUC 438238, OSUC 438239, OSUC 438240, OSUC 438241, OSUC 438242, OSUC 438243, OSUC 438244, OSUC 438245, OSUC 438246, OSUC 438247, OSUC 438248, OSUC 438249, OSUC 438250, OSUC 438251, OSUC 438252, OSUC 438253, OSUC 438254, OSUC 438256, OSUC 438257, OSUC 438258, OSUC 438259, OSUC 438260, OSUC 438261, OSUC 438262, OSUC 438263, OSUC 438264, OSUC 438265, OSUC 438266, OSUC 438267, OSUC 438268, OSUC 438269, OSUC 438270, OSUC 438271, OSUC 438272, OSUC 438273, OSUC 438274, OSUC 438275, OSUC 438277, OSUC 438278, OSUC 438279, OSUC 438280, OSUC 438281 (ANIC); OSUC 448983 (BMNH); OSUC 438276, 448981-448982, 448984-448985 (UQIC); OSUC 438228, 438255, 445349-445350 (WINC).

#### Oxyscelio uncinorum

Burks sp. n.

http://zoobank.org/1514EAB4-C4C6-4AAE-90A1-F9864E0921A6

urn:lsid:biosci.ohio-state.edu:osuc_concepts:307116

http://species-id.net/wiki/Oxyscelio_uncinorum

**Figures 381–386.**
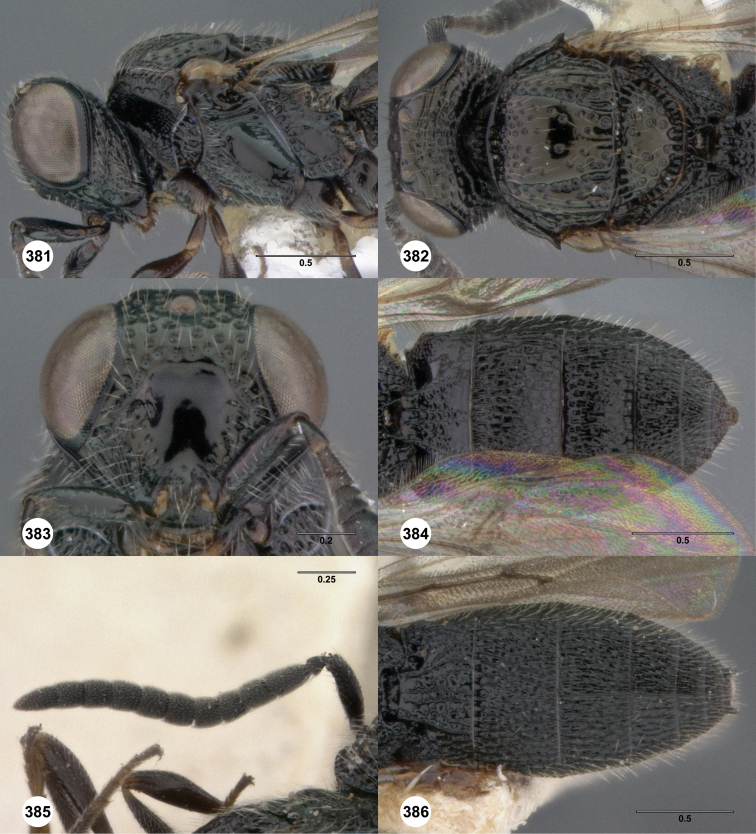
*Oxyscelio uncinorum* sp. n., paratype female (OSUC 462590) **381** Head and mesosoma, lateral view **382** Head and mesosoma, dorsal view **383** Head, anterior view **384** Metasoma, dorsal view. Paratype male (OSUC 359611) **385** Antenna **386** Metasoma, dorsal view. Morphbank^100^

Figures 381–386 ; Morphbank^100^

##### Description.

Female. Body length 3.15–3.75 mm (n=17).

Radicle color and shade: same as scape, both dark brown. Pedicel color: same as scape. A3: shorter than pedicel. A4: broader than long. A5: broader than long.

Ventral clypeal margin: with slightly convex median lobe. Interantennal process: not elongate. Lower frons at dorsal margin of interantennal process: without transverse carina. Transverse curved rugae extending from frontal depression to eye: absent. Median longitudinal carina in frontal depression: absent. Ventral portion of frontal depression: smooth. Dorsal portion of frontal depression: without transverse carinae. Submedian carina: present only as a weak shift in elevation. Frontal depression dorsally: not hood-like, open dorsally. Upper frons major sculpture: umbilicate foveate. Upper frons microsculpture: absent. Hyperoccipital carina: indicated by a set of irregular elevations. Carina connecting occipital carina to hyperoccipital carina: absent. Occipital carina: omicron-shaped, with sharp corners where median portion meets lateral portions. Occiput sculpture: transversely rugose. Extra carina ventral to occipital carina: absent. Gena length: shorter than eye. Major sculpture of gena anteroventrally: umbilicate foveate. Major sculpture of gena posteroventrally: umbilicate foveate; rugose; absent. Microsculpture of gena anteroventrally: absent. Microsculpture of gena posteroventrally: absent.

Lateral pronotal area sculpture: anteriorly smooth, posterodorsal corner with dense microsculpture, ventral corner with irregular carinae. Posterior border of central pronotal area: directed anteriorly, protruding at corner of epomial carina and transverse pronotal carina. Mesoscutum anteriorly: very steep and tall, descending at a right angle or protruding anteriorly. Major sculpture of mesoscutal midlobe anteriorly: umbilicate foveate. Mesoscutal midlobe sculpture at midlength: with large smooth areas. Major sculpture of mesoscutal midlobe posteriorly: umbilicate foveate; longitudinally rugose. Microsculpture of mesoscutal midlobe anteriorly: absent. Microsculpture of mesoscutal midlobe posteriorly: absent. Median mesoscutal carina: present as a vague, occasionally interrupted elevation. Major sculpture of mesoscutellum centrally: absent. Major sculpture of mesoscutellum peripherally: umbilicate foveate. Microsculpture of mesoscutellum centrally: absent. Microsculpture of mesoscutellum peripherally: absent. Mesoscutellar rim: not expanded. Mesoscutellar rim medially: without notch. Mesofemoral depression: with slight, indistinct sculpture dorsally, smooth ventrally. Metascutellum shape: deeply emarginate, with the resulting pair of posterior processes subtriangular and directed dorsally. Metascutellar setae: absent. Metascutellum sculpture: with large smooth posterior fovea. Postmarginal vein: absent. Fore wing apex at rest: exceeding metasomal apex. Coxae color brightness: same color as femora. Spines along tibiae: absent. Lateral propodeal carinae: broadly separated, but parallel for a short distance anteriorly. Setae in metasomal depression: absent. Anterior sculpture of metasomal depression: with median areole or pair of pits. Median propodeal carina: present.

T1 horn: absent. Number of longitudinal carinae of T1 midlobe: 4. T1 lateral carina: protruding laterally, visible from ventral view. T2 sculpture: with longitudinal striae or rugae, setiferous puncta present between them. T2 sublateral longitudinal foveae: absent. T3 metasomal flanges: absent. T4 sculpture: longitudinally striate to rugose, setal pits spanning interspaces. T4 metasomal flanges: absent. T5 sculpture: longitudinally striate to rugose, setal pits spanning interspaces. T5 metasomal flanges: absent. T6: broader than long. Major sculpture of T6: umbilicate punctate. Microsculpture of T6: absent. T6 medially: with medially truncate emargination, sloping down to apical rim. T6 metasomal flanges: absent. T6 raised peripheral rim: absent. S4 sculpture: longitudinally striate or rugose, setal pits spanning interspaces. S5 sculpture: longitudinally striate to rugose, setal pits spanning interspaces. S5 median carina: absent. S6 peripheral carina: absent. S6 apex in relation to T6: not exposed to dorsal view. S6 apex: rounded or acuminate.

*Male*. Body length 3.25–3.85 mm (n=20). A3: longer than pedicel. A5 tyloid shape: narrow, linear. A6: broader than long. A11: longer than broad. Major sculpture of mesoscutal midlobe anteriorly: umbilicate foveate. Major sculpture of mesoscutal midlobe posteriorly: umbilicate foveate; longitudinally rugose. Microsculpture of mesoscutal midlobe anteriorly: granulate. Microsculpture of mesoscutal midlobe posteriorly: absent. Major sculpture of mesoscutellum centrally: umbilicate foveate. Major sculpture of mesoscutellum peripherally: umbilicate foveate. Microsculpture of mesoscutellum centrally: absent. Microsculpture of mesoscutellum peripherally: absent. Fore wing apex at rest: exceeding metasomal apex. T1 midlobe longitudinal carinae: 4; obscured by other raised sculpture. T3 metasomal flanges: absent. T4 metasomal flanges: absent. T5 metasomal flanges: absent. T6 metasomal flanges: absent. T7: with a pair of sharply defined spine-like posterolateral projections.

##### Diagnosis.

Both sexes: Body entirely dark brown, including antennae and legs. Mesoscutum and mesoscutellum black. Frontal depression shallow, transverse carinae absent; submedian carina indicated by weak rugae. Hyperoccipital carina indicated by rugae. Occipital carina complete, omicron-shaped medially. Metascutellum deeply concave, emarginate apically, projecting dorsally. Tibiae with flattened spines. T1 lateral carina expanded laterally. Metasomal sterna with longitudinal rugae. Female: A3 shorter than pedicel. A4, A5 broader than long. T1 midlobe with 4 longitudinal carinae. T6 without metasomal flanges. Fore wing long enough to reach beyond metasomal apex. Main body of T6 not abruptly separated from apical rim. Male: A3 longer than pedicel. A4 broader than long, A11 longer than broad. T1 midlobe with 4 longitudinal carinae that may be obscured by raised sculpture. Fore wing long enough to exceed metasomal apex. T7 with elongate spine-like posterior projections.

##### Etymology.

Latin noun, genitive case, meaning “barbs.”

##### Link to distribution map.

[http://hol.osu.edu/map-full.html?id=307116]

##### Associations.

Collected on *Eucalyptus stellulata* Sieber: [Myrtales: Myrtaceae]

##### Material examined.

Holotype, female: AUSTRALIA: ACT, Honeysuckle Creek, 35°35'S, 149°00'E, 21.III–31.III.1985, malaise trap, I. Naumann & J. Cardale, OSUC 359606 (deposited in ANIC). Paratypes: AUSTRALIA: 15 females, 43 males, ANIC DB 32-020901, OSUC 359607, OSUC 359608, OSUC 359609, OSUC 359610, OSUC 359611, OSUC 359612, OSUC 359613, OSUC 359614, OSUC 359615, OSUC 359616, OSUC 359617, OSUC 359618, OSUC 359619, OSUC 359620, OSUC 359621, OSUC 359622, OSUC 436998, OSUC 436999, OSUC 437000, OSUC 437001, OSUC 439583, OSUC 439584, OSUC 439585, OSUC 439588, OSUC 439589, OSUC 453964, OSUC 453965, OSUC 453966, OSUC 453967, OSUC 453968, OSUC 453969, OSUC 453970, OSUC 453971, OSUC 453972, OSUC 453973, OSUC 453974, OSUC 453975, OSUC 453976, OSUC 453977, OSUC 453978, OSUC 453979, OSUC 453980, OSUC 453981, OSUC 453982, OSUC 453983, OSUC 453984, OSUC 453985 (ANIC); NSW Agriculture ASCT00132286 (ASCU); OSUC 448923-448924 (BMNH); OSUC 462590 (CNCI); OSUC 453986 (MVMA); OSUC 439586, 453987-453989 (TDAH); OSUC 439587 (WINC).

##### Comments.

*Oxyscelio uncinorum* is one of a few Australian species with distinct tibial spines. These are apparently expanded setae, and therefore can be difficult to distinguish, especially on males because of their more densely setose tibiae. Tasmanian specimens were closely examined for differences from mainland specimens, but were not found to be more or less identical.

#### Oxyscelio valdecatenae

Burks sp. n.

http://zoobank.org/02FF1B12-6A0F-499D-9025-EE82FF356794

urn:lsid:biosci.ohio-state.edu:osuc_concepts:307125

http://species-id.net/wiki/Oxyscelio_valdecatenae

**Figures 387–392.**
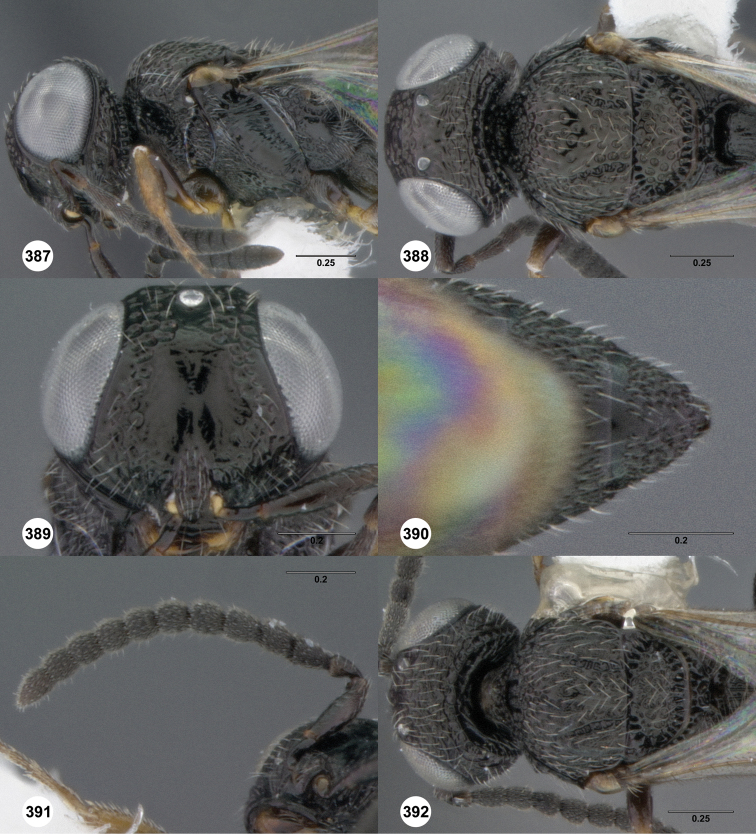
*Oxyscelio valdecatenae* sp. n., paratype female (OSUC 462580) **387** Head and mesosoma, lateral view **388** Head and mesosoma, dorsal view **389** Head, anterior view **390** Metasoma, dorsal view. Paratype male (OSUC 449000) **391** Antenna **392** Metasoma, dorsal view. Morphbank^101^

Figures 387–392 ; Morphbank^101^

##### Description.

Female. Body length 2.75–3.35 mm (n=4).

Radicle color and shade: same as scape, both dark brown. Pedicel color: same as scape. A3: longer than pedicel. A4: longer than broad. A5: broader than long.

Ventral clypeal margin: with slightly convex median lobe. Interantennal process: not elongate. Lower frons at dorsal margin of interantennal process: without transverse carina. Transverse curved rugae extending from frontal depression to eye: absent. Median longitudinal carina in frontal depression: absent. Ventral portion of frontal depression: smooth. Dorsal portion of frontal depression: without transverse carinae. Submedian carina: absent. Frontal depression dorsally: not hood-like, open dorsally. Upper frons major sculpture: umbilicate foveate. Upper frons microsculpture: granulate. Hyperoccipital carina: indicated by a set of irregular elevations. Carina connecting occipital carina to hyperoccipital carina: absent. Occipital carina: uniformly rounded dorsally. Occiput sculpture: umbilicate foveate; transversely rugose. Extra carina ventral to occipital carina: absent. Gena length: shorter than eye. Major sculpture of gena anteroventrally: umbilicate foveate. Major sculpture of gena posteroventrally: umbilicate foveate; umbilicate punctate. Microsculpture of gena anteroventrally: granulate. Microsculpture of gena posteroventrally: granulate.

Lateral pronotal area sculpture: anteriorly smooth, posterodorsal corner with dense microsculpture, ventral corner with irregular carinae. Posterior border of central pronotal area: directed posteriorly, epomial carina absent or meeting transverse pronotal carina at arch on lateral surface of pronotum. Mesoscutum anteriorly: very steep and tall, descending at a right angle or protruding anteriorly. Major sculpture of mesoscutal midlobe anteriorly: umbilicate foveate. Mesoscutal midlobe sculpture at midlength: with large smooth areas. Major sculpture of mesoscutal midlobe posteriorly: umbilicate foveate. Microsculpture of mesoscutal midlobe anteriorly: granulate. Microsculpture of mesoscutal midlobe posteriorly: absent. Median mesoscutal carina: present as a vague, occasionally interrupted elevation. Major sculpture of mesoscutellum centrally: umbilicate foveate. Major sculpture of mesoscutellum peripherally: umbilicate foveate. Microsculpture of mesoscutellum centrally: punctate. Microsculpture of mesoscutellum peripherally: punctate. Mesoscutellar rim: not expanded. Mesoscutellar rim medially: without notch. Mesofemoral depression: with slight, indistinct sculpture dorsally, smooth ventrally. Metascutellum shape: slightly emarginate posteriorly, concave but elevated posteriorly. Metascutellar setae: absent. Metascutellum sculpture: with large smooth posterior fovea. Postmarginal vein: present. Fore wing apex at rest: reaching near apex of T5; reaching middle of T6. Coxae color brightness: same color as femora. Spines along tibiae: absent. Lateral propodeal carinae: broadly separated, not parallel anteriorly. Setae in metasomal depression: absent. Anterior sculpture of metasomal depression: with median areole or pair of pits. Median propodeal carina: absent.

T1 horn: absent. Number of longitudinal carinae of T1 midlobe: obscured by other raised sculpture. T1 lateral carina: straight. T2 sculpture: with longitudinal striae or rugae, setiferous puncta present between them. T2 sublateral longitudinal foveae: absent. T3 metasomal flanges: absent. T4 sculpture: longitudinally striate to rugose, setal pits spanning interspaces. T4 metasomal flanges: absent. T5 sculpture: longitudinally striate to rugose, setal pits spanning interspaces. T5 metasomal flanges: absent. T6: broader than long. Major sculpture of T6: umbilicate punctate. Microsculpture of T6: absent. T6 medially: flat and tapering to a rounded apex, not separated from apical rim. T6 metasomal flanges: absent. T6 raised peripheral rim: absent. S4 sculpture: longitudinally striate or rugose, setal pits spanning interspaces. S5 sculpture: longitudinally striate to rugose, setal pits spanning interspaces. S5 median carina: absent. S6 peripheral carina: absent. S6 apex in relation to T6: not exposed to dorsal view. S6 apex: rounded or acuminate.

*Male*. Body length 2.8 mm (n=1). A3: longer than pedicel. A5 tyloid shape: narrow, linear. A6: longer than broad. A11: broader than long. Major sculpture of mesoscutal midlobe anteriorly: umbilicate foveate. Major sculpture of mesoscutal midlobe posteriorly: umbilicate foveate; longitudinally rugose. Microsculpture of mesoscutal midlobe anteriorly: granulate. Microsculpture of mesoscutal midlobe posteriorly: absent. Major sculpture of mesoscutellum centrally: umbilicate foveate. Major sculpture of mesoscutellum peripherally: umbilicate foveate. Microsculpture of mesoscutellum centrally: absent. Microsculpture of mesoscutellum peripherally: absent. Fore wing apex at rest: exceeding metasomal apex. T1 midlobe longitudinal carinae: 5. T3 metasomal flanges: absent. T4 metasomal flanges: absent. T5 metasomal flanges: absent. T6 metasomal flanges: absent. T7: truncate.

##### Diagnosis.

Both sexes: Frontal depression shallow, transverse carinae absent; submedian carina absent medially. Genal carina expanded, with large foveae between it and gena laterally. Hyperoccipital carina indicated by rugae. Occipital carina complete, convex. Epomial corner slightly protruding. Mesoscutum with raised longitudinal smooth area postero-medially. Metascutellum broad, deeply concave, slightly emarginate apically. Postmarginal vein present. Entire leg dark brown. Metasomal depression with irregular sculpture. T1 lateral carina not expanded laterally. Metasomal flanges absent. Female: A3 longer than pedicel. A4 longer than broad, A5 broader than long. Mesoscutellum without granulate sculpture, densely foveate medially. T1 midlobe carinae obscured by raised area. Fore wing long enough to reach apex of T5 or middle of T6. Male: Fore wing long enough to reach beyond metasomal apex. T7 truncate apically, without apical protrusions. *Oxyscelio valdecatenae* is smaller than *Oxyscelio catenae*, but differs chiefly in having a much more strongly sculptured mesoscutellum. Some additional more subtle differences exist, especially in metasomal length and frontal depression sculpture.

##### Etymology.

Latin noun, genitive case, meaning “strong chain.”

##### Link to distribution map.

[http://hol.osu.edu/map-full.html?id=307125]

##### Material examined.

Holotype, female: AUSTRALIA: WA, 5km SE Nornalup, coastal heathlands, Walpole-Nornalup National Park, 17.XII-18.XII.1990, pan trap, A. D. Austin, OSUC 448998 (deposited in WAMP). Paratypes: AUSTRALIA: 3 females, 1 male, OSUC 462579-462580 (CNCI); OSUC 448999-449000 (WINC).

#### Oxyscelio velamenti

Burks sp. n.

http://zoobank.org/0619CBDE-BF3F-4AAE-9AC1-A02F4AF61D02

urn:lsid:biosci.ohio-state.edu:osuc_concepts:307117

http://species-id.net/wiki/Oxyscelio_velamenti

**Figures 393–396.**
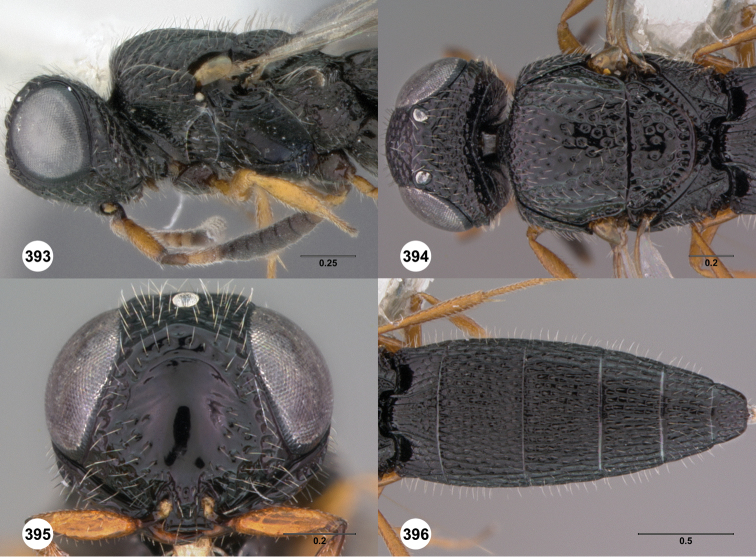
*Oxyscelio velamenti* sp. n., holotype female (OSUC 436034) **393** Head and mesosoma, lateral view **394** Head and mesosoma, dorsal view **395** Head, anterior view **396** Metasoma, dorsal view. Morphbank^102^

Figures 393–396 ; Morphbank^102^

##### Description.

Female. Body length 3.4–3.5 mm (n=4).

Radicle color and shade: darker than scape. Pedicel color: same as scape. A3: shorter than pedicel; as long as pedicel. A4: broader than long. A5: broader than long.

Ventral clypeal margin: with slightly convex median lobe. Interantennal process: not elongate. Lower frons at dorsal margin of interantennal process: without transverse carina. Transverse curved rugae extending from frontal depression to eye: absent. Median longitudinal carina in frontal depression: absent. Ventral portion of frontal depression: with medially interrupted transverse carinae. Dorsal portion of frontal depression: with medially interrupted transverse carinae. Submedian carina: present. Frontal depression dorsally: hood-like, dorsally protruding. Upper frons major sculpture: umbilicate foveate. Upper frons microsculpture: absent. Hyperoccipital carina: absent. Carina connecting occipital carina to hyperoccipital carina: absent. Occipital carina: weakly arched dorsally, with rounded lateral corners. Occiput sculpture: irregularly sculptured. Extra carina ventral to occipital carina: present, complete. Gena length: shorter than eye. Major sculpture of gena anteroventrally: umbilicate foveate. Major sculpture of gena posteroventrally: umbilicate foveate; absent. Microsculpture of gena anteroventrally: absent. Microsculpture of gena posteroventrally: absent.

Lateral pronotal area sculpture: with shallow irregular carinae, posterodorsal corner with dense microsculpture. Posterior border of central pronotal area: directed anteriorly, protruding at corner of epomial carina and transverse pronotal carina. Mesoscutum anteriorly: very steep and tall, descending at a right angle or protruding anteriorly. Major sculpture of mesoscutal midlobe anteriorly: umbilicate foveate. Mesoscutal midlobe sculpture at midlength: with large smooth areas. Major sculpture of mesoscutal midlobe posteriorly: umbilicate foveate. Microsculpture of mesoscutal midlobe anteriorly: granulate. Microsculpture of mesoscutal midlobe posteriorly: absent. Median mesoscutal carina: present as a vague, occasionally interrupted elevation. Major sculpture of mesoscutellum centrally: absent; umbilicate foveate. Major sculpture of mesoscutellum peripherally: umbilicate foveate. Microsculpture of mesoscutellum centrally: absent. Microsculpture of mesoscutellum peripherally: punctate. Mesoscutellar rim: not expanded. Mesoscutellar rim medially: without notch. Mesofemoral depression: longitudinally striate dorsally, smooth ventrally. Metascutellum shape: not emarginate, concave but elevated posteriorly. Metascutellar setae: absent. Metascutellum sculpture: with large smooth posterior fovea. Postmarginal vein: absent; but marginal vein curving slightly at apex. Fore wing apex at rest: reaching base of T5. Coxae color brightness: darker than femora. Spines along tibiae: absent. Lateral propodeal carinae: broadly separated, not parallel anteriorly. Setae in metasomal depression: absent. Anterior sculpture of metasomal depression: absent. Median propodeal carina: absent.

T1 horn: absent. Number of longitudinal carinae of T1 midlobe: 4. T1 lateral carina: protruding laterally, visible from ventral view. T2 sculpture: with longitudinal striae or rugae, setiferous puncta present between them. T2 sublateral longitudinal foveae: absent. T3 metasomal flanges: absent. T4 sculpture: longitudinally striate to rugose, setal pits spanning interspaces. T4 metasomal flanges: present as slightly protruding sharp corners. T5 sculpture: longitudinally striate to rugose, setal pits spanning interspaces. T5 metasomal flanges: present as strongly protruding acuminate flanges. T6: broader than long. Major sculpture of T6: longitudinally striate; umbilicate foveate. Microsculpture of T6: absent. T6 medially: slightly emarginate, not separated from apical rim. T6 metasomal flanges: present as slightly expanded lateral rims, rounded posteriorly. T6 raised peripheral rim: absent. S4 sculpture: longitudinally striate or rugose, setal pits spanning interspaces. S5 sculpture: longitudinally striate to rugose, setal pits spanning interspaces. S5 median carina: absent. S6 peripheral carina: present, posteriorly complete. S6 apex in relation to T6: exposed to dorsal view by T6 emargination. S6 apex: rounded or acuminate.

*Male*. unknown.

##### Diagnosis.

Both sexes: Frontal depression deep, without carinae ventrally, dorsal separator interrupted medially, one or more interrupted carinae present above dorsal separator; submedian carina weakly defined or absent medially. Hyperoccipital carina absent. Occipital carina complete, medially weakly convex. Mesoscutellar rim not expanded, without median notch. Metascutellum tiny, projecting dorsally. Coxa darker than rest of leg. Postmarginal vein absent. Female: A3 longer than pedicel. A4, A5 broader than long. T4, T5 without distinct metasomal flanges. Main surface of T6 with slightly expanded lateral margins, truncate or very slightly emarginate apically, but sharply separated from apical rim. S6 exposed to dorsal view, rounded apically. *Oxyscelio scismatis* has a similar metasomal apex, but differs in having a strong submedian carina and T5 metasomal flanges. *Oxyscelio contusionis* is very similar, but has a strongly concave T6.

##### Etymology.

Latin noun, genitive case, meaning “a covering.”

##### Link to distribution map.

[http://hol.osu.edu/map-full.html?id=307117]

##### Material examined.

Holotype, female: AUSTRALIA: SA, coastal dunes, 15km SSW Streaky Bay, 10.XI–15.XI.1987, pan trap, A. D. Austin, OSUC 436034 (deposited in SAMA). Paratypes: AUSTRALIA: 4 females, OSUC 436030-436033 (WINC).

#### Oxyscelio verrucae

Burks sp. n.

http://zoobank.org/2C1C5441-6310-4DC7-8651-0B8BD2C8C3AB

urn:lsid:biosci.ohio-state.edu:osuc_concepts:307118

http://species-id.net/wiki/Oxyscelio_verrucae

**Figures 397–402.**
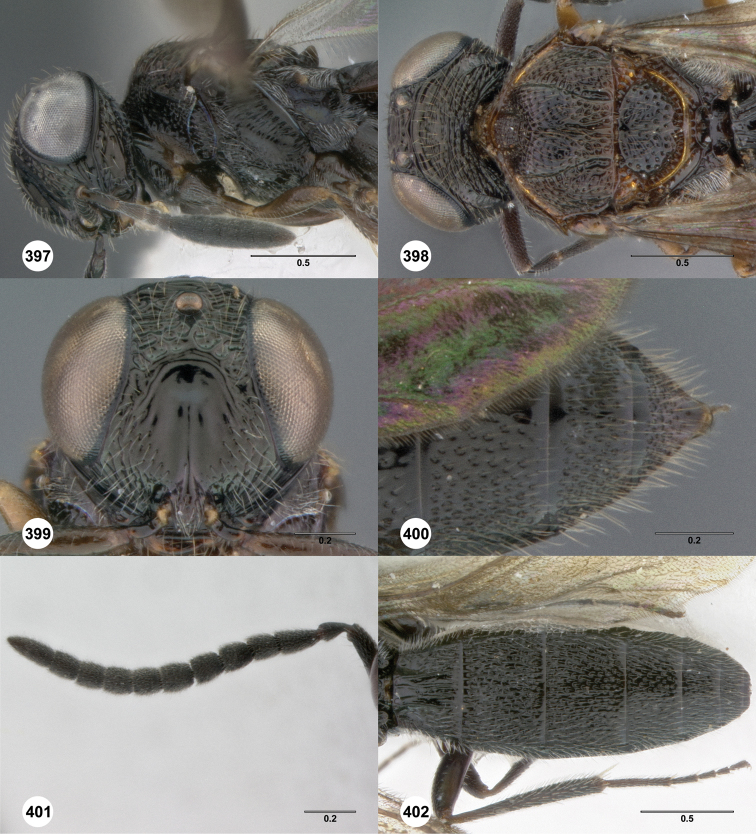
*Oxyscelio verrucae* sp. n., paratype female (OSUC 438819) **397** Head and mesosoma, lateral view **398** Head and mesosoma, dorsal view **399** Head, anterior view **400** Metasoma, dorsal view. Paratype male (OSUC 438825) **401** Antenna **402** Metasoma, dorsal view. Morphbank^103^

Figures 397–402 ; Morphbank^103^

##### Description.

Female. Body length 3.5–4 mm (n=11).

Radicle color and shade: same as scape, both dark brown. Pedicel color: at least partially darker than scape. A3: longer than pedicel. A4: longer than broad; as long as broad. A5: broader than long.

Ventral clypeal margin: with slightly convex median lobe. Interantennal process: not elongate. Lower frons at dorsal margin of interantennal process: without transverse carina. Transverse curved rugae extending from frontal depression to eye: absent. Median longitudinal carina in frontal depression: absent. Ventral portion of frontal depression: smooth. Dorsal portion of frontal depression: with some transverse carinae. Submedian carina: present. Frontal depression dorsally: not hood-like, open dorsally. Upper frons major sculpture: umbilicate foveate; irregularly rugose. Upper frons microsculpture: absent. Hyperoccipital carina: present as a single carina. Carina connecting occipital carina to hyperoccipital carina: absent. Occipital carina: present laterally, absent medially. Occiput sculpture: irregularly sculptured. Extra carina ventral to occipital carina: present, complete. Gena length: shorter than eye. Major sculpture of gena anteroventrally: umbilicate foveate; umbilicate punctate. Major sculpture of gena posteroventrally: umbilicate foveate; umbilicate punctate. Microsculpture of gena anteroventrally: absent. Microsculpture of gena posteroventrally: absent.

Lateral pronotal area sculpture: smooth anteriorly, densely setose posteriorly. Posterior border of central pronotal area: directed posteriorly, epomial carina absent or meeting transverse pronotal carina at arch on lateral surface of pronotum. Mesoscutum anteriorly: very steep and tall, descending at a right angle or protruding anteriorly. Major sculpture of mesoscutal midlobe anteriorly: transversely rugose; umbilicate punctate. Mesoscutal midlobe sculpture at midlength: not different from nearby sculpture. Major sculpture of mesoscutal midlobe posteriorly: umbilicate foveate; longitudinally rugose. Microsculpture of mesoscutal midlobe anteriorly: absent. Microsculpture of mesoscutal midlobe posteriorly: absent. Median mesoscutal carina: absent. Major sculpture of mesoscutellum centrally: umbilicate foveate; obliquely rugose. Major sculpture of mesoscutellum peripherally: umbilicate punctate. Microsculpture of mesoscutellum centrally: absent. Microsculpture of mesoscutellum peripherally: absent. Mesoscutellar rim: not expanded. Mesoscutellar rim medially: without notch. Mesofemoral depression: longitudinally striate dorsally, smooth ventrally. Metascutellum shape: deeply emarginate, with the resulting pair of posterior processes subtriangular and directed dorsally. Metascutellar setae: absent. Metascutellum sculpture: with large smooth posterior fovea. Postmarginal vein: absent. Fore wing apex at rest: exceeding metasomal apex. Coxae color brightness: darker than femora. Spines along tibiae: absent. Lateral propodeal carinae: broadly separated, but parallel for a short distance anteriorly. Setae in metasomal depression: absent. Anterior sculpture of metasomal depression: absent. Median propodeal carina: absent.

T1 horn: absent. Number of longitudinal carinae of T1 midlobe: 4. T1 lateral carina: protruding laterally, visible from ventral view. T2 sculpture: anteriorly longitudinally rugose with setal pits spanning interspaces, posteromedially sparsely foveate. T2 sublateral longitudinal foveae: absent. T3 metasomal flanges: absent. T4 sculpture: densely foveate, longitudinal sculpture irregular. T4 metasomal flanges: absent. T5 sculpture: densely foveate, longitudinal sculpture irregular. T5 metasomal flanges: absent. T6: broader than long. Major sculpture of T6: umbilicate punctate. Microsculpture of T6: absent. T6 medially: flat and tapering to a rounded apex, not separated from apical rim. T6 metasomal flanges: absent. T6 raised peripheral rim: absent. S4 sculpture: smooth, with tiny umbilicate foveae. S5 sculpture: smooth, with tiny umbilicate foveae. S5 median carina: absent. S6 peripheral carina: absent. S6 apex in relation to T6: not exposed to dorsal view. S6 apex: rounded or acuminate.

*Male*. Body length 3.15–3.8 mm (n=17). A3: longer than pedicel. A5 tyloid shape: narrow, linear. A6: broader than long. A11: longer than broad. Major sculpture of mesoscutal midlobe anteriorly: transversely rugose; umbilicate punctate. Major sculpture of mesoscutal midlobe posteriorly: umbilicate foveate; longitudinally rugose. Microsculpture of mesoscutal midlobe anteriorly: absent; granulate. Microsculpture of mesoscutal midlobe posteriorly: absent. Major sculpture of mesoscutellum centrally: umbilicate foveate; umbilicate punctate. Major sculpture of mesoscutellum peripherally: umbilicate punctate. Microsculpture of mesoscutellum centrally: absent. Microsculpture of mesoscutellum peripherally: absent. Fore wing apex at rest: exceeding metasomal apex. T1 midlobe longitudinal carinae: 5. T3 metasomal flanges: absent. T4 metasomal flanges: absent. T5 metasomal flanges: absent. T6 metasomal flanges: absent. T7: truncate.

##### Diagnosis.

Both sexes: Frontal depression small and shallow, transverse carinae absent; submedian carina sharp. Hyperoccipital carina indicated by a set of sharp carinae. Occipital carina complete or incomplete, sometimes gradually becoming indistinguishable medially; occiput with many transverse rugae. Mesoscutum and sometimes mesoscutellum with deeply sunken midline, causing surrounding areas to apparently protrude. Metascutellum broad and deeply concave, laterally with only a narrow longitudinally striate area, weakly emarginate or truncate apically, projecting dorsally. Postmarginal vein absent or extremely short. T1 lateral carina not expanded laterally. Female: A3 longer than pedicel. A4 longer than broad, A5 nearly as long as broad. T1 midlobe with 4 longitudinal carinae that may be obscured by a smooth raised area. Fore wing long enough to reach middle of T6. T6 broader than long. Male: All flagellomeres past A3 about as long as broad. T1 midlobe with 5 longitudinal carinae (submedian pair weak). Fore wing long enough to reach beyond T7. T7 tiny, truncate or weakly convex.

##### Etymology.

Latin noun, genitive case, meaning “wart.”

##### Link to distribution map.

[http://hol.osu.edu/map-full.html?id=307118]

##### Material examined.

Holotype, female: AUSTRALIA: NSW, Point Lookout, New England National Park, 30°29'S, 152°25'E, 12.II–22.II.1984, malaise trap, I. D. Naumann, OSUC 442343 (deposited in ANIC). Paratypes: AUSTRALIA: 11 females, 17 males, Australian Museum K245261 (AMSA); OSUC 438815-438817, 438820-438824, 442342, 442345-442354 (ANIC); OSUC 227558, 462571, 462573-462574 (CNCI); OSUC 438818-438819, 438825, 442344 (WINC).

##### Comments.

*Oxyscelio verrucae* is unusual in having a deep median impression on the mesoscutum, such that submedian areas apparently protrude. It is also unusual in having a large number of strong transverse rugae on the occiput, making it difficult to determine if the occipital carina is present medially or not. This species otherwise resembles *Oxyscelio concoloripes*, and is provisionally placed in a group with it. Some specimens exist with a completely dark brown body, including antennae and legs. These are considered to be melanistic specimens of the same species.

#### Oxyscelio viator

Burks sp. n.

http://zoobank.org/A8368A31-075B-4092-9A8F-E30F89AE231E

urn:lsid:biosci.ohio-state.edu:osuc_concepts:307122

http://species-id.net/wiki/Oxyscelio_viator

**Figures 403–406.**
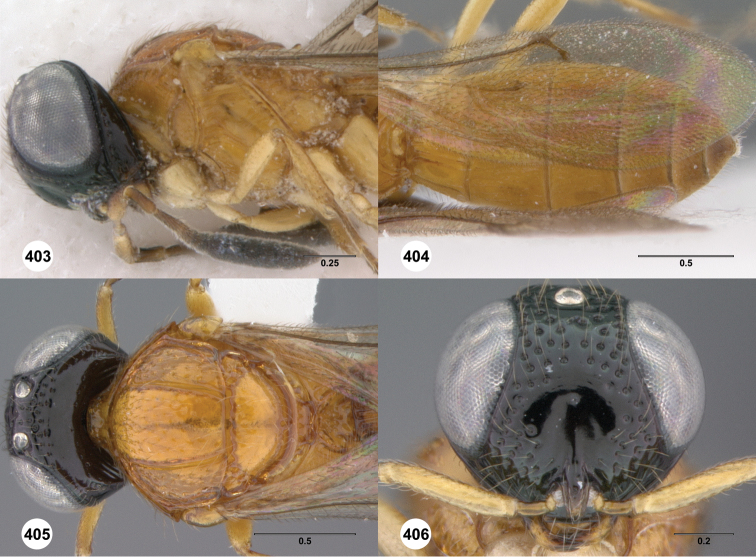
*Oxyscelio viator* sp. n., holotype female (OSUC 148457) **403** Head and mesosoma, lateral view **404** Metasoma, dorsal view. Paratype female (OSUC 283330) **405** Head and mesosoma, dorsal view **406** Head, anterior view. Morphbank^104^

Figures 403–406 ; Morphbank^104^

##### Description.

Female. Body length 3.4–3.7 mm (n=2).

Radicle color and shade: same as scape, both yellowish or reddish. Pedicel color: at least partially darker than scape. A3: longer than pedicel. A4: longer than broad. A5: broader than long.

Ventral clypeal margin: with slightly convex median lobe. Interantennal process: not elongate. Lower frons at dorsal margin of interantennal process: without transverse carina. Transverse curved rugae extending from frontal depression to eye: absent. Median longitudinal carina in frontal depression: absent. Ventral portion of frontal depression: smooth. Dorsal portion of frontal depression: without transverse carinae. Submedian carina: absent. Frontal depression dorsally: not hood-like, open dorsally. Upper frons major sculpture: umbilicate foveate. Upper frons microsculpture: absent. Hyperoccipital carina: absent. Carina connecting occipital carina to hyperoccipital carina: absent. Occipital carina: present laterally, absent medially. Occiput sculpture: smooth. Extra carina ventral to occipital carina: absent. Gena length: shorter than eye. Major sculpture of gena anteroventrally: umbilicate foveate; absent. Major sculpture of gena posteroventrally: umbilicate punctate; absent. Microsculpture of gena anteroventrally: absent. Microsculpture of gena posteroventrally: absent.

Lateral pronotal area sculpture: anteriorly smooth, posterodorsal corner with dense microsculpture, ventral corner with irregular carinae. Posterior border of central pronotal area: directed posteriorly, epomial carina absent or meeting transverse pronotal carina at arch on lateral surface of pronotum. Mesoscutum anteriorly: not steep, forming less than a right angle. Major sculpture of mesoscutal midlobe anteriorly: umbilicate foveate. Mesoscutal midlobe sculpture at midlength: not different from nearby sculpture. Major sculpture of mesoscutal midlobe posteriorly: umbilicate foveate. Microsculpture of mesoscutal midlobe anteriorly: absent. Microsculpture of mesoscutal midlobe posteriorly: absent. Median mesoscutal carina: absent. Major sculpture of mesoscutellum centrally: umbilicate foveate. Major sculpture of mesoscutellum peripherally: umbilicate foveate; umbilicate punctate. Microsculpture of mesoscutellum centrally: absent. Microsculpture of mesoscutellum peripherally: absent. Mesoscutellar rim: not expanded. Mesoscutellar rim medially: without notch. Mesofemoral depression: longitudinally striate dorsally, smooth ventrally. Metascutellum shape: deeply emarginate, with the resulting pair of posterior processes subtriangular and directed dorsally. Metascutellar setae: absent. Metascutellum sculpture: with large smooth posterior fovea. Postmarginal vein: present. Fore wing apex at rest: exceeding metasomal apex. Coxae color brightness: same color as femora. Spines along tibiae: absent. Lateral propodeal carinae: broadly separated, not parallel anteriorly. Setae in metasomal depression: absent. Anterior sculpture of metasomal depression: with median areole or pair of pits. Median propodeal carina: absent.

T1 horn: absent. Number of longitudinal carinae of T1 midlobe: 5. T1 lateral carina: straight. T2 sculpture: with longitudinal striae or rugae, setiferous puncta present between them. T2 sublateral longitudinal foveae: absent. T3 metasomal flanges: absent. T4 sculpture: longitudinally striate to rugose, setal pits spanning interspaces. T4 metasomal flanges: absent. T5 sculpture: mostly smooth, with tiny umbilicate foveae. T5 metasomal flanges: absent. T6: broader than long. Major sculpture of T6: umbilicate punctate. Microsculpture of T6: absent. T6 medially: flat and tapering to a rounded apex, not separated from apical rim. T6 metasomal flanges: absent. T6 raised peripheral rim: absent. S4 sculpture: longitudinally striate or rugose, setal pits spanning interspaces. S5 sculpture: longitudinally striate to rugose, setal pits spanning interspaces. S5 median carina: absent. S6 peripheral carina: absent. S6 apex in relation to T6: not exposed to dorsal view. S6 apex: rounded or acuminate.

*Male*. unknown.

##### Diagnosis.

Both sexes: Frontal depression nearly flat; submedian carina absent. Hyperoccipital carina absent. Occipital carina incomplete, lateral portions not approaching hyperoccipital carina. Occiput umbilicate punctate. Metascutellum broad and deeply emarginate. Postmarginal vein present. Coxa same color as rest of leg. T1 lateral carina not expanded laterally. Metasomal flanges absent. Female: A3 longer than pedicel. Mesoscutellum with small umbilicate foveae or pits. T1 midlobe without anterior horn, with 5 longitudinal carinae. Fore wing long enough to reach beyond metasomal apex. T6 broader than long.

##### Etymology.

Latin noun in apposition, meaning “traveller.”

##### Link to distribution map.

[http://hol.osu.edu/map-full.html?id=307122]

##### Material examined.

Holotype, female: VANUATU: Sanma Prov., Espiritu Santo Isl., 900A ground / moist lowland forest, MG09A2, Penaoru, 14°58'00.17"S, 166°39'21.69"E, ~900m, 18.XI–30.XI.2006, malaise trap, C. Villemant, OSUC 283330 (deposited in MNHN). Paratype: FIJI: 1 female, OSUC 148457 (QMBA).

##### Comments.

*Oxyscelio viator* is unique within the *flavipes*-group in lacking a hyperoccipital carina. A weak swelling occurs in the area, but this is incomplete and not recognizable as a carina. The otherwise unusually smooth head and mesosoma help distinguish this species.

#### Oxyscelio wa

Burks sp. n.

http://zoobank.org/BB25A9D6-18C5-405A-B3B6-18DE7C18CE09

urn:lsid:biosci.ohio-state.edu:osuc_concepts:307119

http://species-id.net/wiki/Oxyscelio_wa

**Figures 407–412.**
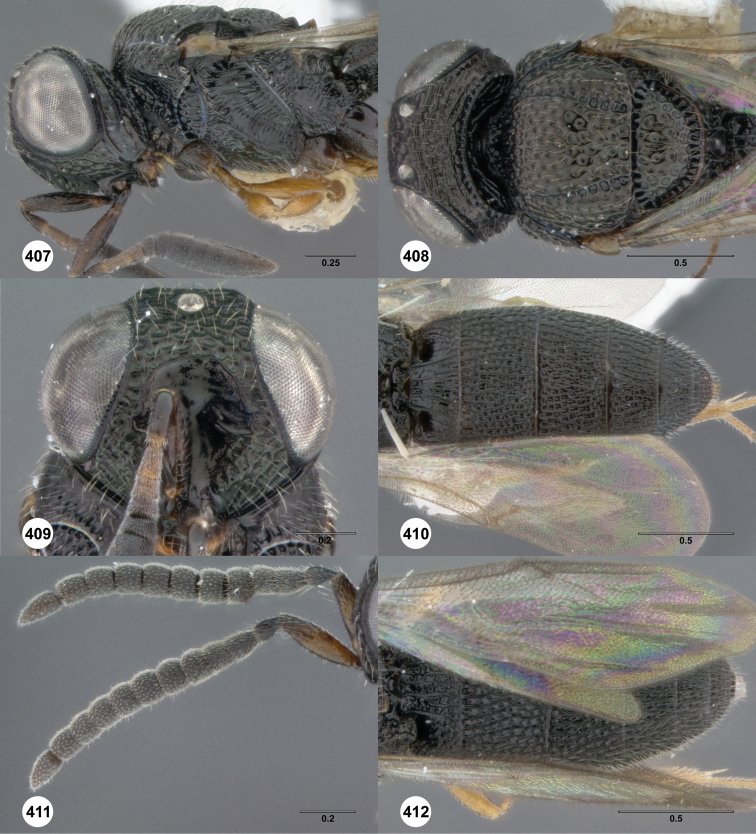
*Oxyscelio wa* sp. n., holotype female (OSUC 449005) **407** Head and mesosoma, lateral view **408** Head and mesosoma, dorsal view **409** Head, anterior view. Paratype female (OSUC 449009) **410** Metasoma, dorsal view. Paratype male (OSUC 449019) **411** Antenna **412** Metasoma, dorsal view. Morphbank^105^

Figures 407–412 ; Morphbank^105^

##### Description.

Female. Body length 3.2–3.55 mm (n=10).

Radicle color and shade: same as scape, both dark brown. Pedicel color: same as scape. A3: longer than pedicel. A4: broader than long. A5: broader than long.

Ventral clypeal margin: with slightly convex median lobe. Interantennal process: not elongate. Lower frons at dorsal margin of interantennal process: without transverse carina. Transverse curved rugae extending from frontal depression to eye: absent. Median longitudinal carina in frontal depression: absent. Ventral portion of frontal depression: smooth. Dorsal portion of frontal depression: without transverse carinae. Submedian carina: present. Frontal depression dorsally: not hood-like, open dorsally. Upper frons major sculpture: umbilicate foveate; irregularly rugose. Upper frons microsculpture: absent. Hyperoccipital carina: indicated by a set of irregular elevations. Carina connecting occipital carina to hyperoccipital carina: absent. Occipital carina: weakly arched dorsally, with rounded lateral corners. Occiput sculpture: irregularly sculptured. Extra carina ventral to occipital carina: present, complete. Gena length: shorter than eye. Major sculpture of gena anteroventrally: umbilicate foveate. Major sculpture of gena posteroventrally: umbilicate foveate; rugose. Microsculpture of gena anteroventrally: granulate. Microsculpture of gena posteroventrally: granulate.

Lateral pronotal area sculpture: densely covered with setiferous puncta. Posterior border of central pronotal area: directed posteriorly, epomial carina absent or meeting transverse pronotal carina at arch on lateral surface of pronotum. Mesoscutum anteriorly: very steep and tall, descending at a right angle or protruding anteriorly. Major sculpture of mesoscutal midlobe anteriorly: umbilicate foveate; longitudinally rugose. Mesoscutal midlobe sculpture at midlength: not different from nearby sculpture. Major sculpture of mesoscutal midlobe posteriorly: umbilicate foveate; longitudinally rugose. Microsculpture of mesoscutal midlobe anteriorly: granulate. Microsculpture of mesoscutal midlobe posteriorly: absent. Median mesoscutal carina: present as a vague, occasionally interrupted elevation. Major sculpture of mesoscutellum centrally: umbilicate foveate. Major sculpture of mesoscutellum peripherally: umbilicate foveate. Microsculpture of mesoscutellum centrally: absent. Microsculpture of mesoscutellum peripherally: absent. Mesoscutellar rim: not expanded. Mesoscutellar rim medially: without notch. Mesofemoral depression: longitudinally striate dorsally and ventrally. Metascutellum shape: slightly emarginate posteriorly, concave but elevated posteriorly. Metascutellar setae: absent. Metascutellum sculpture: with large smooth posterior fovea. Postmarginal vein: present. Fore wing apex at rest: exceeding metasomal apex. Coxae color brightness: darker than femora. Spines along tibiae: absent. Lateral propodeal carinae: broadly separated, not parallel anteriorly. Setae in metasomal depression: absent. Anterior sculpture of metasomal depression: absent. Median propodeal carina: absent.

T1 horn: absent. Number of longitudinal carinae of T1 midlobe: 4. T1 lateral carina: protruding laterally, visible from ventral view. T2 sculpture: with longitudinal striae or rugae, setiferous puncta present between them. T2 sublateral longitudinal foveae: absent. T3 metasomal flanges: absent. T4 sculpture: longitudinally striate to rugose, setal pits spanning interspaces. T4 metasomal flanges: absent. T5 sculpture: longitudinally striate to rugose, setal pits spanning interspaces. T5 metasomal flanges: absent. T6: broader than long. Major sculpture of T6: umbilicate punctate; umbilicate foveate. Microsculpture of T6: absent. T6 medially: flat and tapering to a rounded apex, not separated from apical rim. T6 metasomal flanges: absent. T6 raised peripheral rim: absent. S4 sculpture: longitudinally striate or rugose, setal pits spanning interspaces. S5 sculpture: longitudinally striate to rugose, setal pits spanning interspaces. S5 median carina: present. S6 peripheral carina: absent. S6 apex in relation to T6: not exposed to dorsal view. S6 apex: rounded or acuminate.

*Male*. Body length 3.1–3.3 mm (n=7). A3: longer than pedicel. A5 tyloid shape: narrow, linear. A6: broader than long. A11: longer than broad; as long as broad. Major sculpture of mesoscutal midlobe anteriorly: umbilicate foveate. Major sculpture of mesoscutal midlobe posteriorly: umbilicate foveate. Microsculpture of mesoscutal midlobe anteriorly: granulate. Microsculpture of mesoscutal midlobe posteriorly: absent. Major sculpture of mesoscutellum centrally: umbilicate punctate. Major sculpture of mesoscutellum peripherally: umbilicate foveate. Microsculpture of mesoscutellum centrally: absent. Microsculpture of mesoscutellum peripherally: absent. Fore wing apex at rest: exceeding metasomal apex. T1 midlobe longitudinal carinae: 4. T3 metasomal flanges: absent. T4 metasomal flanges: absent. T5 metasomal flanges: absent. T6 metasomal flanges: absent. T7: truncate.

##### Diagnosis.

Both sexes: Frontal depression with broadly interrupted transverse carinae, including some above dorsal separator; submedian carina weakly indicated but complete. Hyperoccipital carina indicated by weak rugae. Occipital carina complete, weakly convex medially. Metascutellum deeply concave, emarginate apically, projecting dorsally. Postmarginal vein present, short. Coxa darker than rest of leg. T1 lateral carina expanded laterally. Metasomal flanges absent. Female: A4, A5 much broader than long. Mesoscutellum without granulate sculpture. Fore wing long enough to reach middle of T6 or beyond metasomal apex. T1 midlobe with 4 longitudinal carinae. T6 without metasomal flanges, main body of tergum not separated from apical rim, strongly sloping apically but not concave. Male: A4 broader than long, A11 slightly longer than broad. T1 midlobe with 4 longitudinal carinae. Fore wing long enough to reach beyond metasomal apex. T7 tiny, truncate apically. *Oxyscelio wa* is very similar to *Oxyscelio nigricoxa* and in some ways to *Oxyscelio hyalinipennis*, but differs in having a shorter A4 and A5 in females, and in having a postmarginal vein in combination with a truncate T7 in males. Known specimens of *Oxyscelio wa* also have dark coxae, which does not occur in *Oxyscelio hyalinipennis*. The fore wing venation is at most slightly separated from the anterior wing margin, which aids in distinguishing this species from *Oxyscelio nigricoxa*, a species where the venation does not closely approach the anterior wing margin.

##### Etymology.

Arbitrary combination of letters, does not change spelling to agree with gender.

##### Link to distribution map.

[http://hol.osu.edu/map-full.html?id=307119]

##### Material examined.

Holotype, female: AUSTRALIA: WA, Mount Cooke, 17.II-18.IV.1991, malaise trap, M. S. Harvey & J. M. Waldock, OSUC 449005 (deposited in WAMP). Paratypes: AUSTRALIA: 9 females, 7 males, OSUC 449006-449021 (WINC).

## Supplementary Material

XML Treatment for Oxyscelio

XML Treatment for Oxyscelio aciculae

XML Treatment for Oxyscelio anfractus

XML Treatment for Oxyscelio atricoxa

XML Treatment for Oxyscelio bellariorum

XML Treatment for Oxyscelio bicoloripedis

XML Treatment for Oxyscelio brevitas

XML Treatment for Oxyscelio catenae

XML Treatment for Oxyscelio caudarum

XML Treatment for Oxyscelio circulorum

XML Treatment for Oxyscelio clivi

XML Treatment for Oxyscelio clupei

XML Treatment for Oxyscelio concoloripes

XML Treatment for Oxyscelio conjuncti

XML Treatment for Oxyscelio contusionis

XML Treatment for Oxyscelio corrugationis

XML Treatment for Oxyscelio croci

XML Treatment for Oxyscelio cuspidis

XML Treatment for Oxyscelio densitatis

XML Treatment for Oxyscelio dissimulationis

XML Treatment for Oxyscelio divisionis

XML Treatment for Oxyscelio exiguitatis

XML Treatment for Oxyscelio flavipes

XML Treatment for Oxyscelio fluctuum

XML Treatment for Oxyscelio foliorum

XML Treatment for Oxyscelio funis

XML Treatment for Oxyscelio grandis

XML Treatment for Oxyscelio gressus

XML Treatment for Oxyscelio hamorum

XML Treatment for Oxyscelio hyalinipennis

XML Treatment for Oxyscelio incisurae

XML Treatment for Oxyscelio lenitatis

XML Treatment for Oxyscelio leviventris

XML Treatment for Oxyscelio limbi

XML Treatment for Oxyscelio liminis

XML Treatment for Oxyscelio linguae

XML Treatment for Oxyscelio lintris

XML Treatment for Oxyscelio livens

XML Treatment for Oxyscelio magniclava

XML Treatment for Oxyscelio mirellus

XML Treatment for Oxyscelio montanus

XML Treatment for Oxyscelio mystacis

XML Treatment for Oxyscelio nasi

XML Treatment for Oxyscelio nigriclava

XML Treatment for Oxyscelio nigricoxa

XML Treatment for Oxyscelio nitoris

XML Treatment for Oxyscelio obliquiatis

XML Treatment for Oxyscelio oblongiclypei

XML Treatment for Oxyscelio obturationis

XML Treatment for Oxyscelio oculi

XML Treatment for Oxyscelio palati

XML Treatment for Oxyscelio pectinis

XML Treatment for Oxyscelio pollicis

XML Treatment for Oxyscelio proceritatis

XML Treatment for Oxyscelio productionis

XML Treatment for Oxyscelio radii

XML Treatment for Oxyscelio rami

XML Treatment for Oxyscelio rugulosus

XML Treatment for Oxyscelio rupturae

XML Treatment for Oxyscelio sarcinae

XML Treatment for Oxyscelio scismatis

XML Treatment for Oxyscelio sciuri

XML Treatment for Oxyscelio scutorum

XML Treatment for Oxyscelio sepisessor

XML Treatment for Oxyscelio shakespearei

XML Treatment for Oxyscelio sinuationis

XML Treatment for Oxyscelio solitarius

XML Treatment for Oxyscelio sordes

XML Treatment for Oxyscelio spatulae

XML Treatment for Oxyscelio stipulae

XML Treatment for Oxyscelio stringerae

XML Treatment for Oxyscelio tenuitatis

XML Treatment for Oxyscelio truncationis

XML Treatment for Oxyscelio tubi

XML Treatment for Oxyscelio umbonis

XML Treatment for Oxyscelio uncinorum

XML Treatment for Oxyscelio valdecatenae

XML Treatment for Oxyscelio velamenti

XML Treatment for Oxyscelio verrucae

XML Treatment for Oxyscelio viator

XML Treatment for Oxyscelio wa

## Endnotes

1 urn:lsid:biocol.org:col:1008

2 urn:lsid:biocol.org:col:32975

3 urn:lsid:biocol.org:col:32981

4 urn:lsid:biocol.org:col:32997

5 urn:lsid:biocol.org:col:1009

6 urn:lsid:biocol.org:col:1012

7 urn:lsid:biocol.org:col:33527

8 urn:lsid:biocol.org:col:33545

9 urn:lsid:biocol.org:col:33791

10 urn:lsid:biocol.org:col:33864

11 urn:lsid:biocol.org:col:33928

12 urn:lsid:biocol.org:col:1019

13 urn:lsid:biocol.org:col:1014

14 urn:lsid:biocol.org:col:34157

15 urn:lsid:biocol.org:col:34161

16 urn:lsid:biocol.org:col:34165

17 urn:lsid:biocol.org:col:34829

18 urn:lsid:biocol.org:col:34982

19 urn:lsid:biocol.org:col:34248

20 urn:lsid:biocol.org:col:34344

21 urn:lsid:biocol.org:col:34423

22 urn:lsid:biocol.org:col:34515

23 urn:lsid:biocol.org:col:1019

24 urn:lsid:biocol.org:col:34578

25 urn:lsid:biocol.org:col:34593

26 http://www.morphbank.net/?id=823007

27 http://www.morphbank.net/?id=823008

28 http://www.morphbank.net/?id=823009

29 http://www.morphbank.net/?id=823010

30 http://www.morphbank.net/?id=823011

31 http://www.morphbank.net/?id=823012

32 http://www.morphbank.net/?id=823013

33 http://www.morphbank.net/?id=823014

34 http://www.morphbank.net/?id=823015

35 http://www.morphbank.net/?id=823016

36 http://www.morphbank.net/?id=823017

37 http://www.morphbank.net/?id=823018

38 http://www.morphbank.net/?id=823019

39 http://www.morphbank.net/?id=823020

40 http://www.morphbank.net/?id=823021

41 http://www.morphbank.net/?id=823022

42 http://www.morphbank.net/?id=823023

43 http://www.morphbank.net/?id=823024

44 http://www.morphbank.net/?id=823025

45 http://www.morphbank.net/?id=823026

46 http://www.morphbank.net/?id=823027

47 http://www.morphbank.net/?id=823028

48 http://www.morphbank.net/?id=823029

49 http://www.morphbank.net/?id=823030

50 http://www.morphbank.net/?id=823031

51 http://www.morphbank.net/?id=823032

52 http://www.morphbank.net/?id=823033

53 http://www.morphbank.net/?id=823034

54 http://www.morphbank.net/?id=823035

55 http://www.morphbank.net/?id=823036

56 http://www.morphbank.net/?id=823037

57 http://www.morphbank.net/?id=823038

58 http://www.morphbank.net/?id=823039

59 http://www.morphbank.net/?id=823040

60 http://www.morphbank.net/?id=823041

61 http://www.morphbank.net/?id=823042

62 http://www.morphbank.net/?id=823043

63 http://www.morphbank.net/?id=823044

64 http://www.morphbank.net/?id=823045

65 http://www.morphbank.net/?id=823046

66 http://www.morphbank.net/?id=823047

67 http://www.morphbank.net/?id=823048

68 http://www.morphbank.net/?id=823049

69 http://www.morphbank.net/?id=823050

70 http://www.morphbank.net/?id=823051

71 http://www.morphbank.net/?id=823052

72 http://www.morphbank.net/?id=823053

73 http://www.morphbank.net/?id=823054

74 http://www.morphbank.net/?id=823055

75 http://www.morphbank.net/?id=823056

76 http://www.morphbank.net/?id=823057

77 http://www.morphbank.net/?id=823058

78 http://www.morphbank.net/?id=823059

79 http://www.morphbank.net/?id=823060

80 http://www.morphbank.net/?id=823061

81 http://www.morphbank.net/?id=823062

82 http://www.morphbank.net/?id=823063

83 http://www.morphbank.net/?id=823065

84 http://www.morphbank.net/?id=823066

85 http://www.morphbank.net/?id=823067

86 http://www.morphbank.net/?id=823068

87 http://www.morphbank.net/?id=823069

88 http://www.morphbank.net/?id=823070

89 http://www.morphbank.net/?id=823071

90 http://www.morphbank.net/?id=823072

91 http://www.morphbank.net/?id=823073

92 http://www.morphbank.net/?id=823074

93 http://www.morphbank.net/?id=823075

94 http://www.morphbank.net/?id=823076

95 http://www.morphbank.net/?id=823077

96 http://www.morphbank.net/?id=823078

97 http://www.morphbank.net/?id=823079

98 http://www.morphbank.net/?id=823080

99 http://www.morphbank.net/?id=823081

100 http://www.morphbank.net/?id=823082

101 http://www.morphbank.net/?id=823083

102 http://www.morphbank.net/?id=823084

103 http://www.morphbank.net/?id=823085

104 http://www.morphbank.net/?id=823086

105 http://www.morphbank.net/?id=823087

## Appendix I

Taxonomic records for all records used in the present paper. (doi: 10.3897/zookeys.327.5152.app1) File format: DarwinCore Archive.

## Appendix II

Locality records for all records used in the present paper. (doi: 10.3897/zookeys.327.5152.app2) File format: DarwinCore Archive.

## Appendix III

Characters. = used in phylogenetic analysis.

1. Radicle color and shade
  1. same as scape, both yellowish or reddish
  2. darker than scape
  3. same as scape, both dark brown
2. Pedicel color
  1. same as scape
  2. at least partially darker than scape
3. A3 [female]
  1. longer than pedicel
  2. shorter than pedicel
  3. as long as pedicel
4. A4 [female]
  1. longer than broad
  2. broader than long
  3. as long as broad
5. A5 [female]
  1. longer than broad
  2. broader than long
  3. as long as broad
6. Ventral clypeal margin
  1. concave
  2. uniformly convex
  3. with slightly convex median lobe
  4. with elongate median lobe
7. Interantennal process
  1. not elongate
  2. elongate
8. Lower frons at dorsal margin of interantennal process
  1. without transverse carina
  2. with transverse ledge, face sharply receding below it
  3. with oblique carina extending towards mouth corner
9. Transverse curved rugae extending from frontal depression to eye
  1. absent
  2. present
10. Median longitudinal carina in frontal depression
  1. absent
  2. present
11. Ventral portion of frontal depression
  1. with transverse carinae
  2. with medially interrupted transverse carinae
  3. with medially interrupted transverse foveae
  4. smooth
12. Dorsal portion of frontal depression
  1. without transverse carinae
  2. with some transverse carinae
  3. with medially interrupted transverse carinae
13. Submedian carina
  1. present
  2. absent
  3. present only as a weak shift in elevation
14. Frontal depression dorsally
  1. not hood-like, open dorsally
  2. hood-like, dorsally protruding
15. Upper frons major sculpture
  1. umbilicate foveate
  2. irregularly rugose
  3. transversely rugose
16. Upper frons microsculpture
  1. absent
  2. granulate
  3. punctate
17. Hyperoccipital carina
  1. absent
  2. present as a single carina
  3. indicated by a set of irregular elevations
  4. present as multiple regular elevations
18. Carina connecting occipital carina to hyperoccipital carina
  1. absent
  2. present
19. Occipital carina
  1. uniformly rounded dorsally
  2. weakly arched dorsally, with rounded lateral corners
  3. weakly arched dorsally, with sharp lateral corners
  4. broadly angular, with rounded median peak
  5. omicron-shaped, with sharp corners where median portion meets lateral portions
  6. present laterally, absent medially
20. Occiput sculpture
  1. smooth
  2. umbilicate foveate medially, becoming smooth laterally
  3. umbilicate foveate
  4. transversely rugose
  5. irregularly sculptured
  6. umbilicate punctate
21. Extra carina ventral to occipital carina
  1. absent
  2. present, complete
  3. present, medially incomplete
22. Gena length
  1. shorter than eye
  2. as long as eye
23. Major sculpture of gena anteroventrally
  1. umbilicate foveate
  2. rugose
  3. umbilicate punctate
  4. absent
24. Major sculpture of gena posteroventrally
  1. umbilicate foveate
  2. rugose
  3. umbilicate punctate
  4. absent
25. Microsculpture of gena anteroventrally
  1. absent
  2. granulate
  3. punctate
26. Microsculpture of gena posteroventrally
  1. absent
  2. granulate
  3. punctate
27. Lateral pronotal area sculpture
  1. with shallow irregular carinae, without microsculpture
  2. irregularly foveate, with smooth area dorsally
  3. with shallow irregular carinae, posterodorsal corner with dense microsculpture
  4. with a series of arched carinae, posterodorsal corner with weak longitudinal rugae
  5. anteriorly smooth, posterodorsal corner with dense microsculpture, ventral corner with irregular carinae
  6. densely covered with setiferous puncta
  7. irregularly sculptured
  8. smooth anteriorly, densely setose posteriorly
  9. mostly granulate, ventral corner with irregular carinae
28. Posterior border of central pronotal area
  1. directed anteriorly, protruding at corner of epomial carina and transverse pronotal carina
  2. directed posteriorly, epomial carina absent or meeting transverse pronotal carina at arch on lateral surface of pronotum
29. Mesoscutum anteriorly
  1. very steep and tall, descending at a right angle or protruding anteriorly
  2. not steep, forming less than a right angle
30. Major sculpture of mesoscutal midlobe anteriorly [female]
  1. umbilicate foveate
  2. longitudinally rugose
  3. transversely rugose
  4. umbilicate punctate
  5. irregularly rugose
31. Mesoscutal midlobe sculpture at midlength [female]
  1. not different from nearby sculpture
  2. with large smooth areas
  3. with narrow curved smooth elevations
32. Major sculpture of mesoscutal midlobe posteriorly [female]
  1. umbilicate foveate
  2. longitudinally rugose
  3. obliquely rugose
  4. umbilicate punctate
  5. irregularly rugose
  6. transversely rugose
33. Microsculpture of mesoscutal midlobe anteriorly [female]
  1. absent
  2. granulate
34. Microsculpture of mesoscutal midlobe posteriorly [female]
  1. absent
  2. granulate
  3. punctate
35. Median mesoscutal carina
  1. present as a narrow carina
  2. absent
  3. present as a flattened or rounded elevation
  4. present as a vague, occasionally interrupted elevation
  5. present as a ruga
36. Major sculpture of mesoscutellum centrally [female]
  1. absent
  2. umbilicate foveate
  3. longitudinally rugose
  4. obliquely rugose
  5. umbilicate punctate
  6. irregularly rugose
37. Major sculpture of mesoscutellum peripherally [female]
  1. umbilicate foveate
  2. longitudinally rugose
  3. obliquely rugose
  4. umbilicate punctate
  5. irregularly rugose
38. Microsculpture of mesoscutellum centrally [female]
  1. absent
  2. granulate
  3. punctate
39. Microsculpture of mesoscutellum peripherally [female]
  1. absent
  2. granulate
  3. punctate
40. Mesoscutellar rim
  1. not expanded
  2. expanded
41. Mesoscutellar rim medially
  1. without notch
  2. with notch
42. Mesofemoral depression
  1. longitudinally striate dorsally and ventrally
  2. longitudinally striate dorsally, smooth ventrally
  3. with slight, indistinct sculpture dorsally, smooth ventrally
  4. smooth
43. Metascutellum shape
  1. deeply emarginate, with the resulting pair of posterior processes subtriangular and directed dorsally
  2. not emarginate, forming a flat, concave shelf
  3. not emarginate, concave but elevated posteriorly
  4. slightly emarginate posteriorly, concave but elevated posteriorly
  5. not emarginate, convex dorsally
  6. weakly emarginate apically, not concave dorsally
44. Metascutellar setae
  1. absent
  2. present
45. Metascutellum sculpture [female]
  1. with large smooth posterior fovea
  2. with a median carina ending in a broad granulate patch posteriorly
  3. with a longitudinal median ruga and some weak transverse carinae
  4. with three parallel and evenly spaced longitudinal carinae
  5. with a median carina, otherwise weakly sculptured
  6. with many longitudinal rugae
  7. dense and irregular
  8. densely umbilicate foveate
46. Postmarginal vein
  1. absent
  2. present
  3. but marginal vein curving slightly at apex
47. Forewing apex position at rest [female]
  1. reaching middle of T5
  2. reaching near apex of T5
  3. reaching base of T5
  4. not reaching base of T5
  5. exceeding metasomal apex
  6. reaching middle of T6
48. Coxae
  1. same color as femora
  2. darker than femora
49. Spines along tibiae
  1. absent
  2. present
50. Lateral propodeal carinae
  1. broadly separated, not parallel anteriorly
  2. narrowly separated, subparallel anteriorly
  3. meeting near propodeal midlength
  4. broadly separated, subparallel anteriorly
51. Setae in metasomal depression
  1. present
  2. absent
52. Anterior sculpture of metasomal depression
  1. absent
  2. with median areole or pair of pits
  3. with series of longitudinal carinae extending to lateral propodeal carinae
53. Median propodeal carina
  1. absent
  2. present
54. T1 horn [female]
  1. absent
  2. present
55. Number of longitudinal carinae of T1 midlobe [female]
  1. 3
  2. 4
  3. 5
  4. 6
  5. obscured by other raised sculpture
  6. more than 6
56. T1 lateral carina
  1. straight
  2. protruding laterally, visible from ventral view
57. T2 sculpture
  1. densely foveate, longitudinal sculpture irregular
  2. with longitudinal striae or rugae, setiferous puncta present between them
  3. anteriorly longitudinally rugose with setal pits spanning interspaces, posteromedially sparsely foveate
  4. densely foveate with granulate microsculpture, longitudinal sculpture irregular
  5. mostly smooth with setiferous puncta medially, laterally with some longitudinal carinae
58. T2 sublateral longitudinal foveae [female]
  1. absent
  2. present
59. T3 metasomal flanges [female]
  1. absent
  2. present
60. T4 sculpture
  1. longitudinally striate to rugose, setal pits spanning interspaces
  2. densely foveate, longitudinal sculpture irregular
  3. mostly smooth, with tiny umbilicate foveae
61. T4 metasomal flanges [female]
  1. absent
  2. present as slightly protruding sharp corners
  3. present as strongly protruding flanges
62. T5 sculpture
  1. longitudinally striate to rugose, setal pits spanning interspaces
  2. densely foveate, interspaces not raised
  3. densely foveate, longitudinal sculpture irregular
  4. mostly smooth, with tiny umbilicate foveae
63. T5 metasomal flanges [female]
  1. absent
  2. present as slightly protruding acuminate flanges
  3. present, blade-like with rounded margins
  4. present, rounded and lobe-like
  5. present as strongly protruding acuminate flanges
  6. present as strong posterior corners
64. T6 shape [female]
  1. longer than broad
  2. broader than long
65. Major sculpture of T6 [female]
  1. umbilicate punctate
  2. longitudinally striate
  3. umbilicate foveate
66. Microsculpture of T6 [female]
  1. absent
  2. granulate
67. T6 medially [female]
  1. with medially truncate emargination, separated from apical rim
  2. with deep emargination that is V-shaped medially, separated from apical rim
  3. slightly emarginate, not separated from apical rim
  4. with medially truncate emargination, sloping down to apical rim
  5. with broad emargination between protruding posterolateral corners, separated from apical rim
  6. with a median projection set off by an abrupt narrowing posterior to tiny metasomal flanges, not separated from apical rim
  7. flat and tapering to a rounded apex, not separated from apical rim
  8. tapering to a sharp point, not separated from apical rim
  9. strongly convex, tapering and sloping down to a rounded apex, not separated from apical rim
  10. truncate, separated from apical rim
68. T6 metasomal flanges [female]
  1. absent
  2. present as tiny apical sharp projections
  3. present, very broadly rounded but with sharp apices
  4. present, very broadly rounded, with rounded apices
  5. present as slightly expanded lateral rims, rounded posteriorly
  6. present as slightly expanded lateral rims, truncate posteriorly
  7. present, broad and elongate, with slight lateral incisions near midlength
  8. present as spine-like structures posterolaterally
  9. present subapically
69. T6 raised peripheral rim [female]
  1. present
  2. absent
70. S4 sculpture
  1. longitudinally striate or rugose, setal pits spanning interspaces
  2. densely setose, setal pits between very weak longitudinal rugae
  3. sparsely foveolate, with tiny pits in interspaces
  4. smooth, with tiny umbilicate foveae
71. S5 sculpture
  1. longitudinally striate to rugose, setal pits spanning interspaces
  2. densely setose, setal pits between very weak longitudinal rugae
  3. sparsely foveate, with tiny pits in interspaces
  4. smooth, with tiny umbilicate foveae
72. S5 median carina
  1. present
  2. absent
73. S6 peripheral carina [female]
  1. present, posteriorly complete
  2. absent
74. S6 apex in relation to T6 [female]
  1. exposed to dorsal view by T6 emargination
  2. not exposed to dorsal view
75. S6 apex [female]
  1. truncate
  2. rounded or acuminate
76. A3 [male]
  1. longer than pedicel
  2. shorter than pedicel
  3. as long as pedicel
77. A5 tyloid shape
  1. narrow, linear
  2. expanded, ovate or sinuate
78. A6 [male]
  1. longer than broad
  2. broader than long
79. A11 [male]
  1. longer than broad
  2. broader than long
  3. as long as broad
80. Major sculpture of mesoscutal midlobe anteriorly [male]
  1. umbilicate foveate
  2. irregularly rugose
  3. transversely rugose
  4. umbilicate punctate
81. Major sculpture of mesoscutal midlobe posteriorly [male]
  1. umbilicate foveate
  2. irregularly rugose
  3. transversely rugose
  4. umbilicate punctate
  5. obliquely rugose
  6. longitudinally rugose
82. Microsculpture of mesoscutal midlobe anteriorly [male]
  1. absent
  2. granulate
83. Microsculpture of mesoscutal midlobe posteriorly [male]
  1. absent
  2. granulate
84. Major sculpture of mesoscutellum centrally [male]
  1. umbilicate foveate
  2. longitudinally rugose
  3. umbilicate punctate
  4. absent
  5. irregularly rugose
85. Major sculpture of mesoscutellum peripherally [male]
  1. umbilicate foveate
  2. longitudinally rugose
  3. umbilicate punctate
  4. irregularly rugose
86. Microsculpture of mesoscutellum centrally [male]
  1. absent
  2. granulate
  3. punctate
87. Microsculpture of mesoscutellum peripherally [male]
  1. absent
  2. granulate
  3. punctate
88. Forewing apex position at rest [male]
  1. reaching middle of T5
  2. reaching apex of T5
  3. exceeding metasomal apex
  4. reaching middle of T6
89. T1 midlobe longitudinal carinae [male]
  1. 3
  2. 4
  3. 5
  4. 6 or more
  5. obscured by other raised sculpture
90. T3 metasomal flanges [male]
  1. absent
  2. present
91. T4 metasomal flanges [male]
  1. absent
  2. present
92. T5 metasomal flanges [male]
  1. absent
  2. present as slightly protruding acuminate flanges
  3. present, rounded and lobe-like
  4. present as sharp corners that do not protrude
  5. present as strongly protruding acuminate flanges
93. T6 metasomal flanges [male]
  1. absent
  2. present as slightly protruding acuminate flanges
  3. present, rounded and lobe-like
  4. present as sharp corners that do not protrude
  5. present as strongly protruding acuminate flanges
  6. present as subapical tubercles
94. T7 [male]
  1. with a pair of sharply defined spine-like posterolateral projections
  2. broadly and deeply emarginate, with rounded posterolateral margins
  3. M-shaped, with a triangular median emargination
  4. weakly emarginate
  5. truncate
  6. broadly emarginate, with sharply pointed posterolateral lobes
